# On trends and patterns in macroevolution: Williston’s law and the branchiostegal series of extant and extinct osteichthyans

**DOI:** 10.1186/s12862-019-1436-x

**Published:** 2019-06-10

**Authors:** Eduardo Ascarrunz, Marcelo R. Sánchez-Villagra, Ricardo Betancur-R, Michel Laurin

**Affiliations:** 1 Department of Geosciences, University of Fribourg, Chemin du Musée 4, 1700 Fribourg, Switzerland; 2 Paläontologisches Institut und Museum, Universität Zürich, Karl-Schmid-Strasse 4, 8006 Zürich, Switzerland; 3 Department of Biology, University of Oklahoma, Norman, 73019 USA; 4 CR2P, UMR 7207 (CNRS/MNHN/Sorbonne Université), Muséum National d‘Histoire Naturelle, Bâtiment de Géologie, Case postale 48, 43 rue Buffon, F-75231, cedex 05 Paris, France

**Keywords:** Phylogeny, Palaeontology, Williston’s law, Evolutionary trend, Early burst

## Abstract

**Background:**

The branchiostegal series consists of an alignment of bony elements in the posterior portion of the skull of osteichthyan vertebrates. We trace the evolution of the number of elements in a comprehensive survey that includes 440 extant and 66 extinct species. Using a newly updated actinopterygian tree in combination with phylogenetic comparative analyses, we test whether osteichthyan branchiostegals follow an evolutionary trend under ‘Williston’s law’, which postulates that osteichthyan lineages experienced a reduction of bony elements over time.

**Results:**

We detected no overall macroevolutionary trend in branchiostegal numbers, providing no support for ‘Williston’s law’. This result is robust to the subsampling of palaeontological data, but the estimation of the model parameters is much more ambiguous.

**Conclusions:**

We find substantial evidence for a macroevolutionary dynamic favouring an ‘early burst’ of trait evolution over alternative models. Our study highlights the challenges of accurately reconstructing macroevolutionary dynamics even with large amounts of data about extant and extinct taxa.

**Electronic supplementary material:**

The online version of this article (10.1186/s12862-019-1436-x) contains supplementary material, which is available to authorized users.

## Background

The evolution of the number of skull bones has been postulated to follow a general trend towards the reduction in the number of individual parts, resulting from losses and fusions of bones. This simplification trend is known as “Williston’s law” [1], and it has recently been studied most intensively in tetrapod dermal skull bones [2–4]. For instance, a study of tetrapod skulls documented the systematic loss of bones connected to few other bones during evolution [3], emphasizing the importance of networks during growth and adult geometry [4]. In synapsid stem-mammalian lineages, a pattern of reduction in the number of skull and lower jaw bones (through either loss or fusion) during approximately 150 million years has also been described [2].

**Fig. 1:**
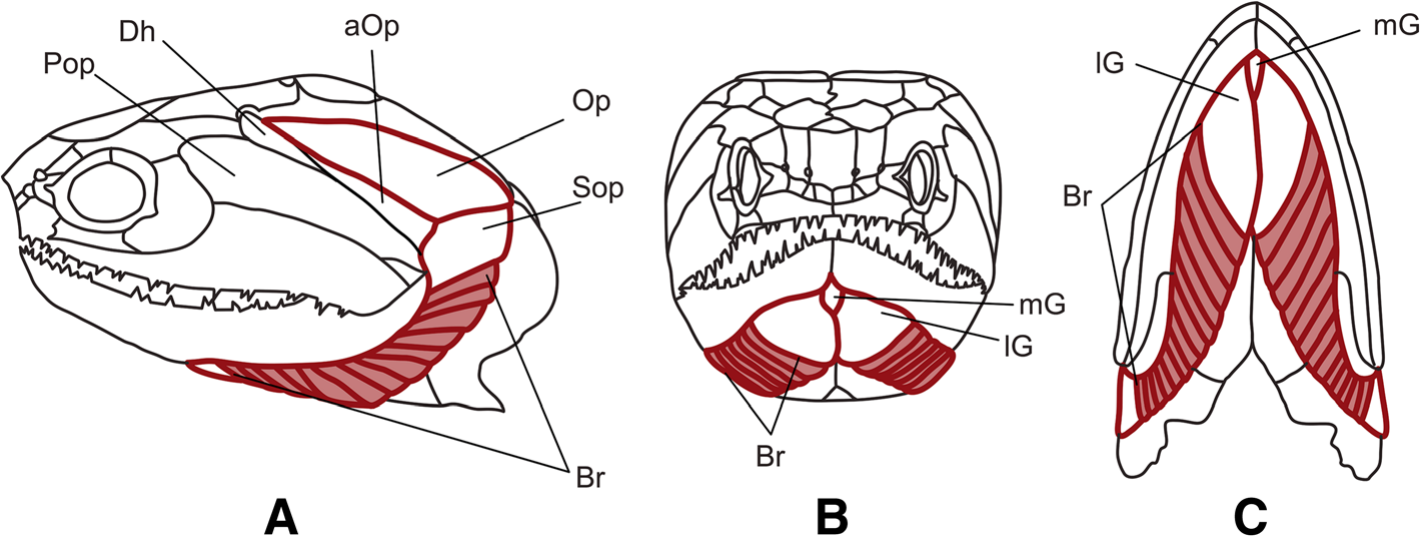
Skull of the Devonian actinopterygian *Cheirolepis trailli*^†^ in lateral (**a**), anterior (**b**), and ventral (**c**) view (after [59]). Opercular/ branchiostegal series in red outlines with branchiostegal rays in light red fill. The pattern of these bones in this stem-actinopterygian may be considered the basic actinopterygian pattern. The elements in this succession include the operculum, suboperculum, branchiostegal rays, and gulars. Some authors include also Dh dermohyale, aOp accessory operculum, Pop preoperculum (and other absent bones here) in the series, while others exclude the gulars from it (for references see text). Any of these elements may be missing, hence the synonymous names ‘opercular series’, ‘branchiostegal series’, ‘operculo-branchiostegal series’, and ‘operculo-gular series’. Op, operculum; Sop, suboperculum; Br, branchiostegal rays; lG, lateral gular; mG medial gular

**Fig. 2:**
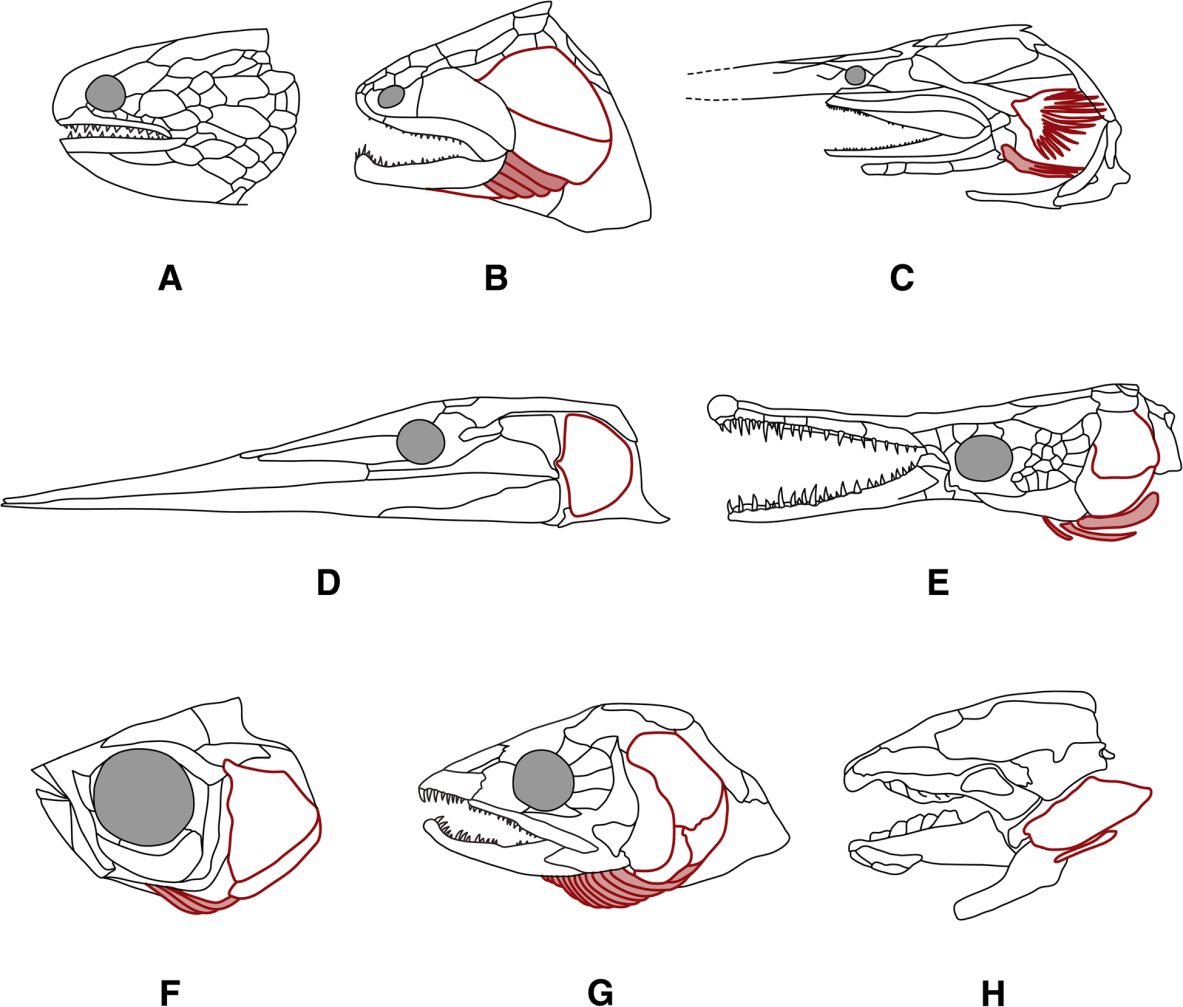
Diversity of the opercular/branchiostegal series (in red outlines; branchiostegal rays in light red fill) in osteichthyans (skulls in left lateral view); **a** *Dialipina*^†^ (Devonian; [60]). The region of the cheek and the gill cover is studded with multiple bony plates that makes it impossible to delineate an opercular/branchiostegal series [56]. **b** *Guiyu*^†^ (Silurian; [32]) showing the “standard pattern” of the opercular/branchiostegal series, including (from dorsal to ventral) operculum, suboperculum, a number of branchiostegal rays, and gular. **c** The recent paddlefish *Polydon* ([5]) without operculum, the larger bone being the suboperculum and the smaller one a single branchiostegal ray. **d** *Saurichthys*^†^ (Triassic; [11]) with a single element, the suboperculum. **e** The gar *Lepisosteus* ([5]) with operculum and suboperculum and three branchiostegals. **f** The zebrafish *Danio rerio* ([61]); as in all cypriniforms its opercular series consists of three elements. **g** The salmon *Salmo* ([6]), with variable number of branchiostegal rays (9–13), even within the same species. **h** The Australian lungfish *Neoceratodus* ([5]), with a small suboperculum and no branchiostegal rays. Elements from the opercular series may be missing (e.g. the operculum and the gulars in paddlefish, the branchiostegals in lungfish, all elements in saccopharyngiforms ([62] not shown in Fig. 2)

**Fig. 3:**
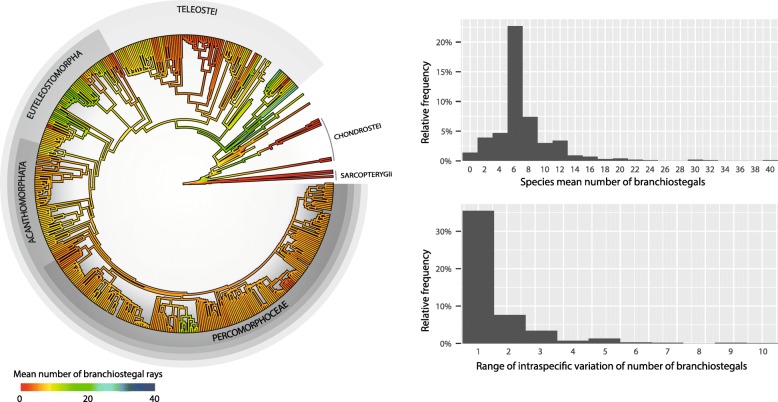
Phylogenetic distribution of mean branchiostegal ray numbers (left), and histograms of the species mean and range of branchiostegal numbers (right). The nodal ancestral values were reconstructed under the EB model, and interpolated along branches using the contMap function of the R package phytools v. 0.6 [63, 64]. Branch lengths are proportional to time. The silhouettes show the approximate position of selected clades. The age of the root is 443 Ma. Note that the intraspecific variation in the number of branchiostegals is probably underreported

Here, we evaluate the hypothesis that the elements of a meristic series of skull bones of osteichthyians, the branchiostegal ray series (BRS), followed Williston’s law. This hypothesis was first postulated by McAllister, based on a comprehensive study of the variation of the BRS in osteichthyans [5]. The BRS consists of long struts of dermal bone that form a series of elements covering the gills together with the opercular bone series ([6]; Fig. 1). The shape, relative size and the number of elements in the meristic BRS series are highly variable across osteichthyans [7–9]; Fig. 2), with fusions and losses documented in both extant and extinct species (e.g. [10–12]). While the BSR is absent in the extant species of sarcopterygians that we surveyed, the structures are well-characterized in actinopterygians or ray-finned fishes, an extraordinarily diverse group that comprises roughly half of the extant vertebrate diversity. The branchiostegal rays are mostly linked to ventilatory function, with a more prominent suction pump being coupled with a larger number of rays [13, 14]. The BRS is thus part of the buccal pump, a structure that is thought to have played a major role in the evolutionary radiation of actinopterygians [15, 16]. Branchiostegal rays can be highly variable in number and shape within species (Fig. 3; [5]), as is the case with *Oncorrhynchus nerka*, where documented variation is in the range of 10 to 20 elements. High intraspecific variation is also observed in some fossils such as *Discoserra pectinodon*^†^ [26, 27]). It has been suggested that stress can induce intra-specific variation of BRS [28], and asymmetries in left and right BRS counts have been observed in some species (e.g., the bonefish *Albula vulpes*; see [5], p. 36). Variation in the number of rays, however, is not uniformly distributed and clade-specific patterns have been documented [29, 30].

Major advances in resolving the phylogeny of actinopterygians [17, 18], and the development of statistical approaches for hypothesis-testing and model-fitting in comparative biology [19–21] provide an avenue to examine BRS count evolution. Comparative approaches have the potential to deliver major insights into the origin of species diversity and morphological disparity or evolutionary patterns in general, particularly when data from fossils and extant species are analysed in a unified phylogenetic framework [22–25]. Much of the information on the anatomy of ostheichytians is richly documented in works that can now be mined to examine how character complexes have evolved.

To test whether the evolution of the BRS conforms to Williston’s law, we used an integrative phy`logenetic framework including placement for about one thousand eight hundred extant and extinct species coupled with osteological data from about five hundred species. Two phylogenetic comparative approaches were implemented. The first approach consists of a comparison of the fit of models that can incorporate a tendency towards reduction in the count of BRS (i.e., Brownian Motion with a trend and Ornstein-Uhlenbeck) with that of other macroevolutionary models for continuous traits (e.g., Brownian Motion, without trend Early Burst, and white noise). The second approach comprises likelihood ratio tests between models that assume either symmetrical or asymmetrical transition rates across discrete character states. We further explore the effects of paleontological data on model fitting through taxon subsampling tests. Previous studies on evolutionary patterns have shown that neontological studies find the early burst model to be rare [31], whereas the palaeontological literature suggests this model may be more common [25]. Using a solid phylogenetic framework that integrates neontological and paleontological data, our study offers a rigorous statistical analysis of the effects of data on fossils in reconstructing evolutionary models based on macroevolutionary patterns.

## Methods

### Compilation of BRS data

**Fig. 4:**
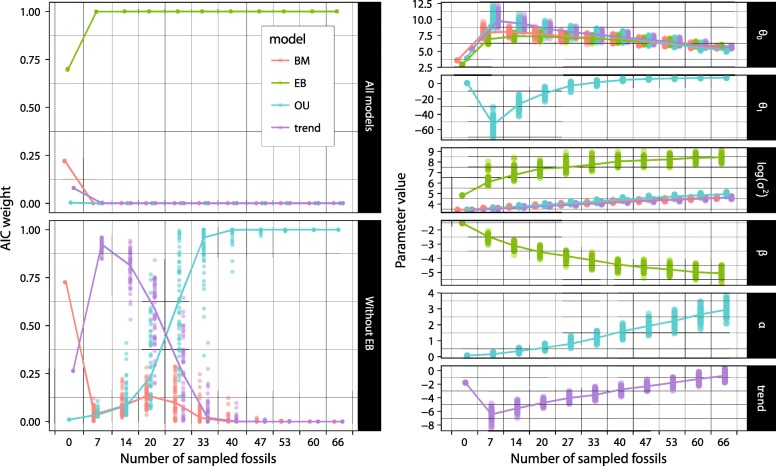
Effect of fossil sample size on model support and parameter estimates. Random subsampling of the fossil data shows that model support (Akaike weight) for EB becomes overwhelming with just 7 sampled fossils (extinct taxa), but the relative support of other models only stabilizes at some point between 40 and 47 sampled fossils (left side). In contrast, the model parameter estimates (right side) do not seem to approach an asymptote as more fossils are added, except the adaptive optimum (*θ*_1_) of the OU model and, to a lesser extent, the rate of exponential decay (β) of the EB model. Note that we introduced a small horizontal displacement in the points in order to visually separate the various model series; the analyses were performed with the fossil sample sizes labelled on the horizontal scale, with no intermediate values. Also, the Brownian diffusion rate (σ^2^) is shown log-transformed in order to better accommodate the large range of values. The lines connect the medians between sample size categories; θ_0_ is the reconstructed number of branchiostegals at the root of the tree. The white noise is not shown here because it is strongly rejected by our results (see Table 1)

We collected branchiostegal count data for 600 taxa (mostly at the species level), 506 of which are represented in our reference tree. Most of the anatomical data were taken from McAllister [5], although several other sources were also consulted (see Additional file 1). In cases where the number of branchiostegals were not stated in written form, we relied on figures, but only if the branchiostegal series was fully labelled with distinct and countable elements. For taxa in which ranges of branchiostegal numbers are reported, we used the mean value rounded off to the nearest upper integer for the discrete Markov models (see below). For many fossils, the original sources reported the branchiostegal ray counts as a minimum value with an unknown maximum, or as a point estimate with an unspecified uncertainty range (e.g. “around 10”). To accommodate those uncertainties, we arbitrarily assigned taxa lacking data on intraspecific variation to a range of variation equal to ±1 (higher than the range observed in most taxa for which polymorphism is reported; Fig. 4), defining “soft minima” and “soft maxima” for branchiostegal count values.

An anonymous referee suggested that there might be errors of homology in our data sources. While we did not attempt to validate the homology statements in every consulted publication, we see no reason to expect scattered homology errors to introduce systematic biases in our analyses. Throughout the paper we use the word “loss” of branchiostegal rays in the broad sense to refer to both losses, in the strict sense, as well as fusions. Gregory’s original formulation [1] and more recent works [2, 3] have considered the reduction in number described by Williston’s law to involve both types of processes.

### Phylogeny with extant and extinct taxa

The phylogeny used for the analyses is based on the time-scaled supertree of 1841 species (1582 extant and 259 extinct species) [23]. For a few taxa that were not represented in the tree, we used the paleontological literature for phylogenetic placement. These include extinct sarcopterygians, such as *Guiyu oneiros*^†^ [32], *Onychodus jandemarrai*^†^ [33], *Osteolepis macrolepidotus*^†^ [33], *Diplocercides*^†^ [34], *Rhabdoderma*^†^ [34], *Gyroptychius milleri*^†^ [35], and *Eusthenopteron foordi*^†^ [35]. In cases where our sources gave BRS count data at the genus or family level, we assigned the BRS count value to one of the species in the corresponding clade in our reference phylogeny. This is a potential source of error due to taxonomic instability, but we expect such errors to have a limited effect considering the higher-level phylogenetic scale of our study.

Divergence times between the added extant taxa that were not sufficiently constrained by the fossil record were set according to recent molecular studies. This concerned chiefly extant dipnoans, following Heinicke et al. [36]. The inclusion of data on extinct taxa serves both to increase statistical power and to reveal details about the dynamics of trait evolution of the clades in question [22, 37–39]. To maximize the inclusion of species for which we had information on branchiostegal counts in the phylogeny, we swapped tip values for 20 extinct congeneric species by assuming the monophyly of subtending genera. We also rescaled the tree, following a procedure similar to that of Betancur-R. et al. [23], in order to account for the stratigraphic ranges of the extinct species examined. For tree rescaling, we used the R package paleotree v. 2.7 [40], imposing a “minimum branch length” stratigraphic fit [41] of 1 My while maintaining the node ages estimated from the molecular clock analysis [42]. To take into account uncertainty in fossil ages, we generated 50 rescaled trees by randomly sampling the fossil tip ages from the span of the stratigraphic intervals in which the fossils were found (we found that sampling more than 50 trees made the analyses prohibitively slow). The 50 rescaled trees were pruned down from 1841 to 506 species for which BRS data are available.

### Macroevolutionary analyses and model fitting

By treating the number of branchiostegals as a continuous trait, we first assessed the relative fit of models of trait evolution using the R packages mvMORPH v. 1.0.7 [43] and geiger v. 2.0.6 [44]. We fitted five models (Additional file 3: Figure S1): Brownian Motion (BM), Ornstein-Uhlenbeck (OU), Early Burst (EB), BM with a trend (called “drift” in geiger), and white noise. In the simplest BM model (Additional file 3: Figure S1a), the trait evolves stochastically along the branches of the tree, where the length of the branches represents evolutionary time. At any given time, the trait value can increase or decrease following a normal probability distribution centred on 0 with variance σ^2^. The variance of the trait increases indefinitely, but because the normal distribution is symmetrical, the mean trait value of all the species in the tree will oscillate around the initial value at the root of the tree (θ0). Without an overall trend in the value of the trait, traits evolving according to Williston’s law are expected to have a poorer fit to BM than to the trend or OU models (see below).

The OU model (Additional file 3: Figure S1b) is a modified BM process where the trait is drawn towards an optimum value (θ_1_). As the trait drifts farther away from the optimum value θ_1_, it experiences a stronger pull back to it. The magnitude of that pull is controlled by an attractor strength parameter α. If the trait value at the root θ_0_ is different from θ_1_, the mean trait value of all the lineages will tend to increase or decrease over time, eventually coming to oscillate around θ_1_ (unlike BM with a trend, where the trait value increases or decreases indefinitely). Thus, the OU model can describe a Williston’s law scenario when θ_0_ > θ_1_ and α is not very small.

Early burst (EB) is another variation of BM in which the rate of evolution decreases exponentially over time, adding an extra rate change parameter β (Additional file 3: Figure S1c). Such an exponential decrease in the rate of evolution is consistent with an evolutionary radiation, in which a clade diversifies quickly to occupy vacant niches, and then slows down as niche space is filled. The EB model describes no trend in the value of the trait.

The Brownian motion model with a trend (Additional file 3: Figure S1d; “trend model” hereafter) is a slight variation of the BM model, in which the mean of the normal distribution that describes the evolution of the trait is shifted by some amount corresponding to a trend parameter [38, 45]. Negative trend parameters describe an overall decrease in the mean trait value relative to the root, as stipulated by Williston’s law for the number of bony elements. Conversely, a positive trend parameter represents an overall increase and therefore could be interpreted as evidence against Williston’s law. In the trend model the mean trait value increases or decreases indefinitely.

Finally, the “white noise” model (Additional file 3: Figure S1e) is similar to the BM model, except that it ignores phylogenetic structure. In all the species the trait has the same common initial value at the root, but it then evolves independently for each species, ignoring phylogenetic covariance. Under this model, similar trait values in closely related species are purely coincidental. As with the BM model, the white noise model does not describe any trends. A trait with very low phylogenetic signal (e.g., due to extreme rates of evolution, or strong environmental or developmental effects) is expected to fit better the white noise model than any of the other models tested.

In order to facilitate the numerical approximations of the likelihood computations, we rescaled the branches to have a total tree height of 1. Model parameter estimates are reported in the same scale throughout the paper. While OU fitting did not converge using geiger, the likelihood scores and model parameter estimates obtained through mvMORPH and geiger were virtually identical (at least down to the third decimal). Therefore, we consider the outputs of these two programs to be readily comparable but report the results with geiger for the “white noise” model and mvMORPH for all the others (“white noise” is not available in mvMORPH).

In order to determine the impact of range polymorphisms, model fitting was also performed with both soft minima and maxima of branchiostegal counts. We compared the relative fit of the models using Akaike weights. The Akaike information criterion (AIC) is a heuristic founded on information theory that balances the goodness of fit of the data (likelihood scores) and the number of parameters in the model. The AIC expressed as Akaike weights indicates the relative support for each of the models being compared; models with greater Akaike weights are preferred because they offer better trade-off between goodness of fit and number of parameters. By definition, the Akaike weights of all the models considered add up to 1.

In an alternative model-fitting framework, we also tested the presence of an overall bias to the gain or loss of branchiostegals, treating the number of branchiostegal elements as a discrete trait. This was done using symmetric (MkS) and asymmetric (MkA) Markov models of discrete state transitions (e.g., [47]). Because the number of branchiostegals is a meristic trait, both models are ordered; i.e., the only state transitions allowed are gains or losses of a single branchiostegal ray at the time. In the asymmetric model, there are two parameters: rate of branchiostegal gain and rate of branchiostegal loss. The symmetric model has a single parameter, with both gains and losses sharing the same rate. Fitting the Markov models was conducted using the *ace* function of the R package ape [48], and the best-fitting model was determined via likelihood-ratio tests.

### Assessing the robustness of model parameters: post-predictive simulations and jackknifing

The adequacy of the BM and EB models was further explored via post-predictive simulations with the R package arbutus v. 0.1 [46]. The arbutus package takes the phylogeny and the model parameters estimated from the original dataset and uses them to generate hundreds or thousands of simulated traits. The simulations are then used to compute a set of statistics that are compared to the empirical data. Finding statistics in the emprirical data that are outliers in the distribution of simulated data is indicative of poor adequacy of the model to represent certain features of the data.

We also used jackknifing to assess the impact of the size of the fossil and extant species samples in model support and parameter estimation (R code scripts are provided in an additional file [Additional file 2]). We performed two suites of jackknife analyses, one that incrementally removed all 66 fossils from the tree, and a second that incrementally removed up to 66 of the extant species examined (15% of the total 440 extant species in the tree). The incremental removals were done in 10 steps, and at each step we repeated the analysis 500 times randomly selecting different sets of species for removal.

## Results

The evolution in the number of branchiostegal rays fits best an ‘early burst’ pattern. This model has a mean Akaike weight of nearly 1, whereas all the other models combined have Akaike weights < 10^− 43^ over the 50 rescaled trees (Table 1). This indicates that the dominant feature in the macroevolutionary dynamics of the trait consists of variation in “tempo” (rate of evolution), more than biases in the directionality of the changes of the trait value (“mode”) that are the focus of this study. Among the other models, the “trend” model had a much lower support than BM (2.3–2.8x lower), and OU had by far the strongest AIC support (10^14^ times greater Akaike weight than BM). The relative support for the “white noise” model was practically null, with an Akaike weight over 30 orders of magnitude smaller than that of other models, corroborating the presence of significant phylogenetic signal in the data.

**Table 1:** Model parameters and support estimated on 50 rescaled trees (mean value ± standard deviation). White noise was fitted with geiger, OU was fitted with mvMORPH. For all the other models, the log-likelihood (lnL) and parameters of all other fitted models were practically identical between geiger and mvMORPH; the mvMORPH results are shown here. θ_0_, root state; trend, trend parameters. See the methods section for explanation of the other parameters

	θ_0_	θ_1_	σ^2^	β	α	trend	lnL	AIC	Akaike weight
BM	5.55 ± 0.09	–	100.73 ± 7.23	–	–	–	− 1374.41 ± 17.90	2752.83	2.45 × 10^−53^
EB	5.80 ± 0.09	–	4708.20 ± 1453.58	−5.04 ± 0.30	–	–	− 1252.25 ± 16.82	2510.55	1.00
OU	5.18 ± 0.14	7.49 ± 0.17	141.84 ± 15.30	–	2.98 ± 0.44	–	− 1350.80 ± 12.42	2709.67	5.80 × 10^−44^
Trend	5.60 ± 0.11	–	100.71 ± 7.24	–	–	−0.75 ± 0.62	−1374.35 ± 17.85	2754.75	9.40 × 10^−54^
White noise	7.48 ± 0.00	–	18.05 ± 0.00	–	–	–	− 1449.92 ± 0.00	2903.84	3.97 × 10^−86^

Among the models studied, “trend” and OU can be both indicative of the evolution of BRS under “Williston’s law.” The “trend” model allows an unbounded decrease in the expected trait value over time, while the OU model can also represent changing selecting regimes, in which a trait is initially subject to directional selection and then gradually shifts to stabilising selection as the trait value approaches its adaptive optimum (θ_1_). Such a shift in a selective regime would be conceivably more realistic than the “trend” model, as the number of branchiostegals has a natural lower bound of zero. In our results, while the trend parameter of the “trend” model has a negative sign, indicating that branchiostegal evolution shows a tendency toward the loss of elements, the fit of the better-supported OU model fails to corroborate such a pattern. Under OU, the adaptive optimum for the number of branchiostegals (θ_1_) is two or three elements more than the root state (θ_0_). However, the size of our fossil sample seems insufficient to allow us to estimate all parameters of the models reliably (see below). By and large, we failed to obtain strong relative support for a Williston-like dynamic by fitting continuous trait models; instead we discovered strong support for an early burst pattern of BRS evolution.

Mean Akaike weights and model parameter estimates were virtually identical whether we used the minimum, mean, or maximum counts of branchiostegal rays. However, these results were not as robust to jackknifing. As expected, our results show that fossil sample size (Fig. 4) had a greater effect than extant sample size (Additional file 4: Figure S2) on Akaike weights and model parameter estimations. The relative support of the models remains stable with the removal of up to 19 extinct taxa (29% of the total fossil sample). By contrast, jackknifing indicates that most model parameters do not seem to be near convergence as the size of the fossil subsample approaches 100%. Only the “adaptive optimum” θ_1_ parameter of the OU model, and to a lesser extent the exponential rate change parameter (β) of the EB model, seem to have reached a plateau with increasing number of sampled extinct taxa. Much worse, the Brownian diffusion parameter σ^2^ of the EB model and the attractor strength α of OU have clearly not approached the value that they would have with an exhaustive sample of extinct taxa in the complete sample (Fig. 4), which is contrary to what we expected based on similar analyses in previous studies (e.g., [49]: Fig. 4). From this, we conclude that although we can determine which models of evolution are better supported, our sampling of fossils is not comprehensive enough to allow a reliable characterisation of model parameters.

Taxa with more branchiostegals evolve faster, as shown by the S_asr_ test on the EB fit [46], which consists in regressing the absolute value of the phylogenetic independent contrasts against the corresponding nodal values. The correlation observed remains significant using either raw values or square-root transformed data (*p* < 00000.1), indicating that the effect observed is not artefactual. This means that it is easier to gain or lose one branchiostegal when several are present than when only one or two are present, which seems intuitive. Note that although we use the nomenclature from Pennell et al. [46], this test was implemented long before in the PDAP:PDTREE module of Mesquite [50].

Among the departures from the early burst model, there is a highly significant skew to the right, as shown by the D_CDF_ test on the EB fit (*p* < 0.0001). This consists in performing a Kolmogorov-Smirnov test by comparing the distribution of phylogenetic independent contrasts to that of a normal distribution with a mean of 0 and a standard deviation equal to the square root of the mean squared contrasts [46]. This suggests that a few contrasts are much larger than the rest, likely reflecting a “jump-diffusion” process, in which occasional bursts of evolution occur. In our dataset, one such large contrast is found between the Late Carboniferous *Tegeolepis clarki*^†^*,* which has 30 branchiostegals, and the slightly older *Howqualepis rostridens*^†^, which has only 13. Another large contrast is between the Jurassic stem-teleost *Pachycormus*^†^ with 40 branchiostegals, and its sister-group (crown teleosts), whose ancestral state under BM is reconstructed at about 13 branchiostegals. The former of these examples indicating abrupt evolutionary changes might possibly reflect a suboptimal choice of branch lengths subtending extinct taxa, but the latter would be more difficult to explain by this factor given that *Pachycormus*^†^ has a long branch spanning the Early Permian to Middle Jurassic, and that its sister-group is an extant clade with a reasonably well-constrained age. It is important to note, however, that the results of the D_CDF_ test remain unaffected after the removal of these few extinct taxa (*T. clarki*^†^*, H. rostridens*^†^, and *Pachycormus*^†^), suggesting that the D_CDF_ test is not sensitive to those changes alone.

The results from the jackknife analyses indicate that the root value, the adaptive optimum (*θ*_1_), and the trend parameter as functions of the number of sampled extinct taxa, all have a non-monotonic behaviour. For these three parameters (out of six studied here), the value obtained from the full fossil sample is closer to the estimate without fossils than it is to estimates based on reduced fossil samples (e.g. 7–27). In fact, the adaptive optimum estimated with few fossils is negative, which is nonsensical, as it is impossible to have a negative number of branchiostegals. These anomalous estimates may be due, in part, to the uneven distribution of sampled fossils across the phylogeny. Most of the fossils (68%) are concentrated in the region of the tree that spans early cladogenetic events (from sarcopterygians to stem teleosts). Given that the jackknife analyses were exhaustive (including 500 replicates for each number of extinct taxa removed), the results obtained were unexpected and are difficult to interpret (i.e., the procedure should have filtered out much of the random variation associated with subsampling).

Finally, when treating the number of branchiostegal rays as a discrete trait by fitting ordered Markov models, the results are in agreement with our previous analyses: a likelihood-ratio test fails to favour an asymmetric model over a symmetric one. This means that no evidence was found for a significant bias toward either loses or gains of branchiostegal elements (Additional file 5).

## Discussion

We found strong support for the EB model to describe the macroevolutionary pattern of branchiostegal ray numbers. Statistical support for this evolutionary model in empirical studies was initially elusive [31]. Harmon et al. [31] assessed the fit of three models (EB, BM, and Ornstein-Uhlenbeck) to body size (49 clades) and shape (39 clades) data. Of these, size and shape were hypothesized to have evolved according to the EB model in only a single clade, but this conclusion was weakly supported: the Akaike weight of the EB model was greater than that of the two other models, but less than 0.95. In contrast, the support for the EB model in our dataset is overwhelming; other models tested have negligible support (Akaike weight < 10^− 43^). A few previous studies have documented an EB pattern ([51]), but these did not incorporate information from the fossil record. Recent simulations showed a strong decrease in error in parameter estimation associated with incorporating fossils to such analyses [45, 52], but our results suggest that the amount and perhaps the distribution of the fossil data and possible empirical deviations from the models are also important. In this respect, our dataset is not ideal, though the Akaike weights show that our results represent one of the strongest support reported for the EB relative to BM and OU models in empirical studies.

We found no support in our dataset for Williston’s law – a trend towards the reduction in the number of bony elements over time. Our results were robust to analyses treating branchiostegal elements as either continuous or discrete via Markov models. The “trend” model was only the fourth best-supported model, far behind the EB and slightly behind the BM and OU models. The interpretation of a comparatively higher support for the OU model (relative to non-EB models) is more challenging. The fact that the estimated adaptive optimum for this model is higher than the root value (by two or three branchiostegals) is in direct contradiction with the pattern expected by Williston’s law (i.e., it shows a slight increase over time rather than a reduction). However, it should be noted that while the higher support for OU over BM can be suggestive of the presence of biases in the mode of evolution, the OU model as fitted here is unrealistic. This results from the imposition of a uniform adaptive regime, with a single adaptive optimum over the entire osteichthyan phylogeny, an assumption that is likely violated given the extraordinary diversity of osteichthyans in terms of form, function, and ecology. It seems more likely that trends toward the reduction of skeletal elements may characterize some groups of osteichthyans, but not the clade as a whole. Indeed, while McAllister [5] stated that branchiostegal ray evolution follows Williston’s law as “teleostome”-wide phenomenon, he also noted that the apparent trend towards reduction was more evident in certain groups (e.g. “palaeoniscids”, a paraphyletic group of early actinoptegyrigians), and associated to deep-sea habitats, morphology of the buccal apparatus, and a broad attachment of the branchiostegal membrane (see also [14, 30, 53]). In addition, Hubbs [30] suggested that low numbers of branchiostegals were associated to freshwater environments. Unfortunately, while we did not sample fossils for many of the clades needed to test those associations, the results of the jackknife analyses cast significant doubt on conclusions that could be drawn from low fossil sample sizes.

Our study shows the importance of fossils in documenting evolutionary patterns that would be poorly constrained based solely on neontological data [22, 45, 52, 54, 55]. This is most evident from our jackknifing analyses, which show that model support and parameter estimates are strongly affected by the inclusion of fossils (Fig. 4). This phenomenon does not correspond to a simple increase in total sample size, as performing the same analyses removing extant species instead of fossils has a comparatively negligible effect (see Additional file 3). Another example is that of the distribution of the trait among extant sarcopterygians and chondrosteans, which fails to capture the much greater diversity of their closely related extinct forms, as explained above. Extant chondrosteans and sarcopterygians have very few rays (0–3), whereas their ancient relatives had a greater and more variable number of elements (0–17 among the Paleozoic sarcopterygians, and up to 30 among the Paleozoic actinopterygians included in our dataset). These data exemplify why the EB model was strongly supported.

The fossil record is rich enough to provide a significantly more reliable reconstruction of trait evolution and model fitting than analyses that are exclusively neontological [45, 52, 54]. Our study further illustrates this phenomenon, but it is also an example of how a large paleontological sample may still be insufficient to reliably determine model parameters (Fig. 4). Given that other studies are sometimes performed with fewer fossils, a jackknifing analysis, such as the one we performed, seems advisable if conclusions are to be drawn from the specific values of these parameters. In addition to the fossil sample size, the temporal and phylogenetic distribution of the sampled fossils could have a significant impact. The ability to detect trends depends on the reconstruction of the root value, on which older fossils will have a greater effect (e.g., [45]). Also, uneven sampling of fossils across clades could give greater weight to some clades over others in the detection of a model that is intended to fit the entire tree. Our own data present such an uneven sampling, with the majority of fossils sampled for sarcopterygians and non-neopterygian actinopterygians. However, the sheer diversity in branchiostegal number of early neopterygians sampled seems to buffer against a potential bias in model selection.

Part of the failure of several model parameters to approach the asymptotic value in the jackknife analysis may be linked to the inability of candidate models to closely describe the data. In particular, the various clades in the tree appear to show diverse modes of evolution. A visual examination of the data suggests that there are clade-specific changes in the evolutionary rate, and our quantitative analyses strongly support this conclusion. This was also shown by Pennell et al.’s C_var_ test on the EB fit, which compares the coefficient of variation of the contrasts on the empirical data to that of a population of datasets generated with similar parameters (*p* < 0.0001). For instance, Paleozoic sarcopterygians have a fairly variable but often high number of branchiostegals, ranging from 0 in *Diplocercides*^†^ to 17 in *Eusthenopteron foordi*^†^ and *Gyroptychius milleri*^†^*,* but extant sarcopterygians lack branchiostgeals (*Latimeria chalumnae, Neoceratodus forsteri*, and *Lepidosiren paradoxa*). Similarly, the Paleozoic actinopterygians generally had more branchiostegals (e.g., 12 in *Cheirolepis trailli*^†^, 13 in *Cheirolepis schultzei*^†^, and 17 in *Osorioichthys marginis*^†^) than extant basal members of the clade (none in extant polypterids; a single one in *Polyodon*, and two in *Acipenser*). However, no such trend is obvious among teleosts, which form the bulk of our extant sample (although our sample of extinct taxa is less dense among teleosts than in other parts of the tree, so this conclusion is not very robust). A corollary of this heterogeneity in evolutionary rates is that in large clades (e.g., salmonids), the number of elements between closely related groups may vary substantially, whereas in other clades the number of elements remains rather constant (e.g., all 22 sampled species of cypriniforms have three branchiostegals [5]).

Our study indirectly addresses the question of whether micromery (dermal skeleton composed of small elements, often capped by a single odontode) or macromery (i.e., dermal skeleton composed of a few large elements, each of which is typically capped by several odontodes, if these are present) comprise the primitive states for osteichthyans. This question has long been debated [65] but remains unsolved (e.g. [57]). If micromery were primitive, we would expect a decrease in number of skeletal elements over time, at least early in osteichthyan history. By contrast, macromery implies the reverse prediction. In early osteichthyans, there are examples of both mainly micromeric (e.g., *Dialipina*^†^ [56]: Fig. 1a; *Cheirolepis*^†^ [57]), or mainly macromeric (e.g. *Guiyu*^†^ Fig. 1b, [32]) taxa, so this polarity is currently unclear [58]. Given that we found no support for a “trend” model, our study does not allow discriminating strongly between these hypotheses, though the fact that OU’s optimal value is inferred to be slightly greater than the root condition provides some (weak) support for the macromery hypothesis.

## Additional files

Additional file 1: Collected fossil branchiostegal data. Table. Number of branchiostegal rays in extinct (†) and extant species, and sources. The branchiostegal count data can also be found in CSV format in Additional file 2. (PDF 593 kb)

Additional file 2: Data and scripts used in the subsampling analyses. This consists of 4 files (a,b,c,d). Data files include the branchiostegal count and stratigraphic information collected for extant and extinct species (bst_analysis.csv, tabular data), and the time-scaled trees (trees.tre, Newick format) used in the analyses. The 2 R script files also included were used for running the jackniffing analyses that subsample extinct (fossil_subsampling_1.R) or extant species (extant_subsampling_1.R). The “species” column in the CSV file contains the species in the tree to which the data were mapped. The “old_label” column contains the names of the taxa as they appeared in our original sources. An anonymous referee kindly noted that our data had an erroneous value of 1 branchiostegal ray for *Lepidosiren paradoxa*, which does not have any branchiostegals in reality. We did not rerun our analyses because they are very time-consuming, and this small correction is unlikely to have any significant effect. However, we amended the CVS file that we provide here, so that the error does not propagate to any possible future uses of the data by other researchers. (ZIP 162 kb)

Additional file 3: **Figure S1.** Illustrative simulations of trait evolution under the continuous models used in this study. The black point represents the initial trait value at the root of the tree (θ_0_, set to zero in all these simulations). (PDF 44.7 kB)

Additional file 4: **Figure S2.** Results of jackkinifing of extant species. Impact of the number of extant species sampled on Akaike weight model support (Akaike weights including all models in top left, Akaike weights excluding EB in bottom left) and the parameter estimates of the macroevolutionary models (right). Refer to Fig. 4 for explanations of the model parameters. (PNG 268 kb)

Additional file 5: Table. Model fitting of Markov models using the mean number ofbranchiostegals, the minimum (observed and “soft”) number of branchiostegals, and the maximum (observed and “soft”) number of branchiostegals. Valuesreported are the median of the model log-likelihoods and rates fitted to 50recalibrated trees. Rate 1 is the rate of branchiostegal gain in the asymmetricmodel, or gain and loss in the symmetric model. Rate 2 is the rate of branchiostegalloss in the asymmetric model.. (DOCX 268 kb)
