# Supplementary material for: On trends and patterns in macroevolution: Williston’s law and the branchiostegal series of extant and extinct osteichthyans
Source: BMC Evol Biol. 2019 Jun 10;19:117. doi: 10.1186/s12862-019-1436-x (PMC6558815; doi:10.1186/s12862-019-1436-x)
Supplement: Supplementary file 1 — Collected fossil branchiostegal data. Table. Number of branchiostegal rays in extinct (†) and extant species, and sources. The branchiostegal count data can also be found in CSV format in Additional file 2. (PDF 593 kb) [file 12862_2019_1436_MOESM1_ESM.pdf]

Species	Number of branchiostegals	Citation
Cheirothrix	5-9	McAllister 1968
Dactylopogon	12	McAllister 1968
Ablennes	9-15	McAllister 1968
Acanthonemus subaureus†	(4 countable at least)	Blot 1969
Acanthurus triostegus	5	McAllister 1968
Acipenser fulvescens	2	Findeis 1997
Acropomatidae	7	McAllister 1968
Aetheodontus besanensis†	3 at least	Bürgin 1992
Agonidae	6	McAllister 1968
Akysis sp.	6	McAllister 1968
Albula vulpes	10-13	McAllister 1968
Aldrovandia affinis	10	McAllister 1968
Alepisaurus ferox	8	McAllister 1968
Alepocephalus tenebrosus	6-7	FISHBASE
Alestes	4	McAllister 1968
Allolepidotus belloti†	10	Lombardo 2001
Alosa pseudoharengus	7	McAllister 1968
Aluterus	6	McAllister 1968
Amblysemius†	20 at least	Lamber 1994
Amia calva	10-13	McAllister 1968
Ammodytes hexapterus	7	McAllister 1968
Amphilius	9	McAllister 1968
Amphistium paradoxum†	5 possibly up to 7	Blot 1969
Anaethalion angustus†	14-15	Arratia 1987
Anaethalion knorri†	21	Arratia 1988
Anarhichadidae	6-7	McAllister 1968
Anguilla rostrata	11	McAllister 1968
Anguillavis bathshebae†	15 at least	McAllister 1968
Anguillavis quadripinnis†	12 at least	McAllister 1968
Anoplogaster cornuta	8	McAllister 1968
Anoplopomatidae	6	McAllister 1968
Anotopterus pharao	7-8	McAllister 1968
Antennarius monodi†	3 at least	Carnevale et al.2006
Antennatus coccineus	6	McAllister 1968
Antigonia muelleri	7	McAllister 1968
Antigonia rubescens	6	McAllister 1968
Antimora rostrata	7	McAllister 1968
Apeltes	3	McAllister 1968
Aphredoderus sayanus	6	McAllister 1968
Apogonidae	6-8	McAllister 1968
Aracanidae	6	McAllister 1968
Arapaima gigas	10-11	McAllister 1968
Archaephippus asper†	8	Blot 1969
Archoplites clarki†	6 at leat	Smith et al. 2
Argentina	5-7	McAllister 1968
Argyropelecus	10	McAllister 1968
Ariommatidae	6	McAllister 1968
Aristostomias scintillans	18	McAllister 1968
Astroblepus sp.	4	McAllister 1968

Astyanax mexicanus	4	McAllister 1968
Ateleopus japonicus	8-9	McAllister 1968
Atherinomorus	6	McAllister 1968
Atherinopsis californiensis	5-6	FISHBASE
Auchenipteridae	6-7	McAllister 1968
Aulopus filamentosus	15	McAllister 1968
Aulorhynchus flavidus	4	McAllister 1968
Aulostomus chinensis	4	McAllister 1968
Aulostomus maculatus	4	McAllister 1968
Australosomus kochi†	7	Nielsen 1949
Auxis rochei	7	Jones 1963
Avitoluvarus eocaenicus†	5	Bannikov and Tyler 2001
Bagarius	12	McAllister 1968
Bagrus	12	McAllister 1968
Balistes	6	McAllister 1968
Barbourisia rufa	8	McAllister 1968
Bathophilus flemingi pawneei	10	McAllister 1968
Bathydraconidae	7	McAllister 1968
Bathygadus	6-7	McAllister 1968
Bathylaco nigricans	9-10	FISHBASE
Bathylagus	2	McAllister 1968
Bathymasteridae	6	McAllister 1968
Bathypterois atricolor	12	McAllister 1968
Bathysaurus	11-12	McAllister 1968
Beagiascus pulcherrimus†	14-15	Mickle et at. 2009
Belichthys minimus†	16	Hutchinson 1975
Bellwoodilabrus landinii†	6	Bannikov et al. 2010
Belonesox	6	McAllister 1968
Benthosema	9	McAllister 1968
Beryx	7-9	McAllister 1968
Birgeria stensioei†	ca.20	Romano et al. 2009
Blenniidae	5-7	McAllister 1968
Bonapartia	11-16	McAllister 1968
Boreosomus piveteaui†	8	Nielsen 1942
Bothidae	6-7	McAllister 1968
Bovichtidae	7	McAllister 1968
Bramidae	7-8	McAllister 1968
Britoichthys marizalensis†	7	De Figueiredo 2004
Brotula multibarbata	8	McAllister 1968
Brychaetus muelleri†	15	Roelling 1974
Brycon	4	McAllister 1968
Callichthys	4	McAllister 1968
Callionymidae	5-7	McAllister 1968
Calotomus preisli†	7	Bellwood 1991
Campostoma oligolepis	3	McAllister 1968
Canthigaster	5-6	McAllister 1968
Carangodes cephalus†	6 possibly 7	Blot 1969
Carangopsis brevis†	6 possibly 7	Blot 1969
Carangopsis dorsalis†	7	Blot 1969
Carpathichtys polonicus†	(7 visible)	Jerzmanska 1979
Carpiodes	3	McAllister 1968

Centrarchidae	5-7	McAllister 1968
Centrolophidae	7	McAllister 1968
Ceratias holboelli	6	McAllister 1968
Cetomimus compunctus	10	McAllister 1968
Cetomimus gilli	9	McAllister 1968
Cetopsis	8	McAllister 1968
Cetostoma regani	8	McAllister 1968
Chaca chaca	8	McAllister 1968
Chaenopsidae	6	McAllister 1968
Chaenopsis alepidota†	6	Springer 1993
Chaetodontidae	6-7	McAllister 1968
Channa striata	5	McAllister 1968
Channichthyidae	6	McAllister 1968
Chanos chanos	4	McAllister 1968
Chauliodus macouni danae	20	McAllister 1968
Chaulipareion mahengeense†	7-9	Murray and Wilson 2005
Chaunax	6	McAllister 1968
Cheilodactylidae	3-6	McAllister 1968
Cheilopogon pinnatibarbatus	10	McAllister 1968
Cheirolepis schultzei†	13	Swartz 2009
Cheirolepis traillí†	12	Pearson 1979
Chiasmodontidae	6-7	McAllister 1968
Chilomycterus	6	McAllister 1968
Chirocentrus dorab	8	McAllister 1968
Chirostomias pliopterus	22	McAllister 1968
Chlorophthalmus agassizi	8-10	McAllister 1968
Chologaster	6	McAllister 1968
Chondrosteus acipenseroides†	10	Hilton et al 2011
Cichlidae	5-6	McAllister 1968
Cionichthys dunklei†	1	Schaeffer 1967
Cirrhitidae	6	McAllister 1968
Citharinus	4	McAllister 1968
Clarias	7-9	McAllister 1968
Clinidae	5-7	McAllister 1968
Cobitis taenia	3	McAllister 1968
Coccocephalus wildi†	0	Poplin et al. 1996
Coelodus subdiscus†	2	Kriwet et al. 1999
Coelorinchus	6	McAllister 1968
Coilia nasus	11	McAllister 1968
Cololabis saira	14-15	McAllister 1968
Conger	8-10	McAllister 1968
Coregonus	7-10	McAllister 1968
Corydoras	3	McAllister 1968
Coryphaenidae	7	McAllister 1968
Coryphaenoides	6	McAllister 1968
Cottus	6	McAllister 1968
Cranoglanis	8	McAllister 1968
Creediidae	7	McAllister 1968
Crossognathus danubiensis†	21	Cavin and Grigorescu 2005
Cryptacanthodidae	6	McAllister 1968
Cryptopsaras couesii	6	McAllister 1968

Ctenognathichthys bellottii†	3 at least	Bürgin 1992
Ctenopoma	6	McAllister 1968
Culaea	3	McAllister 1968
Cyclopteridae	6	McAllister 1968
Cyclothone	10-14	McAllister 1968
Cymatogaster aggregata	6	McAllister 1968
Cynoglossus	6	McAllister 1968
Cyprinodon	5-6	McAllister 1968
Cypselurus	11-13	McAllister 1968
Cyranorhis bergeraci†	10-12	Lund et al. 1997
Dactylopteridae	6	McAllister 1968
Dactyloscopidae	6	McAllister 1968
Daedalichthys higginsi†	2	Brough 1936
Daninia spinosa†	5	Lombardo 2001
Danio rerio	3	McAllister 1968
Denticeps clupeoides	5	McAllister 1968
Dermogenys	10	McAllister 1968
Deutschenchelys micklichi†	11 around	Prokofiev 2012
Diodon	6	McAllister 1968
Diplocercides†	0	Gardiner 1984
Diplomystes	8	McAllister 1968
Diplomystus brevissimus†	9	Nelson 1970
Diplophos	11-14	McAllister 1968
Diretmus argenteus	5-9	McAllister 1968
Distichodus	3-4	McAllister 1968
Dolopichthys allector	6	McAllister 1968
Doradidae	6-7	McAllister 1968
Dorosoma cepedianum	5	FISHBASE
Drepaneidae	6	McAllister 1968
Ductor vestenae†	5	Blot 1969
Echeneis naucrates	9	McAllister 1968
Electrophorus electricus	4	McAllister 1968
Eleotridae	4-6	McAllister 1968
Elops saurus	26-33	FISHBASE
Emmelichthyidae	6-7	McAllister 1968
Enchelurus syriacus†	8 at least	McAllister 1968
Engraulis	9-14	McAllister 1968
Enischnorhynchus dallasensis†	16	Bardack 1965
Enoplosidae	7	McAllister 1968
Eohiodon falcatus†	10	Grande 1979
Eohiodon rosei†	7-10	Guo-Qing et al. 1997b
Eohiodon woodruffi†	8-10	Guo-Qing et al. 1997b
Eolates gracilis†	7	Otero 2004
Eolates racilis†	7	Otero 2004
Eoplatax papilio†	8	Blot 1969
Ephippidae	6-7	McAllister 1968
Erpetoichthys	0	McAllister 1968
Esox americanus	11-16	Wilson 1984
Esox lucius	11-20	Wilson 1984
Esox tiemani†	11 right 12 left	Wilson 1984
Eucinostomus	6	McAllister 1968

Eurypharyngidae	0	McAllister 1968
Eusthenopterus foordi†	8+ 7 submandibulars	Jarvik 1980
Eustomias	16	McAllister 1968
Evenkia eunotoptera†	6	Xu et al. 2016
Evermannella balbo	8	McAllister 1968
Exellia velifer†	7 at least	Blot 1969
Exocoetus	10-11	McAllister 1968
Fistularia petimba	5	McAllister 1968
Frippia labroiformis†	6	Bannikov et al. 2012
Fukangichthys longidorsalis†	7	Xu et al. 2014
Fundulus	6	McAllister 1968
Furo trottii†	9	Lombardo 2001
Gadomus	7	McAllister 1968
Gadus morhua	8	Grygory WK. 2002/1933
Gaidropsarus	7	McAllister 1968
Gaidropsarus pilleri†	6 or7	Carnevale et al 2013
Galaxias maculatus	5	McAllister 1968
Gambusia	6	McAllister 1968
Gasteropelecus	4	McAllister 1968
Gasterosteus aculeatus	3	McAllister 1968
Gempylidae	7-8	McAllister 1968
Genypterus	7	McAllister 1968
Gephyroberyx	8	McAllister 1968
Gigantactis	6	McAllister 1968
Gigantura chuni	0	McAllister 1968
Gigantura indica	0	McAllister 1968
Glarithurus friedmani†	5	Tyler et al. 2011
Glaucolepis stensioei†	15	Nielsen 1942
Glaucosomatidae	7	McAllister 1968
Glyptocephalus zachirus	7	McAllister 1968
Gobiesox maeandricus	6	McAllister 1968
Gobiidae	3-5	McAllister 1968
Godsilia lanceolata†	? 2 visible	Monsch 2006
Gogosardina coatesi†	8 at least	Choo et al. 2009
Gonorynchus	4-5	McAllister 1968
Gonostoma	10-14	McAllister 1968
Gornylistes prodigiosus†	2 at least	Bannikov and Tyler 2008
Griphognathus whithei†	3+1 median submand.	Campbell et al.
Guildayichthys carnegiei†	7	Lund 2000
Guiyu oneiros†	3	Zhu et al. 2009
Gymnarchidae	4	McAllister 1968
Gymnotus carapo	4	McAllister 1968
Gyrinocheilus aymoneri	3	McAllister 1968
Gyroptychius milleri†	8	Jarvik 1948
Haemulidae	6-7	McAllister 1968
Halargyreus	7	McAllister 1968
Halosauropsis macrochir	11	McAllister 1968
Harpadon	17-26	McAllister 1968
Harpagiferidae	5	McAllister 1968
Helichthys browni†	1	Brough 1936
Helostoma	6	McAllister 1968

Hemiodus	4-5	McAllister 1968
Hemiramphus	10-14	McAllister 1968
Hemisilurus	10-12	McAllister 1968
Heterandria	5-6	McAllister 1968
Heteronectes chaneti†	6 at least	Friedman 2012
Heteropneustes fossilis	7-8	McAllister 1968
Heteropriacanthus cruentatus	6	McAllister 1968
Hexagrammos	6	McAllister 1968
Himantolophus	6	McAllister 1968
Hiodon alosoides	9	McAllister 1968
Hiodon consteniorum†	8	Guo-Qing and Wilson 1994
Hiodon tergisus	8-9	McAllister 1968
Hippoglossus	7	McAllister 1968
Histrio	6	McAllister 1968
Holocentrus	8	McAllister 1968
Hoplostethus	8	McAllister 1968
Howqualepis rostridens†	13	Long 1988
Hypentelium	3	McAllister 1968
Hypomesus	6-8	McAllister 1968
Hypophthalmichthys molitrix	3	McAllister 1968
Hypoptychidae	4	McAllister 1968
Hyporhamphus	13	McAllister 1968
Icosteus aenigmaticus	6-7	McAllister 1968
Ictalurus	8-9	McAllister 1968
Ictiobus	3	McAllister 1968
Ijimaia antillarum	7	McAllister 1968
Ilisha elongata	6	Nelson 1970
Indostomidae	5-6	McAllister 1968
Ipnops agassizi	10	McAllister 1968
Ipnops murrai	10	McAllister 1968
Iso rhothophilus	6	McAllister 1968
Istiophoridae	7	McAllister 1968
Kentuckia deani†	>7	Rayner 1951
Knightia eocaena† and K. alta†	8 about	Grande 1982
Kuhlia	6	McAllister 1968
Kurtus indicus	7	McAllister 1968
Kyphosichthys grandei†	5	Xu and Wu 2012
Kyphosidae	6-7	McAllister 1968
Labridae	5-6	McAllister 1968
Laemonema	7	McAllister 1968
Laminospondylus transversus†	16 or more	McAllister 1968
Lampadena	9-10	McAllister 1968
Lampanyctus macdonaldi	9	McAllister 1968
Lampris	6-7	McAllister 1968
Lateopisciculus turrifumosus†	5	Murray and Wilson 1996
Lates calcarifer	7	Otero 2004
Lates microlepis	7	Otero 2004
Latimeria chalumnae	0	McAllister 1968
Leiognathidae	5-6	McAllister 1968
Lepadichthys	6	McAllister 1968
Lepidion	7-8	McAllister 1968

Lepidoblepharon ophthalmolepis	6	McAllister 1968
Lepidopsetta bilineata	7	McAllister 1968
Lepidorhombus	7	McAllister 1968
Lepidosiren	1	McAllister 1968
Lepisosteus osseus	3	McAllister 1968
Lepophidium	7	McAllister 1968
Leptobramidae	6	McAllister 1968
Leptolepis sprattiformis†	18	Nelson 1970
Leptostomias bermudensis	19	McAllister 1968
Lethrinidae	6	McAllister 1968
Limanda	7	McAllister 1968
Limnomis delaneyi†	10-12	Daeschler 2000
Lineagruan judithi†	4-6	Mickle et at. 2009
Lineagruan snowyi†	12	Mickle et at. 2009
Liobagrus	12	McAllister 1968
Liparis	6	McAllister 1968
Lobotidae	6	McAllister 1968
Lophiodes	6	McAllister 1968
Lophius	6	McAllister 1968
Loricariidae	4	McAllister 1968
Lota lota	7-8	McAllister 1968
Luciobrama macrocephalus	3	McAllister 1968
Luganoia lepidosteoides†	3 at least	Bürgin 1992
Luoxiongichthys hyperdorsalis†	4-8	Wen 2012
Lutjanidae	6-7	McAllister 1968
Luxilus coccogenis	3	McAllister 1968
Lycoptera davidi†	10 ? from fig.	Hilton 2003
Lycoptera middendorffi†	10	McAllister 1968
Lyopsetta exilis	7	McAllister 1968
Macrognathus	6	McAllister 1968
Macropinna	3	McAllister 1968
Macrosemius fourneti†	8	Ebert et al. 2016
Mahengichthys singidaensis†	2 at least	Davis et al. 2013
Malacanthidae	6	McAllister 1968
Malacocephalus	7	McAllister 1968
Malacosteus	8	McAllister 1968
Malapterurus	6	McAllister 1968
Mallotus	8-10	McAllister 1968
Margrethia obtusirostra	13	McAllister 1968
Mastacembelus	6	McAllister 1968
Maurolicus	9-10	McAllister 1968
Mcconichthys longipinnis†	6	Grande 1988
Megalops atlanticus	23-25	McAllister 1968
Melamphaes	8	McAllister 1968
Melanocetus	6	McAllister 1968
Melanogrammus	7	McAllister 1968
Melanostomias spilorhynchus	13	McAllister 1968
Melanotaenia	6	McAllister 1968
Mene maculata†	7	Blot 1969
Mene purdyi†	(3 of) 7	Friedman 2005
Mene rhombea†	5 probably 7 or 8	Blot 1969

Menidae	7	McAllister 1968
Menidia menidia	6	McAllister 1968
Menticirrhus undulatus littoralis	7	McAllister 1968
Meridensia meridensis†	6-7	Bürgin 1992
Merluccius productus	7	McAllister 1968
Mesopoma planti†	7-8	Coates 1999
Microdesmidae	4-6	McAllister 1968
Microstomus pacificus	7	McAllister 1968
Mimia toombsi†	12	Gardiner 1984
Mimipiscis bartrami†	9 at least	Choo 2012
Mola mola	6	McAllister 1968
Monocentris japonicus	8	McAllister 1968
Monodactylidae	6-7	McAllister 1968
Monopterus albus	6	McAllister 1968
Moythomasia durgaringa†	12	Gardiner 1984
Moythomasia durgaringa†	12	Choo 2016
Moythomasia lineata†	10 at least	Choo 2015
Mugilidae	5-6	McAllister 1968
Mullidae	4-5	McAllister 1968
Muraenesox	16-22	McAllister 1968
Myctophum	8-12	McAllister 1968
Mylopharyngodon piceus	3	McAllister 1968
Myrichthys	32	McAllister 1968
Mystus	9-13	McAllister 1968
Nandidae	6	McAllister 1968
Nansenia	3-4	McAllister 1968
Nematistiidae	7-8	McAllister 1968
Nematogenys	12	McAllister 1968
Nemichthys scolopaceus	8-15	McAllister 1968
Nemipteridae	5-7	McAllister 1968
Neoceratodus forsteri	0	McAllister 1968
Neochanna	6-7	McAllister 1968
Neoclinus nudus†	6	Springer 1993
Neoscopelus macrolepidotus	9	McAllister 1968
Nomeidae	6	McAllister 1968
Notacanthus chemnitzii	9	McAllister 1968
Notemigonus crysoleucas	3	McAllister 1968
Notropis asperifrons	3	McAllister 1968
Novumbra hubbsi	6-7	McAllister 1968
Odacidae	5	McAllister 1968
Omosudis lowei	8	McAllister 1968
Oncorhynchus ketopsis†	16	Eiting et al. 2007
Oncorhynchus nerka	10-20	McAllister 1968
Oneirodes	6	McAllister 1968
Onychodus jandemarrai†	1 modified	Andrews et al.2005
Ophidion	8-10	McAllister 1968
Opistognathidae	6	McAllister 1968
Oplegnathidae	6-7	McAllister 1968
Opsanus	6	McAllister 1968
Opsariichthys uncirostris bidens	3	McAllister 1968
Orthogonikleithrus francogalliensis†	12	Konwert 2016

Oryzias latipes	6	McAllister 1968
Osmerus mordax	7	McAllister 1968
Osorioichthys marginis†	17	Taverne 1997
Osphronemidae	6	McAllister 1968
Osteoglossum bicirrhosum	11	McAllister 1968
Osteolepidotus panderi†	4	Jarvik 1948
Osteolepis macrolepidotus†	7	Jarvik 1948
Ostracion	6	McAllister 1968
Ottaviania leptacanthus†	7 complete?	Bannikov 2006
Oxyporhamphus	10	McAllister 1968
Pachyamia latimaxillaris†	17 at least	Chalifa and Tchernov
Pachycormus macropterus†	40 about	Lehman J-P. 1949
Palaeoesox fritzschi†	6	McAllister 1968
Pangasiidae	7-11	McAllister 1968
Pantodon buchholzi	8 (9?)	McAllister 1968
Paraclinus walkeri†	7	Springer 1993
Paralepis coregonoides	7	McAllister 1968
Paralichthys lethostigma	7	McAllister 1968
Paralycoptera wui†	ca. 13	Xu et al. 2009
Paramblypterus decorus†	9	Dietze 2000
Paramblypterus duvernoyi†	8	Dietze 2000
Paramblypterus gelberti†	10	Dietze 2000
Paratrachichthys	8	McAllister 1968
Paratrachinotus tenuiceps†	6 suspicious 7	Blot 1969
Paraulopus	8	McAllister 1968
Parenchodus longipterygius†	11	Raab et al. 1987
Parodon	4	McAllister 1968
Pasaichthys pleuronectiformis†	5	Blot 1969
Pavelichthys daniltshenkoi†	10 about	Prokofiev 2007
Peipiaosteus pani†	5	Hilton et al 2011
Pellona flavipinnis	6	Nelson 1970
Peltoperleidus bellipinnis†	5-6 about	Bürgin 1992
Peltopleurus lissocephalus†	5-6 about	Bürgin 1992
Peltopleurus rugosus†	5-6	Bürgin 1992
Pempheridae	6-7	McAllister 1968
Peprilus	6	McAllister 1968
Perca fluviatilis	7	McAllister 1968
Percichthyidae	7	McAllister 1968
Percophidae	5-7	McAllister 1968
Percopsis omiscomaycus	6	McAllister 1968
Percopsis transmontana	6	McAllister 1968
Peripeltopleurus vexillipinnis†	5-6 about	Bürgin 1992
Peristediidae	7	McAllister 1968
Perleidus altolepis†	6	Lombardo 2001
Perleidus woodwardi†	9	Gregory 2002/1933
Phareodus encaustus†	9-10	Guo-Qing et al. 1997a
Phenacobius uranops	3	McAllister 1968
Pholidichthyidae	4-6	McAllister 1968
Pholidopleurus ticinensis†	12 about	Bürgin 1992
Photonectes margarita	13	McAllister 1968
Pimephales promelas notatus	3	McAllister 1968

Pinguipedidae	6	McAllister 1968
Platichthys stellatus	7	McAllister 1968
Platycephalidae	7	McAllister 1968
Platysiagum minus†	7-8	Bürgin 1992
Plecoglossus	5-6	McAllister 1968
Plesiopidae	5-6	McAllister 1968
Pleuronectes	7	McAllister 1968
Plotosus	11-13	McAllister 1968
Poecilia	5-6	McAllister 1968
Pollichthys	11-12	McAllister 1968
Polycentridae	6	McAllister 1968
Polydactylus	7	McAllister 1968
Polymixia	7	McAllister 1968
Polyodon spathula	1	Findeis 1997
Polypterus	0	McAllister 1968
Pomacanthidae	6-7	McAllister 1968
Pomacentridae	4-6	McAllister 1968
Pomatomidae	7	McAllister 1968
Porichthys notatus	6	McAllister 1968
Poromitra	8	McAllister 1968
Prionotus carolinus	7	McAllister 1968
Prohollardia avita†	6	Tyler et al. 1993
Protacanthodes nimesensis†	5 at least	Tyler and Bannikov 2011
Protopsephurus liui†	3	Hilton et al 2011
Psammoperca waigiensis	7	Otero 2004
Psettichthys melanostictus	7	McAllister 1968
Psettodes	7	McAllister 1968
Psettopsis subarcuatus†	6 quite certainly 7	Blot 1969
Pseudauxides speciosus†	5 at least	Monsch 200
Pseudeutropius syn. Clupisoma	8	McAllister 1968
Pseudochromidae	5-7	McAllister 1968
Pseudogramma polyacantha	7	McAllister 1968
Pseudomugil	6	McAllister 1968
Pseudorhombus	6-7	McAllister 1968
Psychrolutidae	7	McAllister 1968
Ptycholepis barboi†	7	Bürgin 1992
Ptycholepis priscus†	8	Bürgin 1992
Pungitius pungitius	3	McAllister 1968
Rachycentridae	7	McAllister 1968
Ranzania laevis	5	McAllister 1968
Regalecus	6	McAllister 1968
Remora osteochir australis	8	McAllister 1968
Retropinna	5-6	McAllister 1968
Rhabdoderma†	0	Gardiner 1984
Rhinesomus triqueter	6	McAllister 1968
Rhinichthys cataractae	3	McAllister 1968
Rhombichthys intoccabilis†	6 about probably more	Khalloufi 2010
Rondeletia bicolor	8	McAllister 1968
Rondeletia loricata	8	McAllister 1968
Rouleina	6	McAllister 1968
Saccopharyngidae	0	McAllister 1968

Sagamichthys abei	8	FISHBASE
Salangichthys	4	McAllister 1968
Salmo	9-13	McAllister 1968
Salvelinus	8-14	McAllister 1968
Sangiorgioichthys sui†	8-9	Lopez-Arabello 2011
Sargocentron diadema	8	McAllister 1968
Saurichthys curionii†, S.costasquamosus†	1?	Rieppel 1985
Saurichthys dawaziensis†	1	Wu et al. 2009
Saurichthys madagascariensis†	ca.3	Lehmann 1952
Saurida undusquamis	16	McAllister 1968
Scanilepis dubia†	9-11	Xu et al. 2015
Scaphirhynchus albus	2	Findeis 1997
Scaphirhynchus platorynchus	2	Findeis 1997
Scaridae	5	McAllister 1968
Scatophagidae	6-7	McAllister 1968
Scatophagus frontalis†	5 possibly 6	Blot 1969
Scomberesox	14	McAllister 1968
Scopelarchidae	6-8	McAllister 1968
Scopelengys tristis	8	McAllister 1968
Scopelogadus	8	McAllister 1968
Scophthalmus aquosus	7	McAllister 1968
Scorpaenidae	6-8	McAllister 1968
Semionotus elegans†	8	Olsen and McCune 1991
Semotilus atromaculatus	3	McAllister 1968
Seriola prisca†	7	Blot 1969
Serrivomer	7-8	McAllister 1968
Siganidae	5	McAllister 1968
Sillaginidae	6	McAllister 1968
Sinosaurichthys longimedialis†, S.minuta†	0	Wu et al. 2011
Sinosaurichthys longipectoralis†	1	Wu et al. 2011
Solea	6-7	McAllister 1968
Soleichthys	6	McAllister 1968
Sorbinichthys africanus†	5	Murray and Wilson 2011
Spaniodon latus†	15-16	Taverne et al. 2003
Sphyraenidae	7	McAllister 1968
Spinachia	3	McAllister 1968
Squaliobarbus curriculus	3	McAllister 1968
Stegotrachelus finlayi†	12	Swartz 2009
Steindachneria	7	McAllister 1968
Stemonosudis	7	McAllister 1968
Stenotomus caprinus	6	McAllister 1968
Stephanolepis	6	McAllister 1968
Stichaeidae	6	McAllister 1968
Stomias	14-19	McAllister 1968
Stylephorus	5	McAllister 1968
Sudis	7-9	McAllister 1968
Sufflamen	6	McAllister 1968
Synanceiidae	6-7	McAllister 1968
Syngnathus fuscus	2	McAllister 1968

Synodontis	6-7	McAllister 1968
Synodus foetens	17	McAllister 1968
Tactostoma macropus	13	McAllister 1968
Talismania bifurcata	7	McAllister 1968
Tanakia lanceolata himantegus	3	McAllister 1968
tanulus seychellensis†	6	Springer 1993
Tegeolepis clarki†	30 about	Dunkle et al. 1973
Tetraodon	5-6	McAllister 1968
Thaleichthys	6-8	McAllister 1968
Thoracocharax	5	McAllister 1968
Thursius macrolepidotus†	8 probably (5 visib.)	Jarvik 1948
Thursius pholidotus†	8	Jarvik 1948
Thymallus	8-12	McAllister 1968
Tomognathus mordax†	7 at least	McAllister 1968
Toxotidae	7	McAllister 1968
Trachinocephalus myops	16	McAllister 1968
Trachinotus falcatus	8	McAllister 1968
Trachipterus	6	McAllister 1968
Trachonurus	7	McAllister 1968
Trematomus bernacchii	6	McAllister 1968
Triacanthus	6	McAllister 1968
Trichiuridae	7-8	McAllister 1968
Trichomycterus sp.	7-8	McAllister 1968
Tripterygiidae	6-7	McAllister 1968
Tripterygion tripteronotus†	6	Springer 1993
Tylosurus	10	McAllister 1968
Umbra limi	4-5	McAllister 1968
Uranoscopidae	6	McAllister 1968
Urophycis chuss	7	McAllister 1968
Urophycis tenuis	7	McAllister 1968
Vomeropsis trurus†	7 but certainly 8	Blot 1969
Watsonulus eugnathoides†	12-14	Olsen 1984
Wendyichthys dicksonii†	12-13	Lund et al. 1997
Xanthichthys	6	McAllister 1968
Xenocypris argentea	3	McAllister 1968
Xenodermichthys	6-7	McAllister 1968
Xenomystus	3	McAllister 1968
Xiphiidae	7	McAllister 1968
Xixiaichthys tongxinens†	13	Jiang-Yong 2004
Zacco sieboldii platypus	3	McAllister 1968
Zanclidae	4	McAllister 1968
Zaproridae	6	McAllister 1968
Zeus faber	7	McAllister 1968
Zoarcidae	4-7	McAllister 1968

References

Andrews SM, Long J, Ahlberg PE, Barwick R, Campbell K. The structure of the sarcopterygian Onychodus jandemarrai n. sp. from Gogo, Western Australia: with a functional interpretation of the skeleton. Trans. R. Soc. Edinb. Earth Sci 2006;96:197-307.

Arratia G. Anaethalion and similar teleosts (Actinopterygii, Pisces) from the Late Jurassic (Tithonian) of southern Germany and their relationships. Palaeontographica A. 1987;200:1–44.

Bannikov AF, Carnevale G. Bellwoodilabrus landinii n. gen., n. sp., a new genus and species of labrid fish (Teleostei, Perciformes) from the Eocene of Monte Bolca." Geodiversitas 2010;32(2):201-220.

Bannikov AF, Carnevale G. Frippia labroiformis n. gen. n. sp., a new perciform fish from the Eocene of Pesciara di Bolca, Italy. Bollettino della Società Paleontologica Italiana 2012;51(3):156-165.

Bannikov AF, Tyler JC. A new genus and species of triggerfish from the middle Eocene of the northern Caucasus, the earliest member of the Balistidae (Tetraodontiformes). Paleontological Journal. 2008;42:615–620.

Bannikov AF, Tyler JC. A new species of the luvarid fish genus Avitoluvarus (Acanthuroidei: Perciformes) from the Eocene of the Caucasus in southwest Russia. PROCEEDINGS-BIOLOGICAL SOCIETY OF WASHINGTON. 2001;114:579–588.

Bannikov AF. Fishes from the Eocene of Bolca, northern Italy, previously classified in the Sparidae, Serranidae and Haemulidae (Perciformes). Geodiversitas 2006;28(2):249-275.

Bardack D. New Upper Cretaceous Teleost fish from Texas. The University of Kansas Paleontological Contributions 1965;1:1-9

Bellwood DR, Schultz O. A review of the fossil record of the parrotfishes (Labroidei: Scaridae) with a description of a new Calotomus species from the Middle Miocene (Badenian) of Austria. Ann. Naturhist. Mus. Wien1991;92:55-71.

Blot J. Les poissons fossiles du monte Bolca classés jusqu'ici dans les familles des Carangidae Menidae Ephippidae Scatophagidae. Museo civico di Verona. Memorie fuori serie 1969;2:1-525.

Brough J. On the evolution of bony fishes during the Triassic period. Biological Reviews 1936;11(3):385-405.

Bürgin T. Basal ray-finned fishes (Osteichthyes; Actinopterygii) from the Middle Triassic of Monte San Giorgio (Canton Tessin, Switzerland). Schweiz Paläontolog Abh 1992;114:1-164.

Campbell KSW, Barwick RE. Paleozoic Lungfishes-A Review. Journal Of Morphology Supplement 1986;1:93-131

Carnevale G, Pietsch TW. Filling the gap: a fossil frogfish, genus Antennarius (Teleostei, Lophiiformes, Antennariidae), from the Miocene of Algeria Journal of Zoology 2006;270:448–457

Carnevale G. Harzhauser M. Middle Miocene rockling (Teleostei, Gadidae) from the Paratethys (St.Margarethen in Burgenland, Austria). Bulletin of Geosciences 2013;88(3):609–620

Cavin L, Grigorescu D. A new Crossognathus (Actinopterygii, Teleostei) from the Lower Cretaceous of Romania with comments on Crossognathidae relationships. Geodiversitas. 2005;27:5–16.

Chalifa Y, Tchernov E. Pachyamia latimaxillaris, new genus and species (Actinopterygii: Amiidae), from the Cenomanian of Jerusalem. Journal of Vertebrate Paleontology. 1982;2:269–85.

Choo B, Long JA, Trinajstic K. A new genus and species of basal actinopterygian fish from the Upper Devonian Gogo Formation of Western Australia. Acta Zoologica. 2009;90:194–210.

Choo B. A new species of the Devonian actinopterygian Moythomasia from Bergisch Gladbach, Germany, and fresh observations on M. durgaringa from the Gogo Formation of Western Australia. Journal of Vertebrate Paleontology. 2015;35:e952817.

Choo B. Revision of the actinopterygian genus Mimipiscis (=Mimia) from the Upper Devonian Gogo Formation of Western Australia and the interrelationships of the early Actinopterygii. Earth and Environmental Science Transactions of The Royal Society of Edinburgh. 2012;102:77–104.

CoatesMI. Endocranial preservation of a Carboniferous actinopterygian from Lancashire, UK, and the interrelationships of primitive actinopterygians. Philosophical Transactions of the Royal Society of London. Series B, Biological Sciences 1999;354:435–462.

Daeschler EB. An early actinopterygian fish from the Catskill Formation (Late Devonian, Famennian) in Pennsylvania, USA. Proceedings of The Academy of Natural Sciences of Philadelphia 2000;150:181-192.

Davis MP, Arratia G, Kaiser TM. The first fossil shellear and its implications for the evolution and divergence of the Kneriidae (Teleostei: Gonorynchiformes). Mesozoic Fishes. 2013;325–362.

De Figueiredo FJ. A new euteleostean fish from the Lower Cretaceous of Tucano Basin, north-eastern Brazil. Arquivos do Museu Nacional Rio de Janeiro 2004;62(3):293-307.

Dietze K. A revision of paramblypterid and amblypterid actinopterygians from Upper Carboniferous–Lower Permian lacustrine deposits of Central Europe. Palaeontology 2000;43(5):927-966.

Dunkle DH, Schaeffer B. Tegeolepis clarki (Newberry), a palaeonisciform from the Upper Devonian Ohio Shale. Palaeontographica Abteilung A 1973;143:151–158. Cited in: Swartz 2009

Ebert M, Lane JA, Kölbl-Ebert M. Palaeomacrosemius thiollieri, gen. et sp. nov., a new Macrosemiidae (Neopterygii) from the Upper Jurassic of the Solnhofen Archipelago (Germany) and Cerin (France), with a revision of the genus Macrosemius. Journal of Vertebrate Paleontology. 2016;36:e1196081.

Eiting T P, Smith GR. Miocene salmon (Oncorhynchus) from Western North America: Gill Raker evolution correlated with plankton productivity in the Eastern Pacific. Palaeogeography, Palaeoclimatology, Palaeoecology , 2007;249(3):412-424.

Findeis EK. Osteology and phylogenetic interrelationships of sturgeons (Acipenseridae). Environmental Biology of Fishes. 1997;48:73–126.

[FISHBASE] Froese, R. and D. Pauly. Editors. World Wide Web electronic publication. FishBase. 2017. www.fishbase.org.

Friedman M, Johnson GD. A new species of Mene (Perciformes: Menidae) from the Paleocene of South America, with notes on paleoenvironment and a brief review of menid fishes. Journal of Vertebrate Paleontology2005;25(4):770-783.

Friedman M. Osteology of †Heteronectes chaneti (Acanthomorpha, Pleuronectiformes), an Eocene stem flatfish, with a discussion of flatfish sister-group relationships. Journal of Vertebrate Paleontology. 2012;32:735–56.

Grande L. A revision of the fossil genus Knightia, with a description of a new genus from the Green River formation (Teleostei, Clupeidae). American Museum novitates 1982;2731:1-22

Grande L. A Well Preserved Paracanthopterygian Fish (Teleostei) from Freshwater Lower Paleocene Deposits of Montana. Journal of Vertebrate Paleontology. 1988;8:117–30.

Grande, L. (1979). Eohiodon falcatus, a new species of hiodontid (Pisces) from the late Early Eocene Green River Formation of Wyoming. Journal of Paleontology1979;53(1):103-111.

Guo-Qing L, Grande L, Wilson MV. The species of† Phareodus (Teleostei: Osteoglossidae) from the Eocene of North America and their phylogenetic relationships. Journal of Vertebrate Paleontology. 1997a;17:487–505.

Guo-Qing L, Wilson MVH, Grande L. Review of Eohiodon (Teleostei: Osteoglossomorpha) from Western North America, with a Phylogenetic Reassessment of Hiodontidae. Journal of Paleontology. 1997b;71:1109–24.

Guo-Qing L, Wilson MVH. An Eocene species of Hiodon from Montana, its phylogenetic relationships, and the evolution of the postcranial skeleton in the Hiodontidae (Teleostei). Journal of Vertebrate Paleontology. 1994;14:153–67.

Hilton EJ. Comparative osteology and phylogenetic systematics of fossil and living bony-tongue fishes (Actinopterygii, Teleostei, Osteoglossomorpha). Zoological Journal of the Linnean Society 2003;137: 1–100

Hutchinson P. Two Triassic fish from South Africa and Australia, with comments on the evolution of the Chondrostei. Palaeontology 1975;18(3): 613-629.

Hutchinson P.The anatomy and phylogenetic position of Helichthys, a redfieldiiform fish from the Triassic of South Africa."Palaeontology 1978;21(4):881-891.

Jarvik E. On the morphology and taxonomy of the middle devonian osteolepid fishes of Scotland. Kungl Sv Vet Akad Handlingar 1948;3,25(1):1-301.

Jerzmanska A. Oligocene Alepocephaloid fishes from the Polish Carpathians. Acta Palaeontologica Polonica 1979;24(1):65-76,

Jiang-Yong Z. New fossil osteoglossomorph from Ningxia, China. Journal of Vertebrate Paleontology. 2004;24:515–24.

Jiang-Yong Z. New fossil osteoglossomorph from Ningxia, China. Journal of Vertebrate Paleontology. 2004;24:515–24.

Jones S. Synopsis of biological data on the long corseletted frigate mackerel Auxis thynnoides Bleeker 1855. FAO Fisheries Biology Synopsis No.71 Species Synopsis No. 28. 1963;782–810.

Khalloufi B, Zaragüeta-Bagils R, Lelièvre H. Rhombichthys intoccabilis, gen. et sp. nov.(Ellimmichthyiformes, Clupeomorpha, Teleostei), from the Cenomanian (Upper Cretaceous) of Ein Yabrud, Middle East: anatomical description and phylogenetic implications. Journal of Vertebrate Paleontology 2010;30(1):57-67.

Konwert M. Orthogonikleithrus francogalliensis, sp. nov. (Teleostei, Orthogonikleithridae) from the Late Jurassic Plattenkalks of Cerin (France). Journal of Vertebrate Paleontology. 2016;36:e1101377.

Kriwet J, Poyato-Ariza FJ. A revision of the pycnodontid fish Coelodus subdiscus Wenz 1989, from the Early Cretaceous of Montsec (Lleida, Spain). Treballs del Museu de Geologia de Barcelona 1999;8:33-65.

Lambers PH. The halecomorph fishes Caturus and Amblysemius in the lithographic limestone of Solnhofen (Tithonian), Bavaria. Geobios. 1994;27:91–9.

Lehman J-P. Etude d'un Pachycormus du Lias de Normandie. Kungl. Svenska Vetenskap.Handlingar 1949;4,1(2):1-44

Lombardo C. Actinopterygians from the middle Triassic of northern Italy and canton Ticino (Switzerland): anatomical déscriptions and nomenclatural problems. Rivista Italiana di Paleontologia e Stratigrafia. 2001;107(3):345-369.

Long JA New palaeoniscoid fishes from the Late Devonian and Early Carboniferous of Victoria. Memoirs of the Association of Australasian Palaeontologists 1988;7:1-64.

Lopez-Arbarello A, Sun ZY, Sferco E. Tintori A, Xu GH, Sun YL, Jiang DY. New species of Sangiorgioichthys Tintori and Lombardo, 2007 (Neopterygii, Semionotiformes) from the Anisian of Luoping (Yunnan Province, South China). Zootaxa 2011;2749:25-39.

Lund R, Poplin C.The Rhadinichthyids (Paleoniscoid Actinopterygians) from the Bear Gulch Limestone of Montana (USA, Lower Carboniferous) Richard Lund and Cécile PoplinSource: Journal of Vertebrate Paleontology.1997;17(3):466-486

McAllister DE.Evolution of branchiostegals and classification of teleostome fishes. National Museum of Canada Biological series 77 1968;221:1-239

Monsch KA. A revision of scombrid fishes (Scombroidei, Perciformes) from the Middle Eocene of Monte Bolca, Italy. Palaeontology 2006;49(4):873-888.

Murray AM, Wilson MVH. A new Palaeocene genus and species of percopsiform (Teleostei: Paracanthopterygii) from the Paskapoo Formation, Smoky Tower, Alberta. Can. J. Earth Sci. 1996;33:429–38.

Nelson GJ. The hyobranchial apparatus of teleostean fishes of the families Engraulidae and Chirocentridae. American Museum novitates 1970;2410:

Olsen PE, McCune AR. Morphology of the Semionotus elegans species group from the Early Jurassic part of the Newark Supergroup of eastern North America with comments on the Family Semionotidae (Neopterygii). Journal of Vertebrate Paleontology. 1991;11:269–92.

Olsen PE. The Skull and Pectoral Girdle of the Parasemionotid Fish Watsonulus eugnathoides from the Early Triassic Sakamena Group of Madagascar, with Comments on the Relationships of the Holostean Fishes. Journal of Vertebrate Paleontology. 1984;4:481–99.

Otero O. Anatomy, systematics and phylogeny of both recent and fossil latid fishes (Teleostei, Perciformes, Latidae). Zoological Journal of the Linnean Society. 2004;141:81–133.

Prokofiev A M. Oligocene eel from the Frauenweiler site (Germany). Journal of Ichthyology2012;52(1):11-18.

Prokofiev AM. A redescription and relationships of the congrid eel Pavelichthys daniltshenkoi (Anguilliformes: Congridae) from the lower Oligocene of Northern Caucasus. Journal of Ichthyology 2007;47(5):335-340.

Raab M, Chalifa Y. A new enchodontid fish genus from the Upper Cenomanian of Jerusalem, Israel. Palaeontology,1987;30(4): 717-731.

Roellig HF. The cranial osteology of Brychaetus muelleri (Pisces Osteoglossidae) Eocene, Isle of Sheppey. Journal of Paleontology. 1974;947–951.

Schaeffer B. Late Triassic fishes from the western United States. Bulletin of the American Museum of Natural History 1967;135:285-342.

Smith GR, Miller RR. Taxonomy of fishes from Miocene Clarkia lake beds, Idaho. Late Cenozoic history of the Pacific Northwest. American Association for the Advancement of Science San Francisco 1985:75-83.

Springer VG. Definition of the suborder Blennioidei and its included families (Pisces: Perciformes). Bulletin of Marine Science 1993;52(1):472-495.

Swartz BA. Devonian actinopterygian phylogeny and evolution based on a redescription of Stegotrachelus finlayi. Zoological Journal of the Linnean Society 2009;156(4):750-784.

Taverne L, Filleul A. Osteology and relationships of the genus Spaniodon (Teleostei, Salmoniformes) from the Santonian (Upper Cretaceous) of Lebanon. Palaeontology, 2003;46(5):927-944.

Tyler JC, Bannikov AF. New specimen of rare fossil triacanthid fish genus Protacanthodes from the Eocene of Monte Bolca, Italy (Triacanthidae, Tetraodontiformes). Studi e Ricerche sui Giacimenti Terziari di Bolca, Museo Civico di Storia Naturale di Verona. 2011;13:29–35.

Tyler JC, Jerzmanska A, Bannikov AF, Swidnicki J. Two new genera and species of Oligocene spikefishes (Tetraodontiformes: Triacanthodidae), the first fossils of the Hollardiinae and Triacanthodinae [Internet]. Smithsonian Institution Press; 1993 [cited 2016 Sep 13].

Tyler JC, Micklich NR. A new genus and species of surgeon fish (Perciformes, Acanthuridae) from the Oligocene of Kanton Glarus, Switzerland. Swiss Journal of Palaeontology 2011;130(2):203-216.

Wen W. Zhang QY, Hu SX, Zhou CY. Xie T. Huang JY. Chen ZQ, Benton MJ. A new basal actinopterygian fish from the Anisian (Middle Triassic) of Luoping, Yunnan Province, Southwest China. Acta Palaeontologica Polonica 2012;57 (1):149–160.

Wilson MV. Osteology of the Palaeocene teleost Esox tiemani. Palaeontology1984;27(3):597-608.

Xu G, Wu F. A deep-bodied ginglymodian fish from the Middle Triassic of eastern Yunnan Province, China, and the phylogeny of lower neopterygians. Chin. Sci. Bull. 2012;57:111–8.

Xu G-H, Chang M-M. Redescription of †Paralycoptera wui Chang & Chou, 1977 (Teleostei: Osteoglossoidei) from the Early Cretaceous of eastern China. Zoological Journal of the Linnean Society. 2009;157:83–106.

Xu G-H, Gao K-Q, Finarelli JA. A revision of the Middle Triassic scanilepiform fish Fukangichthys longidorsalis from Xinjiang, China, with comments on the phylogeny of the Actinopteri. Journal of Vertebrate Paleontology. 2014;34:747–59.
