# Supplementary material for: On trends and patterns in macroevolution: Williston’s law and the branchiostegal series of extant and extinct osteichthyans
Source: BMC Evol Biol. 2019 Jun 10;19:117. doi: 10.1186/s12862-019-1436-x (PMC6558815; doi:10.1186/s12862-019-1436-x)
Supplement: Supplementary file 2 — Data and scripts used in the subsampling analyses. This consists of 4 files (a,b,c,d). Data files include the branchiostegal count and stratigraphic information collected for extant and extinct species (bst_analysis.csv, tabular data), and the time-scaled trees (trees.tre, Newick format) used in the analyses. The 2 R script files also included were used for running the jackniffing analyses that subsample extinct (fossil_subsampling_1.R) or extant species (extant_subsampling_1.R). The “species” column in the CSV file contains the species in the tree to which the data were mapped. The “old_label” column contains the names of the taxa as they appeared in our original sources. An anonymous referee kindly noted that our data had an erroneous value of 1 branchiostegal ray for Lepidosiren paradoxa, which does not have any branchiostegals in reality. We did not rerun our analyses because they are very time-consuming, and this small correction is unlikely to have any significant effect. However, we amended the CVS file that we provide here, so that the error does not propagate to any possible future uses of the data by other researchers. (ZIP 162 kb) [file 12862_2019_1436_MOESM2_ESM.zip › Additional file 2/Additional file 2a trees.docx]

((Guiyu_oneiros:1,(Onychodus_jandemarrai:32.33324654,((Diplocercides:1,(Latimeriidae_Latimeria_chalumnae:329.4898298,Rhabdoderma:1):36.86979371):46.15600352,((Neoceratodontidae_Neoceratodus_forsteri:279.7982796,Lepidosirenidae_Lepidosiren_paradoxa:279.7982796):128.2017204,((Osteolepis_macrolepidotus:1,Gyroptychius_milleri:10.26892947):1,Eusthenopterus_foordi:26.5943684):5.157057854):4.515627):1):12.20779963):17.27657537,((Cheirolepis_trailli:1,Cheirolepis_schultzei:3.949629384):4.833425468,((Polypteridae_Erpetoichthys_calabaricus:29.200001,Polypteridae_Polypterus_senegalus:29.200001):365.800001,(Osorioichthys_marginis:21.9889774,((Tegeolepis_clarki:18.64133568,Howqualepis_rostridens:1):5.245034329,((Gogosardina_coatesi:1,(Mimipiscis_bartrami:6.514790867,Mimipiscis_toombsi:1):1.645701291):6.395739686,((Moythomasia_lineata:1,Moythomasia_durgaringa:5.147218138):2.521423074,(Stegotrachelus_finlayi:1,(Limnomis_delaneyi:1,(Wendyichthys_dicksoni:29.96659292,(Kentuckia_deani:1,((Mesopoma_planti:1.613707556,Mesopoma_carricki:1):7.765595924,((Birgeria_stensioei:1,(Chondrosteus_acipenseroides:1,(Saurichthys_dawaziensis:1,((Protopsephurus_liui:1,Polyodontidae_Polyodon_spathula:128.1121006):10.7879014,(Acipenseridae_Acipenser_fulvescens:38.579335,(Acipenseridae_Scaphirhynchus_platorynchus:19.375515,Acipenseridae_Scaphirhynchus_albus:19.375515):19.20382):100.320667):23.29263209):39.970592):50.57758212):97.3591928,(Boreosomus:75.13376872,(((Fukangichthys_longidorsalis:8.722820679,(Scanilepis_dubia:45.50989011,Evenkia_eunotoptera:1):1):1,Australosomus:2.952988259):70.53448252,(Perleidus_altolepis:85.05925185,(Luganoia_lepidosteoides:88.84510488,(((Macrosemius_fourneti:91.86789771,((Semionotus_elegans:1,Lepisosteidae_Lepisosteus_osseus:201.5919114):43.5009669,Kyphosichthys_grandei:1):1):21.8071227,(Watsonulus_eugnathoides:1,(Amblysemius:1,(Pachyamia_latimaxillaris:2.842402089,(Tomognathus_mordax:1,Amiidae_Amia_calva:99.98243771):1):51.55188613):100.331943):15.03373414):54.6,(Pachycormus:103.5001385,(Crossognathus_danubiensis:179.2902078,(((Anaethalion_angustus:1,Anaethalion_knorri:5.048392857):44.61684283,((Megalopidae_Megalops_atlanticus:133.565966,Elopidae_Elops_saurus:133.565966):62.634035,(Albulidae_Albula_vulpes:150.800001,((Notacanthidae_Notacanthus_chemnitzii:50.669287,(Halosauridae_Halosauropsis_macrochir:40.366211,Halosauridae_Aldrovandia_affinis:40.366211):10.303076):50.330714,(((Eurypharyngidae_Eurypharynx_pelecanoides:25.866364,Saccopharyngidae_Saccopharynx_ampullaceus:25.866364):44.322494,(Nemichthyidae_Nemichthys_scolopaceus:58.739485,Anguillidae_Anguilla_rostrata:58.739485):11.449373):9.198895,(Serrivomeridae_Serrivomer_beanii:76.053487,(Congridae_Conger_oceanicus:65.416147,(Muraenesocidae_Muraenesox_cinereus:57.193624,Ophichthidae_Myrichthys_maculosus:57.193624):8.222523):10.63734):3.334266):21.612248):49.8):45.4):1):86.1,((Lycoptera_davidi:97.58482601,(Paralycoptera_wui:108.3714212,(Xixiaichthys_tongxinens:100.888581,(((Eohiodon_woodruffi:1,Eohiodon_rosei:2.044793584):1,(Hiodon_consteniorum:1,(Hiodontidae_Hiodon_tergisus:9.520291,Hiodontidae_Hiodon_alosoides:9.520291):26.93921607):19.3304413):171.3100526,(Chauliopareion_mahengeense:120.2055177,(Pantodontidae_Pantodon_buchholzi:163.100001,((((Brychaetus_muelleri:1,Phareodus_encaustus:11.86549486):1,Osteoglossidae_Osteoglossum_bicirrhosum:57.92613347):46.92297853,Arapaimidae_Arapaima_gigas:104.849112):43.475578,(Notopteridae_Xenomystus_nigri:120.300002,Gymnarchidae_Gymnarchus_niloticus:120.300002):28.024688):14.775311):1):63):1):1):1):43.7,((((Diplomystus_brevissimus:43.9612409,Sorbinichthys_africanus:1):94.86978024,(Denticipitidae_Denticeps_clupeoides:188.900001,(Chirocentridae_Chirocentrus_dorab:87.096454,((Engraulidae_Coilia_nasus:47.06527,Engraulidae_Engraulis_mordax_eurystole:47.06527):33.347234,((Pristigasteridae_Pellona_flavipinnis:27.450803,Pristigasteridae_Ilisha_elongata:27.450803):46.430359,(Clupeidae_Alosa_pseudoharengus:54.138864,Clupeidae_Dorosoma_cepedianum:54.138864):19.742298):6.531342):6.68395):101.803547):1):40.3,((Alepocephalidae_Talismania_bifurcata:53.266521,((Alepocephalidae_Bathylaco_nigricans:33.802485,Alepocephalidae_Alepocephalus_tenebrosus:33.802485):10.988666,((Alepocephalidae_Rouleina_attrita:22.275085,Alepocephalidae_Xenodermichthys_copei:22.275085):19.924916,Platytroctidae_Sagamichthys_abei:42.200001):2.59115):8.47537):166.430015,((Gonorynchidae_Gonorynchus_abbreviatus:175.900001,(Mahengichthys_singidaensis:104.4607047,Chanidae_Chanos_chanos:147.100001):28.8):22.7,((((Gyrinocheilidae_Gyrinocheilus_sp:70.111694,(Catostomidae_Hypentelium_nigricans:23.594381,(Catostomidae_Carpiodes_carpio:16.355089,Catostomidae_Ictiobus_bubalus:16.355089):7.239292):46.517313):8.688307,Cobitidae_Cobitis_taenia:78.800001):20.5,(Cyprinidae_Danio_rerio:63.300001,(((Cyprinidae_Zacco_sieboldii_platypus:12.727958,Cyprinidae_Opsariichthys_uncirostris_bidens:12.727958):14.610086,((Cyprinidae_Xenocypris_argentea:12.904177,Cyprinidae_Hypophthalmichthys_molitrix:12.904177):2.917503,(Cyprinidae_Luciobrama_macrocephalus:13.439618,(Cyprinidae_Squaliobarbus_curriculus:11.472717,Cyprinidae_Mylopharyngodon_piceus:11.472717):1.966901):2.382062):11.516364):12.401957,(Cyprinidae_Tanakia_lanceolata_himantegus:35.004261,(Cyprinidae_Notemigonus_crysoleucas:22.933404,(Cyprinidae_Semotilus_atromaculatus:21.325215,((Cyprinidae_Campostoma_oligolepis:12.800846,Cyprinidae_Rhinichthys_cataractae:12.800846):4.408707,(Cyprinidae_Phenacobius_uranops:12.822623,(Cyprinidae_Pimephales_promelas_notatus:9.460336,(Cyprinidae_Luxilus_coccogenis:6.738861,Cyprinidae_Notropis_asperifrons:6.738861):2.721475):3.362287):4.38693):4.115662):1.608189):12.070857):4.73574):23.56):36):73.6,((Gymnotidae_Electrophorus_electricus:63.909447,Gymnotidae_Gymnotus_sp:63.909447):83.924067,(((Distichodontidae_Distichodus_maculatus:103.604365,Citharinidae_Citharinus_congicus:103.604365):11.170138,(Alestidae_Alestes_baremoze:106.666979,((Parodontidae_Parodon_nasus:68.800001,Hemiodontidae_Hemiodus_immaculatus:68.800001):22.415197,((Bryconidae_Brycon_pesu:66.003418,(Gasteropelecidae_Thoracocharax_stellatus:27.746828,Gasteropelecidae_Gasteropelecus_sternicla:27.746828):38.25659):1.227196,Characidae_Astyanax_mexicanus:67.230614):23.984584):15.451781):8.107524):22.236489,((Nematogenyidae_Nematogenys_inermis:110.648758,(Trichomycteridae_Trichomycterus_sp:105.886466,((Loricariidae_Loricaria_simillima:70.313425,Astroblepidae_Astroblepus_sp:70.313425):28.122359,(Callichthyidae_Callichthys_callichthys:69.200001,Callichthyidae_Corydoras_trilineatus:69.200001):29.235783):7.450682):4.762292):5.151243,(Diplomystidae_Diplomystes_nahuelbutaensis:106.203801,((Clariidae_Clarias_batrachus:47.60069,Heteropneustidae_Heteropneustes_fossilis:47.60069):38.718856,(((Sisoridae_Bagarius_yarrelli:55.522058,(Amblycipitidae_Liobagrus_aequilabris:48.027606,Akysidae_Akysis_sp:48.027606):7.494452):21.358911,(Schilbeidae_Pseudeutropius_brachypopterus:74.448774,(Bagridae_Mystus_bocourti:36.077943,Bagridae_Bagrus_ubangensis:36.077943):38.370831):2.432195):8.408138,(((Chacidae_Chaca_sp:72.429482,Cetopsidae_Cetopsis_coecutiens:72.429482):10.714797,(Cranoglanididae_Cranoglanis_bouderius:68.700001,Ictaluridae_Ictalurus_punctatus:68.700001):14.444278):1.039619,(Pangasiidae_Pangasianodon_hypophthalmus:82.158061,((Amphiliidae_Amphilius_jacksonii:69.199757,(Malapteruridae_Malapterurus_beninensis:64.740321,Mochokidae_Synodontis_batesii:64.740321):4.459436):10.993535,((Plotosidae_Plotosus_lineatus:73.039645,Siluridae_Hemisilurus_moolenburghi:73.039645):5.503862,(Auchenipteridae_Ageneiosus_atronasus:51.101654,Doradidae_Anduzedoras_oxyrhynchus:51.101654):27.441853):1.649785):1.964769):2.025837):1.105209):1.030439):19.884255):9.5962):21.210991):10.822522):25.066487):25.7):21.096535):10.503465):20.4,(Orthogonikleithrus_francogalliensis:78.90555601,(((Bathylagidae_Bathylagus_euryops:70.542512,(Microstomatidae_Nansenia_longicauda_ardesiaca:51.394,(Opisthoproctidae_Macropinna_microstoma:33.11268,Argentinidae_Argentina_sialis_silus:33.11268):18.28132):19.148512):89.057489,((Galaxiidae_Neochanna_burrowsius:34.505049,Galaxiidae_Galaxias_maculatus:34.505049):111.121934,(((Umbridae_Novumbra_hubbsi:55.762491,Umbridae_Umbra_limi:55.762491):23.63751,(Esocidae_Esox_lucius:31.366195,Esocidae_Esox_americanus:31.366195):48.033806):25.064928,(Salmonidae_Coregonus_clupeaformis:35.300001,(Salmonidae_Thymallus_brevirostris:32.456522,(Salmonidae_Oncorhynchus_nerka_mykiss:19.929533,(Salmonidae_Salvelinus_alpinus:16.737138,Salmonidae_Salmo_salar:16.737138):3.192395):12.526989):2.843479):69.164928):41.162054):13.973018):55.253787,(((Retropinnidae_Retropinna_semoni:73.700001,(((Osmeridae_Osmerus_mordax:10.332899,Osmeridae_Thaleichthys_pacificus:10.332899):5.872101,(Osmeridae_Mallotus_villosus:14.961758,Osmeridae_Hypomesus_pretiosus:14.961758):1.243242):18.133836,(Plecoglossidae_Plecoglossus_altivelis:28.70318,Salangidae_Neosalangichthys_ishikawae:28.70318):5.635656):39.361165):55.7,((Diplophidae_Diplophos_taenia:77.330737,((Gonostomatidae_Bonapartia_pedaliota:54.297445,Gonostomatidae_Margrethia_obtusirostra:54.297445):20.014374,(Gonostomatidae_Gonostoma_elongatum:69.948163,Gonostomatidae_Cyclothone_microdon:69.948163):4.363656):3.018918):6.469264,(Phosichthyidae_Pollichthys_mauli:77.722827,((Sternoptychidae_Argyropelecus_gigas:25.230481,Sternoptychidae_Maurolicus_weitzmani:25.230481):47.329876,(Stomiidae_Chauliodus_macouni_danae:63.126299,(Stomiidae_Stomias_boa:36.381906,(Stomiidae_Chirostomias_pliopterus:31.578039,((Stomiidae_Melanostomias_margaritifer:21.790927,Stomiidae_Leptostomias_longibarba:21.790927):6.240428,((Stomiidae_Photonectes_margarita:19.053467,Stomiidae_Tactostoma_macropus:19.053467):7.977888,(Stomiidae_Malacosteus_niger:25.031355,(Stomiidae_Eustomias_polyaster:24.031355,(Stomiidae_Aristostomias_scintillans:23.031355,Stomiidae_Bathophilus_flemingi_pawneei:23.031355):1):1):2):1):3.546684):4.803867):26.744393):9.434058):5.16247):6.077174):45.6):81.9,((Ateleopodidae_Ateleopus_japonicus:8.089622,Ateleopodidae_Ijimaia_antillarum:8.089622):184.210379,(((Synodontidae_Trachinocephalus_myops:34.928634,Synodontidae_Synodus_foetens:34.928634):78.971367,((Paraulopidae_Paraulopus_oblongus:101.985288,(Synodontidae_Saurida_gracilis:50.257509,Synodontidae_Harpadon_microchir:50.257509):51.727779):8.326796,(((Aulopidae_Aulopus_filamentosus:47.160387,Bathysauridae_Bathysaurus_ferox:47.160387):10.843902,(Ipnopidae_Ipnops_murrayi:13.82,Ipnopidae_Ipnops_agassizi:13.82):44.184289):23.635948,((Ipnopidae_Bathypterois_atricolor:69.328167,(Giganturidae_Gigantura_indica:13.525058,Giganturidae_Gigantura_chuni:13.525058):55.803109):6.462647,(Scopelarchidae_Benthalbella_macropinna:62.490696,(Chlorophthalmidae_Chlorophthalmus_agassizi:61.13635,(Sudidae_Sudis_atrox:55.026295,((Alepisauridae_Anotopterus_pharao:45.661943,(Alepisauridae_Omosudis_lowii:36.284216,Alepisauridae_Alepisaurus_ferox:36.284216):9.377727):8.238175,(Paralepididae_Stemonosudis_intermedia_macrura:50.122687,(Evermannellidae_Evermannella_balbo:46.686813,Paralepididae_Paralepis_coregonoides:46.686813):3.435874):3.777431):1.126177):6.110055):1.354346):13.300118):5.849423):28.671847):3.587917):68.9,(((Neoscopelidae_Neoscopelus_macrolepidotus:42.408365,Neoscopelidae_Scopelengys_tristis:42.408365):31.191636,((Myctophidae_Lampadena_speculigera:39.81673,Myctophidae_Lampanyctus_macdonaldi:39.81673):11.872,(Myctophidae_Benthosema_glaciale:28.30241,Myctophidae_Myctophum_punctatum:28.30241):23.38632):21.911271):99.5,(((Lamprididae_Lampris_guttatus:65.552505,(Regalecidae_Regalecus_russelii:37.682943,Trachipteridae_Trachipterus_trachypterus:37.682943):27.869562):84.747496,(((Mcconichthys_longipinnis:1,(Aphredoderidae_Aphredoderus_sayanus:41.500001,Amblyopsidae_Chologaster_cornuta:41.500001):21.47414738):1,(Lateopisciculus_turrifumosus:1,(Percopsidae_Percopsis_omiscomaycus:14.617149,Percopsidae_Percopsis_transmontana:14.617149):43.95560305):5.401396329):71.01618362,(Zeidae_Zeus_faber:107.100001,(Stylephoridae_Stylephorus_chordatus:78.800001,(Merlucciidae_Merluccius_productus:45.655086,(((Macrouridae_Gadomus_dispar:9.947595,Macrouridae_Bathygadus_favosus:9.947595):28.939269,(Steindachneriidae_Steindachneria_argentea:35.929071,((Macrouridae_Malacocephalus_laevis:17.761718,Macrouridae_Trachonurus_sulcatus:17.761718):10.126413,(Macrouridae_Coelorinchus_caribbaeus:21.883517,Macrouridae_Coryphaenoides_armatus:21.883517):6.004614):8.04094):2.957793):2.613137,((Moridae_Laemonema_goodebeanorum:25.08833,(Moridae_Halargyreus_johnsonii:16.444614,(Moridae_Lepidion_ensiferus:5.35693,Moridae_Antimora_rostrata:5.35693):11.087684):8.643716):13.1716,((Phycidae_Urophycis_tenuis:5.240351,Phycidae_Urophycis_chuss:5.240351):28.607969,(Lotidae_Gaidropsarus_ensis:29.800286,(Lotidae_Lota_lota:23.935071,(Gadidae_Melanogrammus_aeglefinus:5.86839,Gadidae_Gadus_morhua:5.86839):18.066681):5.865215):4.048034):4.41161):3.240071):4.155085):33.144915):28.3):27.890331):15.309669):11.08283,(Polymixiidae_Polymixia_japonica:154.800001,(((Diretmidae_Diretmus_argenteus:53.99855,(Monocentridae_Monocentris_japonica:33.600001,((Trachichthyidae_Hoplostethus_occidentalis_atlanticus:24.361165,Trachichthyidae_Gephyroberyx_darwinii:24.361165):7.919156,(Anoplogastridae_Anoplogaster_cornuta:31.22776,Trachichthyidae_Paratrachichthys_sajademalensis:31.22776):1.052561):1.31968):20.398549):71.275924,((Berycidae_Beryx_decadactylus:70.612394,(Melamphaidae_Poromitra_crassiceps:39.138751,(Melamphaidae_Scopelogadus_beanii:32.603956,Melamphaidae_Melamphaes_suborbitalis:32.603956):6.534795):31.473643):40.295775,(((Rondeletiidae_Rondeletia_bicolor:18.616456,Rondeletiidae_Rondeletia_loricata:18.616456):36.662305,Barbourisiidae_Barbourisia_rufa:55.278761):34.605685,(Cetomimidae_Cetostoma_regani:55.492218,(Cetomimidae_Cetomimus_craneae:10.537863,Cetomimidae_Cetomimus_compuctus:10.537863):44.954355):34.392228):21.023723):14.366305):21.189147,((Holocentridae_Sargocentron_diadema:25.43456,Holocentridae_Holocentrus_rufus:25.43456):119.565441,((Ophidiidae_Brotula_multibarbata:66.227334,(Ophidiidae_Lepophidium_profundorum:23.036156,(Ophidiidae_Genypterus_blacodes:21.590629,Ophidiidae_Ophidion_holbrookii:21.590629):1.445527):43.191178):66.572667,((Batrachoididae_Porichthys_notatus:39.800001,Batrachoididae_Opsanus_tau:39.800001):87,(((Kurtidae_Kurtus_indicus:80.300001,Apogonidae_Apogon_campbelli:80.300001):22,(Eleotridae_Eleotris_acanthopoma_pisonis:64.430477,(Gobiidae_Gobiosoma_bosc:45.630567,Microdesmidae_Microdesmus_longipinnis:45.630567):18.79991):37.869524):19.3,((((Dactylopteridae_Dactylopterus_volitans:68.121568,(Aulostomidae_Aulostomus_maculatus:3.200001,Aulostomidae_Aulostomus_chinensis:3.200001):64.921567):6.178433,((Fistulariidae_Fistularia_petimba:65.463647,Mullidae_Mullus_auratus:65.463647):6.26906,(Callionymidae_Callionymus_sp_bairdi:69.154942,Syngnathidae_Syngnathus_fuscus:69.154942):2.577765):2.567294):20.27,(Chiasmodontidae_Chiasmodon_sp:43.900001,((Centrolophidae_Icichthys_lockingtoni:34.370774,Pomatomidae_Pomatomus_saltatrix:34.370774):6.934458,(((Ariommatidae_Ariomma_bondi:25.644121,Nomeidae_Psenes_cyanophrys:25.644121):5.370208,Scombridae_Auxis_rochei:31.014329):6.588438,((Stromateidae_Peprilus_paru:35.602767,(Gempylidae_Paradiplospinus_gracilis:31.499638,Bramidae_Brama_brama:31.499638):4.103129):1,(Icosteidae_Icosteus_aenigmaticus:33.972252,Trichiuridae_Trichiurus_lepturus:33.972252):2.630515):1):3.702465):2.594769):50.67):21.916687,((((((Synbranchidae_Monopterus_albus:69.146846,Indostomidae_Indostomus_paradoxus:69.146846):4.548479,(Mastacembelidae_Macrognathus_siamensis:18.057555,Mastacembelidae_Mastacembelus_erythrotaenia:18.057555):55.63777):7.004676,((Anabantidae_Ctenopoma_acutirostre_kingsleyae:62.782323,(Helostomatidae_Helostoma_temminkii:59.687826,Osphronemidae_Trichopodus_pectoralis:59.687826):3.094497):7.86905,(Channidae_Channa_striata:67.428514,Nandidae_Nandus_nandus:67.428514):3.222859):10.048628):15.7,((Nematistiidae_Nematistius_pectoralis:60.067614,((Coryphaenidae_Coryphaena_hippurus:19.124214,Rachycentridae_Rachycentron_canadum:19.124214):12.775787,(Echeneidae_Remora_osteochir_australis:10.178122,Echeneidae_Echeneis_naucrates:10.178122):21.721879):28.167613):9.732387,((Sphyraenidae_Sphyraena_sphyraena:65.900885,(Menidae_Mene_maculata:60.339531,Polynemidae_Polydactylus_octonemus:60.339531):5.561354):2.899116,((((Leptobramidae_Leptobrama_muelleri:46.58559,Toxotidae_Toxotes_jaculatrix:46.58559):5.096327,(Xiphiidae_Xiphias_gladius:27.833842,Istiophoridae_Istiophorus_platypterus:27.833842):23.848075):5.298938,Carangidae_Trachinotus_falcatus:56.980855):4.861848,((Eolates_gracilis:1,(Centropomidae_Psammoperca_waigiensis:33.068144,(Centropomidae_Lates_calcarifer:13.595548,Centropomidae_Lates_microlepis:13.595548):19.472596):21.75434953):6.020209469,(Heteronectes_chaneti:6.806819153,(Amphistium_paradoxum:3.244534071,(Psettodidae_Psettodes_erumei:57.842703,(Citharidae_Lepidoblepharon_ophthalmolepis:53.600001,((Cynoglossidae_Cynoglossus_interruptus:42.400002,(Soleidae_Solea_solea:24.944331,Soleidae_Soleichthys_heterorhinos:24.944331):17.455671):9.674607,((Scophthalmidae_Scophthalmus_aquosus:23.305906,Scophthalmidae_Lepidorhombus_boscii:23.305906):26.973285,((Paralichthyidae_Paralichthys_dentatus:40.749719,Bothidae_Bothus_lunatus:40.749719):4.387561,(Paralichthyidae_Pseudorhombus_pentophthalmus:21.404631,(((Pleuronectidae_Glyptocephalus_zachirus:5.096079,Pleuronectidae_Microstomus_pacificus:5.096079):2.538752,(Pleuronectidae_Lyopsetta_exilis:6.085211,Pleuronectidae_Hippoglossus_hippoglossus:6.085211):1.54962):2.215451,(Pleuronectidae_Limanda_limanda:8.798272,((Pleuronectidae_Platichthys_stellatus:3.717184,Pleuronectidae_Pleuronectes_platessa:3.717184):3.081088,(Pleuronectidae_Lepidopsetta_bilineata:4.798272,Pleuronectidae_Psettichthys_melanostictus:4.798272):2):2):1.05201):11.554349):23.732649):5.141911):1.795418):1.525392):4.242702):1):1):1):1):6.957298):1):26.6):12.043384,(((Polycentridae_Polycentrus_schomburgkii:94.214904,(Pholidichthyidae_Pholidichthys_leucotaenia:88.700001,Cichlidae_Cichla_temensis:88.700001):5.514903):1,(((Atherinopsidae_Atherinopsis_californiensis:48.715388,Atherinopsidae_Menidia_menidia:48.715388):22.225577,(Isonidae_Iso_sp:49.317292,(Atherinidae_Atherinomorus_stipes:43.78646,(Melanotaeniidae_Melanotaenia_splendida:39.870052,Pseudomugilidae_Pseudomugil_gertrudae:39.870052):3.916408):5.530832):21.623673):6.46052,(((Poeciliidae_Poecilia_latipinna_reticulata:14.292152,(Poeciliidae_Heterandria_formosa:11.366823,(Poeciliidae_Gambusia_affinis:5.736451,Poeciliidae_Belonesox_belizanus:5.736451):5.630372):2.925329):30.13151,(Cyprinodontidae_Cyprinodon_variegatus:40.469994,Fundulidae_Fundulus_parvipinnis:40.469994):3.953668):31.977823,(Adrianichthyidae_Oryzias_latipes:71.878571,((Zenarchopteridae_Dermogenys_collettei:29.883878,((Belonidae_Tylosurus_crocodilus:6.568056,Belonidae_Ablennes_hians:6.568056):17.429484,(Scomberesocidae_Cololabis_saira:2.646712,Scomberesocidae_Scomberesox_saurus:2.646712):21.350828):5.886338):17.178962,(Hemiramphidae_Hyporhamphus_affinis:36.892311,((Hemiramphidae_Hemiramphus_brasiliensis:13.391766,Hemiramphidae_Oxyporhamphus_micropterus:13.391766):10.259915,((Exocoetidae_Cypselurus_callopterus:6.413654,Exocoetidae_Exocoetus_monocirrhus:6.413654):4.605967,Exocoetidae_Cheilopogon_pinnatibarbatus:11.019621):12.63206):13.24063):10.170529):24.815731):4.522914):1):17.813419):1.999954,((Pseudochromidae_Halidesmus_scapularis:84.850287,Pomacentridae_Pomacentrus_brachialis:84.850287):8.187103,(Embiotocidae_Cymatogaster_aggregata:91.529056,(Mugilidae_Mugil_cephalus:89.757655,(Plesiopidae_Plesiops_coeruleolineatus:86.977996,(Opistognathidae_Opistognathus_maxillosus:79.196597,(((Gobiesocidae_Lepadichthys_lineatus:37.560081,Gobiesocidae_Gobiesox_maeandricus:37.560081):35.985217,Tripterygiidae_Enneanectes_altivelis:73.545298):3.122761,((Clinidae_Clinus_cottoides:50.741024,(Dactyloscopidae_Platygillellus_rubrocinctus:45.641062,Chaenopsidae_Chaenopsis_alepidota:45.641062):5.099962):17.520576,(Blenniidae_Stanulus_seychellensis:31.042358,Blenniidae_Blenniella_cyanostigma:31.042358):37.219242):8.406459):2.528538):7.781399):2.779659):1.771401):1.508334):4.177468):11.228527):3.450865,(Gerreidae_Eucinostomus_argenteus:108.000002,(((Labridae_Lachnolaimus_maximus:59.822938,Odacidae_Haletta_semifasciata:59.822938):16.826114,Scaridae_Scarus_globiceps:76.649052):30.35095,(((Uranoscopidae_Uranoscopus_sulphureus:83.996275,(Ammodytidae_Ammodytes_hexapterus:78.584442,Pinguipedidae_Parapercis_punctulata:78.584442):5.411833):11.903726,(((Acropomatidae_Acropoma_japonicum:90.360051,((Percophidae_Acanthaphritis_unoorum:66.16617,Creediidae_Limnichthys_sp:66.16617):18.796708,(Glaucosomatidae_Glaucosoma_buergeri:66.617288,Pempheridae_Pempheris_schomburgkii:66.617288):18.34559):5.397173):2.814411,(((Oplegnathidae_Oplegnathus_punctatus:61.206964,Kuhliidae_Kuhlia_rupestris:61.206964):7.298332,Kyphosidae_Kyphosus_sectatrix:68.505296):16.157201,(Percichthyidae_Percichthys_trucha:69.999181,((Cirrhitidae_Cirrhitichthys_falco:66.467051,Cheilodactylidae_Cheilodactylus_fasciatus:66.467051):2.53213,(Enoplosidae_Enoplosus_armatus:63.04721,Centrarchidae_Acantharchus_pomotis:63.04721):5.951971):1):14.663316):8.511965):1.725539,(Serranidae_Pseudogramma_polyacantha:82.200001,(Percidae_Perca_fluviatilis:78.577195,((Bovichtidae_Bovichtus_diacanthus:63.021097,(Nototheniidae_Notothenia_coriiceps:9.671082,(Harpagiferidae_Harpagifer_antarcticus:8.390321,(Bathydraconidae_Gymnodraco_acuticeps:7.390321,Channichthyidae_Chionodraco_rastrospinosus:7.390321):1):1.280761):53.350015):12.936917,(((Peristediidae_Peristedion_ecuadorense:54.532565,Triglidae_Prionotus_carolinus:54.532565):11.953546,(Synanceiidae_Synanceia_verrucosa:62.510934,Scorpaenidae_Scorpaenodes_guamensis:62.510934):3.975177):6.621302,(Platycephalidae_Platycephalus_indicus:70.795272,(Anoplopomatidae_Anoplopoma_fimbria:46.504594,((Bathymasteridae_Rathbunella_hypoplecta:22.798552,(Stichaeidae_Cebidichthys_violaceus:15.58542,(Zoarcidae_Zoarces_americanus_viviparus:12.493576,(Anarhichadidae_Anarrhichthys_ocellatus:9.933504,(Zaproridae_Zaprora_silenus:8.933504,Cryptacanthodidae_Cryptacanthodes_maculatus:8.933504):1):2.560072):3.091844):7.213132):19.028758,(((Aulorhynchidae_Aulorhynchus_flavidus:25.280385,Hypoptychidae_Hypoptychus_dybowskii:25.280385):1.019618,((Gasterosteidae_Culaea_inconstans:19.844899,(Gasterosteidae_Apeltes_quadracus:17.136644,Gasterosteidae_Spinachia_spinachia:17.136644):2.708255):1.782129,(Gasterosteidae_Pungitius_pungitius:16.827665,Gasterosteidae_Gasterosteus_aculeatus:16.827665):4.799363):4.672975):13.53479,(Hexagrammidae_Hexagrammos_decagrammus:27.818969,((Cyclopteridae_Cyclopterus_lumpus:20.762394,Liparidae_Liparis_pulchellus:20.762394):4.037609,(Agonidae_Hypsagonus_quadricornis:20.371382,(Cottidae_Cottus_carolinae:18.19359,Psychrolutidae_Psychrolutes_phrictus:18.19359):2.177792):4.428621):3.018966):12.015824):1.992517):4.677284):24.290678):2.312141):2.850601):2.619181):3.622806):12.7):1):10.100001,((Drepaneidae_Drepane_punctata:67.658011,Ephippidae_Chaetodipterus_faber:67.658011):37.341991,(((Lobotidae_Lobotes_pacificus_surinamensis:86.438004,Sciaenidae_Menticirrhus_undulatus_littoralis:86.438004):5.761997,((Monodactylidae_Monodactylus_sebae:70.333247,(Avitoluvarus_eocaenicus:20.50205407,(Zanclidae_Zanclus_cornutus:55.859022,Acanthuridae_Acanthurus_triostegus:55.859022):4.740979):9.733246):15.366754,((Pomacanthidae_Pomacanthus_semicirculatus:54.434118,(Leiognathidae_Leiognathus_equulus:32.700001,Chaetodontidae_Chelmon_rostratus:32.700001):21.734117):17.438962,(Emmelichthyidae_Erythrocles_schlegelii:69.871718,(Malacanthidae_Malacanthus_plumieri:68.303733,(Haemulidae_Haemulon_aurolineatum:64.600001,Lutjanidae_Lutjanus_griseus:64.600001):3.703732):1.567985):2.001362):13.826921):6.5):11.800001,((Sillaginidae_Sillago_sihama:85.853361,(Nemipteridae_Pentapodus_caninus:80.270573,(Lethrinidae_Lethrinus_erythropterus:74.370539,Sparidae_Stenotomus_chrysops:74.370539):5.900034):5.582788):17.146641,(Siganidae_Siganus_spinus:102.000002,((Scatophagidae_Scatophagus_argus:75.860371,Priacanthidae_Heteropriacanthus_cruentatus:75.860371):25.139631,((Caproidae_Antigonia_rubescens:10.905674,Caproidae_Antigonia_capros:10.905674):89.094328,(((Lophiidae_Lophiodes_reticulatus:22.462185,Lophiidae_Lophius_gastrophysus:22.462185):42.032119,((Antennariidae_Histrio_histrio:12.333102,Antennariidae_Antennatus_coccineus:12.333102):33.466899,(Chaunacidae_Chaunax_suttkusi:38.499563,(Gigantactinidae_Gigantactis_sp:32.911618,((Ceratiidae_Cryptopsaras_couesii:20.548708,Ceratiidae_Ceratias_holboelli:20.548708):10.416157,((Himantolophidae_Himantolophus_albinares_sagamius:14.456802,Melanocetidae_Melanocetus_murrayi:14.456802):9.124159,(Oneirodidae_Oneirodes_macrosteus:10.164357,Oneirodidae_Dolopichthys_sp:10.164357):13.416604):7.383904):1.946753):5.587945):7.300438):18.694303):34.505698,(((Prohollardia_avita:40.3444058,(Protacanthodes_nimesensis:1,Triacanthidae_Triacanthus_biaculeatus:44.14074597):20.35222503):10.123893,(Aracanidae_Aracana_aurita:54.800001,(Ostraciidae_Ostracion_cubicus:51.000002,Ostraciidae_Rhinesomus_triqueter:51.000002):3.799999):19.816863):3.183137,(((Diodontidae_Diodon_holocanthus:12.198387,Diodontidae_Chilomycterus_schoepfii:12.198387):43.701614,(Tetraodontidae_Canthigaster_bennetti:24.22571,Tetraodontidae_Tetraodon_miurus:24.22571):31.674291):17.334859,((Molidae_Ranzania_laevis:22,Molidae_Mola_mola:22):46.61027,((Monacanthidae_Aluterus_scriptus:27.823604,Monacanthidae_Stephanolepis_hispidus:27.823604):15.976396,(Balistidae_Xanthichthys_ringens:20.7,(Balistidae_Balistes_vetula:19.7,Balistidae_Sufflamen_fraenatum:19.7):1):23.1):24.81027):4.62459):4.565141):21.200001):1):1):1):1):1):1):1):1):1):3.894248):4.592438):5.113313):5.2):6):12.2):1.46362):8.33638):6.58283):11.71717):9.7):9.5):19):3.553787):17.946213):17.8):23.2):9.5):1):1):37.2):1):1):1):1):23.6):1):8.033451918):1):10.830758):16.23959447):1):1):1):1):3.796196615):1):47);

((Guiyu_oneiros:1,(Onychodus_jandemarrai:32.32661556,((Diplocercides:1,(Latimeriidae_Latimeria_chalumnae:328.175614,Rhabdoderma:1):38.80363664):45.53637632,((Neoceratodontidae_Neoceratodus_forsteri:279.7982796,Lepidosirenidae_Lepidosiren_paradoxa:279.7982796):129.3376531,((Osteolepis_macrolepidotus:1,Gyroptychius_milleri:15.85242978):1,Eusthenopterus_foordi:27.51527804):1):3.379694327):1):12.98353658):16.50083842,((Cheirolepis_trailli:1,Cheirolepis_schultzei:6.365677784):3.830395377,((Polypteridae_Erpetoichthys_calabaricus:29.200001,Polypteridae_Polypterus_senegalus:29.200001):365.800001,(Osorioichthys_marginis:19.73170253,((Tegeolepis_clarki:17.47052125,Howqualepis_rostridens:1):1,((Gogosardina_coatesi:2.408515773,(Mimipiscis_bartrami:2.023942171,Mimipiscis_toombsi:1):1):5.199737475,((Moythomasia_lineata:1,Moythomasia_durgaringa:6.607113765):1.390889002,(Stegotrachelus_finlayi:1,(Limnomis_delaneyi:1,(Wendyichthys_dicksoni:30.89073828,(Kentuckia_deani:1,((Mesopoma_planti:1,Mesopoma_carricki:5.928874389):3.959645396,((Birgeria_stensioei:1,(Chondrosteus_acipenseroides:1,(Saurichthys_dawaziensis:1,((Protopsephurus_liui:1,Polyodontidae_Polyodon_spathula:128.6472799):10.25272209,(Acipenseridae_Acipenser_fulvescens:38.579335,(Acipenseridae_Scaphirhynchus_platorynchus:19.375515,Acipenseridae_Scaphirhynchus_albus:19.375515):19.20382):100.320667):23.85330809):38.7048497):51.34066202):97.30117919,(Boreosomus:74.34354176,(((Fukangichthys_longidorsalis:9.505150962,(Scanilepis_dubia:44.56786075,Evenkia_eunotoptera:1):1):1,Australosomus:2.692648323):70.67979083,(Perleidus_altolepis:88.85209666,(Luganoia_lepidosteoides:90.13021611,(((Macrosemius_fourneti:91.0609925,((Semionotus_elegans:1,Lepisosteidae_Lepisosteus_osseus:201.5165843):45.27638282,Kyphosichthys_grandei:1):1):20.10703386,(Watsonulus_eugnathoides:1,(Amblysemius:1,(Pachyamia_latimaxillaris:1.623529564,(Tomognathus_mordax:1,Amiidae_Amia_calva:98.53611656):1):52.00092947):101.6281394):14.73481561):54.6,(Pachycormus:106.2930851,(Crossognathus_danubiensis:177.4031209,(((Anaethalion_angustus:1.988016107,Anaethalion_knorri:1):44.58176507,((Megalopidae_Megalops_atlanticus:133.565966,Elopidae_Elops_saurus:133.565966):62.634035,(Albulidae_Albula_vulpes:150.800001,((Notacanthidae_Notacanthus_chemnitzii:50.669287,(Halosauridae_Halosauropsis_macrochir:40.366211,Halosauridae_Aldrovandia_affinis:40.366211):10.303076):50.330714,(((Eurypharyngidae_Eurypharynx_pelecanoides:25.866364,Saccopharyngidae_Saccopharynx_ampullaceus:25.866364):44.322494,(Nemichthyidae_Nemichthys_scolopaceus:58.739485,Anguillidae_Anguilla_rostrata:58.739485):11.449373):9.198895,(Serrivomeridae_Serrivomer_beanii:76.053487,(Congridae_Conger_oceanicus:65.416147,(Muraenesocidae_Muraenesox_cinereus:57.193624,Ophichthidae_Myrichthys_maculosus:57.193624):8.222523):10.63734):3.334266):21.612248):49.8):45.4):1):86.1,((Lycoptera_davidi:98.74287055,(Paralycoptera_wui:108.0061912,(Xixiaichthys_tongxinens:102.3138484,(((Eohiodon_woodruffi:1,Eohiodon_rosei:1.779957311):1,(Hiodon_consteniorum:1,(Hiodontidae_Hiodon_tergisus:9.520291,Hiodontidae_Hiodon_alosoides:9.520291):28.77511377):17.90653458):170.8980616,(Chauliopareion_mahengeense:118.742584,(Pantodontidae_Pantodon_buchholzi:163.100001,(((Osteoglossidae_Osteoglossum_bicirrhosum:51.43195837,(Brychaetus_muelleri:1,Phareodus_encaustus:2.756981622):1):53.41715363,Arapaimidae_Arapaima_gigas:104.849112):43.475578,(Notopteridae_Xenomystus_nigri:120.300002,Gymnarchidae_Gymnarchus_niloticus:120.300002):28.024688):14.775311):1):63):1):1):1):43.7,((((Diplomystus_brevissimus:50.49243436,Sorbinichthys_africanus:1):89.12680064,(Denticipitidae_Denticeps_clupeoides:188.900001,(Chirocentridae_Chirocentrus_dorab:87.096454,((Engraulidae_Coilia_nasus:47.06527,Engraulidae_Engraulis_mordax_eurystole:47.06527):33.347234,((Pristigasteridae_Pellona_flavipinnis:27.450803,Pristigasteridae_Ilisha_elongata:27.450803):46.430359,(Clupeidae_Alosa_pseudoharengus:54.138864,Clupeidae_Dorosoma_cepedianum:54.138864):19.742298):6.531342):6.68395):101.803547):1):40.3,((Alepocephalidae_Talismania_bifurcata:53.266521,((Alepocephalidae_Bathylaco_nigricans:33.802485,Alepocephalidae_Alepocephalus_tenebrosus:33.802485):10.988666,((Alepocephalidae_Rouleina_attrita:22.275085,Alepocephalidae_Xenodermichthys_copei:22.275085):19.924916,Platytroctidae_Sagamichthys_abei:42.200001):2.59115):8.47537):166.430015,((Gonorynchidae_Gonorynchus_abbreviatus:175.900001,(Mahengichthys_singidaensis:99.59961258,Chanidae_Chanos_chanos:147.100001):28.8):22.7,((((Gyrinocheilidae_Gyrinocheilus_sp:70.111694,(Catostomidae_Hypentelium_nigricans:23.594381,(Catostomidae_Carpiodes_carpio:16.355089,Catostomidae_Ictiobus_bubalus:16.355089):7.239292):46.517313):8.688307,Cobitidae_Cobitis_taenia:78.800001):20.5,(Cyprinidae_Danio_rerio:63.300001,(((Cyprinidae_Zacco_sieboldii_platypus:12.727958,Cyprinidae_Opsariichthys_uncirostris_bidens:12.727958):14.610086,((Cyprinidae_Xenocypris_argentea:12.904177,Cyprinidae_Hypophthalmichthys_molitrix:12.904177):2.917503,(Cyprinidae_Luciobrama_macrocephalus:13.439618,(Cyprinidae_Squaliobarbus_curriculus:11.472717,Cyprinidae_Mylopharyngodon_piceus:11.472717):1.966901):2.382062):11.516364):12.401957,(Cyprinidae_Tanakia_lanceolata_himantegus:35.004261,(Cyprinidae_Notemigonus_crysoleucas:22.933404,(Cyprinidae_Semotilus_atromaculatus:21.325215,((Cyprinidae_Campostoma_oligolepis:12.800846,Cyprinidae_Rhinichthys_cataractae:12.800846):4.408707,(Cyprinidae_Phenacobius_uranops:12.822623,(Cyprinidae_Pimephales_promelas_notatus:9.460336,(Cyprinidae_Luxilus_coccogenis:6.738861,Cyprinidae_Notropis_asperifrons:6.738861):2.721475):3.362287):4.38693):4.115662):1.608189):12.070857):4.73574):23.56):36):73.6,((Gymnotidae_Electrophorus_electricus:63.909447,Gymnotidae_Gymnotus_sp:63.909447):83.924067,(((Distichodontidae_Distichodus_maculatus:103.604365,Citharinidae_Citharinus_congicus:103.604365):11.170138,(Alestidae_Alestes_baremoze:106.666979,((Parodontidae_Parodon_nasus:68.800001,Hemiodontidae_Hemiodus_immaculatus:68.800001):22.415197,((Bryconidae_Brycon_pesu:66.003418,(Gasteropelecidae_Thoracocharax_stellatus:27.746828,Gasteropelecidae_Gasteropelecus_sternicla:27.746828):38.25659):1.227196,Characidae_Astyanax_mexicanus:67.230614):23.984584):15.451781):8.107524):22.236489,((Nematogenyidae_Nematogenys_inermis:110.648758,(Trichomycteridae_Trichomycterus_sp:105.886466,((Loricariidae_Loricaria_simillima:70.313425,Astroblepidae_Astroblepus_sp:70.313425):28.122359,(Callichthyidae_Callichthys_callichthys:69.200001,Callichthyidae_Corydoras_trilineatus:69.200001):29.235783):7.450682):4.762292):5.151243,(Diplomystidae_Diplomystes_nahuelbutaensis:106.203801,((Clariidae_Clarias_batrachus:47.60069,Heteropneustidae_Heteropneustes_fossilis:47.60069):38.718856,(((Sisoridae_Bagarius_yarrelli:55.522058,(Amblycipitidae_Liobagrus_aequilabris:48.027606,Akysidae_Akysis_sp:48.027606):7.494452):21.358911,(Schilbeidae_Pseudeutropius_brachypopterus:74.448774,(Bagridae_Mystus_bocourti:36.077943,Bagridae_Bagrus_ubangensis:36.077943):38.370831):2.432195):8.408138,(((Chacidae_Chaca_sp:72.429482,Cetopsidae_Cetopsis_coecutiens:72.429482):10.714797,(Cranoglanididae_Cranoglanis_bouderius:68.700001,Ictaluridae_Ictalurus_punctatus:68.700001):14.444278):1.039619,(Pangasiidae_Pangasianodon_hypophthalmus:82.158061,((Amphiliidae_Amphilius_jacksonii:69.199757,(Malapteruridae_Malapterurus_beninensis:64.740321,Mochokidae_Synodontis_batesii:64.740321):4.459436):10.993535,((Plotosidae_Plotosus_lineatus:73.039645,Siluridae_Hemisilurus_moolenburghi:73.039645):5.503862,(Auchenipteridae_Ageneiosus_atronasus:51.101654,Doradidae_Anduzedoras_oxyrhynchus:51.101654):27.441853):1.649785):1.964769):2.025837):1.105209):1.030439):19.884255):9.5962):21.210991):10.822522):25.066487):25.7):21.096535):10.503465):20.4,(Orthogonikleithrus_francogalliensis:78.61371364,(((Bathylagidae_Bathylagus_euryops:70.542512,(Microstomatidae_Nansenia_longicauda_ardesiaca:51.394,(Opisthoproctidae_Macropinna_microstoma:33.11268,Argentinidae_Argentina_sialis_silus:33.11268):18.28132):19.148512):89.057489,((Galaxiidae_Neochanna_burrowsius:34.505049,Galaxiidae_Galaxias_maculatus:34.505049):111.121934,(((Umbridae_Novumbra_hubbsi:55.762491,Umbridae_Umbra_limi:55.762491):23.63751,(Esocidae_Esox_lucius:31.366195,Esocidae_Esox_americanus:31.366195):48.033806):25.064928,(Salmonidae_Coregonus_clupeaformis:35.300001,(Salmonidae_Thymallus_brevirostris:32.456522,(Salmonidae_Oncorhynchus_nerka_mykiss:19.929533,(Salmonidae_Salvelinus_alpinus:16.737138,Salmonidae_Salmo_salar:16.737138):3.192395):12.526989):2.843479):69.164928):41.162054):13.973018):55.253787,(((Retropinnidae_Retropinna_semoni:73.700001,(((Osmeridae_Osmerus_mordax:10.332899,Osmeridae_Thaleichthys_pacificus:10.332899):5.872101,(Osmeridae_Mallotus_villosus:14.961758,Osmeridae_Hypomesus_pretiosus:14.961758):1.243242):18.133836,(Plecoglossidae_Plecoglossus_altivelis:28.70318,Salangidae_Neosalangichthys_ishikawae:28.70318):5.635656):39.361165):55.7,((Diplophidae_Diplophos_taenia:77.330737,((Gonostomatidae_Bonapartia_pedaliota:54.297445,Gonostomatidae_Margrethia_obtusirostra:54.297445):20.014374,(Gonostomatidae_Gonostoma_elongatum:69.948163,Gonostomatidae_Cyclothone_microdon:69.948163):4.363656):3.018918):6.469264,(Phosichthyidae_Pollichthys_mauli:77.722827,((Sternoptychidae_Argyropelecus_gigas:25.230481,Sternoptychidae_Maurolicus_weitzmani:25.230481):47.329876,(Stomiidae_Chauliodus_macouni_danae:63.126299,(Stomiidae_Stomias_boa:36.381906,(Stomiidae_Chirostomias_pliopterus:31.578039,((Stomiidae_Melanostomias_margaritifer:21.790927,Stomiidae_Leptostomias_longibarba:21.790927):6.240428,((Stomiidae_Photonectes_margarita:19.053467,Stomiidae_Tactostoma_macropus:19.053467):7.977888,(Stomiidae_Malacosteus_niger:25.031355,(Stomiidae_Eustomias_polyaster:24.031355,(Stomiidae_Aristostomias_scintillans:23.031355,Stomiidae_Bathophilus_flemingi_pawneei:23.031355):1):1):2):1):3.546684):4.803867):26.744393):9.434058):5.16247):6.077174):45.6):81.9,((Ateleopodidae_Ateleopus_japonicus:8.089622,Ateleopodidae_Ijimaia_antillarum:8.089622):184.210379,(((Synodontidae_Trachinocephalus_myops:34.928634,Synodontidae_Synodus_foetens:34.928634):78.971367,((Paraulopidae_Paraulopus_oblongus:101.985288,(Synodontidae_Saurida_gracilis:50.257509,Synodontidae_Harpadon_microchir:50.257509):51.727779):8.326796,(((Aulopidae_Aulopus_filamentosus:47.160387,Bathysauridae_Bathysaurus_ferox:47.160387):10.843902,(Ipnopidae_Ipnops_murrayi:13.82,Ipnopidae_Ipnops_agassizi:13.82):44.184289):23.635948,((Ipnopidae_Bathypterois_atricolor:69.328167,(Giganturidae_Gigantura_indica:13.525058,Giganturidae_Gigantura_chuni:13.525058):55.803109):6.462647,(Scopelarchidae_Benthalbella_macropinna:62.490696,(Chlorophthalmidae_Chlorophthalmus_agassizi:61.13635,(Sudidae_Sudis_atrox:55.026295,((Alepisauridae_Anotopterus_pharao:45.661943,(Alepisauridae_Omosudis_lowii:36.284216,Alepisauridae_Alepisaurus_ferox:36.284216):9.377727):8.238175,(Paralepididae_Stemonosudis_intermedia_macrura:50.122687,(Evermannellidae_Evermannella_balbo:46.686813,Paralepididae_Paralepis_coregonoides:46.686813):3.435874):3.777431):1.126177):6.110055):1.354346):13.300118):5.849423):28.671847):3.587917):68.9,(((Neoscopelidae_Neoscopelus_macrolepidotus:42.408365,Neoscopelidae_Scopelengys_tristis:42.408365):31.191636,((Myctophidae_Lampadena_speculigera:39.81673,Myctophidae_Lampanyctus_macdonaldi:39.81673):11.872,(Myctophidae_Benthosema_glaciale:28.30241,Myctophidae_Myctophum_punctatum:28.30241):23.38632):21.911271):99.5,(((Lamprididae_Lampris_guttatus:65.552505,(Regalecidae_Regalecus_russelii:37.682943,Trachipteridae_Trachipterus_trachypterus:37.682943):27.869562):84.747496,(((Mcconichthys_longipinnis:1,(Aphredoderidae_Aphredoderus_sayanus:41.500001,Amblyopsidae_Chologaster_cornuta:41.500001):25.35122427):1,(Lateopisciculus_turrifumosus:1,(Percopsidae_Percopsis_omiscomaycus:14.617149,Percopsidae_Percopsis_transmontana:14.617149):44.86051537):8.373560898):67.13910673,(Zeidae_Zeus_faber:107.100001,(Stylephoridae_Stylephorus_chordatus:78.800001,(Merlucciidae_Merluccius_productus:45.655086,(((Macrouridae_Gadomus_dispar:9.947595,Macrouridae_Bathygadus_favosus:9.947595):28.939269,(Steindachneriidae_Steindachneria_argentea:35.929071,((Macrouridae_Malacocephalus_laevis:17.761718,Macrouridae_Trachonurus_sulcatus:17.761718):10.126413,(Macrouridae_Coelorinchus_caribbaeus:21.883517,Macrouridae_Coryphaenoides_armatus:21.883517):6.004614):8.04094):2.957793):2.613137,((Moridae_Laemonema_goodebeanorum:25.08833,(Moridae_Halargyreus_johnsonii:16.444614,(Moridae_Lepidion_ensiferus:5.35693,Moridae_Antimora_rostrata:5.35693):11.087684):8.643716):13.1716,((Phycidae_Urophycis_tenuis:5.240351,Phycidae_Urophycis_chuss:5.240351):28.607969,(Lotidae_Gaidropsarus_ensis:29.800286,(Lotidae_Lota_lota:23.935071,(Gadidae_Melanogrammus_aeglefinus:5.86839,Gadidae_Gadus_morhua:5.86839):18.066681):5.865215):4.048034):4.41161):3.240071):4.155085):33.144915):28.3):27.890331):15.309669):11.08283,(Polymixiidae_Polymixia_japonica:154.800001,(((Diretmidae_Diretmus_argenteus:53.99855,(Monocentridae_Monocentris_japonica:33.600001,((Trachichthyidae_Hoplostethus_occidentalis_atlanticus:24.361165,Trachichthyidae_Gephyroberyx_darwinii:24.361165):7.919156,(Anoplogastridae_Anoplogaster_cornuta:31.22776,Trachichthyidae_Paratrachichthys_sajademalensis:31.22776):1.052561):1.31968):20.398549):71.275924,((Berycidae_Beryx_decadactylus:70.612394,(Melamphaidae_Poromitra_crassiceps:39.138751,(Melamphaidae_Scopelogadus_beanii:32.603956,Melamphaidae_Melamphaes_suborbitalis:32.603956):6.534795):31.473643):40.295775,(((Rondeletiidae_Rondeletia_bicolor:18.616456,Rondeletiidae_Rondeletia_loricata:18.616456):36.662305,Barbourisiidae_Barbourisia_rufa:55.278761):34.605685,(Cetomimidae_Cetostoma_regani:55.492218,(Cetomimidae_Cetomimus_craneae:10.537863,Cetomimidae_Cetomimus_compuctus:10.537863):44.954355):34.392228):21.023723):14.366305):21.189147,((Holocentridae_Sargocentron_diadema:25.43456,Holocentridae_Holocentrus_rufus:25.43456):119.565441,((Ophidiidae_Brotula_multibarbata:66.227334,(Ophidiidae_Lepophidium_profundorum:23.036156,(Ophidiidae_Genypterus_blacodes:21.590629,Ophidiidae_Ophidion_holbrookii:21.590629):1.445527):43.191178):66.572667,((Batrachoididae_Porichthys_notatus:39.800001,Batrachoididae_Opsanus_tau:39.800001):87,(((Kurtidae_Kurtus_indicus:80.300001,Apogonidae_Apogon_campbelli:80.300001):22,(Eleotridae_Eleotris_acanthopoma_pisonis:64.430477,(Gobiidae_Gobiosoma_bosc:45.630567,Microdesmidae_Microdesmus_longipinnis:45.630567):18.79991):37.869524):19.3,((((Dactylopteridae_Dactylopterus_volitans:68.121568,(Aulostomidae_Aulostomus_maculatus:3.200001,Aulostomidae_Aulostomus_chinensis:3.200001):64.921567):6.178433,((Fistulariidae_Fistularia_petimba:65.463647,Mullidae_Mullus_auratus:65.463647):6.26906,(Callionymidae_Callionymus_sp_bairdi:69.154942,Syngnathidae_Syngnathus_fuscus:69.154942):2.577765):2.567294):20.27,(Chiasmodontidae_Chiasmodon_sp:43.900001,((Centrolophidae_Icichthys_lockingtoni:34.370774,Pomatomidae_Pomatomus_saltatrix:34.370774):6.934458,(((Ariommatidae_Ariomma_bondi:25.644121,Nomeidae_Psenes_cyanophrys:25.644121):5.370208,Scombridae_Auxis_rochei:31.014329):6.588438,((Stromateidae_Peprilus_paru:35.602767,(Gempylidae_Paradiplospinus_gracilis:31.499638,Bramidae_Brama_brama:31.499638):4.103129):1,(Icosteidae_Icosteus_aenigmaticus:33.972252,Trichiuridae_Trichiurus_lepturus:33.972252):2.630515):1):3.702465):2.594769):50.67):21.916687,((((((Synbranchidae_Monopterus_albus:69.146846,Indostomidae_Indostomus_paradoxus:69.146846):4.548479,(Mastacembelidae_Macrognathus_siamensis:18.057555,Mastacembelidae_Mastacembelus_erythrotaenia:18.057555):55.63777):7.004676,((Anabantidae_Ctenopoma_acutirostre_kingsleyae:62.782323,(Helostomatidae_Helostoma_temminkii:59.687826,Osphronemidae_Trichopodus_pectoralis:59.687826):3.094497):7.86905,(Channidae_Channa_striata:67.428514,Nandidae_Nandus_nandus:67.428514):3.222859):10.048628):15.7,((Nematistiidae_Nematistius_pectoralis:60.067614,((Coryphaenidae_Coryphaena_hippurus:19.124214,Rachycentridae_Rachycentron_canadum:19.124214):12.775787,(Echeneidae_Remora_osteochir_australis:10.178122,Echeneidae_Echeneis_naucrates:10.178122):21.721879):28.167613):9.732387,((Sphyraenidae_Sphyraena_sphyraena:65.900885,(Menidae_Mene_maculata:60.339531,Polynemidae_Polydactylus_octonemus:60.339531):5.561354):2.899116,((((Leptobramidae_Leptobrama_muelleri:46.58559,Toxotidae_Toxotes_jaculatrix:46.58559):5.096327,(Xiphiidae_Xiphias_gladius:27.833842,Istiophoridae_Istiophorus_platypterus:27.833842):23.848075):5.298938,Carangidae_Trachinotus_falcatus:56.980855):4.861848,((Eolates_gracilis:1,(Centropomidae_Psammoperca_waigiensis:33.068144,(Centropomidae_Lates_calcarifer:13.595548,Centropomidae_Lates_microlepis:13.595548):19.472596):23.11157544):4.662983557,(Heteronectes_chaneti:7.900994579,(Amphistium_paradoxum:7.969807984,(Psettodidae_Psettodes_erumei:57.842703,(Citharidae_Lepidoblepharon_ophthalmolepis:53.600001,((Cynoglossidae_Cynoglossus_interruptus:42.400002,(Soleidae_Solea_solea:24.944331,Soleidae_Soleichthys_heterorhinos:24.944331):17.455671):9.674607,((Scophthalmidae_Scophthalmus_aquosus:23.305906,Scophthalmidae_Lepidorhombus_boscii:23.305906):26.973285,((Paralichthyidae_Paralichthys_dentatus:40.749719,Bothidae_Bothus_lunatus:40.749719):4.387561,(Paralichthyidae_Pseudorhombus_pentophthalmus:21.404631,(((Pleuronectidae_Glyptocephalus_zachirus:5.096079,Pleuronectidae_Microstomus_pacificus:5.096079):2.538752,(Pleuronectidae_Lyopsetta_exilis:6.085211,Pleuronectidae_Hippoglossus_hippoglossus:6.085211):1.54962):2.215451,(Pleuronectidae_Limanda_limanda:8.798272,((Pleuronectidae_Platichthys_stellatus:3.717184,Pleuronectidae_Pleuronectes_platessa:3.717184):3.081088,(Pleuronectidae_Lepidopsetta_bilineata:4.798272,Pleuronectidae_Psettichthys_melanostictus:4.798272):2):2):1.05201):11.554349):23.732649):5.141911):1.795418):1.525392):4.242702):1):1):1):1):6.957298):1):26.6):12.043384,(((Polycentridae_Polycentrus_schomburgkii:94.214904,(Pholidichthyidae_Pholidichthys_leucotaenia:88.700001,Cichlidae_Cichla_temensis:88.700001):5.514903):1,(((Atherinopsidae_Atherinopsis_californiensis:48.715388,Atherinopsidae_Menidia_menidia:48.715388):22.225577,(Isonidae_Iso_sp:49.317292,(Atherinidae_Atherinomorus_stipes:43.78646,(Melanotaeniidae_Melanotaenia_splendida:39.870052,Pseudomugilidae_Pseudomugil_gertrudae:39.870052):3.916408):5.530832):21.623673):6.46052,(((Poeciliidae_Poecilia_latipinna_reticulata:14.292152,(Poeciliidae_Heterandria_formosa:11.366823,(Poeciliidae_Gambusia_affinis:5.736451,Poeciliidae_Belonesox_belizanus:5.736451):5.630372):2.925329):30.13151,(Cyprinodontidae_Cyprinodon_variegatus:40.469994,Fundulidae_Fundulus_parvipinnis:40.469994):3.953668):31.977823,(Adrianichthyidae_Oryzias_latipes:71.878571,((Zenarchopteridae_Dermogenys_collettei:29.883878,((Belonidae_Tylosurus_crocodilus:6.568056,Belonidae_Ablennes_hians:6.568056):17.429484,(Scomberesocidae_Cololabis_saira:2.646712,Scomberesocidae_Scomberesox_saurus:2.646712):21.350828):5.886338):17.178962,(Hemiramphidae_Hyporhamphus_affinis:36.892311,((Hemiramphidae_Hemiramphus_brasiliensis:13.391766,Hemiramphidae_Oxyporhamphus_micropterus:13.391766):10.259915,((Exocoetidae_Cypselurus_callopterus:6.413654,Exocoetidae_Exocoetus_monocirrhus:6.413654):4.605967,Exocoetidae_Cheilopogon_pinnatibarbatus:11.019621):12.63206):13.24063):10.170529):24.815731):4.522914):1):17.813419):1.999954,((Pseudochromidae_Halidesmus_scapularis:84.850287,Pomacentridae_Pomacentrus_brachialis:84.850287):8.187103,(Embiotocidae_Cymatogaster_aggregata:91.529056,(Mugilidae_Mugil_cephalus:89.757655,(Plesiopidae_Plesiops_coeruleolineatus:86.977996,(Opistognathidae_Opistognathus_maxillosus:79.196597,(((Gobiesocidae_Lepadichthys_lineatus:37.560081,Gobiesocidae_Gobiesox_maeandricus:37.560081):35.985217,Tripterygiidae_Enneanectes_altivelis:73.545298):3.122761,((Clinidae_Clinus_cottoides:50.741024,(Dactyloscopidae_Platygillellus_rubrocinctus:45.641062,Chaenopsidae_Chaenopsis_alepidota:45.641062):5.099962):17.520576,(Blenniidae_Stanulus_seychellensis:31.042358,Blenniidae_Blenniella_cyanostigma:31.042358):37.219242):8.406459):2.528538):7.781399):2.779659):1.771401):1.508334):4.177468):11.228527):3.450865,(Gerreidae_Eucinostomus_argenteus:108.000002,(((Labridae_Lachnolaimus_maximus:59.822938,Odacidae_Haletta_semifasciata:59.822938):16.826114,Scaridae_Scarus_globiceps:76.649052):30.35095,(((Uranoscopidae_Uranoscopus_sulphureus:83.996275,(Ammodytidae_Ammodytes_hexapterus:78.584442,Pinguipedidae_Parapercis_punctulata:78.584442):5.411833):11.903726,(((Acropomatidae_Acropoma_japonicum:90.360051,((Percophidae_Acanthaphritis_unoorum:66.16617,Creediidae_Limnichthys_sp:66.16617):18.796708,(Glaucosomatidae_Glaucosoma_buergeri:66.617288,Pempheridae_Pempheris_schomburgkii:66.617288):18.34559):5.397173):2.814411,(((Oplegnathidae_Oplegnathus_punctatus:61.206964,Kuhliidae_Kuhlia_rupestris:61.206964):7.298332,Kyphosidae_Kyphosus_sectatrix:68.505296):16.157201,(Percichthyidae_Percichthys_trucha:69.999181,((Cirrhitidae_Cirrhitichthys_falco:66.467051,Cheilodactylidae_Cheilodactylus_fasciatus:66.467051):2.53213,(Enoplosidae_Enoplosus_armatus:63.04721,Centrarchidae_Acantharchus_pomotis:63.04721):5.951971):1):14.663316):8.511965):1.725539,(Serranidae_Pseudogramma_polyacantha:82.200001,(Percidae_Perca_fluviatilis:78.577195,((Bovichtidae_Bovichtus_diacanthus:63.021097,(Nototheniidae_Notothenia_coriiceps:9.671082,(Harpagiferidae_Harpagifer_antarcticus:8.390321,(Bathydraconidae_Gymnodraco_acuticeps:7.390321,Channichthyidae_Chionodraco_rastrospinosus:7.390321):1):1.280761):53.350015):12.936917,(((Peristediidae_Peristedion_ecuadorense:54.532565,Triglidae_Prionotus_carolinus:54.532565):11.953546,(Synanceiidae_Synanceia_verrucosa:62.510934,Scorpaenidae_Scorpaenodes_guamensis:62.510934):3.975177):6.621302,(Platycephalidae_Platycephalus_indicus:70.795272,(Anoplopomatidae_Anoplopoma_fimbria:46.504594,((Bathymasteridae_Rathbunella_hypoplecta:22.798552,(Stichaeidae_Cebidichthys_violaceus:15.58542,(Zoarcidae_Zoarces_americanus_viviparus:12.493576,(Anarhichadidae_Anarrhichthys_ocellatus:9.933504,(Zaproridae_Zaprora_silenus:8.933504,Cryptacanthodidae_Cryptacanthodes_maculatus:8.933504):1):2.560072):3.091844):7.213132):19.028758,(((Aulorhynchidae_Aulorhynchus_flavidus:25.280385,Hypoptychidae_Hypoptychus_dybowskii:25.280385):1.019618,((Gasterosteidae_Culaea_inconstans:19.844899,(Gasterosteidae_Apeltes_quadracus:17.136644,Gasterosteidae_Spinachia_spinachia:17.136644):2.708255):1.782129,(Gasterosteidae_Pungitius_pungitius:16.827665,Gasterosteidae_Gasterosteus_aculeatus:16.827665):4.799363):4.672975):13.53479,(Hexagrammidae_Hexagrammos_decagrammus:27.818969,((Cyclopteridae_Cyclopterus_lumpus:20.762394,Liparidae_Liparis_pulchellus:20.762394):4.037609,(Agonidae_Hypsagonus_quadricornis:20.371382,(Cottidae_Cottus_carolinae:18.19359,Psychrolutidae_Psychrolutes_phrictus:18.19359):2.177792):4.428621):3.018966):12.015824):1.992517):4.677284):24.290678):2.312141):2.850601):2.619181):3.622806):12.7):1):10.100001,((Drepaneidae_Drepane_punctata:67.658011,Ephippidae_Chaetodipterus_faber:67.658011):37.341991,(((Lobotidae_Lobotes_pacificus_surinamensis:86.438004,Sciaenidae_Menticirrhus_undulatus_littoralis:86.438004):5.761997,((Monodactylidae_Monodactylus_sebae:70.333247,(Avitoluvarus_eocaenicus:19.81613707,(Zanclidae_Zanclus_cornutus:55.859022,Acanthuridae_Acanthurus_triostegus:55.859022):4.740979):9.733246):15.366754,((Pomacanthidae_Pomacanthus_semicirculatus:54.434118,(Leiognathidae_Leiognathus_equulus:32.700001,Chaetodontidae_Chelmon_rostratus:32.700001):21.734117):17.438962,(Emmelichthyidae_Erythrocles_schlegelii:69.871718,(Malacanthidae_Malacanthus_plumieri:68.303733,(Haemulidae_Haemulon_aurolineatum:64.600001,Lutjanidae_Lutjanus_griseus:64.600001):3.703732):1.567985):2.001362):13.826921):6.5):11.800001,((Sillaginidae_Sillago_sihama:85.853361,(Nemipteridae_Pentapodus_caninus:80.270573,(Lethrinidae_Lethrinus_erythropterus:74.370539,Sparidae_Stenotomus_chrysops:74.370539):5.900034):5.582788):17.146641,(Siganidae_Siganus_spinus:102.000002,((Scatophagidae_Scatophagus_argus:75.860371,Priacanthidae_Heteropriacanthus_cruentatus:75.860371):25.139631,((Caproidae_Antigonia_rubescens:10.905674,Caproidae_Antigonia_capros:10.905674):89.094328,(((Lophiidae_Lophiodes_reticulatus:22.462185,Lophiidae_Lophius_gastrophysus:22.462185):42.032119,((Antennariidae_Histrio_histrio:12.333102,Antennariidae_Antennatus_coccineus:12.333102):33.466899,(Chaunacidae_Chaunax_suttkusi:38.499563,(Gigantactinidae_Gigantactis_sp:32.911618,((Ceratiidae_Cryptopsaras_couesii:20.548708,Ceratiidae_Ceratias_holboelli:20.548708):10.416157,((Himantolophidae_Himantolophus_albinares_sagamius:14.456802,Melanocetidae_Melanocetus_murrayi:14.456802):9.124159,(Oneirodidae_Oneirodes_macrosteus:10.164357,Oneirodidae_Dolopichthys_sp:10.164357):13.416604):7.383904):1.946753):5.587945):7.300438):18.694303):34.505698,(((Prohollardia_avita:36.72293564,(Protacanthodes_nimesensis:1,Triacanthidae_Triacanthus_biaculeatus:46.79327682):17.69969418):10.123893,(Aracanidae_Aracana_aurita:54.800001,(Ostraciidae_Ostracion_cubicus:51.000002,Ostraciidae_Rhinesomus_triqueter:51.000002):3.799999):19.816863):3.183137,(((Diodontidae_Diodon_holocanthus:12.198387,Diodontidae_Chilomycterus_schoepfii:12.198387):43.701614,(Tetraodontidae_Canthigaster_bennetti:24.22571,Tetraodontidae_Tetraodon_miurus:24.22571):31.674291):17.334859,((Molidae_Ranzania_laevis:22,Molidae_Mola_mola:22):46.61027,((Monacanthidae_Aluterus_scriptus:27.823604,Monacanthidae_Stephanolepis_hispidus:27.823604):15.976396,(Balistidae_Xanthichthys_ringens:20.7,(Balistidae_Balistes_vetula:19.7,Balistidae_Sufflamen_fraenatum:19.7):1):23.1):24.81027):4.62459):4.565141):21.200001):1):1):1):1):1):1):1):1):1):3.894248):4.592438):5.113313):5.2):6):12.2):1.46362):8.33638):6.58283):11.71717):9.7):9.5):19):3.553787):17.946213):17.8):23.2):9.5):1):1):37.2):1):1):1):1):23.6):1):6.558106552):1):6.940087955):20.97429617):1):1):1.039733166):1):4.387777153):1):47);

((Guiyu_oneiros:1,(Onychodus_jandemarrai:35.95643967,((Diplocercides:1,(Latimeriidae_Latimeria_chalumnae:330.8519472,Rhabdoderma:1):35.21577325):46.4479066,((Neoceratodontidae_Neoceratodus_forsteri:279.7982796,Lepidosirenidae_Lepidosiren_paradoxa:279.7982796):128.2017204,((Osteolepis_macrolepidotus:1,Gyroptychius_milleri:7.806090736):1,Eusthenopterus_foordi:21.39326682):7.805782067):4.515627):1):13.48400314):16.00037186,((Cheirolepis_trailli:1,Cheirolepis_schultzei:6.562550873):2.710300333,((Polypteridae_Erpetoichthys_calabaricus:29.200001,Polypteridae_Polypterus_senegalus:29.200001):365.800001,(Osorioichthys_marginis:25.59867897,((Tegeolepis_clarki:15.43428326,Howqualepis_rostridens:1):4.129181474,((Gogosardina_coatesi:3.072706217,(Mimipiscis_bartrami:1,Mimipiscis_toombsi:5.231320745):1):5.806846747,((Moythomasia_lineata:1,Moythomasia_durgaringa:12.1358468):1.811740139,(Stegotrachelus_finlayi:1,(Limnomis_delaneyi:1,(Wendyichthys_dicksoni:29.58864968,(Kentuckia_deani:1,((Mesopoma_planti:1,Mesopoma_carricki:9.058389735):6.577313546,((Birgeria_stensioei:1,(Chondrosteus_acipenseroides:1,(Saurichthys_dawaziensis:1,((Protopsephurus_liui:1,Polyodontidae_Polyodon_spathula:130.3866202):8.513381812,(Acipenseridae_Acipenser_fulvescens:38.579335,(Acipenseridae_Scaphirhynchus_platorynchus:19.375515,Acipenseridae_Scaphirhynchus_albus:19.375515):19.20382):100.320667):25.31885321):37.97751862):50.59562242):97.30800475,(Boreosomus:74.46425932,((Australosomus:2.716298839,(Fukangichthys_longidorsalis:6.605665101,(Scanilepis_dubia:44.41275189,Evenkia_eunotoptera:1):1):1):70.94675549,(Perleidus_altolepis:84.52096274,(Luganoia_lepidosteoides:89.7445247,(((Macrosemius_fourneti:93.06953239,((Semionotus_elegans:46.64591514,Kyphosichthys_grandei:1):1,Lepisosteidae_Lepisosteus_osseus:248.5496693):1):18.35033172,(Watsonulus_eugnathoides:1,(Amblysemius:1,(Pachyamia_latimaxillaris:2.403436902,(Tomognathus_mordax:1,Amiidae_Amia_calva:97.18868896):1):50.33171948):104.0731189):15.30647365):54.6,(Pachycormus:105.7042979,(Crossognathus_danubiensis:172.8796309,(((Anaethalion_angustus:4.513797743,Anaethalion_knorri:1):45.06865764,((Megalopidae_Megalops_atlanticus:133.565966,Elopidae_Elops_saurus:133.565966):62.634035,(Albulidae_Albula_vulpes:150.800001,((Notacanthidae_Notacanthus_chemnitzii:50.669287,(Halosauridae_Halosauropsis_macrochir:40.366211,Halosauridae_Aldrovandia_affinis:40.366211):10.303076):50.330714,(((Eurypharyngidae_Eurypharynx_pelecanoides:25.866364,Saccopharyngidae_Saccopharynx_ampullaceus:25.866364):44.322494,(Nemichthyidae_Nemichthys_scolopaceus:58.739485,Anguillidae_Anguilla_rostrata:58.739485):11.449373):9.198895,(Serrivomeridae_Serrivomer_beanii:76.053487,(Congridae_Conger_oceanicus:65.416147,(Muraenesocidae_Muraenesox_cinereus:57.193624,Ophichthidae_Myrichthys_maculosus:57.193624):8.222523):10.63734):3.334266):21.612248):49.8):45.4):1):86.1,((Lycoptera_davidi:99.23609009,(Paralycoptera_wui:112.1498093,(Xixiaichthys_tongxinens:101.6323267,(((Eohiodon_woodruffi:1,Eohiodon_rosei:1.097518247):1,(Hiodon_consteniorum:1,(Hiodontidae_Hiodon_tergisus:9.520291,Hiodontidae_Hiodon_alosoides:9.520291):26.283608):20.65637907):170.6397229,(Chauliopareion_mahengeense:119.1897926,(Pantodontidae_Pantodon_buchholzi:163.100001,(((Arapaimidae_Arapaima_gigas:79.3,(Brychaetus_muelleri:1,Phareodus_encaustus:10.20655754):22.9653313):25.549112,Osteoglossidae_Osteoglossum_bicirrhosum:104.849112):43.475578,(Notopteridae_Xenomystus_nigri:120.300002,Gymnarchidae_Gymnarchus_niloticus:120.300002):28.024688):14.775311):1):63):1):1):1):43.7,((((Diplomystus_brevissimus:47.47656903,Sorbinichthys_africanus:1):91.14957908,(Denticipitidae_Denticeps_clupeoides:188.900001,(Chirocentridae_Chirocentrus_dorab:87.096454,((Engraulidae_Coilia_nasus:47.06527,Engraulidae_Engraulis_mordax_eurystole:47.06527):33.347234,((Pristigasteridae_Pellona_flavipinnis:27.450803,Pristigasteridae_Ilisha_elongata:27.450803):46.430359,(Clupeidae_Alosa_pseudoharengus:54.138864,Clupeidae_Dorosoma_cepedianum:54.138864):19.742298):6.531342):6.68395):101.803547):1):40.3,((Alepocephalidae_Talismania_bifurcata:53.266521,((Alepocephalidae_Bathylaco_nigricans:33.802485,Alepocephalidae_Alepocephalus_tenebrosus:33.802485):10.988666,((Alepocephalidae_Rouleina_attrita:22.275085,Alepocephalidae_Xenodermichthys_copei:22.275085):19.924916,Platytroctidae_Sagamichthys_abei:42.200001):2.59115):8.47537):166.430015,((Gonorynchidae_Gonorynchus_abbreviatus:175.900001,(Mahengichthys_singidaensis:103.4310151,Chanidae_Chanos_chanos:147.100001):28.8):22.7,((((Gyrinocheilidae_Gyrinocheilus_sp:70.111694,(Catostomidae_Hypentelium_nigricans:23.594381,(Catostomidae_Carpiodes_carpio:16.355089,Catostomidae_Ictiobus_bubalus:16.355089):7.239292):46.517313):8.688307,Cobitidae_Cobitis_taenia:78.800001):20.5,(Cyprinidae_Danio_rerio:63.300001,(((Cyprinidae_Zacco_sieboldii_platypus:12.727958,Cyprinidae_Opsariichthys_uncirostris_bidens:12.727958):14.610086,((Cyprinidae_Xenocypris_argentea:12.904177,Cyprinidae_Hypophthalmichthys_molitrix:12.904177):2.917503,(Cyprinidae_Luciobrama_macrocephalus:13.439618,(Cyprinidae_Squaliobarbus_curriculus:11.472717,Cyprinidae_Mylopharyngodon_piceus:11.472717):1.966901):2.382062):11.516364):12.401957,(Cyprinidae_Tanakia_lanceolata_himantegus:35.004261,(Cyprinidae_Notemigonus_crysoleucas:22.933404,(Cyprinidae_Semotilus_atromaculatus:21.325215,((Cyprinidae_Campostoma_oligolepis:12.800846,Cyprinidae_Rhinichthys_cataractae:12.800846):4.408707,(Cyprinidae_Phenacobius_uranops:12.822623,(Cyprinidae_Pimephales_promelas_notatus:9.460336,(Cyprinidae_Luxilus_coccogenis:6.738861,Cyprinidae_Notropis_asperifrons:6.738861):2.721475):3.362287):4.38693):4.115662):1.608189):12.070857):4.73574):23.56):36):73.6,((Gymnotidae_Electrophorus_electricus:63.909447,Gymnotidae_Gymnotus_sp:63.909447):83.924067,(((Distichodontidae_Distichodus_maculatus:103.604365,Citharinidae_Citharinus_congicus:103.604365):11.170138,(Alestidae_Alestes_baremoze:106.666979,((Parodontidae_Parodon_nasus:68.800001,Hemiodontidae_Hemiodus_immaculatus:68.800001):22.415197,((Bryconidae_Brycon_pesu:66.003418,(Gasteropelecidae_Thoracocharax_stellatus:27.746828,Gasteropelecidae_Gasteropelecus_sternicla:27.746828):38.25659):1.227196,Characidae_Astyanax_mexicanus:67.230614):23.984584):15.451781):8.107524):22.236489,((Nematogenyidae_Nematogenys_inermis:110.648758,(Trichomycteridae_Trichomycterus_sp:105.886466,((Loricariidae_Loricaria_simillima:70.313425,Astroblepidae_Astroblepus_sp:70.313425):28.122359,(Callichthyidae_Callichthys_callichthys:69.200001,Callichthyidae_Corydoras_trilineatus:69.200001):29.235783):7.450682):4.762292):5.151243,(Diplomystidae_Diplomystes_nahuelbutaensis:106.203801,((Clariidae_Clarias_batrachus:47.60069,Heteropneustidae_Heteropneustes_fossilis:47.60069):38.718856,(((Sisoridae_Bagarius_yarrelli:55.522058,(Amblycipitidae_Liobagrus_aequilabris:48.027606,Akysidae_Akysis_sp:48.027606):7.494452):21.358911,(Schilbeidae_Pseudeutropius_brachypopterus:74.448774,(Bagridae_Mystus_bocourti:36.077943,Bagridae_Bagrus_ubangensis:36.077943):38.370831):2.432195):8.408138,(((Chacidae_Chaca_sp:72.429482,Cetopsidae_Cetopsis_coecutiens:72.429482):10.714797,(Cranoglanididae_Cranoglanis_bouderius:68.700001,Ictaluridae_Ictalurus_punctatus:68.700001):14.444278):1.039619,(Pangasiidae_Pangasianodon_hypophthalmus:82.158061,((Amphiliidae_Amphilius_jacksonii:69.199757,(Malapteruridae_Malapterurus_beninensis:64.740321,Mochokidae_Synodontis_batesii:64.740321):4.459436):10.993535,((Plotosidae_Plotosus_lineatus:73.039645,Siluridae_Hemisilurus_moolenburghi:73.039645):5.503862,(Auchenipteridae_Ageneiosus_atronasus:51.101654,Doradidae_Anduzedoras_oxyrhynchus:51.101654):27.441853):1.649785):1.964769):2.025837):1.105209):1.030439):19.884255):9.5962):21.210991):10.822522):25.066487):25.7):21.096535):10.503465):20.4,(Orthogonikleithrus_francogalliensis:79.07242978,(((Bathylagidae_Bathylagus_euryops:70.542512,(Microstomatidae_Nansenia_longicauda_ardesiaca:51.394,(Opisthoproctidae_Macropinna_microstoma:33.11268,Argentinidae_Argentina_sialis_silus:33.11268):18.28132):19.148512):89.057489,((Galaxiidae_Neochanna_burrowsius:34.505049,Galaxiidae_Galaxias_maculatus:34.505049):111.121934,(((Umbridae_Novumbra_hubbsi:55.762491,Umbridae_Umbra_limi:55.762491):23.63751,(Esocidae_Esox_lucius:31.366195,Esocidae_Esox_americanus:31.366195):48.033806):25.064928,(Salmonidae_Coregonus_clupeaformis:35.300001,(Salmonidae_Thymallus_brevirostris:32.456522,(Salmonidae_Oncorhynchus_nerka_mykiss:19.929533,(Salmonidae_Salvelinus_alpinus:16.737138,Salmonidae_Salmo_salar:16.737138):3.192395):12.526989):2.843479):69.164928):41.162054):13.973018):55.253787,(((Retropinnidae_Retropinna_semoni:73.700001,(((Osmeridae_Osmerus_mordax:10.332899,Osmeridae_Thaleichthys_pacificus:10.332899):5.872101,(Osmeridae_Mallotus_villosus:14.961758,Osmeridae_Hypomesus_pretiosus:14.961758):1.243242):18.133836,(Plecoglossidae_Plecoglossus_altivelis:28.70318,Salangidae_Neosalangichthys_ishikawae:28.70318):5.635656):39.361165):55.7,((Diplophidae_Diplophos_taenia:77.330737,((Gonostomatidae_Bonapartia_pedaliota:54.297445,Gonostomatidae_Margrethia_obtusirostra:54.297445):20.014374,(Gonostomatidae_Gonostoma_elongatum:69.948163,Gonostomatidae_Cyclothone_microdon:69.948163):4.363656):3.018918):6.469264,(Phosichthyidae_Pollichthys_mauli:77.722827,((Sternoptychidae_Argyropelecus_gigas:25.230481,Sternoptychidae_Maurolicus_weitzmani:25.230481):47.329876,(Stomiidae_Chauliodus_macouni_danae:63.126299,(Stomiidae_Stomias_boa:36.381906,(Stomiidae_Chirostomias_pliopterus:31.578039,((Stomiidae_Melanostomias_margaritifer:21.790927,Stomiidae_Leptostomias_longibarba:21.790927):6.240428,((Stomiidae_Photonectes_margarita:19.053467,Stomiidae_Tactostoma_macropus:19.053467):7.977888,(Stomiidae_Malacosteus_niger:25.031355,(Stomiidae_Eustomias_polyaster:24.031355,(Stomiidae_Aristostomias_scintillans:23.031355,Stomiidae_Bathophilus_flemingi_pawneei:23.031355):1):1):2):1):3.546684):4.803867):26.744393):9.434058):5.16247):6.077174):45.6):81.9,((Ateleopodidae_Ateleopus_japonicus:8.089622,Ateleopodidae_Ijimaia_antillarum:8.089622):184.210379,(((Synodontidae_Trachinocephalus_myops:34.928634,Synodontidae_Synodus_foetens:34.928634):78.971367,((Paraulopidae_Paraulopus_oblongus:101.985288,(Synodontidae_Saurida_gracilis:50.257509,Synodontidae_Harpadon_microchir:50.257509):51.727779):8.326796,(((Aulopidae_Aulopus_filamentosus:47.160387,Bathysauridae_Bathysaurus_ferox:47.160387):10.843902,(Ipnopidae_Ipnops_murrayi:13.82,Ipnopidae_Ipnops_agassizi:13.82):44.184289):23.635948,((Ipnopidae_Bathypterois_atricolor:69.328167,(Giganturidae_Gigantura_indica:13.525058,Giganturidae_Gigantura_chuni:13.525058):55.803109):6.462647,(Scopelarchidae_Benthalbella_macropinna:62.490696,(Chlorophthalmidae_Chlorophthalmus_agassizi:61.13635,(Sudidae_Sudis_atrox:55.026295,((Alepisauridae_Anotopterus_pharao:45.661943,(Alepisauridae_Omosudis_lowii:36.284216,Alepisauridae_Alepisaurus_ferox:36.284216):9.377727):8.238175,(Paralepididae_Stemonosudis_intermedia_macrura:50.122687,(Evermannellidae_Evermannella_balbo:46.686813,Paralepididae_Paralepis_coregonoides:46.686813):3.435874):3.777431):1.126177):6.110055):1.354346):13.300118):5.849423):28.671847):3.587917):68.9,(((Neoscopelidae_Neoscopelus_macrolepidotus:42.408365,Neoscopelidae_Scopelengys_tristis:42.408365):31.191636,((Myctophidae_Lampadena_speculigera:39.81673,Myctophidae_Lampanyctus_macdonaldi:39.81673):11.872,(Myctophidae_Benthosema_glaciale:28.30241,Myctophidae_Myctophum_punctatum:28.30241):23.38632):21.911271):99.5,(((Lamprididae_Lampris_guttatus:65.552505,(Regalecidae_Regalecus_russelii:37.682943,Trachipteridae_Trachipterus_trachypterus:37.682943):27.869562):84.747496,(((Mcconichthys_longipinnis:1,(Aphredoderidae_Aphredoderus_sayanus:41.500001,Amblyopsidae_Chologaster_cornuta:41.500001):24.48261412):1,(Lateopisciculus_turrifumosus:1,(Percopsidae_Percopsis_omiscomaycus:14.617149,Percopsidae_Percopsis_transmontana:14.617149):45.39118285):6.97428327):68.00771688,(Zeidae_Zeus_faber:107.100001,(Stylephoridae_Stylephorus_chordatus:78.800001,(Merlucciidae_Merluccius_productus:45.655086,(((Macrouridae_Gadomus_dispar:9.947595,Macrouridae_Bathygadus_favosus:9.947595):28.939269,(Steindachneriidae_Steindachneria_argentea:35.929071,((Macrouridae_Malacocephalus_laevis:17.761718,Macrouridae_Trachonurus_sulcatus:17.761718):10.126413,(Macrouridae_Coelorinchus_caribbaeus:21.883517,Macrouridae_Coryphaenoides_armatus:21.883517):6.004614):8.04094):2.957793):2.613137,((Moridae_Laemonema_goodebeanorum:25.08833,(Moridae_Halargyreus_johnsonii:16.444614,(Moridae_Lepidion_ensiferus:5.35693,Moridae_Antimora_rostrata:5.35693):11.087684):8.643716):13.1716,((Phycidae_Urophycis_tenuis:5.240351,Phycidae_Urophycis_chuss:5.240351):28.607969,(Lotidae_Gaidropsarus_ensis:29.800286,(Lotidae_Lota_lota:23.935071,(Gadidae_Melanogrammus_aeglefinus:5.86839,Gadidae_Gadus_morhua:5.86839):18.066681):5.865215):4.048034):4.41161):3.240071):4.155085):33.144915):28.3):27.890331):15.309669):11.08283,(Polymixiidae_Polymixia_japonica:154.800001,(((Diretmidae_Diretmus_argenteus:53.99855,(Monocentridae_Monocentris_japonica:33.600001,((Trachichthyidae_Hoplostethus_occidentalis_atlanticus:24.361165,Trachichthyidae_Gephyroberyx_darwinii:24.361165):7.919156,(Anoplogastridae_Anoplogaster_cornuta:31.22776,Trachichthyidae_Paratrachichthys_sajademalensis:31.22776):1.052561):1.31968):20.398549):71.275924,((Berycidae_Beryx_decadactylus:70.612394,(Melamphaidae_Poromitra_crassiceps:39.138751,(Melamphaidae_Scopelogadus_beanii:32.603956,Melamphaidae_Melamphaes_suborbitalis:32.603956):6.534795):31.473643):40.295775,(((Rondeletiidae_Rondeletia_bicolor:18.616456,Rondeletiidae_Rondeletia_loricata:18.616456):36.662305,Barbourisiidae_Barbourisia_rufa:55.278761):34.605685,(Cetomimidae_Cetostoma_regani:55.492218,(Cetomimidae_Cetomimus_craneae:10.537863,Cetomimidae_Cetomimus_compuctus:10.537863):44.954355):34.392228):21.023723):14.366305):21.189147,((Holocentridae_Sargocentron_diadema:25.43456,Holocentridae_Holocentrus_rufus:25.43456):119.565441,((Ophidiidae_Brotula_multibarbata:66.227334,(Ophidiidae_Lepophidium_profundorum:23.036156,(Ophidiidae_Genypterus_blacodes:21.590629,Ophidiidae_Ophidion_holbrookii:21.590629):1.445527):43.191178):66.572667,((Batrachoididae_Porichthys_notatus:39.800001,Batrachoididae_Opsanus_tau:39.800001):87,(((Kurtidae_Kurtus_indicus:80.300001,Apogonidae_Apogon_campbelli:80.300001):22,(Eleotridae_Eleotris_acanthopoma_pisonis:64.430477,(Gobiidae_Gobiosoma_bosc:45.630567,Microdesmidae_Microdesmus_longipinnis:45.630567):18.79991):37.869524):19.3,((((Dactylopteridae_Dactylopterus_volitans:68.121568,(Aulostomidae_Aulostomus_maculatus:3.200001,Aulostomidae_Aulostomus_chinensis:3.200001):64.921567):6.178433,((Fistulariidae_Fistularia_petimba:65.463647,Mullidae_Mullus_auratus:65.463647):6.26906,(Callionymidae_Callionymus_sp_bairdi:69.154942,Syngnathidae_Syngnathus_fuscus:69.154942):2.577765):2.567294):20.27,(Chiasmodontidae_Chiasmodon_sp:43.900001,((Centrolophidae_Icichthys_lockingtoni:34.370774,Pomatomidae_Pomatomus_saltatrix:34.370774):6.934458,(((Ariommatidae_Ariomma_bondi:25.644121,Nomeidae_Psenes_cyanophrys:25.644121):5.370208,Scombridae_Auxis_rochei:31.014329):6.588438,((Stromateidae_Peprilus_paru:35.602767,(Gempylidae_Paradiplospinus_gracilis:31.499638,Bramidae_Brama_brama:31.499638):4.103129):1,(Icosteidae_Icosteus_aenigmaticus:33.972252,Trichiuridae_Trichiurus_lepturus:33.972252):2.630515):1):3.702465):2.594769):50.67):21.916687,((((((Synbranchidae_Monopterus_albus:69.146846,Indostomidae_Indostomus_paradoxus:69.146846):4.548479,(Mastacembelidae_Macrognathus_siamensis:18.057555,Mastacembelidae_Mastacembelus_erythrotaenia:18.057555):55.63777):7.004676,((Anabantidae_Ctenopoma_acutirostre_kingsleyae:62.782323,(Helostomatidae_Helostoma_temminkii:59.687826,Osphronemidae_Trichopodus_pectoralis:59.687826):3.094497):7.86905,(Channidae_Channa_striata:67.428514,Nandidae_Nandus_nandus:67.428514):3.222859):10.048628):15.7,((Nematistiidae_Nematistius_pectoralis:60.067614,((Coryphaenidae_Coryphaena_hippurus:19.124214,Rachycentridae_Rachycentron_canadum:19.124214):12.775787,(Echeneidae_Remora_osteochir_australis:10.178122,Echeneidae_Echeneis_naucrates:10.178122):21.721879):28.167613):9.732387,((Sphyraenidae_Sphyraena_sphyraena:65.900885,(Menidae_Mene_maculata:60.339531,Polynemidae_Polydactylus_octonemus:60.339531):5.561354):2.899116,((((Leptobramidae_Leptobrama_muelleri:46.58559,Toxotidae_Toxotes_jaculatrix:46.58559):5.096327,(Xiphiidae_Xiphias_gladius:27.833842,Istiophoridae_Istiophorus_platypterus:27.833842):23.848075):5.298938,Carangidae_Trachinotus_falcatus:56.980855):4.861848,((Eolates_gracilis:1,(Centropomidae_Psammoperca_waigiensis:33.068144,(Centropomidae_Lates_calcarifer:13.595548,Centropomidae_Lates_microlepis:13.595548):19.472596):22.94466039):4.82989861,(Heteronectes_chaneti:4.679321497,(Amphistium_paradoxum:4.513404568,(Psettodidae_Psettodes_erumei:57.842703,(Citharidae_Lepidoblepharon_ophthalmolepis:53.600001,((Cynoglossidae_Cynoglossus_interruptus:42.400002,(Soleidae_Solea_solea:24.944331,Soleidae_Soleichthys_heterorhinos:24.944331):17.455671):9.674607,((Scophthalmidae_Scophthalmus_aquosus:23.305906,Scophthalmidae_Lepidorhombus_boscii:23.305906):26.973285,((Paralichthyidae_Paralichthys_dentatus:40.749719,Bothidae_Bothus_lunatus:40.749719):4.387561,(Paralichthyidae_Pseudorhombus_pentophthalmus:21.404631,(((Pleuronectidae_Glyptocephalus_zachirus:5.096079,Pleuronectidae_Microstomus_pacificus:5.096079):2.538752,(Pleuronectidae_Lyopsetta_exilis:6.085211,Pleuronectidae_Hippoglossus_hippoglossus:6.085211):1.54962):2.215451,(Pleuronectidae_Limanda_limanda:8.798272,((Pleuronectidae_Platichthys_stellatus:3.717184,Pleuronectidae_Pleuronectes_platessa:3.717184):3.081088,(Pleuronectidae_Lepidopsetta_bilineata:4.798272,Pleuronectidae_Psettichthys_melanostictus:4.798272):2):2):1.05201):11.554349):23.732649):5.141911):1.795418):1.525392):4.242702):1):1):1):1):6.957298):1):26.6):12.043384,(((Polycentridae_Polycentrus_schomburgkii:94.214904,(Pholidichthyidae_Pholidichthys_leucotaenia:88.700001,Cichlidae_Cichla_temensis:88.700001):5.514903):1,(((Atherinopsidae_Atherinopsis_californiensis:48.715388,Atherinopsidae_Menidia_menidia:48.715388):22.225577,(Isonidae_Iso_sp:49.317292,(Atherinidae_Atherinomorus_stipes:43.78646,(Melanotaeniidae_Melanotaenia_splendida:39.870052,Pseudomugilidae_Pseudomugil_gertrudae:39.870052):3.916408):5.530832):21.623673):6.46052,(((Poeciliidae_Poecilia_latipinna_reticulata:14.292152,(Poeciliidae_Heterandria_formosa:11.366823,(Poeciliidae_Gambusia_affinis:5.736451,Poeciliidae_Belonesox_belizanus:5.736451):5.630372):2.925329):30.13151,(Cyprinodontidae_Cyprinodon_variegatus:40.469994,Fundulidae_Fundulus_parvipinnis:40.469994):3.953668):31.977823,(Adrianichthyidae_Oryzias_latipes:71.878571,((Zenarchopteridae_Dermogenys_collettei:29.883878,((Belonidae_Tylosurus_crocodilus:6.568056,Belonidae_Ablennes_hians:6.568056):17.429484,(Scomberesocidae_Cololabis_saira:2.646712,Scomberesocidae_Scomberesox_saurus:2.646712):21.350828):5.886338):17.178962,(Hemiramphidae_Hyporhamphus_affinis:36.892311,((Hemiramphidae_Hemiramphus_brasiliensis:13.391766,Hemiramphidae_Oxyporhamphus_micropterus:13.391766):10.259915,((Exocoetidae_Cypselurus_callopterus:6.413654,Exocoetidae_Exocoetus_monocirrhus:6.413654):4.605967,Exocoetidae_Cheilopogon_pinnatibarbatus:11.019621):12.63206):13.24063):10.170529):24.815731):4.522914):1):17.813419):1.999954,((Pseudochromidae_Halidesmus_scapularis:84.850287,Pomacentridae_Pomacentrus_brachialis:84.850287):8.187103,(Embiotocidae_Cymatogaster_aggregata:91.529056,(Mugilidae_Mugil_cephalus:89.757655,(Plesiopidae_Plesiops_coeruleolineatus:86.977996,(Opistognathidae_Opistognathus_maxillosus:79.196597,(((Gobiesocidae_Lepadichthys_lineatus:37.560081,Gobiesocidae_Gobiesox_maeandricus:37.560081):35.985217,Tripterygiidae_Enneanectes_altivelis:73.545298):3.122761,((Clinidae_Clinus_cottoides:50.741024,(Dactyloscopidae_Platygillellus_rubrocinctus:45.641062,Chaenopsidae_Chaenopsis_alepidota:45.641062):5.099962):17.520576,(Blenniidae_Stanulus_seychellensis:31.042358,Blenniidae_Blenniella_cyanostigma:31.042358):37.219242):8.406459):2.528538):7.781399):2.779659):1.771401):1.508334):4.177468):11.228527):3.450865,(Gerreidae_Eucinostomus_argenteus:108.000002,(((Labridae_Lachnolaimus_maximus:59.822938,Odacidae_Haletta_semifasciata:59.822938):16.826114,Scaridae_Scarus_globiceps:76.649052):30.35095,(((Uranoscopidae_Uranoscopus_sulphureus:83.996275,(Ammodytidae_Ammodytes_hexapterus:78.584442,Pinguipedidae_Parapercis_punctulata:78.584442):5.411833):11.903726,(((Acropomatidae_Acropoma_japonicum:90.360051,((Percophidae_Acanthaphritis_unoorum:66.16617,Creediidae_Limnichthys_sp:66.16617):18.796708,(Glaucosomatidae_Glaucosoma_buergeri:66.617288,Pempheridae_Pempheris_schomburgkii:66.617288):18.34559):5.397173):2.814411,(((Oplegnathidae_Oplegnathus_punctatus:61.206964,Kuhliidae_Kuhlia_rupestris:61.206964):7.298332,Kyphosidae_Kyphosus_sectatrix:68.505296):16.157201,(Percichthyidae_Percichthys_trucha:69.999181,((Cirrhitidae_Cirrhitichthys_falco:66.467051,Cheilodactylidae_Cheilodactylus_fasciatus:66.467051):2.53213,(Enoplosidae_Enoplosus_armatus:63.04721,Centrarchidae_Acantharchus_pomotis:63.04721):5.951971):1):14.663316):8.511965):1.725539,(Serranidae_Pseudogramma_polyacantha:82.200001,(Percidae_Perca_fluviatilis:78.577195,((Bovichtidae_Bovichtus_diacanthus:63.021097,(Nototheniidae_Notothenia_coriiceps:9.671082,(Harpagiferidae_Harpagifer_antarcticus:8.390321,(Bathydraconidae_Gymnodraco_acuticeps:7.390321,Channichthyidae_Chionodraco_rastrospinosus:7.390321):1):1.280761):53.350015):12.936917,(((Peristediidae_Peristedion_ecuadorense:54.532565,Triglidae_Prionotus_carolinus:54.532565):11.953546,(Synanceiidae_Synanceia_verrucosa:62.510934,Scorpaenidae_Scorpaenodes_guamensis:62.510934):3.975177):6.621302,(Platycephalidae_Platycephalus_indicus:70.795272,(Anoplopomatidae_Anoplopoma_fimbria:46.504594,((Bathymasteridae_Rathbunella_hypoplecta:22.798552,(Stichaeidae_Cebidichthys_violaceus:15.58542,(Zoarcidae_Zoarces_americanus_viviparus:12.493576,(Anarhichadidae_Anarrhichthys_ocellatus:9.933504,(Zaproridae_Zaprora_silenus:8.933504,Cryptacanthodidae_Cryptacanthodes_maculatus:8.933504):1):2.560072):3.091844):7.213132):19.028758,(((Aulorhynchidae_Aulorhynchus_flavidus:25.280385,Hypoptychidae_Hypoptychus_dybowskii:25.280385):1.019618,((Gasterosteidae_Culaea_inconstans:19.844899,(Gasterosteidae_Apeltes_quadracus:17.136644,Gasterosteidae_Spinachia_spinachia:17.136644):2.708255):1.782129,(Gasterosteidae_Pungitius_pungitius:16.827665,Gasterosteidae_Gasterosteus_aculeatus:16.827665):4.799363):4.672975):13.53479,(Hexagrammidae_Hexagrammos_decagrammus:27.818969,((Cyclopteridae_Cyclopterus_lumpus:20.762394,Liparidae_Liparis_pulchellus:20.762394):4.037609,(Agonidae_Hypsagonus_quadricornis:20.371382,(Cottidae_Cottus_carolinae:18.19359,Psychrolutidae_Psychrolutes_phrictus:18.19359):2.177792):4.428621):3.018966):12.015824):1.992517):4.677284):24.290678):2.312141):2.850601):2.619181):3.622806):12.7):1):10.100001,((Drepaneidae_Drepane_punctata:67.658011,Ephippidae_Chaetodipterus_faber:67.658011):37.341991,(((Lobotidae_Lobotes_pacificus_surinamensis:86.438004,Sciaenidae_Menticirrhus_undulatus_littoralis:86.438004):5.761997,((Monodactylidae_Monodactylus_sebae:70.333247,(Avitoluvarus_eocaenicus:21.27492449,(Zanclidae_Zanclus_cornutus:55.859022,Acanthuridae_Acanthurus_triostegus:55.859022):4.740979):9.733246):15.366754,((Pomacanthidae_Pomacanthus_semicirculatus:54.434118,(Leiognathidae_Leiognathus_equulus:32.700001,Chaetodontidae_Chelmon_rostratus:32.700001):21.734117):17.438962,(Emmelichthyidae_Erythrocles_schlegelii:69.871718,(Malacanthidae_Malacanthus_plumieri:68.303733,(Haemulidae_Haemulon_aurolineatum:64.600001,Lutjanidae_Lutjanus_griseus:64.600001):3.703732):1.567985):2.001362):13.826921):6.5):11.800001,((Sillaginidae_Sillago_sihama:85.853361,(Nemipteridae_Pentapodus_caninus:80.270573,(Lethrinidae_Lethrinus_erythropterus:74.370539,Sparidae_Stenotomus_chrysops:74.370539):5.900034):5.582788):17.146641,(Siganidae_Siganus_spinus:102.000002,((Scatophagidae_Scatophagus_argus:75.860371,Priacanthidae_Heteropriacanthus_cruentatus:75.860371):25.139631,((Caproidae_Antigonia_rubescens:10.905674,Caproidae_Antigonia_capros:10.905674):89.094328,(((Lophiidae_Lophiodes_reticulatus:22.462185,Lophiidae_Lophius_gastrophysus:22.462185):42.032119,((Antennariidae_Histrio_histrio:12.333102,Antennariidae_Antennatus_coccineus:12.333102):33.466899,(Chaunacidae_Chaunax_suttkusi:38.499563,(Gigantactinidae_Gigantactis_sp:32.911618,((Ceratiidae_Cryptopsaras_couesii:20.548708,Ceratiidae_Ceratias_holboelli:20.548708):10.416157,((Himantolophidae_Himantolophus_albinares_sagamius:14.456802,Melanocetidae_Melanocetus_murrayi:14.456802):9.124159,(Oneirodidae_Oneirodes_macrosteus:10.164357,Oneirodidae_Dolopichthys_sp:10.164357):13.416604):7.383904):1.946753):5.587945):7.300438):18.694303):34.505698,(((Prohollardia_avita:38.83283487,(Protacanthodes_nimesensis:1,Triacanthidae_Triacanthus_biaculeatus:46.28975612):18.20321488):10.123893,(Aracanidae_Aracana_aurita:54.800001,(Ostraciidae_Ostracion_cubicus:51.000002,Ostraciidae_Rhinesomus_triqueter:51.000002):3.799999):19.816863):3.183137,(((Diodontidae_Diodon_holocanthus:12.198387,Diodontidae_Chilomycterus_schoepfii:12.198387):43.701614,(Tetraodontidae_Canthigaster_bennetti:24.22571,Tetraodontidae_Tetraodon_miurus:24.22571):31.674291):17.334859,((Molidae_Ranzania_laevis:22,Molidae_Mola_mola:22):46.61027,((Monacanthidae_Stephanolepis_hispidus:28.823604,Monacanthidae_Aluterus_scriptus:28.823604):14.976396,(Balistidae_Xanthichthys_ringens:20.7,(Balistidae_Balistes_vetula:19.7,Balistidae_Sufflamen_fraenatum:19.7):1):23.1):24.81027):4.62459):4.565141):21.200001):1):1):1):1):1):1):1):1):1):3.894248):4.592438):5.113313):5.2):6):12.2):1.46362):8.33638):6.58283):11.71717):9.7):9.5):19):3.553787):17.946213):17.8):23.2):9.5):1):1):37.2):1):1):1):1):23.6):1):6.574060777):1):12.90697246):16.49583302):1):1):1):1):2.923134752):1):47);

((Guiyu_oneiros:1,(Onychodus_jandemarrai:34.87333829,((Diplocercides:1,(Latimeriidae_Latimeria_chalumnae:325.5007762,Rhabdoderma:1):43.24993805):43.76491279,((Neoceratodontidae_Neoceratodus_forsteri:279.7982796,Lepidosirenidae_Lepidosiren_paradoxa:279.7982796):128.2017204,((Osteolepis_macrolepidotus:1,Gyroptychius_milleri:5.2259567):1,Eusthenopterus_foordi:16.86553308):9.820554717):4.515627):1):13.18217152):16.30220348,((Cheirolepis_trailli:1,Cheirolepis_schultzei:5.583605662):2.772793364,((Polypteridae_Erpetoichthys_calabaricus:29.200001,Polypteridae_Polypterus_senegalus:29.200001):365.800001,(Osorioichthys_marginis:19.31476443,((Tegeolepis_clarki:21.13035857,Howqualepis_rostridens:1):2.70484133,((Gogosardina_coatesi:1,(Mimipiscis_bartrami:3.734625685,Mimipiscis_toombsi:1):4.170984044):5.674437335,((Moythomasia_lineata:1,Moythomasia_durgaringa:9.20323433):1.472730931,(Stegotrachelus_finlayi:1,(Limnomis_delaneyi:1,(Wendyichthys_dicksoni:27.94571503,(Kentuckia_deani:1,((Mesopoma_planti:1.695124656,Mesopoma_carricki:1):9.506044823,((Birgeria_stensioei:1,(Chondrosteus_acipenseroides:1,(Saurichthys_dawaziensis:1,((Protopsephurus_liui:1,Polyodontidae_Polyodon_spathula:126.9992057):11.90079634,(Acipenseridae_Acipenser_fulvescens:38.579335,(Acipenseridae_Scaphirhynchus_platorynchus:19.375515,Acipenseridae_Scaphirhynchus_albus:19.375515):19.20382):100.320667):24.87454907):37.09986707):51.9899717):97.23561116,(Boreosomus:74.637327,(((Fukangichthys_longidorsalis:7.809555612,(Scanilepis_dubia:44.85373983,Evenkia_eunotoptera:1):1):1,Australosomus:3.065640459):70.37221926,(Perleidus_altolepis:84.10311852,(Luganoia_lepidosteoides:90.6461834,(((Macrosemius_fourneti:95.17977248,((Semionotus_elegans:1,Lepisosteidae_Lepisosteus_osseus:201.7436646):45.7727441,Kyphosichthys_grandei:1):1):19.38359235,(Watsonulus_eugnathoides:1,(Amblysemius:1,(Pachyamia_latimaxillaris:6.867500907,(Tomognathus_mordax:1,Amiidae_Amia_calva:101.4341123):1):48.96911912):101.6191371):14.8776325):54.6,(Pachycormus:103.4223934,(Crossognathus_danubiensis:176.5654319,(((Anaethalion_angustus:1,Anaethalion_knorri:4.041460688):45.79057462,((Megalopidae_Megalops_atlanticus:133.565966,Elopidae_Elops_saurus:133.565966):62.634035,(Albulidae_Albula_vulpes:150.800001,((Notacanthidae_Notacanthus_chemnitzii:50.669287,(Halosauridae_Halosauropsis_macrochir:40.366211,Halosauridae_Aldrovandia_affinis:40.366211):10.303076):50.330714,(((Eurypharyngidae_Eurypharynx_pelecanoides:25.866364,Saccopharyngidae_Saccopharynx_ampullaceus:25.866364):44.322494,(Nemichthyidae_Nemichthys_scolopaceus:58.739485,Anguillidae_Anguilla_rostrata:58.739485):11.449373):9.198895,(Serrivomeridae_Serrivomer_beanii:76.053487,(Congridae_Conger_oceanicus:65.416147,(Muraenesocidae_Muraenesox_cinereus:57.193624,Ophichthidae_Myrichthys_maculosus:57.193624):8.222523):10.63734):3.334266):21.612248):49.8):45.4):1):86.1,((Lycoptera_davidi:97.56604404,(Paralycoptera_wui:104.1571959,(Xixiaichthys_tongxinens:100.4875226,(((Eohiodon_woodruffi:1,Eohiodon_rosei:2.852091247):1,(Hiodon_consteniorum:1,(Hiodontidae_Hiodon_tergisus:9.520291,Hiodontidae_Hiodon_alosoides:9.520291):26.4070695):21.39168039):169.7809601,(Chauliopareion_mahengeense:117.4598073,(Pantodontidae_Pantodon_buchholzi:163.100001,((((Brychaetus_muelleri:1,Phareodus_encaustus:3.831223781):29.27554957,Arapaimidae_Arapaima_gigas:79.3):25.549112,Osteoglossidae_Osteoglossum_bicirrhosum:104.849112):43.475578,(Notopteridae_Xenomystus_nigri:120.300002,Gymnarchidae_Gymnarchus_niloticus:120.300002):28.024688):14.775311):1):63):1):1):1):43.7,((((Diplomystus_brevissimus:45.94669526,Sorbinichthys_africanus:1):91.14448697,(Denticipitidae_Denticeps_clupeoides:188.900001,(Chirocentridae_Chirocentrus_dorab:87.096454,((Engraulidae_Coilia_nasus:47.06527,Engraulidae_Engraulis_mordax_eurystole:47.06527):33.347234,((Pristigasteridae_Pellona_flavipinnis:27.450803,Pristigasteridae_Ilisha_elongata:27.450803):46.430359,(Clupeidae_Alosa_pseudoharengus:54.138864,Clupeidae_Dorosoma_cepedianum:54.138864):19.742298):6.531342):6.68395):101.803547):1):40.3,((Alepocephalidae_Talismania_bifurcata:53.266521,((Alepocephalidae_Bathylaco_nigricans:33.802485,Alepocephalidae_Alepocephalus_tenebrosus:33.802485):10.988666,((Alepocephalidae_Rouleina_attrita:22.275085,Alepocephalidae_Xenodermichthys_copei:22.275085):19.924916,Platytroctidae_Sagamichthys_abei:42.200001):2.59115):8.47537):166.430015,((Gonorynchidae_Gonorynchus_abbreviatus:175.900001,(Mahengichthys_singidaensis:99.524047,Chanidae_Chanos_chanos:147.100001):28.8):22.7,((((Gyrinocheilidae_Gyrinocheilus_sp:70.111694,(Catostomidae_Hypentelium_nigricans:23.594381,(Catostomidae_Carpiodes_carpio:16.355089,Catostomidae_Ictiobus_bubalus:16.355089):7.239292):46.517313):8.688307,Cobitidae_Cobitis_taenia:78.800001):20.5,(Cyprinidae_Danio_rerio:63.300001,(((Cyprinidae_Zacco_sieboldii_platypus:12.727958,Cyprinidae_Opsariichthys_uncirostris_bidens:12.727958):14.610086,((Cyprinidae_Xenocypris_argentea:12.904177,Cyprinidae_Hypophthalmichthys_molitrix:12.904177):2.917503,(Cyprinidae_Luciobrama_macrocephalus:13.439618,(Cyprinidae_Squaliobarbus_curriculus:11.472717,Cyprinidae_Mylopharyngodon_piceus:11.472717):1.966901):2.382062):11.516364):12.401957,(Cyprinidae_Tanakia_lanceolata_himantegus:35.004261,(Cyprinidae_Notemigonus_crysoleucas:22.933404,(Cyprinidae_Semotilus_atromaculatus:21.325215,((Cyprinidae_Campostoma_oligolepis:12.800846,Cyprinidae_Rhinichthys_cataractae:12.800846):4.408707,(Cyprinidae_Phenacobius_uranops:12.822623,(Cyprinidae_Pimephales_promelas_notatus:9.460336,(Cyprinidae_Luxilus_coccogenis:6.738861,Cyprinidae_Notropis_asperifrons:6.738861):2.721475):3.362287):4.38693):4.115662):1.608189):12.070857):4.73574):23.56):36):73.6,((Gymnotidae_Electrophorus_electricus:63.909447,Gymnotidae_Gymnotus_sp:63.909447):83.924067,(((Distichodontidae_Distichodus_maculatus:103.604365,Citharinidae_Citharinus_congicus:103.604365):11.170138,(Alestidae_Alestes_baremoze:106.666979,((Parodontidae_Parodon_nasus:68.800001,Hemiodontidae_Hemiodus_immaculatus:68.800001):22.415197,((Bryconidae_Brycon_pesu:66.003418,(Gasteropelecidae_Thoracocharax_stellatus:27.746828,Gasteropelecidae_Gasteropelecus_sternicla:27.746828):38.25659):1.227196,Characidae_Astyanax_mexicanus:67.230614):23.984584):15.451781):8.107524):22.236489,((Nematogenyidae_Nematogenys_inermis:110.648758,(Trichomycteridae_Trichomycterus_sp:105.886466,((Loricariidae_Loricaria_simillima:70.313425,Astroblepidae_Astroblepus_sp:70.313425):28.122359,(Callichthyidae_Callichthys_callichthys:69.200001,Callichthyidae_Corydoras_trilineatus:69.200001):29.235783):7.450682):4.762292):5.151243,(Diplomystidae_Diplomystes_nahuelbutaensis:106.203801,((Clariidae_Clarias_batrachus:47.60069,Heteropneustidae_Heteropneustes_fossilis:47.60069):38.718856,(((Sisoridae_Bagarius_yarrelli:55.522058,(Amblycipitidae_Liobagrus_aequilabris:48.027606,Akysidae_Akysis_sp:48.027606):7.494452):21.358911,(Schilbeidae_Pseudeutropius_brachypopterus:74.448774,(Bagridae_Mystus_bocourti:36.077943,Bagridae_Bagrus_ubangensis:36.077943):38.370831):2.432195):8.408138,(((Chacidae_Chaca_sp:72.429482,Cetopsidae_Cetopsis_coecutiens:72.429482):10.714797,(Cranoglanididae_Cranoglanis_bouderius:68.700001,Ictaluridae_Ictalurus_punctatus:68.700001):14.444278):1.039619,(Pangasiidae_Pangasianodon_hypophthalmus:82.158061,((Amphiliidae_Amphilius_jacksonii:69.199757,(Malapteruridae_Malapterurus_beninensis:64.740321,Mochokidae_Synodontis_batesii:64.740321):4.459436):10.993535,((Plotosidae_Plotosus_lineatus:73.039645,Siluridae_Hemisilurus_moolenburghi:73.039645):5.503862,(Auchenipteridae_Ageneiosus_atronasus:51.101654,Doradidae_Anduzedoras_oxyrhynchus:51.101654):27.441853):1.649785):1.964769):2.025837):1.105209):1.030439):19.884255):9.5962):21.210991):10.822522):25.066487):25.7):21.096535):10.503465):20.4,(Orthogonikleithrus_francogalliensis:77.91987599,(((Bathylagidae_Bathylagus_euryops:70.542512,(Microstomatidae_Nansenia_longicauda_ardesiaca:51.394,(Opisthoproctidae_Macropinna_microstoma:33.11268,Argentinidae_Argentina_sialis_silus:33.11268):18.28132):19.148512):89.057489,((Galaxiidae_Neochanna_burrowsius:34.505049,Galaxiidae_Galaxias_maculatus:34.505049):111.121934,(((Umbridae_Novumbra_hubbsi:55.762491,Umbridae_Umbra_limi:55.762491):23.63751,(Esocidae_Esox_lucius:31.366195,Esocidae_Esox_americanus:31.366195):48.033806):25.064928,(Salmonidae_Coregonus_clupeaformis:35.300001,(Salmonidae_Thymallus_brevirostris:32.456522,(Salmonidae_Oncorhynchus_nerka_mykiss:19.929533,(Salmonidae_Salvelinus_alpinus:16.737138,Salmonidae_Salmo_salar:16.737138):3.192395):12.526989):2.843479):69.164928):41.162054):13.973018):55.253787,(((Retropinnidae_Retropinna_semoni:73.700001,(((Osmeridae_Osmerus_mordax:10.332899,Osmeridae_Thaleichthys_pacificus:10.332899):5.872101,(Osmeridae_Mallotus_villosus:14.961758,Osmeridae_Hypomesus_pretiosus:14.961758):1.243242):18.133836,(Plecoglossidae_Plecoglossus_altivelis:28.70318,Salangidae_Neosalangichthys_ishikawae:28.70318):5.635656):39.361165):55.7,((Diplophidae_Diplophos_taenia:77.330737,((Gonostomatidae_Bonapartia_pedaliota:54.297445,Gonostomatidae_Margrethia_obtusirostra:54.297445):20.014374,(Gonostomatidae_Gonostoma_elongatum:69.948163,Gonostomatidae_Cyclothone_microdon:69.948163):4.363656):3.018918):6.469264,(Phosichthyidae_Pollichthys_mauli:77.722827,((Sternoptychidae_Argyropelecus_gigas:25.230481,Sternoptychidae_Maurolicus_weitzmani:25.230481):47.329876,(Stomiidae_Chauliodus_macouni_danae:63.126299,(Stomiidae_Stomias_boa:36.381906,(Stomiidae_Chirostomias_pliopterus:31.578039,((Stomiidae_Melanostomias_margaritifer:21.790927,Stomiidae_Leptostomias_longibarba:21.790927):6.240428,((Stomiidae_Photonectes_margarita:19.053467,Stomiidae_Tactostoma_macropus:19.053467):7.977888,(Stomiidae_Malacosteus_niger:25.031355,(Stomiidae_Eustomias_polyaster:24.031355,(Stomiidae_Aristostomias_scintillans:23.031355,Stomiidae_Bathophilus_flemingi_pawneei:23.031355):1):1):2):1):3.546684):4.803867):26.744393):9.434058):5.16247):6.077174):45.6):81.9,((Ateleopodidae_Ateleopus_japonicus:8.089622,Ateleopodidae_Ijimaia_antillarum:8.089622):184.210379,(((Synodontidae_Trachinocephalus_myops:34.928634,Synodontidae_Synodus_foetens:34.928634):78.971367,((Paraulopidae_Paraulopus_oblongus:101.985288,(Synodontidae_Saurida_gracilis:50.257509,Synodontidae_Harpadon_microchir:50.257509):51.727779):8.326796,(((Aulopidae_Aulopus_filamentosus:47.160387,Bathysauridae_Bathysaurus_ferox:47.160387):10.843902,(Ipnopidae_Ipnops_murrayi:13.82,Ipnopidae_Ipnops_agassizi:13.82):44.184289):23.635948,((Ipnopidae_Bathypterois_atricolor:69.328167,(Giganturidae_Gigantura_indica:13.525058,Giganturidae_Gigantura_chuni:13.525058):55.803109):6.462647,(Scopelarchidae_Benthalbella_macropinna:62.490696,(Chlorophthalmidae_Chlorophthalmus_agassizi:61.13635,(Sudidae_Sudis_atrox:55.026295,((Alepisauridae_Anotopterus_pharao:45.661943,(Alepisauridae_Omosudis_lowii:36.284216,Alepisauridae_Alepisaurus_ferox:36.284216):9.377727):8.238175,(Paralepididae_Stemonosudis_intermedia_macrura:50.122687,(Evermannellidae_Evermannella_balbo:46.686813,Paralepididae_Paralepis_coregonoides:46.686813):3.435874):3.777431):1.126177):6.110055):1.354346):13.300118):5.849423):28.671847):3.587917):68.9,(((Neoscopelidae_Neoscopelus_macrolepidotus:42.408365,Neoscopelidae_Scopelengys_tristis:42.408365):31.191636,((Myctophidae_Lampadena_speculigera:39.81673,Myctophidae_Lampanyctus_macdonaldi:39.81673):11.872,(Myctophidae_Benthosema_glaciale:28.30241,Myctophidae_Myctophum_punctatum:28.30241):23.38632):21.911271):99.5,(((Lamprididae_Lampris_guttatus:65.552505,(Regalecidae_Regalecus_russelii:37.682943,Trachipteridae_Trachipterus_trachypterus:37.682943):27.869562):84.747496,(((Mcconichthys_longipinnis:1,(Aphredoderidae_Aphredoderus_sayanus:41.500001,Amblyopsidae_Chologaster_cornuta:41.500001):25.08446898):1,(Lateopisciculus_turrifumosus:1,(Percopsidae_Percopsis_omiscomaycus:14.617149,Percopsidae_Percopsis_transmontana:14.617149):45.43799441):7.529326573):67.40586202,(Zeidae_Zeus_faber:107.100001,(Stylephoridae_Stylephorus_chordatus:78.800001,(Merlucciidae_Merluccius_productus:45.655086,(((Macrouridae_Gadomus_dispar:9.947595,Macrouridae_Bathygadus_favosus:9.947595):28.939269,(Steindachneriidae_Steindachneria_argentea:35.929071,((Macrouridae_Malacocephalus_laevis:17.761718,Macrouridae_Trachonurus_sulcatus:17.761718):10.126413,(Macrouridae_Coelorinchus_caribbaeus:21.883517,Macrouridae_Coryphaenoides_armatus:21.883517):6.004614):8.04094):2.957793):2.613137,((Moridae_Laemonema_goodebeanorum:25.08833,(Moridae_Halargyreus_johnsonii:16.444614,(Moridae_Lepidion_ensiferus:5.35693,Moridae_Antimora_rostrata:5.35693):11.087684):8.643716):13.1716,((Phycidae_Urophycis_tenuis:5.240351,Phycidae_Urophycis_chuss:5.240351):28.607969,(Lotidae_Gaidropsarus_ensis:29.800286,(Lotidae_Lota_lota:23.935071,(Gadidae_Melanogrammus_aeglefinus:5.86839,Gadidae_Gadus_morhua:5.86839):18.066681):5.865215):4.048034):4.41161):3.240071):4.155085):33.144915):28.3):27.890331):15.309669):11.08283,(Polymixiidae_Polymixia_japonica:154.800001,(((Diretmidae_Diretmus_argenteus:53.99855,(Monocentridae_Monocentris_japonica:33.600001,((Trachichthyidae_Hoplostethus_occidentalis_atlanticus:24.361165,Trachichthyidae_Gephyroberyx_darwinii:24.361165):7.919156,(Anoplogastridae_Anoplogaster_cornuta:31.22776,Trachichthyidae_Paratrachichthys_sajademalensis:31.22776):1.052561):1.31968):20.398549):71.275924,((Berycidae_Beryx_decadactylus:70.612394,(Melamphaidae_Poromitra_crassiceps:39.138751,(Melamphaidae_Scopelogadus_beanii:32.603956,Melamphaidae_Melamphaes_suborbitalis:32.603956):6.534795):31.473643):40.295775,(((Rondeletiidae_Rondeletia_bicolor:18.616456,Rondeletiidae_Rondeletia_loricata:18.616456):36.662305,Barbourisiidae_Barbourisia_rufa:55.278761):34.605685,(Cetomimidae_Cetostoma_regani:55.492218,(Cetomimidae_Cetomimus_craneae:10.537863,Cetomimidae_Cetomimus_compuctus:10.537863):44.954355):34.392228):21.023723):14.366305):21.189147,((Holocentridae_Sargocentron_diadema:25.43456,Holocentridae_Holocentrus_rufus:25.43456):119.565441,((Ophidiidae_Brotula_multibarbata:66.227334,(Ophidiidae_Lepophidium_profundorum:23.036156,(Ophidiidae_Genypterus_blacodes:21.590629,Ophidiidae_Ophidion_holbrookii:21.590629):1.445527):43.191178):66.572667,((Batrachoididae_Porichthys_notatus:39.800001,Batrachoididae_Opsanus_tau:39.800001):87,(((Kurtidae_Kurtus_indicus:80.300001,Apogonidae_Apogon_campbelli:80.300001):22,(Eleotridae_Eleotris_acanthopoma_pisonis:64.430477,(Gobiidae_Gobiosoma_bosc:45.630567,Microdesmidae_Microdesmus_longipinnis:45.630567):18.79991):37.869524):19.3,((((Dactylopteridae_Dactylopterus_volitans:68.121568,(Aulostomidae_Aulostomus_maculatus:3.200001,Aulostomidae_Aulostomus_chinensis:3.200001):64.921567):6.178433,((Fistulariidae_Fistularia_petimba:65.463647,Mullidae_Mullus_auratus:65.463647):6.26906,(Callionymidae_Callionymus_sp_bairdi:69.154942,Syngnathidae_Syngnathus_fuscus:69.154942):2.577765):2.567294):20.27,(Chiasmodontidae_Chiasmodon_sp:43.900001,((Centrolophidae_Icichthys_lockingtoni:34.370774,Pomatomidae_Pomatomus_saltatrix:34.370774):6.934458,(((Ariommatidae_Ariomma_bondi:25.644121,Nomeidae_Psenes_cyanophrys:25.644121):5.370208,Scombridae_Auxis_rochei:31.014329):6.588438,((Stromateidae_Peprilus_paru:35.602767,(Gempylidae_Paradiplospinus_gracilis:31.499638,Bramidae_Brama_brama:31.499638):4.103129):1,(Icosteidae_Icosteus_aenigmaticus:33.972252,Trichiuridae_Trichiurus_lepturus:33.972252):2.630515):1):3.702465):2.594769):50.67):21.916687,((((((Synbranchidae_Monopterus_albus:69.146846,Indostomidae_Indostomus_paradoxus:69.146846):4.548479,(Mastacembelidae_Macrognathus_siamensis:18.057555,Mastacembelidae_Mastacembelus_erythrotaenia:18.057555):55.63777):7.004676,((Anabantidae_Ctenopoma_acutirostre_kingsleyae:62.782323,(Helostomatidae_Helostoma_temminkii:59.687826,Osphronemidae_Trichopodus_pectoralis:59.687826):3.094497):7.86905,(Channidae_Channa_striata:67.428514,Nandidae_Nandus_nandus:67.428514):3.222859):10.048628):15.7,((Nematistiidae_Nematistius_pectoralis:60.067614,((Coryphaenidae_Coryphaena_hippurus:19.124214,Rachycentridae_Rachycentron_canadum:19.124214):12.775787,(Echeneidae_Remora_osteochir_australis:10.178122,Echeneidae_Echeneis_naucrates:10.178122):21.721879):28.167613):9.732387,((Sphyraenidae_Sphyraena_sphyraena:65.900885,(Menidae_Mene_maculata:60.339531,Polynemidae_Polydactylus_octonemus:60.339531):5.561354):2.899116,((((Leptobramidae_Leptobrama_muelleri:46.58559,Toxotidae_Toxotes_jaculatrix:46.58559):5.096327,(Xiphiidae_Xiphias_gladius:27.833842,Istiophoridae_Istiophorus_platypterus:27.833842):23.848075):5.298938,Carangidae_Trachinotus_falcatus:56.980855):4.861848,((Eolates_gracilis:1,(Centropomidae_Psammoperca_waigiensis:33.068144,(Centropomidae_Lates_calcarifer:13.595548,Centropomidae_Lates_microlepis:13.595548):19.472596):22.60237332):5.172185683,(Heteronectes_chaneti:4.830054488,(Amphistium_paradoxum:6.927046545,(Psettodidae_Psettodes_erumei:57.842703,(Citharidae_Lepidoblepharon_ophthalmolepis:53.600001,((Cynoglossidae_Cynoglossus_interruptus:42.400002,(Soleidae_Solea_solea:24.944331,Soleidae_Soleichthys_heterorhinos:24.944331):17.455671):9.674607,((Scophthalmidae_Scophthalmus_aquosus:23.305906,Scophthalmidae_Lepidorhombus_boscii:23.305906):26.973285,((Paralichthyidae_Paralichthys_dentatus:40.749719,Bothidae_Bothus_lunatus:40.749719):4.387561,(Paralichthyidae_Pseudorhombus_pentophthalmus:21.404631,(((Pleuronectidae_Glyptocephalus_zachirus:5.096079,Pleuronectidae_Microstomus_pacificus:5.096079):2.538752,(Pleuronectidae_Lyopsetta_exilis:6.085211,Pleuronectidae_Hippoglossus_hippoglossus:6.085211):1.54962):2.215451,(Pleuronectidae_Limanda_limanda:8.798272,((Pleuronectidae_Platichthys_stellatus:3.717184,Pleuronectidae_Pleuronectes_platessa:3.717184):3.081088,(Pleuronectidae_Lepidopsetta_bilineata:4.798272,Pleuronectidae_Psettichthys_melanostictus:4.798272):2):2):1.05201):11.554349):23.732649):5.141911):1.795418):1.525392):4.242702):1):1):1):1):6.957298):1):26.6):12.043384,(((Polycentridae_Polycentrus_schomburgkii:94.214904,(Pholidichthyidae_Pholidichthys_leucotaenia:88.700001,Cichlidae_Cichla_temensis:88.700001):5.514903):1,(((Atherinopsidae_Atherinopsis_californiensis:48.715388,Atherinopsidae_Menidia_menidia:48.715388):22.225577,(Isonidae_Iso_sp:49.317292,(Atherinidae_Atherinomorus_stipes:43.78646,(Melanotaeniidae_Melanotaenia_splendida:39.870052,Pseudomugilidae_Pseudomugil_gertrudae:39.870052):3.916408):5.530832):21.623673):6.46052,(((Poeciliidae_Poecilia_latipinna_reticulata:14.292152,(Poeciliidae_Heterandria_formosa:11.366823,(Poeciliidae_Gambusia_affinis:5.736451,Poeciliidae_Belonesox_belizanus:5.736451):5.630372):2.925329):30.13151,(Cyprinodontidae_Cyprinodon_variegatus:40.469994,Fundulidae_Fundulus_parvipinnis:40.469994):3.953668):31.977823,(Adrianichthyidae_Oryzias_latipes:71.878571,((Zenarchopteridae_Dermogenys_collettei:29.883878,((Belonidae_Tylosurus_crocodilus:6.568056,Belonidae_Ablennes_hians:6.568056):17.429484,(Scomberesocidae_Cololabis_saira:2.646712,Scomberesocidae_Scomberesox_saurus:2.646712):21.350828):5.886338):17.178962,(Hemiramphidae_Hyporhamphus_affinis:36.892311,((Hemiramphidae_Hemiramphus_brasiliensis:13.391766,Hemiramphidae_Oxyporhamphus_micropterus:13.391766):10.259915,((Exocoetidae_Cypselurus_callopterus:6.413654,Exocoetidae_Exocoetus_monocirrhus:6.413654):4.605967,Exocoetidae_Cheilopogon_pinnatibarbatus:11.019621):12.63206):13.24063):10.170529):24.815731):4.522914):1):17.813419):1.999954,((Pseudochromidae_Halidesmus_scapularis:84.850287,Pomacentridae_Pomacentrus_brachialis:84.850287):8.187103,(Embiotocidae_Cymatogaster_aggregata:91.529056,(Mugilidae_Mugil_cephalus:89.757655,(Plesiopidae_Plesiops_coeruleolineatus:86.977996,(Opistognathidae_Opistognathus_maxillosus:79.196597,(((Gobiesocidae_Lepadichthys_lineatus:37.560081,Gobiesocidae_Gobiesox_maeandricus:37.560081):35.985217,Tripterygiidae_Enneanectes_altivelis:73.545298):3.122761,((Clinidae_Clinus_cottoides:50.741024,(Dactyloscopidae_Platygillellus_rubrocinctus:45.641062,Chaenopsidae_Chaenopsis_alepidota:45.641062):5.099962):17.520576,(Blenniidae_Stanulus_seychellensis:31.042358,Blenniidae_Blenniella_cyanostigma:31.042358):37.219242):8.406459):2.528538):7.781399):2.779659):1.771401):1.508334):4.177468):11.228527):3.450865,(Gerreidae_Eucinostomus_argenteus:108.000002,(((Labridae_Lachnolaimus_maximus:59.822938,Odacidae_Haletta_semifasciata:59.822938):16.826114,Scaridae_Scarus_globiceps:76.649052):30.35095,(((Uranoscopidae_Uranoscopus_sulphureus:83.996275,(Ammodytidae_Ammodytes_hexapterus:78.584442,Pinguipedidae_Parapercis_punctulata:78.584442):5.411833):11.903726,(((Acropomatidae_Acropoma_japonicum:90.360051,((Percophidae_Acanthaphritis_unoorum:66.16617,Creediidae_Limnichthys_sp:66.16617):18.796708,(Glaucosomatidae_Glaucosoma_buergeri:66.617288,Pempheridae_Pempheris_schomburgkii:66.617288):18.34559):5.397173):2.814411,(((Oplegnathidae_Oplegnathus_punctatus:61.206964,Kuhliidae_Kuhlia_rupestris:61.206964):7.298332,Kyphosidae_Kyphosus_sectatrix:68.505296):16.157201,(Percichthyidae_Percichthys_trucha:69.999181,((Cirrhitidae_Cirrhitichthys_falco:66.467051,Cheilodactylidae_Cheilodactylus_fasciatus:66.467051):2.53213,(Enoplosidae_Enoplosus_armatus:63.04721,Centrarchidae_Acantharchus_pomotis:63.04721):5.951971):1):14.663316):8.511965):1.725539,(Serranidae_Pseudogramma_polyacantha:82.200001,(Percidae_Perca_fluviatilis:78.577195,((Bovichtidae_Bovichtus_diacanthus:63.021097,(Nototheniidae_Notothenia_coriiceps:9.671082,(Harpagiferidae_Harpagifer_antarcticus:8.390321,(Bathydraconidae_Gymnodraco_acuticeps:7.390321,Channichthyidae_Chionodraco_rastrospinosus:7.390321):1):1.280761):53.350015):12.936917,(((Peristediidae_Peristedion_ecuadorense:54.532565,Triglidae_Prionotus_carolinus:54.532565):11.953546,(Synanceiidae_Synanceia_verrucosa:62.510934,Scorpaenidae_Scorpaenodes_guamensis:62.510934):3.975177):6.621302,(Platycephalidae_Platycephalus_indicus:70.795272,(Anoplopomatidae_Anoplopoma_fimbria:46.504594,((Bathymasteridae_Rathbunella_hypoplecta:22.798552,(Stichaeidae_Cebidichthys_violaceus:15.58542,(Zoarcidae_Zoarces_americanus_viviparus:12.493576,(Anarhichadidae_Anarrhichthys_ocellatus:9.933504,(Zaproridae_Zaprora_silenus:8.933504,Cryptacanthodidae_Cryptacanthodes_maculatus:8.933504):1):2.560072):3.091844):7.213132):19.028758,(((Aulorhynchidae_Aulorhynchus_flavidus:25.280385,Hypoptychidae_Hypoptychus_dybowskii:25.280385):1.019618,((Gasterosteidae_Culaea_inconstans:19.844899,(Gasterosteidae_Apeltes_quadracus:17.136644,Gasterosteidae_Spinachia_spinachia:17.136644):2.708255):1.782129,(Gasterosteidae_Pungitius_pungitius:16.827665,Gasterosteidae_Gasterosteus_aculeatus:16.827665):4.799363):4.672975):13.53479,(Hexagrammidae_Hexagrammos_decagrammus:27.818969,((Cyclopteridae_Cyclopterus_lumpus:20.762394,Liparidae_Liparis_pulchellus:20.762394):4.037609,(Agonidae_Hypsagonus_quadricornis:20.371382,(Cottidae_Cottus_carolinae:18.19359,Psychrolutidae_Psychrolutes_phrictus:18.19359):2.177792):4.428621):3.018966):12.015824):1.992517):4.677284):24.290678):2.312141):2.850601):2.619181):3.622806):12.7):1):10.100001,((Drepaneidae_Drepane_punctata:67.658011,Ephippidae_Chaetodipterus_faber:67.658011):37.341991,(((Lobotidae_Lobotes_pacificus_surinamensis:86.438004,Sciaenidae_Menticirrhus_undulatus_littoralis:86.438004):5.761997,((Monodactylidae_Monodactylus_sebae:70.333247,(Avitoluvarus_eocaenicus:21.57030023,(Zanclidae_Zanclus_cornutus:55.859022,Acanthuridae_Acanthurus_triostegus:55.859022):4.740979):9.733246):15.366754,((Pomacanthidae_Pomacanthus_semicirculatus:54.434118,(Leiognathidae_Leiognathus_equulus:32.700001,Chaetodontidae_Chelmon_rostratus:32.700001):21.734117):17.438962,(Emmelichthyidae_Erythrocles_schlegelii:69.871718,(Malacanthidae_Malacanthus_plumieri:68.303733,(Haemulidae_Haemulon_aurolineatum:64.600001,Lutjanidae_Lutjanus_griseus:64.600001):3.703732):1.567985):2.001362):13.826921):6.5):11.800001,((Sillaginidae_Sillago_sihama:85.853361,(Nemipteridae_Pentapodus_caninus:80.270573,(Lethrinidae_Lethrinus_erythropterus:74.370539,Sparidae_Stenotomus_chrysops:74.370539):5.900034):5.582788):17.146641,(Siganidae_Siganus_spinus:102.000002,((Scatophagidae_Scatophagus_argus:75.860371,Priacanthidae_Heteropriacanthus_cruentatus:75.860371):25.139631,((Caproidae_Antigonia_rubescens:10.905674,Caproidae_Antigonia_capros:10.905674):89.094328,(((Lophiidae_Lophiodes_reticulatus:22.462185,Lophiidae_Lophius_gastrophysus:22.462185):42.032119,((Antennariidae_Histrio_histrio:12.333102,Antennariidae_Antennatus_coccineus:12.333102):33.466899,(Chaunacidae_Chaunax_suttkusi:38.499563,(Gigantactinidae_Gigantactis_sp:32.911618,((Ceratiidae_Cryptopsaras_couesii:20.548708,Ceratiidae_Ceratias_holboelli:20.548708):10.416157,((Himantolophidae_Himantolophus_albinares_sagamius:14.456802,Melanocetidae_Melanocetus_murrayi:14.456802):9.124159,(Oneirodidae_Oneirodes_macrosteus:10.164357,Oneirodidae_Dolopichthys_sp:10.164357):13.416604):7.383904):1.946753):5.587945):7.300438):18.694303):34.505698,(((Prohollardia_avita:37.69167886,(Protacanthodes_nimesensis:1,Triacanthidae_Triacanthus_biaculeatus:44.91051232):19.58245868):10.123893,(Aracanidae_Aracana_aurita:54.800001,(Ostraciidae_Ostracion_cubicus:51.000002,Ostraciidae_Rhinesomus_triqueter:51.000002):3.799999):19.816863):3.183137,(((Diodontidae_Diodon_holocanthus:12.198387,Diodontidae_Chilomycterus_schoepfii:12.198387):43.701614,(Tetraodontidae_Canthigaster_bennetti:24.22571,Tetraodontidae_Tetraodon_miurus:24.22571):31.674291):17.334859,((Molidae_Ranzania_laevis:22,Molidae_Mola_mola:22):46.61027,((Monacanthidae_Aluterus_scriptus:27.823604,Monacanthidae_Stephanolepis_hispidus:27.823604):15.976396,(Balistidae_Xanthichthys_ringens:20.7,(Balistidae_Balistes_vetula:19.7,Balistidae_Sufflamen_fraenatum:19.7):1):23.1):24.81027):4.62459):4.565141):21.200001):1):1):1):1):1):1):1):1):1):3.894248):4.592438):5.113313):5.2):6):12.2):1.46362):8.33638):6.58283):11.71717):9.7):9.5):19):3.553787):17.946213):17.8):23.2):9.5):1):1):37.2):1):1):1):1):23.6):1):5.033227888):1):9.339795192):20.33557677):1):1):1):1):4.191401154):1):47);

((Guiyu_oneiros:1,(Onychodus_jandemarrai:32.6988609,((Diplocercides:1,(Latimeriidae_Latimeria_chalumnae:329.4904831,Rhabdoderma:1):40.93481729):42.09032658,((Neoceratodontidae_Neoceratodus_forsteri:279.7982796,Lepidosirenidae_Lepidosiren_paradoxa:279.7982796):128.2017204,((Osteolepis_macrolepidotus:1,Gyroptychius_milleri:4.065028798):1,Eusthenopterus_foordi:21.15579677):10.16807474):4.515627):1):13.21995619):16.26441881,((Cheirolepis_trailli:1,Cheirolepis_schultzei:6.099446827):6.247393033,((Polypteridae_Erpetoichthys_calabaricus:29.200001,Polypteridae_Polypterus_senegalus:29.200001):365.800001,(Osorioichthys_marginis:21.80509035,((Tegeolepis_clarki:27.50334028,Howqualepis_rostridens:1):1,((Gogosardina_coatesi:1,(Mimipiscis_bartrami:1,Mimipiscis_toombsi:2.202331125):1.34324101):5.392598058,((Moythomasia_lineata:1,Moythomasia_durgaringa:2.474535296):2.163235848,(Stegotrachelus_finlayi:1,(Limnomis_delaneyi:1,(Wendyichthys_dicksoni:25.2719125,(Kentuckia_deani:1,((Mesopoma_planti:3.179735075,Mesopoma_carricki:1):5.509251683,((Birgeria_stensioei:1,(Chondrosteus_acipenseroides:1,(Saurichthys_dawaziensis:1,((Protopsephurus_liui:1,Polyodontidae_Polyodon_spathula:127.1252082):11.77479377,(Acipenseridae_Acipenser_fulvescens:38.579335,(Acipenseridae_Scaphirhynchus_platorynchus:19.375515,Acipenseridae_Scaphirhynchus_albus:19.375515):19.20382):100.320667):24.77355398):37.13321446):51.54106699):97.75216358,(Boreosomus:75.41922656,(((Perleidus_altolepis:82.79030333,(Luganoia_lepidosteoides:91.68883123,(((Macrosemius_fourneti:93.31587985,((Semionotus_elegans:1,Lepisosteidae_Lepisosteus_osseus:201.4985787):44.56161738,Kyphosichthys_grandei:1):1):20.83980489,(Watsonulus_eugnathoides:1,(Amblysemius:1,(Pachyamia_latimaxillaris:1,(Tomognathus_mordax:1,Amiidae_Amia_calva:98.35893882):1.873096651):46.39904162):106.3316492):14.93727474):54.6,(Pachycormus:104.411953,(Crossognathus_danubiensis:176.5146196,(((Anaethalion_angustus:4.12129226,Anaethalion_knorri:1):44.5112777,((Megalopidae_Megalops_atlanticus:133.565966,Elopidae_Elops_saurus:133.565966):62.634035,(Albulidae_Albula_vulpes:150.800001,((Notacanthidae_Notacanthus_chemnitzii:50.669287,(Halosauridae_Halosauropsis_macrochir:40.366211,Halosauridae_Aldrovandia_affinis:40.366211):10.303076):50.330714,(((Eurypharyngidae_Eurypharynx_pelecanoides:25.866364,Saccopharyngidae_Saccopharynx_ampullaceus:25.866364):44.322494,(Nemichthyidae_Nemichthys_scolopaceus:58.739485,Anguillidae_Anguilla_rostrata:58.739485):11.449373):9.198895,(Serrivomeridae_Serrivomer_beanii:76.053487,(Congridae_Conger_oceanicus:65.416147,(Muraenesocidae_Muraenesox_cinereus:57.193624,Ophichthidae_Myrichthys_maculosus:57.193624):8.222523):10.63734):3.334266):21.612248):49.8):45.4):1):86.1,((Lycoptera_davidi:97.83979179,(Paralycoptera_wui:105.5764004,(Xixiaichthys_tongxinens:100.4992951,(((Eohiodon_woodruffi:1,Eohiodon_rosei:5.933841975):1,(Hiodon_consteniorum:1,(Hiodontidae_Hiodon_tergisus:9.520291,Hiodontidae_Hiodon_alosoides:9.520291):28.37195866):19.40302272):169.8047286,(Chauliopareion_mahengeense:118.1438275,(Pantodontidae_Pantodon_buchholzi:163.100001,((((Brychaetus_muelleri:1,Phareodus_encaustus:7.24982161):1,Osteoglossidae_Osteoglossum_bicirrhosum:53.90375514):50.94535686,Arapaimidae_Arapaima_gigas:104.849112):43.475578,(Notopteridae_Xenomystus_nigri:120.300002,Gymnarchidae_Gymnarchus_niloticus:120.300002):28.024688):14.775311):1):63):1):1):1):43.7,((((Diplomystus_brevissimus:45.24308013,Sorbinichthys_africanus:1):92.52113582,(Denticipitidae_Denticeps_clupeoides:188.900001,(Chirocentridae_Chirocentrus_dorab:87.096454,((Engraulidae_Coilia_nasus:47.06527,Engraulidae_Engraulis_mordax_eurystole:47.06527):33.347234,((Pristigasteridae_Pellona_flavipinnis:27.450803,Pristigasteridae_Ilisha_elongata:27.450803):46.430359,(Clupeidae_Alosa_pseudoharengus:54.138864,Clupeidae_Dorosoma_cepedianum:54.138864):19.742298):6.531342):6.68395):101.803547):1):40.3,((Alepocephalidae_Talismania_bifurcata:53.266521,((Alepocephalidae_Bathylaco_nigricans:33.802485,Alepocephalidae_Alepocephalus_tenebrosus:33.802485):10.988666,((Alepocephalidae_Rouleina_attrita:22.275085,Alepocephalidae_Xenodermichthys_copei:22.275085):19.924916,Platytroctidae_Sagamichthys_abei:42.200001):2.59115):8.47537):166.430015,((Gonorynchidae_Gonorynchus_abbreviatus:175.900001,(Mahengichthys_singidaensis:100.5035004,Chanidae_Chanos_chanos:147.100001):28.8):22.7,((((Gyrinocheilidae_Gyrinocheilus_sp:70.111694,(Catostomidae_Hypentelium_nigricans:23.594381,(Catostomidae_Carpiodes_carpio:16.355089,Catostomidae_Ictiobus_bubalus:16.355089):7.239292):46.517313):8.688307,Cobitidae_Cobitis_taenia:78.800001):20.5,(Cyprinidae_Danio_rerio:63.300001,(((Cyprinidae_Zacco_sieboldii_platypus:12.727958,Cyprinidae_Opsariichthys_uncirostris_bidens:12.727958):14.610086,((Cyprinidae_Xenocypris_argentea:12.904177,Cyprinidae_Hypophthalmichthys_molitrix:12.904177):2.917503,(Cyprinidae_Luciobrama_macrocephalus:13.439618,(Cyprinidae_Squaliobarbus_curriculus:11.472717,Cyprinidae_Mylopharyngodon_piceus:11.472717):1.966901):2.382062):11.516364):12.401957,(Cyprinidae_Tanakia_lanceolata_himantegus:35.004261,(Cyprinidae_Notemigonus_crysoleucas:22.933404,(Cyprinidae_Semotilus_atromaculatus:21.325215,((Cyprinidae_Campostoma_oligolepis:12.800846,Cyprinidae_Rhinichthys_cataractae:12.800846):4.408707,(Cyprinidae_Phenacobius_uranops:12.822623,(Cyprinidae_Pimephales_promelas_notatus:9.460336,(Cyprinidae_Luxilus_coccogenis:6.738861,Cyprinidae_Notropis_asperifrons:6.738861):2.721475):3.362287):4.38693):4.115662):1.608189):12.070857):4.73574):23.56):36):73.6,((Gymnotidae_Electrophorus_electricus:63.909447,Gymnotidae_Gymnotus_sp:63.909447):83.924067,(((Distichodontidae_Distichodus_maculatus:103.604365,Citharinidae_Citharinus_congicus:103.604365):11.170138,(Alestidae_Alestes_baremoze:106.666979,((Parodontidae_Parodon_nasus:68.800001,Hemiodontidae_Hemiodus_immaculatus:68.800001):22.415197,((Bryconidae_Brycon_pesu:66.003418,(Gasteropelecidae_Thoracocharax_stellatus:27.746828,Gasteropelecidae_Gasteropelecus_sternicla:27.746828):38.25659):1.227196,Characidae_Astyanax_mexicanus:67.230614):23.984584):15.451781):8.107524):22.236489,((Nematogenyidae_Nematogenys_inermis:110.648758,(Trichomycteridae_Trichomycterus_sp:105.886466,((Loricariidae_Loricaria_simillima:70.313425,Astroblepidae_Astroblepus_sp:70.313425):28.122359,(Callichthyidae_Callichthys_callichthys:69.200001,Callichthyidae_Corydoras_trilineatus:69.200001):29.235783):7.450682):4.762292):5.151243,(Diplomystidae_Diplomystes_nahuelbutaensis:106.203801,((Clariidae_Clarias_batrachus:47.60069,Heteropneustidae_Heteropneustes_fossilis:47.60069):38.718856,(((Sisoridae_Bagarius_yarrelli:55.522058,(Amblycipitidae_Liobagrus_aequilabris:48.027606,Akysidae_Akysis_sp:48.027606):7.494452):21.358911,(Schilbeidae_Pseudeutropius_brachypopterus:74.448774,(Bagridae_Mystus_bocourti:36.077943,Bagridae_Bagrus_ubangensis:36.077943):38.370831):2.432195):8.408138,(((Chacidae_Chaca_sp:72.429482,Cetopsidae_Cetopsis_coecutiens:72.429482):10.714797,(Cranoglanididae_Cranoglanis_bouderius:68.700001,Ictaluridae_Ictalurus_punctatus:68.700001):14.444278):1.039619,(Pangasiidae_Pangasianodon_hypophthalmus:82.158061,((Amphiliidae_Amphilius_jacksonii:69.199757,(Malapteruridae_Malapterurus_beninensis:64.740321,Mochokidae_Synodontis_batesii:64.740321):4.459436):10.993535,((Plotosidae_Plotosus_lineatus:73.039645,Siluridae_Hemisilurus_moolenburghi:73.039645):5.503862,(Auchenipteridae_Ageneiosus_atronasus:51.101654,Doradidae_Anduzedoras_oxyrhynchus:51.101654):27.441853):1.649785):1.964769):2.025837):1.105209):1.030439):19.884255):9.5962):21.210991):10.822522):25.066487):25.7):21.096535):10.503465):20.4,(Orthogonikleithrus_francogalliensis:78.37492958,(((Bathylagidae_Bathylagus_euryops:70.542512,(Microstomatidae_Nansenia_longicauda_ardesiaca:51.394,(Opisthoproctidae_Macropinna_microstoma:33.11268,Argentinidae_Argentina_sialis_silus:33.11268):18.28132):19.148512):89.057489,((Galaxiidae_Neochanna_burrowsius:34.505049,Galaxiidae_Galaxias_maculatus:34.505049):111.121934,(((Umbridae_Novumbra_hubbsi:55.762491,Umbridae_Umbra_limi:55.762491):23.63751,(Esocidae_Esox_lucius:31.366195,Esocidae_Esox_americanus:31.366195):48.033806):25.064928,(Salmonidae_Coregonus_clupeaformis:35.300001,(Salmonidae_Thymallus_brevirostris:32.456522,(Salmonidae_Oncorhynchus_nerka_mykiss:19.929533,(Salmonidae_Salvelinus_alpinus:16.737138,Salmonidae_Salmo_salar:16.737138):3.192395):12.526989):2.843479):69.164928):41.162054):13.973018):55.253787,(((Retropinnidae_Retropinna_semoni:73.700001,(((Osmeridae_Osmerus_mordax:10.332899,Osmeridae_Thaleichthys_pacificus:10.332899):5.872101,(Osmeridae_Mallotus_villosus:14.961758,Osmeridae_Hypomesus_pretiosus:14.961758):1.243242):18.133836,(Plecoglossidae_Plecoglossus_altivelis:28.70318,Salangidae_Neosalangichthys_ishikawae:28.70318):5.635656):39.361165):55.7,((Diplophidae_Diplophos_taenia:77.330737,((Gonostomatidae_Bonapartia_pedaliota:54.297445,Gonostomatidae_Margrethia_obtusirostra:54.297445):20.014374,(Gonostomatidae_Gonostoma_elongatum:69.948163,Gonostomatidae_Cyclothone_microdon:69.948163):4.363656):3.018918):6.469264,(Phosichthyidae_Pollichthys_mauli:77.722827,((Sternoptychidae_Argyropelecus_gigas:25.230481,Sternoptychidae_Maurolicus_weitzmani:25.230481):47.329876,(Stomiidae_Chauliodus_macouni_danae:63.126299,(Stomiidae_Stomias_boa:36.381906,(Stomiidae_Chirostomias_pliopterus:31.578039,((Stomiidae_Melanostomias_margaritifer:21.790927,Stomiidae_Leptostomias_longibarba:21.790927):6.240428,((Stomiidae_Photonectes_margarita:19.053467,Stomiidae_Tactostoma_macropus:19.053467):7.977888,(Stomiidae_Malacosteus_niger:25.031355,(Stomiidae_Eustomias_polyaster:24.031355,(Stomiidae_Aristostomias_scintillans:23.031355,Stomiidae_Bathophilus_flemingi_pawneei:23.031355):1):1):2):1):3.546684):4.803867):26.744393):9.434058):5.16247):6.077174):45.6):81.9,((Ateleopodidae_Ateleopus_japonicus:8.089622,Ateleopodidae_Ijimaia_antillarum:8.089622):184.210379,(((Synodontidae_Trachinocephalus_myops:34.928634,Synodontidae_Synodus_foetens:34.928634):78.971367,((Paraulopidae_Paraulopus_oblongus:101.985288,(Synodontidae_Saurida_gracilis:50.257509,Synodontidae_Harpadon_microchir:50.257509):51.727779):8.326796,(((Aulopidae_Aulopus_filamentosus:47.160387,Bathysauridae_Bathysaurus_ferox:47.160387):10.843902,(Ipnopidae_Ipnops_murrayi:13.82,Ipnopidae_Ipnops_agassizi:13.82):44.184289):23.635948,((Ipnopidae_Bathypterois_atricolor:69.328167,(Giganturidae_Gigantura_indica:13.525058,Giganturidae_Gigantura_chuni:13.525058):55.803109):6.462647,(Scopelarchidae_Benthalbella_macropinna:62.490696,(Chlorophthalmidae_Chlorophthalmus_agassizi:61.13635,(Sudidae_Sudis_atrox:55.026295,((Alepisauridae_Anotopterus_pharao:45.661943,(Alepisauridae_Omosudis_lowii:36.284216,Alepisauridae_Alepisaurus_ferox:36.284216):9.377727):8.238175,(Paralepididae_Stemonosudis_intermedia_macrura:50.122687,(Evermannellidae_Evermannella_balbo:46.686813,Paralepididae_Paralepis_coregonoides:46.686813):3.435874):3.777431):1.126177):6.110055):1.354346):13.300118):5.849423):28.671847):3.587917):68.9,(((Neoscopelidae_Neoscopelus_macrolepidotus:42.408365,Neoscopelidae_Scopelengys_tristis:42.408365):31.191636,((Myctophidae_Lampadena_speculigera:39.81673,Myctophidae_Lampanyctus_macdonaldi:39.81673):11.872,(Myctophidae_Benthosema_glaciale:28.30241,Myctophidae_Myctophum_punctatum:28.30241):23.38632):21.911271):99.5,(((Lamprididae_Lampris_guttatus:65.552505,(Regalecidae_Regalecus_russelii:37.682943,Trachipteridae_Trachipterus_trachypterus:37.682943):27.869562):84.747496,(((Mcconichthys_longipinnis:1,(Aphredoderidae_Aphredoderus_sayanus:41.500001,Amblyopsidae_Chologaster_cornuta:41.500001):23.22261103):1,(Lateopisciculus_turrifumosus:1,(Percopsidae_Percopsis_omiscomaycus:14.617149,Percopsidae_Percopsis_transmontana:14.617149):44.4907011):6.614761937):69.26771997,(Zeidae_Zeus_faber:107.100001,(Stylephoridae_Stylephorus_chordatus:78.800001,(Merlucciidae_Merluccius_productus:45.655086,(((Macrouridae_Gadomus_dispar:9.947595,Macrouridae_Bathygadus_favosus:9.947595):28.939269,(Steindachneriidae_Steindachneria_argentea:35.929071,((Macrouridae_Malacocephalus_laevis:17.761718,Macrouridae_Trachonurus_sulcatus:17.761718):10.126413,(Macrouridae_Coelorinchus_caribbaeus:21.883517,Macrouridae_Coryphaenoides_armatus:21.883517):6.004614):8.04094):2.957793):2.613137,((Moridae_Laemonema_goodebeanorum:25.08833,(Moridae_Halargyreus_johnsonii:16.444614,(Moridae_Lepidion_ensiferus:5.35693,Moridae_Antimora_rostrata:5.35693):11.087684):8.643716):13.1716,((Phycidae_Urophycis_tenuis:5.240351,Phycidae_Urophycis_chuss:5.240351):28.607969,(Lotidae_Gaidropsarus_ensis:29.800286,(Lotidae_Lota_lota:23.935071,(Gadidae_Melanogrammus_aeglefinus:5.86839,Gadidae_Gadus_morhua:5.86839):18.066681):5.865215):4.048034):4.41161):3.240071):4.155085):33.144915):28.3):27.890331):15.309669):11.08283,(Polymixiidae_Polymixia_japonica:154.800001,(((Diretmidae_Diretmus_argenteus:53.99855,(Monocentridae_Monocentris_japonica:33.600001,((Trachichthyidae_Hoplostethus_occidentalis_atlanticus:24.361165,Trachichthyidae_Gephyroberyx_darwinii:24.361165):7.919156,(Anoplogastridae_Anoplogaster_cornuta:31.22776,Trachichthyidae_Paratrachichthys_sajademalensis:31.22776):1.052561):1.31968):20.398549):71.275924,((Berycidae_Beryx_decadactylus:70.612394,(Melamphaidae_Poromitra_crassiceps:39.138751,(Melamphaidae_Scopelogadus_beanii:32.603956,Melamphaidae_Melamphaes_suborbitalis:32.603956):6.534795):31.473643):40.295775,(((Rondeletiidae_Rondeletia_bicolor:18.616456,Rondeletiidae_Rondeletia_loricata:18.616456):36.662305,Barbourisiidae_Barbourisia_rufa:55.278761):34.605685,(Cetomimidae_Cetostoma_regani:55.492218,(Cetomimidae_Cetomimus_craneae:10.537863,Cetomimidae_Cetomimus_compuctus:10.537863):44.954355):34.392228):21.023723):14.366305):21.189147,((Holocentridae_Sargocentron_diadema:25.43456,Holocentridae_Holocentrus_rufus:25.43456):119.565441,((Ophidiidae_Brotula_multibarbata:66.227334,(Ophidiidae_Lepophidium_profundorum:23.036156,(Ophidiidae_Genypterus_blacodes:21.590629,Ophidiidae_Ophidion_holbrookii:21.590629):1.445527):43.191178):66.572667,((Batrachoididae_Porichthys_notatus:39.800001,Batrachoididae_Opsanus_tau:39.800001):87,(((Kurtidae_Kurtus_indicus:80.300001,Apogonidae_Apogon_campbelli:80.300001):22,(Eleotridae_Eleotris_acanthopoma_pisonis:64.430477,(Gobiidae_Gobiosoma_bosc:45.630567,Microdesmidae_Microdesmus_longipinnis:45.630567):18.79991):37.869524):19.3,((((Dactylopteridae_Dactylopterus_volitans:68.121568,(Aulostomidae_Aulostomus_maculatus:3.200001,Aulostomidae_Aulostomus_chinensis:3.200001):64.921567):6.178433,((Fistulariidae_Fistularia_petimba:65.463647,Mullidae_Mullus_auratus:65.463647):6.26906,(Callionymidae_Callionymus_sp_bairdi:69.154942,Syngnathidae_Syngnathus_fuscus:69.154942):2.577765):2.567294):20.27,(Chiasmodontidae_Chiasmodon_sp:43.900001,((Centrolophidae_Icichthys_lockingtoni:34.370774,Pomatomidae_Pomatomus_saltatrix:34.370774):6.934458,(((Ariommatidae_Ariomma_bondi:25.644121,Nomeidae_Psenes_cyanophrys:25.644121):5.370208,Scombridae_Auxis_rochei:31.014329):6.588438,((Stromateidae_Peprilus_paru:35.602767,(Gempylidae_Paradiplospinus_gracilis:31.499638,Bramidae_Brama_brama:31.499638):4.103129):1,(Icosteidae_Icosteus_aenigmaticus:33.972252,Trichiuridae_Trichiurus_lepturus:33.972252):2.630515):1):3.702465):2.594769):50.67):21.916687,((((((Synbranchidae_Monopterus_albus:69.146846,Indostomidae_Indostomus_paradoxus:69.146846):4.548479,(Mastacembelidae_Macrognathus_siamensis:18.057555,Mastacembelidae_Mastacembelus_erythrotaenia:18.057555):55.63777):7.004676,((Anabantidae_Ctenopoma_acutirostre_kingsleyae:62.782323,(Helostomatidae_Helostoma_temminkii:59.687826,Osphronemidae_Trichopodus_pectoralis:59.687826):3.094497):7.86905,(Channidae_Channa_striata:67.428514,Nandidae_Nandus_nandus:67.428514):3.222859):10.048628):15.7,((Nematistiidae_Nematistius_pectoralis:60.067614,((Coryphaenidae_Coryphaena_hippurus:19.124214,Rachycentridae_Rachycentron_canadum:19.124214):12.775787,(Echeneidae_Remora_osteochir_australis:10.178122,Echeneidae_Echeneis_naucrates:10.178122):21.721879):28.167613):9.732387,((Sphyraenidae_Sphyraena_sphyraena:65.900885,(Menidae_Mene_maculata:60.339531,Polynemidae_Polydactylus_octonemus:60.339531):5.561354):2.899116,((((Leptobramidae_Leptobrama_muelleri:46.58559,Toxotidae_Toxotes_jaculatrix:46.58559):5.096327,(Xiphiidae_Xiphias_gladius:27.833842,Istiophoridae_Istiophorus_platypterus:27.833842):23.848075):5.298938,Carangidae_Trachinotus_falcatus:56.980855):4.861848,((Eolates_gracilis:1,(Centropomidae_Psammoperca_waigiensis:33.068144,(Centropomidae_Lates_calcarifer:13.595548,Centropomidae_Lates_microlepis:13.595548):19.472596):22.02368708):5.750871917,(Heteronectes_chaneti:4.247511796,(Amphistium_paradoxum:5.556956996,(Psettodidae_Psettodes_erumei:57.842703,(Citharidae_Lepidoblepharon_ophthalmolepis:53.600001,((Cynoglossidae_Cynoglossus_interruptus:42.400002,(Soleidae_Solea_solea:24.944331,Soleidae_Soleichthys_heterorhinos:24.944331):17.455671):9.674607,((Scophthalmidae_Scophthalmus_aquosus:23.305906,Scophthalmidae_Lepidorhombus_boscii:23.305906):26.973285,((Paralichthyidae_Paralichthys_dentatus:40.749719,Bothidae_Bothus_lunatus:40.749719):4.387561,(Paralichthyidae_Pseudorhombus_pentophthalmus:21.404631,(((Pleuronectidae_Glyptocephalus_zachirus:5.096079,Pleuronectidae_Microstomus_pacificus:5.096079):2.538752,(Pleuronectidae_Lyopsetta_exilis:6.085211,Pleuronectidae_Hippoglossus_hippoglossus:6.085211):1.54962):2.215451,(Pleuronectidae_Limanda_limanda:8.798272,((Pleuronectidae_Platichthys_stellatus:3.717184,Pleuronectidae_Pleuronectes_platessa:3.717184):3.081088,(Pleuronectidae_Lepidopsetta_bilineata:4.798272,Pleuronectidae_Psettichthys_melanostictus:4.798272):2):2):1.05201):11.554349):23.732649):5.141911):1.795418):1.525392):4.242702):1):1):1):1):6.957298):1):26.6):12.043384,(((Polycentridae_Polycentrus_schomburgkii:94.214904,(Pholidichthyidae_Pholidichthys_leucotaenia:88.700001,Cichlidae_Cichla_temensis:88.700001):5.514903):1,(((Atherinopsidae_Atherinopsis_californiensis:48.715388,Atherinopsidae_Menidia_menidia:48.715388):22.225577,(Isonidae_Iso_sp:49.317292,(Atherinidae_Atherinomorus_stipes:43.78646,(Melanotaeniidae_Melanotaenia_splendida:39.870052,Pseudomugilidae_Pseudomugil_gertrudae:39.870052):3.916408):5.530832):21.623673):6.46052,(((Poeciliidae_Poecilia_latipinna_reticulata:14.292152,(Poeciliidae_Heterandria_formosa:11.366823,(Poeciliidae_Gambusia_affinis:5.736451,Poeciliidae_Belonesox_belizanus:5.736451):5.630372):2.925329):30.13151,(Cyprinodontidae_Cyprinodon_variegatus:40.469994,Fundulidae_Fundulus_parvipinnis:40.469994):3.953668):31.977823,(Adrianichthyidae_Oryzias_latipes:71.878571,((Zenarchopteridae_Dermogenys_collettei:29.883878,((Belonidae_Tylosurus_crocodilus:6.568056,Belonidae_Ablennes_hians:6.568056):17.429484,(Scomberesocidae_Cololabis_saira:2.646712,Scomberesocidae_Scomberesox_saurus:2.646712):21.350828):5.886338):17.178962,(Hemiramphidae_Hyporhamphus_affinis:36.892311,((Hemiramphidae_Hemiramphus_brasiliensis:13.391766,Hemiramphidae_Oxyporhamphus_micropterus:13.391766):10.259915,((Exocoetidae_Cypselurus_callopterus:6.413654,Exocoetidae_Exocoetus_monocirrhus:6.413654):4.605967,Exocoetidae_Cheilopogon_pinnatibarbatus:11.019621):12.63206):13.24063):10.170529):24.815731):4.522914):1):17.813419):1.999954,((Pseudochromidae_Halidesmus_scapularis:84.850287,Pomacentridae_Pomacentrus_brachialis:84.850287):8.187103,(Embiotocidae_Cymatogaster_aggregata:91.529056,(Mugilidae_Mugil_cephalus:89.757655,(Plesiopidae_Plesiops_coeruleolineatus:86.977996,(Opistognathidae_Opistognathus_maxillosus:79.196597,(((Gobiesocidae_Lepadichthys_lineatus:37.560081,Gobiesocidae_Gobiesox_maeandricus:37.560081):35.985217,Tripterygiidae_Enneanectes_altivelis:73.545298):3.122761,((Clinidae_Clinus_cottoides:50.741024,(Dactyloscopidae_Platygillellus_rubrocinctus:45.641062,Chaenopsidae_Chaenopsis_alepidota:45.641062):5.099962):17.520576,(Blenniidae_Stanulus_seychellensis:31.042358,Blenniidae_Blenniella_cyanostigma:31.042358):37.219242):8.406459):2.528538):7.781399):2.779659):1.771401):1.508334):4.177468):11.228527):3.450865,(Gerreidae_Eucinostomus_argenteus:108.000002,(((Labridae_Lachnolaimus_maximus:59.822938,Odacidae_Haletta_semifasciata:59.822938):16.826114,Scaridae_Scarus_globiceps:76.649052):30.35095,(((Uranoscopidae_Uranoscopus_sulphureus:83.996275,(Ammodytidae_Ammodytes_hexapterus:78.584442,Pinguipedidae_Parapercis_punctulata:78.584442):5.411833):11.903726,(((Acropomatidae_Acropoma_japonicum:90.360051,((Percophidae_Acanthaphritis_unoorum:66.16617,Creediidae_Limnichthys_sp:66.16617):18.796708,(Glaucosomatidae_Glaucosoma_buergeri:66.617288,Pempheridae_Pempheris_schomburgkii:66.617288):18.34559):5.397173):2.814411,(((Oplegnathidae_Oplegnathus_punctatus:61.206964,Kuhliidae_Kuhlia_rupestris:61.206964):7.298332,Kyphosidae_Kyphosus_sectatrix:68.505296):16.157201,(Percichthyidae_Percichthys_trucha:69.999181,((Cirrhitidae_Cirrhitichthys_falco:66.467051,Cheilodactylidae_Cheilodactylus_fasciatus:66.467051):2.53213,(Enoplosidae_Enoplosus_armatus:63.04721,Centrarchidae_Acantharchus_pomotis:63.04721):5.951971):1):14.663316):8.511965):1.725539,(Serranidae_Pseudogramma_polyacantha:82.200001,(Percidae_Perca_fluviatilis:78.577195,((Bovichtidae_Bovichtus_diacanthus:63.021097,(Nototheniidae_Notothenia_coriiceps:9.671082,(Harpagiferidae_Harpagifer_antarcticus:8.390321,(Bathydraconidae_Gymnodraco_acuticeps:7.390321,Channichthyidae_Chionodraco_rastrospinosus:7.390321):1):1.280761):53.350015):12.936917,(((Peristediidae_Peristedion_ecuadorense:54.532565,Triglidae_Prionotus_carolinus:54.532565):11.953546,(Synanceiidae_Synanceia_verrucosa:62.510934,Scorpaenidae_Scorpaenodes_guamensis:62.510934):3.975177):6.621302,(Platycephalidae_Platycephalus_indicus:70.795272,(Anoplopomatidae_Anoplopoma_fimbria:46.504594,((Bathymasteridae_Rathbunella_hypoplecta:22.798552,(Stichaeidae_Cebidichthys_violaceus:15.58542,(Zoarcidae_Zoarces_americanus_viviparus:12.493576,(Anarhichadidae_Anarrhichthys_ocellatus:9.933504,(Zaproridae_Zaprora_silenus:8.933504,Cryptacanthodidae_Cryptacanthodes_maculatus:8.933504):1):2.560072):3.091844):7.213132):19.028758,(((Aulorhynchidae_Aulorhynchus_flavidus:25.280385,Hypoptychidae_Hypoptychus_dybowskii:25.280385):1.019618,((Gasterosteidae_Culaea_inconstans:19.844899,(Gasterosteidae_Apeltes_quadracus:17.136644,Gasterosteidae_Spinachia_spinachia:17.136644):2.708255):1.782129,(Gasterosteidae_Pungitius_pungitius:16.827665,Gasterosteidae_Gasterosteus_aculeatus:16.827665):4.799363):4.672975):13.53479,(Hexagrammidae_Hexagrammos_decagrammus:27.818969,((Cyclopteridae_Cyclopterus_lumpus:20.762394,Liparidae_Liparis_pulchellus:20.762394):4.037609,(Agonidae_Hypsagonus_quadricornis:20.371382,(Cottidae_Cottus_carolinae:18.19359,Psychrolutidae_Psychrolutes_phrictus:18.19359):2.177792):4.428621):3.018966):12.015824):1.992517):4.677284):24.290678):2.312141):2.850601):2.619181):3.622806):12.7):1):10.100001,((Drepaneidae_Drepane_punctata:67.658011,Ephippidae_Chaetodipterus_faber:67.658011):37.341991,(((Lobotidae_Lobotes_pacificus_surinamensis:86.438004,Sciaenidae_Menticirrhus_undulatus_littoralis:86.438004):5.761997,((Monodactylidae_Monodactylus_sebae:70.333247,(Avitoluvarus_eocaenicus:20.68779995,(Zanclidae_Zanclus_cornutus:55.859022,Acanthuridae_Acanthurus_triostegus:55.859022):4.740979):9.733246):15.366754,((Pomacanthidae_Pomacanthus_semicirculatus:54.434118,(Leiognathidae_Leiognathus_equulus:32.700001,Chaetodontidae_Chelmon_rostratus:32.700001):21.734117):17.438962,(Emmelichthyidae_Erythrocles_schlegelii:69.871718,(Malacanthidae_Malacanthus_plumieri:68.303733,(Haemulidae_Haemulon_aurolineatum:64.600001,Lutjanidae_Lutjanus_griseus:64.600001):3.703732):1.567985):2.001362):13.826921):6.5):11.800001,((Sillaginidae_Sillago_sihama:85.853361,(Nemipteridae_Pentapodus_caninus:80.270573,(Lethrinidae_Lethrinus_erythropterus:74.370539,Sparidae_Stenotomus_chrysops:74.370539):5.900034):5.582788):17.146641,(Siganidae_Siganus_spinus:102.000002,((Scatophagidae_Scatophagus_argus:75.860371,Priacanthidae_Heteropriacanthus_cruentatus:75.860371):25.139631,((Caproidae_Antigonia_rubescens:10.905674,Caproidae_Antigonia_capros:10.905674):89.094328,(((Lophiidae_Lophiodes_reticulatus:22.462185,Lophiidae_Lophius_gastrophysus:22.462185):42.032119,((Antennariidae_Histrio_histrio:12.333102,Antennariidae_Antennatus_coccineus:12.333102):33.466899,(Chaunacidae_Chaunax_suttkusi:38.499563,(Gigantactinidae_Gigantactis_sp:32.911618,((Ceratiidae_Cryptopsaras_couesii:20.548708,Ceratiidae_Ceratias_holboelli:20.548708):10.416157,((Himantolophidae_Himantolophus_albinares_sagamius:14.456802,Melanocetidae_Melanocetus_murrayi:14.456802):9.124159,(Oneirodidae_Oneirodes_macrosteus:10.164357,Oneirodidae_Dolopichthys_sp:10.164357):13.416604):7.383904):1.946753):5.587945):7.300438):18.694303):34.505698,(((Prohollardia_avita:38.58278477,(Protacanthodes_nimesensis:1,Triacanthidae_Triacanthus_biaculeatus:48.24066362):16.25230738):10.123893,(Aracanidae_Aracana_aurita:54.800001,(Ostraciidae_Ostracion_cubicus:51.000002,Ostraciidae_Rhinesomus_triqueter:51.000002):3.799999):19.816863):3.183137,(((Diodontidae_Diodon_holocanthus:12.198387,Diodontidae_Chilomycterus_schoepfii:12.198387):43.701614,(Tetraodontidae_Canthigaster_bennetti:24.22571,Tetraodontidae_Tetraodon_miurus:24.22571):31.674291):17.334859,((Molidae_Ranzania_laevis:22,Molidae_Mola_mola:22):46.61027,((Monacanthidae_Stephanolepis_hispidus:28.823604,Monacanthidae_Aluterus_scriptus:28.823604):14.976396,(Balistidae_Xanthichthys_ringens:20.7,(Balistidae_Balistes_vetula:19.7,Balistidae_Sufflamen_fraenatum:19.7):1):23.1):24.81027):4.62459):4.565141):21.200001):1):1):1):1):1):1):1):1):1):3.894248):4.592438):5.113313):5.2):6):12.2):1.46362):8.33638):6.58283):11.71717):9.7):9.5):19):3.553787):17.946213):17.8):23.2):9.5):1):1):37.2):1):1):1,(Fukangichthys_longidorsalis:10.55293668,(Scanilepis_dubia:47.42296831,Evenkia_eunotoptera:1):1):71.42757732):1,Australosomus:74.42679369):1):22.6):1):3.133535377):1):10.28177806):20.43179962):1):1):1.605520256):1):4.447367683):1):47);

((Guiyu_oneiros:1,(Onychodus_jandemarrai:37.35696206,((Diplocercides:1,(Latimeriidae_Latimeria_chalumnae:330.1359339,Rhabdoderma:1):35.43138783):46.94830527,((Neoceratodontidae_Neoceratodus_forsteri:279.7982796,Lepidosirenidae_Lepidosiren_paradoxa:279.7982796):128.2017204,((Osteolepis_macrolepidotus:1,Gyroptychius_milleri:8.7044125):1,Eusthenopterus_foordi:24.46796757):5.936350361):4.515627):1):13.3923154):16.0920596,((Cheirolepis_trailli:1,Cheirolepis_schultzei:5.495195403):3.160177284,((Polypteridae_Erpetoichthys_calabaricus:29.200001,Polypteridae_Polypterus_senegalus:29.200001):365.800001,(Osorioichthys_marginis:24.11203214,((Tegeolepis_clarki:16.25841465,Howqualepis_rostridens:1):3.21013034,((Gogosardina_coatesi:2.40555104,(Mimipiscis_bartrami:1,Mimipiscis_toombsi:3.96197514):1):5.116581122,((Moythomasia_lineata:1,Moythomasia_durgaringa:4.567968418):1.329233891,(Stegotrachelus_finlayi:1,(Limnomis_delaneyi:1,(Wendyichthys_dicksoni:31.23504271,(Kentuckia_deani:1,((Mesopoma_planti:1.048715417,Mesopoma_carricki:1):9.883167491,((Birgeria_stensioei:1,(Chondrosteus_acipenseroides:1,(Saurichthys_dawaziensis:1,((Protopsephurus_liui:1,Polyodontidae_Polyodon_spathula:129.8905905):9.009411533,(Acipenseridae_Acipenser_fulvescens:38.579335,(Acipenseridae_Scaphirhynchus_platorynchus:19.375515,Acipenseridae_Scaphirhynchus_albus:19.375515):19.20382):100.320667):23.88492139):38.94263882):50.75624429):97.6161945,(Boreosomus:75.62836141,(((Perleidus_altolepis:84.61403697,(Luganoia_lepidosteoides:93.64945472,(((Macrosemius_fourneti:90.66421829,((Kyphosichthys_grandei:1,Lepisosteidae_Lepisosteus_osseus:245.2747262):1,Semionotus_elegans:45.98951386):1):20.62527485,(Watsonulus_eugnathoides:1,(Amblysemius:1,(Pachyamia_latimaxillaris:1.368034585,(Tomognathus_mordax:1,Amiidae_Amia_calva:98.66785238):1):52.04066807):101.0816049):15.10987561):54.6,(Pachycormus:104.1044578,(Crossognathus_danubiensis:177.0510127,(((Anaethalion_angustus:1,Anaethalion_knorri:2.718143919):46.43329351,((Megalopidae_Megalops_atlanticus:133.565966,Elopidae_Elops_saurus:133.565966):62.634035,(Albulidae_Albula_vulpes:150.800001,((Notacanthidae_Notacanthus_chemnitzii:50.669287,(Halosauridae_Halosauropsis_macrochir:40.366211,Halosauridae_Aldrovandia_affinis:40.366211):10.303076):50.330714,(((Eurypharyngidae_Eurypharynx_pelecanoides:25.866364,Saccopharyngidae_Saccopharynx_ampullaceus:25.866364):44.322494,(Nemichthyidae_Nemichthys_scolopaceus:58.739485,Anguillidae_Anguilla_rostrata:58.739485):11.449373):9.198895,(Serrivomeridae_Serrivomer_beanii:76.053487,(Congridae_Conger_oceanicus:65.416147,(Muraenesocidae_Muraenesox_cinereus:57.193624,Ophichthidae_Myrichthys_maculosus:57.193624):8.222523):10.63734):3.334266):21.612248):49.8):45.4):1):86.1,((Lycoptera_davidi:99.86111653,(Paralycoptera_wui:113.8594082,(Xixiaichthys_tongxinens:99.91871628,(((Eohiodon_woodruffi:3.611121254,Eohiodon_rosei:1):1,(Hiodon_consteniorum:1,(Hiodontidae_Hiodon_tergisus:9.520291,Hiodontidae_Hiodon_alosoides:9.520291):29.27712004):17.36553173):170.9370582,(Chauliopareion_mahengeense:121.8838666,(Pantodontidae_Pantodon_buchholzi:163.100001,((((Brychaetus_muelleri:1,Phareodus_encaustus:7.686857879):24.3242442,Arapaimidae_Arapaima_gigas:79.3):25.549112,Osteoglossidae_Osteoglossum_bicirrhosum:104.849112):43.475578,(Notopteridae_Xenomystus_nigri:120.300002,Gymnarchidae_Gymnarchus_niloticus:120.300002):28.024688):14.775311):1):63):1):1):1):43.7,((((Diplomystus_brevissimus:46.50340418,Sorbinichthys_africanus:1):90.53168511,(Denticipitidae_Denticeps_clupeoides:188.900001,(Chirocentridae_Chirocentrus_dorab:87.096454,((Engraulidae_Coilia_nasus:47.06527,Engraulidae_Engraulis_mordax_eurystole:47.06527):33.347234,((Pristigasteridae_Pellona_flavipinnis:27.450803,Pristigasteridae_Ilisha_elongata:27.450803):46.430359,(Clupeidae_Alosa_pseudoharengus:54.138864,Clupeidae_Dorosoma_cepedianum:54.138864):19.742298):6.531342):6.68395):101.803547):1):40.3,((Alepocephalidae_Talismania_bifurcata:53.266521,((Alepocephalidae_Bathylaco_nigricans:33.802485,Alepocephalidae_Alepocephalus_tenebrosus:33.802485):10.988666,((Alepocephalidae_Rouleina_attrita:22.275085,Alepocephalidae_Xenodermichthys_copei:22.275085):19.924916,Platytroctidae_Sagamichthys_abei:42.200001):2.59115):8.47537):166.430015,((Gonorynchidae_Gonorynchus_abbreviatus:175.900001,(Mahengichthys_singidaensis:101.1405783,Chanidae_Chanos_chanos:147.100001):28.8):22.7,((((Gyrinocheilidae_Gyrinocheilus_sp:70.111694,(Catostomidae_Hypentelium_nigricans:23.594381,(Catostomidae_Carpiodes_carpio:16.355089,Catostomidae_Ictiobus_bubalus:16.355089):7.239292):46.517313):8.688307,Cobitidae_Cobitis_taenia:78.800001):20.5,(Cyprinidae_Danio_rerio:63.300001,(((Cyprinidae_Zacco_sieboldii_platypus:12.727958,Cyprinidae_Opsariichthys_uncirostris_bidens:12.727958):14.610086,((Cyprinidae_Xenocypris_argentea:12.904177,Cyprinidae_Hypophthalmichthys_molitrix:12.904177):2.917503,(Cyprinidae_Luciobrama_macrocephalus:13.439618,(Cyprinidae_Squaliobarbus_curriculus:11.472717,Cyprinidae_Mylopharyngodon_piceus:11.472717):1.966901):2.382062):11.516364):12.401957,(Cyprinidae_Tanakia_lanceolata_himantegus:35.004261,(Cyprinidae_Notemigonus_crysoleucas:22.933404,(Cyprinidae_Semotilus_atromaculatus:21.325215,((Cyprinidae_Campostoma_oligolepis:12.800846,Cyprinidae_Rhinichthys_cataractae:12.800846):4.408707,(Cyprinidae_Phenacobius_uranops:12.822623,(Cyprinidae_Pimephales_promelas_notatus:9.460336,(Cyprinidae_Luxilus_coccogenis:6.738861,Cyprinidae_Notropis_asperifrons:6.738861):2.721475):3.362287):4.38693):4.115662):1.608189):12.070857):4.73574):23.56):36):73.6,((Gymnotidae_Electrophorus_electricus:63.909447,Gymnotidae_Gymnotus_sp:63.909447):83.924067,(((Distichodontidae_Distichodus_maculatus:103.604365,Citharinidae_Citharinus_congicus:103.604365):11.170138,(Alestidae_Alestes_baremoze:106.666979,((Parodontidae_Parodon_nasus:68.800001,Hemiodontidae_Hemiodus_immaculatus:68.800001):22.415197,((Bryconidae_Brycon_pesu:66.003418,(Gasteropelecidae_Thoracocharax_stellatus:27.746828,Gasteropelecidae_Gasteropelecus_sternicla:27.746828):38.25659):1.227196,Characidae_Astyanax_mexicanus:67.230614):23.984584):15.451781):8.107524):22.236489,((Nematogenyidae_Nematogenys_inermis:110.648758,(Trichomycteridae_Trichomycterus_sp:105.886466,((Loricariidae_Loricaria_simillima:70.313425,Astroblepidae_Astroblepus_sp:70.313425):28.122359,(Callichthyidae_Callichthys_callichthys:69.200001,Callichthyidae_Corydoras_trilineatus:69.200001):29.235783):7.450682):4.762292):5.151243,(Diplomystidae_Diplomystes_nahuelbutaensis:106.203801,((Clariidae_Clarias_batrachus:47.60069,Heteropneustidae_Heteropneustes_fossilis:47.60069):38.718856,(((Sisoridae_Bagarius_yarrelli:55.522058,(Amblycipitidae_Liobagrus_aequilabris:48.027606,Akysidae_Akysis_sp:48.027606):7.494452):21.358911,(Schilbeidae_Pseudeutropius_brachypopterus:74.448774,(Bagridae_Mystus_bocourti:36.077943,Bagridae_Bagrus_ubangensis:36.077943):38.370831):2.432195):8.408138,(((Chacidae_Chaca_sp:72.429482,Cetopsidae_Cetopsis_coecutiens:72.429482):10.714797,(Cranoglanididae_Cranoglanis_bouderius:68.700001,Ictaluridae_Ictalurus_punctatus:68.700001):14.444278):1.039619,(Pangasiidae_Pangasianodon_hypophthalmus:82.158061,((Amphiliidae_Amphilius_jacksonii:69.199757,(Malapteruridae_Malapterurus_beninensis:64.740321,Mochokidae_Synodontis_batesii:64.740321):4.459436):10.993535,((Plotosidae_Plotosus_lineatus:73.039645,Siluridae_Hemisilurus_moolenburghi:73.039645):5.503862,(Auchenipteridae_Ageneiosus_atronasus:51.101654,Doradidae_Anduzedoras_oxyrhynchus:51.101654):27.441853):1.649785):1.964769):2.025837):1.105209):1.030439):19.884255):9.5962):21.210991):10.822522):25.066487):25.7):21.096535):10.503465):20.4,(Orthogonikleithrus_francogalliensis:76.2584438,(((Bathylagidae_Bathylagus_euryops:70.542512,(Microstomatidae_Nansenia_longicauda_ardesiaca:51.394,(Opisthoproctidae_Macropinna_microstoma:33.11268,Argentinidae_Argentina_sialis_silus:33.11268):18.28132):19.148512):89.057489,((Galaxiidae_Neochanna_burrowsius:34.505049,Galaxiidae_Galaxias_maculatus:34.505049):111.121934,(((Umbridae_Novumbra_hubbsi:55.762491,Umbridae_Umbra_limi:55.762491):23.63751,(Esocidae_Esox_lucius:31.366195,Esocidae_Esox_americanus:31.366195):48.033806):25.064928,(Salmonidae_Coregonus_clupeaformis:35.300001,(Salmonidae_Thymallus_brevirostris:32.456522,(Salmonidae_Oncorhynchus_nerka_mykiss:19.929533,(Salmonidae_Salvelinus_alpinus:16.737138,Salmonidae_Salmo_salar:16.737138):3.192395):12.526989):2.843479):69.164928):41.162054):13.973018):55.253787,(((Retropinnidae_Retropinna_semoni:73.700001,(((Osmeridae_Osmerus_mordax:10.332899,Osmeridae_Thaleichthys_pacificus:10.332899):5.872101,(Osmeridae_Mallotus_villosus:14.961758,Osmeridae_Hypomesus_pretiosus:14.961758):1.243242):18.133836,(Plecoglossidae_Plecoglossus_altivelis:28.70318,Salangidae_Neosalangichthys_ishikawae:28.70318):5.635656):39.361165):55.7,((Diplophidae_Diplophos_taenia:77.330737,((Gonostomatidae_Bonapartia_pedaliota:54.297445,Gonostomatidae_Margrethia_obtusirostra:54.297445):20.014374,(Gonostomatidae_Gonostoma_elongatum:69.948163,Gonostomatidae_Cyclothone_microdon:69.948163):4.363656):3.018918):6.469264,(Phosichthyidae_Pollichthys_mauli:77.722827,((Sternoptychidae_Argyropelecus_gigas:25.230481,Sternoptychidae_Maurolicus_weitzmani:25.230481):47.329876,(Stomiidae_Chauliodus_macouni_danae:63.126299,(Stomiidae_Stomias_boa:36.381906,(Stomiidae_Chirostomias_pliopterus:31.578039,((Stomiidae_Melanostomias_margaritifer:21.790927,Stomiidae_Leptostomias_longibarba:21.790927):6.240428,((Stomiidae_Photonectes_margarita:19.053467,Stomiidae_Tactostoma_macropus:19.053467):7.977888,(Stomiidae_Malacosteus_niger:25.031355,(Stomiidae_Eustomias_polyaster:24.031355,(Stomiidae_Aristostomias_scintillans:23.031355,Stomiidae_Bathophilus_flemingi_pawneei:23.031355):1):1):2):1):3.546684):4.803867):26.744393):9.434058):5.16247):6.077174):45.6):81.9,((Ateleopodidae_Ateleopus_japonicus:8.089622,Ateleopodidae_Ijimaia_antillarum:8.089622):184.210379,(((Synodontidae_Trachinocephalus_myops:34.928634,Synodontidae_Synodus_foetens:34.928634):78.971367,((Paraulopidae_Paraulopus_oblongus:101.985288,(Synodontidae_Saurida_gracilis:50.257509,Synodontidae_Harpadon_microchir:50.257509):51.727779):8.326796,(((Aulopidae_Aulopus_filamentosus:47.160387,Bathysauridae_Bathysaurus_ferox:47.160387):10.843902,(Ipnopidae_Ipnops_murrayi:13.82,Ipnopidae_Ipnops_agassizi:13.82):44.184289):23.635948,((Ipnopidae_Bathypterois_atricolor:69.328167,(Giganturidae_Gigantura_indica:13.525058,Giganturidae_Gigantura_chuni:13.525058):55.803109):6.462647,(Scopelarchidae_Benthalbella_macropinna:62.490696,(Chlorophthalmidae_Chlorophthalmus_agassizi:61.13635,(Sudidae_Sudis_atrox:55.026295,((Alepisauridae_Anotopterus_pharao:45.661943,(Alepisauridae_Omosudis_lowii:36.284216,Alepisauridae_Alepisaurus_ferox:36.284216):9.377727):8.238175,(Paralepididae_Stemonosudis_intermedia_macrura:50.122687,(Evermannellidae_Evermannella_balbo:46.686813,Paralepididae_Paralepis_coregonoides:46.686813):3.435874):3.777431):1.126177):6.110055):1.354346):13.300118):5.849423):28.671847):3.587917):68.9,(((Neoscopelidae_Neoscopelus_macrolepidotus:42.408365,Neoscopelidae_Scopelengys_tristis:42.408365):31.191636,((Myctophidae_Lampadena_speculigera:39.81673,Myctophidae_Lampanyctus_macdonaldi:39.81673):11.872,(Myctophidae_Benthosema_glaciale:28.30241,Myctophidae_Myctophum_punctatum:28.30241):23.38632):21.911271):99.5,(((Lamprididae_Lampris_guttatus:65.552505,(Regalecidae_Regalecus_russelii:37.682943,Trachipteridae_Trachipterus_trachypterus:37.682943):27.869562):84.747496,(((Mcconichthys_longipinnis:1,(Aphredoderidae_Aphredoderus_sayanus:41.500001,Amblyopsidae_Chologaster_cornuta:41.500001):25.40277447):1,(Lateopisciculus_turrifumosus:1,(Percopsidae_Percopsis_omiscomaycus:14.617149,Percopsidae_Percopsis_transmontana:14.617149):45.10394551):8.181680959):67.08755653,(Zeidae_Zeus_faber:107.100001,(Stylephoridae_Stylephorus_chordatus:78.800001,(Merlucciidae_Merluccius_productus:45.655086,(((Macrouridae_Gadomus_dispar:9.947595,Macrouridae_Bathygadus_favosus:9.947595):28.939269,(Steindachneriidae_Steindachneria_argentea:35.929071,((Macrouridae_Malacocephalus_laevis:17.761718,Macrouridae_Trachonurus_sulcatus:17.761718):10.126413,(Macrouridae_Coelorinchus_caribbaeus:21.883517,Macrouridae_Coryphaenoides_armatus:21.883517):6.004614):8.04094):2.957793):2.613137,((Moridae_Laemonema_goodebeanorum:25.08833,(Moridae_Halargyreus_johnsonii:16.444614,(Moridae_Lepidion_ensiferus:5.35693,Moridae_Antimora_rostrata:5.35693):11.087684):8.643716):13.1716,((Phycidae_Urophycis_tenuis:5.240351,Phycidae_Urophycis_chuss:5.240351):28.607969,(Lotidae_Gaidropsarus_ensis:29.800286,(Lotidae_Lota_lota:23.935071,(Gadidae_Melanogrammus_aeglefinus:5.86839,Gadidae_Gadus_morhua:5.86839):18.066681):5.865215):4.048034):4.41161):3.240071):4.155085):33.144915):28.3):27.890331):15.309669):11.08283,(Polymixiidae_Polymixia_japonica:154.800001,(((Diretmidae_Diretmus_argenteus:53.99855,(Monocentridae_Monocentris_japonica:33.600001,((Trachichthyidae_Hoplostethus_occidentalis_atlanticus:24.361165,Trachichthyidae_Gephyroberyx_darwinii:24.361165):7.919156,(Anoplogastridae_Anoplogaster_cornuta:31.22776,Trachichthyidae_Paratrachichthys_sajademalensis:31.22776):1.052561):1.31968):20.398549):71.275924,((Berycidae_Beryx_decadactylus:70.612394,(Melamphaidae_Poromitra_crassiceps:39.138751,(Melamphaidae_Scopelogadus_beanii:32.603956,Melamphaidae_Melamphaes_suborbitalis:32.603956):6.534795):31.473643):40.295775,(((Rondeletiidae_Rondeletia_bicolor:18.616456,Rondeletiidae_Rondeletia_loricata:18.616456):36.662305,Barbourisiidae_Barbourisia_rufa:55.278761):34.605685,(Cetomimidae_Cetostoma_regani:55.492218,(Cetomimidae_Cetomimus_craneae:10.537863,Cetomimidae_Cetomimus_compuctus:10.537863):44.954355):34.392228):21.023723):14.366305):21.189147,((Holocentridae_Sargocentron_diadema:25.43456,Holocentridae_Holocentrus_rufus:25.43456):119.565441,((Ophidiidae_Brotula_multibarbata:66.227334,(Ophidiidae_Lepophidium_profundorum:23.036156,(Ophidiidae_Genypterus_blacodes:21.590629,Ophidiidae_Ophidion_holbrookii:21.590629):1.445527):43.191178):66.572667,((Batrachoididae_Porichthys_notatus:39.800001,Batrachoididae_Opsanus_tau:39.800001):87,(((Kurtidae_Kurtus_indicus:80.300001,Apogonidae_Apogon_campbelli:80.300001):22,(Eleotridae_Eleotris_acanthopoma_pisonis:64.430477,(Gobiidae_Gobiosoma_bosc:45.630567,Microdesmidae_Microdesmus_longipinnis:45.630567):18.79991):37.869524):19.3,((((Dactylopteridae_Dactylopterus_volitans:68.121568,(Aulostomidae_Aulostomus_maculatus:3.200001,Aulostomidae_Aulostomus_chinensis:3.200001):64.921567):6.178433,((Fistulariidae_Fistularia_petimba:65.463647,Mullidae_Mullus_auratus:65.463647):6.26906,(Callionymidae_Callionymus_sp_bairdi:69.154942,Syngnathidae_Syngnathus_fuscus:69.154942):2.577765):2.567294):20.27,(Chiasmodontidae_Chiasmodon_sp:43.900001,((Centrolophidae_Icichthys_lockingtoni:34.370774,Pomatomidae_Pomatomus_saltatrix:34.370774):6.934458,(((Ariommatidae_Ariomma_bondi:25.644121,Nomeidae_Psenes_cyanophrys:25.644121):5.370208,Scombridae_Auxis_rochei:31.014329):6.588438,((Stromateidae_Peprilus_paru:35.602767,(Gempylidae_Paradiplospinus_gracilis:31.499638,Bramidae_Brama_brama:31.499638):4.103129):1,(Icosteidae_Icosteus_aenigmaticus:33.972252,Trichiuridae_Trichiurus_lepturus:33.972252):2.630515):1):3.702465):2.594769):50.67):21.916687,((((((Synbranchidae_Monopterus_albus:69.146846,Indostomidae_Indostomus_paradoxus:69.146846):4.548479,(Mastacembelidae_Macrognathus_siamensis:18.057555,Mastacembelidae_Mastacembelus_erythrotaenia:18.057555):55.63777):7.004676,((Anabantidae_Ctenopoma_acutirostre_kingsleyae:62.782323,(Helostomatidae_Helostoma_temminkii:59.687826,Osphronemidae_Trichopodus_pectoralis:59.687826):3.094497):7.86905,(Channidae_Channa_striata:67.428514,Nandidae_Nandus_nandus:67.428514):3.222859):10.048628):15.7,((Nematistiidae_Nematistius_pectoralis:60.067614,((Coryphaenidae_Coryphaena_hippurus:19.124214,Rachycentridae_Rachycentron_canadum:19.124214):12.775787,(Echeneidae_Remora_osteochir_australis:10.178122,Echeneidae_Echeneis_naucrates:10.178122):21.721879):28.167613):9.732387,((Sphyraenidae_Sphyraena_sphyraena:65.900885,(Menidae_Mene_maculata:60.339531,Polynemidae_Polydactylus_octonemus:60.339531):5.561354):2.899116,((((Leptobramidae_Leptobrama_muelleri:46.58559,Toxotidae_Toxotes_jaculatrix:46.58559):5.096327,(Xiphiidae_Xiphias_gladius:27.833842,Istiophoridae_Istiophorus_platypterus:27.833842):23.848075):5.298938,Carangidae_Trachinotus_falcatus:56.980855):4.861848,((Eolates_gracilis:1,(Centropomidae_Psammoperca_waigiensis:33.068144,(Centropomidae_Lates_calcarifer:13.595548,Centropomidae_Lates_microlepis:13.595548):19.472596):18.48802894):9.286530063,(Heteronectes_chaneti:5.287518047,(Amphistium_paradoxum:9.20486446,(Psettodidae_Psettodes_erumei:57.842703,(Citharidae_Lepidoblepharon_ophthalmolepis:53.600001,((Cynoglossidae_Cynoglossus_interruptus:42.400002,(Soleidae_Solea_solea:24.944331,Soleidae_Soleichthys_heterorhinos:24.944331):17.455671):9.674607,((Scophthalmidae_Scophthalmus_aquosus:23.305906,Scophthalmidae_Lepidorhombus_boscii:23.305906):26.973285,((Paralichthyidae_Paralichthys_dentatus:40.749719,Bothidae_Bothus_lunatus:40.749719):4.387561,(Paralichthyidae_Pseudorhombus_pentophthalmus:21.404631,(((Pleuronectidae_Glyptocephalus_zachirus:5.096079,Pleuronectidae_Microstomus_pacificus:5.096079):2.538752,(Pleuronectidae_Lyopsetta_exilis:6.085211,Pleuronectidae_Hippoglossus_hippoglossus:6.085211):1.54962):2.215451,(Pleuronectidae_Limanda_limanda:8.798272,((Pleuronectidae_Platichthys_stellatus:3.717184,Pleuronectidae_Pleuronectes_platessa:3.717184):3.081088,(Pleuronectidae_Lepidopsetta_bilineata:4.798272,Pleuronectidae_Psettichthys_melanostictus:4.798272):2):2):1.05201):11.554349):23.732649):5.141911):1.795418):1.525392):4.242702):1):1):1):1):6.957298):1):26.6):12.043384,(((Polycentridae_Polycentrus_schomburgkii:94.214904,(Pholidichthyidae_Pholidichthys_leucotaenia:88.700001,Cichlidae_Cichla_temensis:88.700001):5.514903):1,(((Atherinopsidae_Atherinopsis_californiensis:48.715388,Atherinopsidae_Menidia_menidia:48.715388):22.225577,(Isonidae_Iso_sp:49.317292,(Atherinidae_Atherinomorus_stipes:43.78646,(Melanotaeniidae_Melanotaenia_splendida:39.870052,Pseudomugilidae_Pseudomugil_gertrudae:39.870052):3.916408):5.530832):21.623673):6.46052,(((Poeciliidae_Poecilia_latipinna_reticulata:14.292152,(Poeciliidae_Heterandria_formosa:11.366823,(Poeciliidae_Gambusia_affinis:5.736451,Poeciliidae_Belonesox_belizanus:5.736451):5.630372):2.925329):30.13151,(Cyprinodontidae_Cyprinodon_variegatus:40.469994,Fundulidae_Fundulus_parvipinnis:40.469994):3.953668):31.977823,(Adrianichthyidae_Oryzias_latipes:71.878571,((Zenarchopteridae_Dermogenys_collettei:29.883878,((Belonidae_Tylosurus_crocodilus:6.568056,Belonidae_Ablennes_hians:6.568056):17.429484,(Scomberesocidae_Cololabis_saira:2.646712,Scomberesocidae_Scomberesox_saurus:2.646712):21.350828):5.886338):17.178962,(Hemiramphidae_Hyporhamphus_affinis:36.892311,((Hemiramphidae_Hemiramphus_brasiliensis:13.391766,Hemiramphidae_Oxyporhamphus_micropterus:13.391766):10.259915,((Exocoetidae_Cypselurus_callopterus:6.413654,Exocoetidae_Exocoetus_monocirrhus:6.413654):4.605967,Exocoetidae_Cheilopogon_pinnatibarbatus:11.019621):12.63206):13.24063):10.170529):24.815731):4.522914):1):17.813419):1.999954,((Pseudochromidae_Halidesmus_scapularis:84.850287,Pomacentridae_Pomacentrus_brachialis:84.850287):8.187103,(Embiotocidae_Cymatogaster_aggregata:91.529056,(Mugilidae_Mugil_cephalus:89.757655,(Plesiopidae_Plesiops_coeruleolineatus:86.977996,(Opistognathidae_Opistognathus_maxillosus:79.196597,(((Gobiesocidae_Lepadichthys_lineatus:37.560081,Gobiesocidae_Gobiesox_maeandricus:37.560081):35.985217,Tripterygiidae_Enneanectes_altivelis:73.545298):3.122761,((Clinidae_Clinus_cottoides:50.741024,(Dactyloscopidae_Platygillellus_rubrocinctus:45.641062,Chaenopsidae_Chaenopsis_alepidota:45.641062):5.099962):17.520576,(Blenniidae_Stanulus_seychellensis:31.042358,Blenniidae_Blenniella_cyanostigma:31.042358):37.219242):8.406459):2.528538):7.781399):2.779659):1.771401):1.508334):4.177468):11.228527):3.450865,(Gerreidae_Eucinostomus_argenteus:108.000002,(((Labridae_Lachnolaimus_maximus:59.822938,Odacidae_Haletta_semifasciata:59.822938):16.826114,Scaridae_Scarus_globiceps:76.649052):30.35095,(((Uranoscopidae_Uranoscopus_sulphureus:83.996275,(Ammodytidae_Ammodytes_hexapterus:78.584442,Pinguipedidae_Parapercis_punctulata:78.584442):5.411833):11.903726,(((Acropomatidae_Acropoma_japonicum:90.360051,((Percophidae_Acanthaphritis_unoorum:66.16617,Creediidae_Limnichthys_sp:66.16617):18.796708,(Glaucosomatidae_Glaucosoma_buergeri:66.617288,Pempheridae_Pempheris_schomburgkii:66.617288):18.34559):5.397173):2.814411,(((Oplegnathidae_Oplegnathus_punctatus:61.206964,Kuhliidae_Kuhlia_rupestris:61.206964):7.298332,Kyphosidae_Kyphosus_sectatrix:68.505296):16.157201,(Percichthyidae_Percichthys_trucha:69.999181,((Cirrhitidae_Cirrhitichthys_falco:66.467051,Cheilodactylidae_Cheilodactylus_fasciatus:66.467051):2.53213,(Enoplosidae_Enoplosus_armatus:63.04721,Centrarchidae_Acantharchus_pomotis:63.04721):5.951971):1):14.663316):8.511965):1.725539,(Serranidae_Pseudogramma_polyacantha:82.200001,(Percidae_Perca_fluviatilis:78.577195,((Bovichtidae_Bovichtus_diacanthus:63.021097,(Nototheniidae_Notothenia_coriiceps:9.671082,(Harpagiferidae_Harpagifer_antarcticus:8.390321,(Bathydraconidae_Gymnodraco_acuticeps:7.390321,Channichthyidae_Chionodraco_rastrospinosus:7.390321):1):1.280761):53.350015):12.936917,(((Peristediidae_Peristedion_ecuadorense:54.532565,Triglidae_Prionotus_carolinus:54.532565):11.953546,(Synanceiidae_Synanceia_verrucosa:62.510934,Scorpaenidae_Scorpaenodes_guamensis:62.510934):3.975177):6.621302,(Platycephalidae_Platycephalus_indicus:70.795272,(Anoplopomatidae_Anoplopoma_fimbria:46.504594,((Bathymasteridae_Rathbunella_hypoplecta:22.798552,(Stichaeidae_Cebidichthys_violaceus:15.58542,(Zoarcidae_Zoarces_americanus_viviparus:12.493576,(Anarhichadidae_Anarrhichthys_ocellatus:9.933504,(Zaproridae_Zaprora_silenus:8.933504,Cryptacanthodidae_Cryptacanthodes_maculatus:8.933504):1):2.560072):3.091844):7.213132):19.028758,(((Aulorhynchidae_Aulorhynchus_flavidus:25.280385,Hypoptychidae_Hypoptychus_dybowskii:25.280385):1.019618,((Gasterosteidae_Culaea_inconstans:19.844899,(Gasterosteidae_Apeltes_quadracus:17.136644,Gasterosteidae_Spinachia_spinachia:17.136644):2.708255):1.782129,(Gasterosteidae_Pungitius_pungitius:16.827665,Gasterosteidae_Gasterosteus_aculeatus:16.827665):4.799363):4.672975):13.53479,(Hexagrammidae_Hexagrammos_decagrammus:27.818969,((Cyclopteridae_Cyclopterus_lumpus:20.762394,Liparidae_Liparis_pulchellus:20.762394):4.037609,(Agonidae_Hypsagonus_quadricornis:20.371382,(Cottidae_Cottus_carolinae:18.19359,Psychrolutidae_Psychrolutes_phrictus:18.19359):2.177792):4.428621):3.018966):12.015824):1.992517):4.677284):24.290678):2.312141):2.850601):2.619181):3.622806):12.7):1):10.100001,((Drepaneidae_Drepane_punctata:67.658011,Ephippidae_Chaetodipterus_faber:67.658011):37.341991,(((Lobotidae_Lobotes_pacificus_surinamensis:86.438004,Sciaenidae_Menticirrhus_undulatus_littoralis:86.438004):5.761997,((Monodactylidae_Monodactylus_sebae:70.333247,(Avitoluvarus_eocaenicus:22.1011057,(Zanclidae_Zanclus_cornutus:55.859022,Acanthuridae_Acanthurus_triostegus:55.859022):4.740979):9.733246):15.366754,((Pomacanthidae_Pomacanthus_semicirculatus:54.434118,(Leiognathidae_Leiognathus_equulus:32.700001,Chaetodontidae_Chelmon_rostratus:32.700001):21.734117):17.438962,(Emmelichthyidae_Erythrocles_schlegelii:69.871718,(Malacanthidae_Malacanthus_plumieri:68.303733,(Haemulidae_Haemulon_aurolineatum:64.600001,Lutjanidae_Lutjanus_griseus:64.600001):3.703732):1.567985):2.001362):13.826921):6.5):11.800001,((Sillaginidae_Sillago_sihama:85.853361,(Nemipteridae_Pentapodus_caninus:80.270573,(Lethrinidae_Lethrinus_erythropterus:74.370539,Sparidae_Stenotomus_chrysops:74.370539):5.900034):5.582788):17.146641,(Siganidae_Siganus_spinus:102.000002,((Scatophagidae_Scatophagus_argus:75.860371,Priacanthidae_Heteropriacanthus_cruentatus:75.860371):25.139631,((Caproidae_Antigonia_rubescens:10.905674,Caproidae_Antigonia_capros:10.905674):89.094328,(((Lophiidae_Lophiodes_reticulatus:22.462185,Lophiidae_Lophius_gastrophysus:22.462185):42.032119,((Antennariidae_Histrio_histrio:12.333102,Antennariidae_Antennatus_coccineus:12.333102):33.466899,(Chaunacidae_Chaunax_suttkusi:38.499563,(Gigantactinidae_Gigantactis_sp:32.911618,((Ceratiidae_Cryptopsaras_couesii:20.548708,Ceratiidae_Ceratias_holboelli:20.548708):10.416157,((Himantolophidae_Himantolophus_albinares_sagamius:14.456802,Melanocetidae_Melanocetus_murrayi:14.456802):9.124159,(Oneirodidae_Oneirodes_macrosteus:10.164357,Oneirodidae_Dolopichthys_sp:10.164357):13.416604):7.383904):1.946753):5.587945):7.300438):18.694303):34.505698,(((Prohollardia_avita:37.96354313,(Protacanthodes_nimesensis:1,Triacanthidae_Triacanthus_biaculeatus:48.67547183):15.81749917):10.123893,(Aracanidae_Aracana_aurita:54.800001,(Ostraciidae_Ostracion_cubicus:51.000002,Ostraciidae_Rhinesomus_triqueter:51.000002):3.799999):19.816863):3.183137,(((Diodontidae_Diodon_holocanthus:12.198387,Diodontidae_Chilomycterus_schoepfii:12.198387):43.701614,(Tetraodontidae_Canthigaster_bennetti:24.22571,Tetraodontidae_Tetraodon_miurus:24.22571):31.674291):17.334859,((Molidae_Ranzania_laevis:22,Molidae_Mola_mola:22):46.61027,((Monacanthidae_Aluterus_scriptus:27.823604,Monacanthidae_Stephanolepis_hispidus:27.823604):15.976396,(Balistidae_Xanthichthys_ringens:20.7,(Balistidae_Balistes_vetula:19.7,Balistidae_Sufflamen_fraenatum:19.7):1):23.1):24.81027):4.62459):4.565141):21.200001):1):1):1):1):1):1):1):1):1):3.894248):4.592438):5.113313):5.2):6):12.2):1.46362):8.33638):6.58283):11.71717):9.7):9.5):19):3.553787):17.946213):17.8):23.2):9.5):1):1):37.2):1):1):1,(Fukangichthys_longidorsalis:9.043114961,(Scanilepis_dubia:50.73809953,Evenkia_eunotoptera:1):1):71.33938143):1,Australosomus:74.56836799):1):22.6):1):7.076809193):1):10.82685215):16.70293699):1):1):1):1):4.29340267):1):47);

((Guiyu_oneiros:1,(Onychodus_jandemarrai:31.26751552,((Diplocercides:1,(Latimeriidae_Latimeria_chalumnae:330.7068239,Rhabdoderma:1):34.18780614):47.62099694,((Neoceratodontidae_Neoceratodus_forsteri:279.7982796,Lepidosirenidae_Lepidosiren_paradoxa:279.7982796):128.2017204,((Osteolepis_macrolepidotus:1,Gyroptychius_milleri:12.92828317):1,Eusthenopterus_foordi:29.2595873):1.186100626):4.515627):1):12.22903945):17.25533555,((Cheirolepis_trailli:1,Cheirolepis_schultzei:7.051312102):2.688678504,((Polypteridae_Erpetoichthys_calabaricus:29.200001,Polypteridae_Polypterus_senegalus:29.200001):365.800001,(Osorioichthys_marginis:24.16181673,((Tegeolepis_clarki:16.14178313,Howqualepis_rostridens:1):2.427532781,((Gogosardina_coatesi:4.03190139,(Mimipiscis_bartrami:3.432232472,Mimipiscis_toombsi:1):1):5.442399971,((Moythomasia_lineata:1,Moythomasia_durgaringa:5.945937854):1.084797727,(Stegotrachelus_finlayi:1,(Limnomis_delaneyi:1,(Wendyichthys_dicksoni:30.33341435,(Kentuckia_deani:1,((Mesopoma_planti:6.579044215,Mesopoma_carricki:1):4.227470539,((Birgeria_stensioei:1,(Chondrosteus_acipenseroides:1,(Saurichthys_dawaziensis:1,((Protopsephurus_liui:1,Polyodontidae_Polyodon_spathula:130.3192194):8.580782591,(Acipenseridae_Acipenser_fulvescens:38.579335,(Acipenseridae_Scaphirhynchus_platorynchus:19.375515,Acipenseridae_Scaphirhynchus_albus:19.375515):19.20382):100.320667):22.9090863):39.84072101):51.4740942):96.97609749,(Boreosomus:74.65471942,((Australosomus:2.833582162,(Fukangichthys_longidorsalis:9.795273083,(Scanilepis_dubia:44.53284058,Evenkia_eunotoptera:1):1):1):70.59054164,(Perleidus_altolepis:82.94061439,(Luganoia_lepidosteoides:91.91263846,(((Macrosemius_fourneti:92.96653276,((Lepisosteidae_Lepisosteus_osseus:200.9253998,Semionotus_elegans:1):45.40702261,Kyphosichthys_grandei:1):1):20.56757856,(Watsonulus_eugnathoides:1,(Amblysemius:1,(Pachyamia_latimaxillaris:2.552246202,(Tomognathus_mordax:1,Amiidae_Amia_calva:100.9841548):1):50.59877569):100.5146162):14.80245431):54.6,(Pachycormus:103.6946336,(Crossognathus_danubiensis:177.0845905,(((Anaethalion_angustus:1,Anaethalion_knorri:4.978083064):45.39280423,((Megalopidae_Megalops_atlanticus:133.565966,Elopidae_Elops_saurus:133.565966):62.634035,(Albulidae_Albula_vulpes:150.800001,((Notacanthidae_Notacanthus_chemnitzii:50.669287,(Halosauridae_Halosauropsis_macrochir:40.366211,Halosauridae_Aldrovandia_affinis:40.366211):10.303076):50.330714,(((Eurypharyngidae_Eurypharynx_pelecanoides:25.866364,Saccopharyngidae_Saccopharynx_ampullaceus:25.866364):44.322494,(Nemichthyidae_Nemichthys_scolopaceus:58.739485,Anguillidae_Anguilla_rostrata:58.739485):11.449373):9.198895,(Serrivomeridae_Serrivomer_beanii:76.053487,(Congridae_Conger_oceanicus:65.416147,(Muraenesocidae_Muraenesox_cinereus:57.193624,Ophichthidae_Myrichthys_maculosus:57.193624):8.222523):10.63734):3.334266):21.612248):49.8):45.4):1):86.1,((Lycoptera_davidi:97.82340508,(Paralycoptera_wui:108.5461124,(Xixiaichthys_tongxinens:100.1596163,(((Eohiodon_woodruffi:4.966481665,Eohiodon_rosei:1):1,(Hiodon_consteniorum:1,(Hiodontidae_Hiodon_tergisus:9.520291,Hiodontidae_Hiodon_alosoides:9.520291):26.4693173):20.12147281):170.9889199,(Chauliopareion_mahengeense:121.7500014,(Pantodontidae_Pantodon_buchholzi:163.100001,(((Arapaimidae_Arapaima_gigas:79.3,Osteoglossidae_Osteoglossum_bicirrhosum:79.3):25.549112,(Brychaetus_muelleri:1,Phareodus_encaustus:11.85645414):49.49790616):43.475578,(Notopteridae_Xenomystus_nigri:120.300002,Gymnarchidae_Gymnarchus_niloticus:120.300002):28.024688):14.775311):1):63):1):1):1):43.7,((((Diplomystus_brevissimus:47.23513317,Sorbinichthys_africanus:1):90.78106759,(Denticipitidae_Denticeps_clupeoides:188.900001,(Chirocentridae_Chirocentrus_dorab:87.096454,((Engraulidae_Coilia_nasus:47.06527,Engraulidae_Engraulis_mordax_eurystole:47.06527):33.347234,((Pristigasteridae_Pellona_flavipinnis:27.450803,Pristigasteridae_Ilisha_elongata:27.450803):46.430359,(Clupeidae_Alosa_pseudoharengus:54.138864,Clupeidae_Dorosoma_cepedianum:54.138864):19.742298):6.531342):6.68395):101.803547):1):40.3,((Alepocephalidae_Talismania_bifurcata:53.266521,((Alepocephalidae_Bathylaco_nigricans:33.802485,Alepocephalidae_Alepocephalus_tenebrosus:33.802485):10.988666,((Alepocephalidae_Rouleina_attrita:22.275085,Alepocephalidae_Xenodermichthys_copei:22.275085):19.924916,Platytroctidae_Sagamichthys_abei:42.200001):2.59115):8.47537):166.430015,((Gonorynchidae_Gonorynchus_abbreviatus:175.900001,(Mahengichthys_singidaensis:101.1524657,Chanidae_Chanos_chanos:147.100001):28.8):22.7,((((Gyrinocheilidae_Gyrinocheilus_sp:70.111694,(Catostomidae_Hypentelium_nigricans:23.594381,(Catostomidae_Carpiodes_carpio:16.355089,Catostomidae_Ictiobus_bubalus:16.355089):7.239292):46.517313):8.688307,Cobitidae_Cobitis_taenia:78.800001):20.5,(Cyprinidae_Danio_rerio:63.300001,(((Cyprinidae_Zacco_sieboldii_platypus:12.727958,Cyprinidae_Opsariichthys_uncirostris_bidens:12.727958):14.610086,((Cyprinidae_Xenocypris_argentea:12.904177,Cyprinidae_Hypophthalmichthys_molitrix:12.904177):2.917503,(Cyprinidae_Luciobrama_macrocephalus:13.439618,(Cyprinidae_Squaliobarbus_curriculus:11.472717,Cyprinidae_Mylopharyngodon_piceus:11.472717):1.966901):2.382062):11.516364):12.401957,(Cyprinidae_Tanakia_lanceolata_himantegus:35.004261,(Cyprinidae_Notemigonus_crysoleucas:22.933404,(Cyprinidae_Semotilus_atromaculatus:21.325215,((Cyprinidae_Campostoma_oligolepis:12.800846,Cyprinidae_Rhinichthys_cataractae:12.800846):4.408707,(Cyprinidae_Phenacobius_uranops:12.822623,(Cyprinidae_Pimephales_promelas_notatus:9.460336,(Cyprinidae_Luxilus_coccogenis:6.738861,Cyprinidae_Notropis_asperifrons:6.738861):2.721475):3.362287):4.38693):4.115662):1.608189):12.070857):4.73574):23.56):36):73.6,((Gymnotidae_Electrophorus_electricus:63.909447,Gymnotidae_Gymnotus_sp:63.909447):83.924067,(((Distichodontidae_Distichodus_maculatus:103.604365,Citharinidae_Citharinus_congicus:103.604365):11.170138,(Alestidae_Alestes_baremoze:106.666979,((Parodontidae_Parodon_nasus:68.800001,Hemiodontidae_Hemiodus_immaculatus:68.800001):22.415197,((Bryconidae_Brycon_pesu:66.003418,(Gasteropelecidae_Thoracocharax_stellatus:27.746828,Gasteropelecidae_Gasteropelecus_sternicla:27.746828):38.25659):1.227196,Characidae_Astyanax_mexicanus:67.230614):23.984584):15.451781):8.107524):22.236489,((Nematogenyidae_Nematogenys_inermis:110.648758,(Trichomycteridae_Trichomycterus_sp:105.886466,((Loricariidae_Loricaria_simillima:70.313425,Astroblepidae_Astroblepus_sp:70.313425):28.122359,(Callichthyidae_Callichthys_callichthys:69.200001,Callichthyidae_Corydoras_trilineatus:69.200001):29.235783):7.450682):4.762292):5.151243,(Diplomystidae_Diplomystes_nahuelbutaensis:106.203801,((Clariidae_Clarias_batrachus:47.60069,Heteropneustidae_Heteropneustes_fossilis:47.60069):38.718856,(((Sisoridae_Bagarius_yarrelli:55.522058,(Amblycipitidae_Liobagrus_aequilabris:48.027606,Akysidae_Akysis_sp:48.027606):7.494452):21.358911,(Schilbeidae_Pseudeutropius_brachypopterus:74.448774,(Bagridae_Mystus_bocourti:36.077943,Bagridae_Bagrus_ubangensis:36.077943):38.370831):2.432195):8.408138,(((Chacidae_Chaca_sp:72.429482,Cetopsidae_Cetopsis_coecutiens:72.429482):10.714797,(Cranoglanididae_Cranoglanis_bouderius:68.700001,Ictaluridae_Ictalurus_punctatus:68.700001):14.444278):1.039619,(Pangasiidae_Pangasianodon_hypophthalmus:82.158061,((Amphiliidae_Amphilius_jacksonii:69.199757,(Malapteruridae_Malapterurus_beninensis:64.740321,Mochokidae_Synodontis_batesii:64.740321):4.459436):10.993535,((Plotosidae_Plotosus_lineatus:73.039645,Siluridae_Hemisilurus_moolenburghi:73.039645):5.503862,(Auchenipteridae_Ageneiosus_atronasus:51.101654,Doradidae_Anduzedoras_oxyrhynchus:51.101654):27.441853):1.649785):1.964769):2.025837):1.105209):1.030439):19.884255):9.5962):21.210991):10.822522):25.066487):25.7):21.096535):10.503465):20.4,(Orthogonikleithrus_francogalliensis:77.14330024,(((Bathylagidae_Bathylagus_euryops:70.542512,(Microstomatidae_Nansenia_longicauda_ardesiaca:51.394,(Opisthoproctidae_Macropinna_microstoma:33.11268,Argentinidae_Argentina_sialis_silus:33.11268):18.28132):19.148512):89.057489,((Galaxiidae_Neochanna_burrowsius:34.505049,Galaxiidae_Galaxias_maculatus:34.505049):111.121934,(((Umbridae_Novumbra_hubbsi:55.762491,Umbridae_Umbra_limi:55.762491):23.63751,(Esocidae_Esox_lucius:31.366195,Esocidae_Esox_americanus:31.366195):48.033806):25.064928,(Salmonidae_Coregonus_clupeaformis:35.300001,(Salmonidae_Thymallus_brevirostris:32.456522,(Salmonidae_Oncorhynchus_nerka_mykiss:19.929533,(Salmonidae_Salvelinus_alpinus:16.737138,Salmonidae_Salmo_salar:16.737138):3.192395):12.526989):2.843479):69.164928):41.162054):13.973018):55.253787,(((Retropinnidae_Retropinna_semoni:73.700001,(((Osmeridae_Osmerus_mordax:10.332899,Osmeridae_Thaleichthys_pacificus:10.332899):5.872101,(Osmeridae_Mallotus_villosus:14.961758,Osmeridae_Hypomesus_pretiosus:14.961758):1.243242):18.133836,(Plecoglossidae_Plecoglossus_altivelis:28.70318,Salangidae_Neosalangichthys_ishikawae:28.70318):5.635656):39.361165):55.7,((Diplophidae_Diplophos_taenia:77.330737,((Gonostomatidae_Bonapartia_pedaliota:54.297445,Gonostomatidae_Margrethia_obtusirostra:54.297445):20.014374,(Gonostomatidae_Gonostoma_elongatum:69.948163,Gonostomatidae_Cyclothone_microdon:69.948163):4.363656):3.018918):6.469264,(Phosichthyidae_Pollichthys_mauli:77.722827,((Sternoptychidae_Argyropelecus_gigas:25.230481,Sternoptychidae_Maurolicus_weitzmani:25.230481):47.329876,(Stomiidae_Chauliodus_macouni_danae:63.126299,(Stomiidae_Stomias_boa:36.381906,(Stomiidae_Chirostomias_pliopterus:31.578039,((Stomiidae_Melanostomias_margaritifer:21.790927,Stomiidae_Leptostomias_longibarba:21.790927):6.240428,((Stomiidae_Photonectes_margarita:19.053467,Stomiidae_Tactostoma_macropus:19.053467):7.977888,(Stomiidae_Malacosteus_niger:25.031355,(Stomiidae_Eustomias_polyaster:24.031355,(Stomiidae_Aristostomias_scintillans:23.031355,Stomiidae_Bathophilus_flemingi_pawneei:23.031355):1):1):2):1):3.546684):4.803867):26.744393):9.434058):5.16247):6.077174):45.6):81.9,((Ateleopodidae_Ateleopus_japonicus:8.089622,Ateleopodidae_Ijimaia_antillarum:8.089622):184.210379,(((Synodontidae_Trachinocephalus_myops:34.928634,Synodontidae_Synodus_foetens:34.928634):78.971367,((Paraulopidae_Paraulopus_oblongus:101.985288,(Synodontidae_Saurida_gracilis:50.257509,Synodontidae_Harpadon_microchir:50.257509):51.727779):8.326796,(((Aulopidae_Aulopus_filamentosus:47.160387,Bathysauridae_Bathysaurus_ferox:47.160387):10.843902,(Ipnopidae_Ipnops_murrayi:13.82,Ipnopidae_Ipnops_agassizi:13.82):44.184289):23.635948,((Ipnopidae_Bathypterois_atricolor:69.328167,(Giganturidae_Gigantura_indica:13.525058,Giganturidae_Gigantura_chuni:13.525058):55.803109):6.462647,(Scopelarchidae_Benthalbella_macropinna:62.490696,(Chlorophthalmidae_Chlorophthalmus_agassizi:61.13635,(Sudidae_Sudis_atrox:55.026295,((Alepisauridae_Anotopterus_pharao:45.661943,(Alepisauridae_Omosudis_lowii:36.284216,Alepisauridae_Alepisaurus_ferox:36.284216):9.377727):8.238175,(Paralepididae_Stemonosudis_intermedia_macrura:50.122687,(Evermannellidae_Evermannella_balbo:46.686813,Paralepididae_Paralepis_coregonoides:46.686813):3.435874):3.777431):1.126177):6.110055):1.354346):13.300118):5.849423):28.671847):3.587917):68.9,(((Neoscopelidae_Neoscopelus_macrolepidotus:42.408365,Neoscopelidae_Scopelengys_tristis:42.408365):31.191636,((Myctophidae_Lampadena_speculigera:39.81673,Myctophidae_Lampanyctus_macdonaldi:39.81673):11.872,(Myctophidae_Benthosema_glaciale:28.30241,Myctophidae_Myctophum_punctatum:28.30241):23.38632):21.911271):99.5,(((Lamprididae_Lampris_guttatus:65.552505,(Regalecidae_Regalecus_russelii:37.682943,Trachipteridae_Trachipterus_trachypterus:37.682943):27.869562):84.747496,(((Mcconichthys_longipinnis:1,(Aphredoderidae_Aphredoderus_sayanus:41.500001,Amblyopsidae_Chologaster_cornuta:41.500001):25.1887275):1,(Lateopisciculus_turrifumosus:1,(Percopsidae_Percopsis_omiscomaycus:14.617149,Percopsidae_Percopsis_transmontana:14.617149):44.21729418):8.854285313):67.3016035,(Zeidae_Zeus_faber:107.100001,(Stylephoridae_Stylephorus_chordatus:78.800001,(Merlucciidae_Merluccius_productus:45.655086,(((Macrouridae_Gadomus_dispar:9.947595,Macrouridae_Bathygadus_favosus:9.947595):28.939269,(Steindachneriidae_Steindachneria_argentea:35.929071,((Macrouridae_Malacocephalus_laevis:17.761718,Macrouridae_Trachonurus_sulcatus:17.761718):10.126413,(Macrouridae_Coelorinchus_caribbaeus:21.883517,Macrouridae_Coryphaenoides_armatus:21.883517):6.004614):8.04094):2.957793):2.613137,((Moridae_Laemonema_goodebeanorum:25.08833,(Moridae_Halargyreus_johnsonii:16.444614,(Moridae_Lepidion_ensiferus:5.35693,Moridae_Antimora_rostrata:5.35693):11.087684):8.643716):13.1716,((Phycidae_Urophycis_tenuis:5.240351,Phycidae_Urophycis_chuss:5.240351):28.607969,(Lotidae_Gaidropsarus_ensis:29.800286,(Lotidae_Lota_lota:23.935071,(Gadidae_Melanogrammus_aeglefinus:5.86839,Gadidae_Gadus_morhua:5.86839):18.066681):5.865215):4.048034):4.41161):3.240071):4.155085):33.144915):28.3):27.890331):15.309669):11.08283,(Polymixiidae_Polymixia_japonica:154.800001,(((Diretmidae_Diretmus_argenteus:53.99855,(Monocentridae_Monocentris_japonica:33.600001,((Trachichthyidae_Hoplostethus_occidentalis_atlanticus:24.361165,Trachichthyidae_Gephyroberyx_darwinii:24.361165):7.919156,(Anoplogastridae_Anoplogaster_cornuta:31.22776,Trachichthyidae_Paratrachichthys_sajademalensis:31.22776):1.052561):1.31968):20.398549):71.275924,((Berycidae_Beryx_decadactylus:70.612394,(Melamphaidae_Poromitra_crassiceps:39.138751,(Melamphaidae_Scopelogadus_beanii:32.603956,Melamphaidae_Melamphaes_suborbitalis:32.603956):6.534795):31.473643):40.295775,(((Rondeletiidae_Rondeletia_bicolor:18.616456,Rondeletiidae_Rondeletia_loricata:18.616456):36.662305,Barbourisiidae_Barbourisia_rufa:55.278761):34.605685,(Cetomimidae_Cetostoma_regani:55.492218,(Cetomimidae_Cetomimus_craneae:10.537863,Cetomimidae_Cetomimus_compuctus:10.537863):44.954355):34.392228):21.023723):14.366305):21.189147,((Holocentridae_Sargocentron_diadema:25.43456,Holocentridae_Holocentrus_rufus:25.43456):119.565441,((Ophidiidae_Brotula_multibarbata:66.227334,(Ophidiidae_Lepophidium_profundorum:23.036156,(Ophidiidae_Genypterus_blacodes:21.590629,Ophidiidae_Ophidion_holbrookii:21.590629):1.445527):43.191178):66.572667,((Batrachoididae_Porichthys_notatus:39.800001,Batrachoididae_Opsanus_tau:39.800001):87,(((Kurtidae_Kurtus_indicus:80.300001,Apogonidae_Apogon_campbelli:80.300001):22,(Eleotridae_Eleotris_acanthopoma_pisonis:64.430477,(Gobiidae_Gobiosoma_bosc:45.630567,Microdesmidae_Microdesmus_longipinnis:45.630567):18.79991):37.869524):19.3,((((Dactylopteridae_Dactylopterus_volitans:68.121568,(Aulostomidae_Aulostomus_maculatus:3.200001,Aulostomidae_Aulostomus_chinensis:3.200001):64.921567):6.178433,((Fistulariidae_Fistularia_petimba:65.463647,Mullidae_Mullus_auratus:65.463647):6.26906,(Callionymidae_Callionymus_sp_bairdi:69.154942,Syngnathidae_Syngnathus_fuscus:69.154942):2.577765):2.567294):20.27,(Chiasmodontidae_Chiasmodon_sp:43.900001,((Centrolophidae_Icichthys_lockingtoni:34.370774,Pomatomidae_Pomatomus_saltatrix:34.370774):6.934458,(((Ariommatidae_Ariomma_bondi:25.644121,Nomeidae_Psenes_cyanophrys:25.644121):5.370208,Scombridae_Auxis_rochei:31.014329):6.588438,((Stromateidae_Peprilus_paru:35.602767,(Gempylidae_Paradiplospinus_gracilis:31.499638,Bramidae_Brama_brama:31.499638):4.103129):1,(Icosteidae_Icosteus_aenigmaticus:33.972252,Trichiuridae_Trichiurus_lepturus:33.972252):2.630515):1):3.702465):2.594769):50.67):21.916687,((((((Synbranchidae_Monopterus_albus:69.146846,Indostomidae_Indostomus_paradoxus:69.146846):4.548479,(Mastacembelidae_Macrognathus_siamensis:18.057555,Mastacembelidae_Mastacembelus_erythrotaenia:18.057555):55.63777):7.004676,((Anabantidae_Ctenopoma_acutirostre_kingsleyae:62.782323,(Helostomatidae_Helostoma_temminkii:59.687826,Osphronemidae_Trichopodus_pectoralis:59.687826):3.094497):7.86905,(Channidae_Channa_striata:67.428514,Nandidae_Nandus_nandus:67.428514):3.222859):10.048628):15.7,((Nematistiidae_Nematistius_pectoralis:60.067614,((Coryphaenidae_Coryphaena_hippurus:19.124214,Rachycentridae_Rachycentron_canadum:19.124214):12.775787,(Echeneidae_Remora_osteochir_australis:10.178122,Echeneidae_Echeneis_naucrates:10.178122):21.721879):28.167613):9.732387,((Sphyraenidae_Sphyraena_sphyraena:65.900885,(Menidae_Mene_maculata:60.339531,Polynemidae_Polydactylus_octonemus:60.339531):5.561354):2.899116,((((Leptobramidae_Leptobrama_muelleri:46.58559,Toxotidae_Toxotes_jaculatrix:46.58559):5.096327,(Xiphiidae_Xiphias_gladius:27.833842,Istiophoridae_Istiophorus_platypterus:27.833842):23.848075):5.298938,Carangidae_Trachinotus_falcatus:56.980855):4.861848,((Eolates_gracilis:1,(Centropomidae_Psammoperca_waigiensis:33.068144,(Centropomidae_Lates_calcarifer:13.595548,Centropomidae_Lates_microlepis:13.595548):19.472596):15.93869104):11.83586796,(Heteronectes_chaneti:3.916300993,(Amphistium_paradoxum:5.927514874,(Psettodidae_Psettodes_erumei:57.842703,(Citharidae_Lepidoblepharon_ophthalmolepis:53.600001,((Cynoglossidae_Cynoglossus_interruptus:42.400002,(Soleidae_Solea_solea:24.944331,Soleidae_Soleichthys_heterorhinos:24.944331):17.455671):9.674607,((Scophthalmidae_Scophthalmus_aquosus:23.305906,Scophthalmidae_Lepidorhombus_boscii:23.305906):26.973285,((Paralichthyidae_Paralichthys_dentatus:40.749719,Bothidae_Bothus_lunatus:40.749719):4.387561,(Paralichthyidae_Pseudorhombus_pentophthalmus:21.404631,(((Pleuronectidae_Glyptocephalus_zachirus:5.096079,Pleuronectidae_Microstomus_pacificus:5.096079):2.538752,(Pleuronectidae_Lyopsetta_exilis:6.085211,Pleuronectidae_Hippoglossus_hippoglossus:6.085211):1.54962):2.215451,(Pleuronectidae_Limanda_limanda:8.798272,((Pleuronectidae_Platichthys_stellatus:3.717184,Pleuronectidae_Pleuronectes_platessa:3.717184):3.081088,(Pleuronectidae_Lepidopsetta_bilineata:4.798272,Pleuronectidae_Psettichthys_melanostictus:4.798272):2):2):1.05201):11.554349):23.732649):5.141911):1.795418):1.525392):4.242702):1):1):1):1):6.957298):1):26.6):12.043384,(((Polycentridae_Polycentrus_schomburgkii:94.214904,(Pholidichthyidae_Pholidichthys_leucotaenia:88.700001,Cichlidae_Cichla_temensis:88.700001):5.514903):1,(((Atherinopsidae_Atherinopsis_californiensis:48.715388,Atherinopsidae_Menidia_menidia:48.715388):22.225577,(Isonidae_Iso_sp:49.317292,(Atherinidae_Atherinomorus_stipes:43.78646,(Melanotaeniidae_Melanotaenia_splendida:39.870052,Pseudomugilidae_Pseudomugil_gertrudae:39.870052):3.916408):5.530832):21.623673):6.46052,(((Poeciliidae_Poecilia_latipinna_reticulata:14.292152,(Poeciliidae_Heterandria_formosa:11.366823,(Poeciliidae_Gambusia_affinis:5.736451,Poeciliidae_Belonesox_belizanus:5.736451):5.630372):2.925329):30.13151,(Cyprinodontidae_Cyprinodon_variegatus:40.469994,Fundulidae_Fundulus_parvipinnis:40.469994):3.953668):31.977823,(Adrianichthyidae_Oryzias_latipes:71.878571,((Zenarchopteridae_Dermogenys_collettei:29.883878,((Belonidae_Tylosurus_crocodilus:6.568056,Belonidae_Ablennes_hians:6.568056):17.429484,(Scomberesocidae_Cololabis_saira:2.646712,Scomberesocidae_Scomberesox_saurus:2.646712):21.350828):5.886338):17.178962,(Hemiramphidae_Hyporhamphus_affinis:36.892311,((Hemiramphidae_Hemiramphus_brasiliensis:13.391766,Hemiramphidae_Oxyporhamphus_micropterus:13.391766):10.259915,((Exocoetidae_Cypselurus_callopterus:6.413654,Exocoetidae_Exocoetus_monocirrhus:6.413654):4.605967,Exocoetidae_Cheilopogon_pinnatibarbatus:11.019621):12.63206):13.24063):10.170529):24.815731):4.522914):1):17.813419):1.999954,((Pseudochromidae_Halidesmus_scapularis:84.850287,Pomacentridae_Pomacentrus_brachialis:84.850287):8.187103,(Embiotocidae_Cymatogaster_aggregata:91.529056,(Mugilidae_Mugil_cephalus:89.757655,(Plesiopidae_Plesiops_coeruleolineatus:86.977996,(Opistognathidae_Opistognathus_maxillosus:79.196597,(((Gobiesocidae_Lepadichthys_lineatus:37.560081,Gobiesocidae_Gobiesox_maeandricus:37.560081):35.985217,Tripterygiidae_Enneanectes_altivelis:73.545298):3.122761,((Clinidae_Clinus_cottoides:50.741024,(Dactyloscopidae_Platygillellus_rubrocinctus:45.641062,Chaenopsidae_Chaenopsis_alepidota:45.641062):5.099962):17.520576,(Blenniidae_Stanulus_seychellensis:31.042358,Blenniidae_Blenniella_cyanostigma:31.042358):37.219242):8.406459):2.528538):7.781399):2.779659):1.771401):1.508334):4.177468):11.228527):3.450865,(Gerreidae_Eucinostomus_argenteus:108.000002,(((Labridae_Lachnolaimus_maximus:59.822938,Odacidae_Haletta_semifasciata:59.822938):16.826114,Scaridae_Scarus_globiceps:76.649052):30.35095,(((Uranoscopidae_Uranoscopus_sulphureus:83.996275,(Ammodytidae_Ammodytes_hexapterus:78.584442,Pinguipedidae_Parapercis_punctulata:78.584442):5.411833):11.903726,(((Acropomatidae_Acropoma_japonicum:90.360051,((Percophidae_Acanthaphritis_unoorum:66.16617,Creediidae_Limnichthys_sp:66.16617):18.796708,(Glaucosomatidae_Glaucosoma_buergeri:66.617288,Pempheridae_Pempheris_schomburgkii:66.617288):18.34559):5.397173):2.814411,(((Oplegnathidae_Oplegnathus_punctatus:61.206964,Kuhliidae_Kuhlia_rupestris:61.206964):7.298332,Kyphosidae_Kyphosus_sectatrix:68.505296):16.157201,(Percichthyidae_Percichthys_trucha:69.999181,((Cirrhitidae_Cirrhitichthys_falco:66.467051,Cheilodactylidae_Cheilodactylus_fasciatus:66.467051):2.53213,(Enoplosidae_Enoplosus_armatus:63.04721,Centrarchidae_Acantharchus_pomotis:63.04721):5.951971):1):14.663316):8.511965):1.725539,(Serranidae_Pseudogramma_polyacantha:82.200001,(Percidae_Perca_fluviatilis:78.577195,((Bovichtidae_Bovichtus_diacanthus:63.021097,(Nototheniidae_Notothenia_coriiceps:9.671082,(Harpagiferidae_Harpagifer_antarcticus:8.390321,(Bathydraconidae_Gymnodraco_acuticeps:7.390321,Channichthyidae_Chionodraco_rastrospinosus:7.390321):1):1.280761):53.350015):12.936917,(((Peristediidae_Peristedion_ecuadorense:54.532565,Triglidae_Prionotus_carolinus:54.532565):11.953546,(Synanceiidae_Synanceia_verrucosa:62.510934,Scorpaenidae_Scorpaenodes_guamensis:62.510934):3.975177):6.621302,(Platycephalidae_Platycephalus_indicus:70.795272,(Anoplopomatidae_Anoplopoma_fimbria:46.504594,((Bathymasteridae_Rathbunella_hypoplecta:22.798552,(Stichaeidae_Cebidichthys_violaceus:15.58542,(Zoarcidae_Zoarces_americanus_viviparus:12.493576,(Anarhichadidae_Anarrhichthys_ocellatus:9.933504,(Zaproridae_Zaprora_silenus:8.933504,Cryptacanthodidae_Cryptacanthodes_maculatus:8.933504):1):2.560072):3.091844):7.213132):19.028758,(((Aulorhynchidae_Aulorhynchus_flavidus:25.280385,Hypoptychidae_Hypoptychus_dybowskii:25.280385):1.019618,((Gasterosteidae_Culaea_inconstans:19.844899,(Gasterosteidae_Apeltes_quadracus:17.136644,Gasterosteidae_Spinachia_spinachia:17.136644):2.708255):1.782129,(Gasterosteidae_Pungitius_pungitius:16.827665,Gasterosteidae_Gasterosteus_aculeatus:16.827665):4.799363):4.672975):13.53479,(Hexagrammidae_Hexagrammos_decagrammus:27.818969,((Cyclopteridae_Cyclopterus_lumpus:20.762394,Liparidae_Liparis_pulchellus:20.762394):4.037609,(Agonidae_Hypsagonus_quadricornis:20.371382,(Cottidae_Cottus_carolinae:18.19359,Psychrolutidae_Psychrolutes_phrictus:18.19359):2.177792):4.428621):3.018966):12.015824):1.992517):4.677284):24.290678):2.312141):2.850601):2.619181):3.622806):12.7):1):10.100001,((Drepaneidae_Drepane_punctata:67.658011,Ephippidae_Chaetodipterus_faber:67.658011):37.341991,(((Lobotidae_Lobotes_pacificus_surinamensis:86.438004,Sciaenidae_Menticirrhus_undulatus_littoralis:86.438004):5.761997,((Monodactylidae_Monodactylus_sebae:70.333247,(Avitoluvarus_eocaenicus:19.96759885,(Zanclidae_Zanclus_cornutus:55.859022,Acanthuridae_Acanthurus_triostegus:55.859022):4.740979):9.733246):15.366754,((Pomacanthidae_Pomacanthus_semicirculatus:54.434118,(Leiognathidae_Leiognathus_equulus:32.700001,Chaetodontidae_Chelmon_rostratus:32.700001):21.734117):17.438962,(Emmelichthyidae_Erythrocles_schlegelii:69.871718,(Malacanthidae_Malacanthus_plumieri:68.303733,(Haemulidae_Haemulon_aurolineatum:64.600001,Lutjanidae_Lutjanus_griseus:64.600001):3.703732):1.567985):2.001362):13.826921):6.5):11.800001,((Sillaginidae_Sillago_sihama:85.853361,(Nemipteridae_Pentapodus_caninus:80.270573,(Lethrinidae_Lethrinus_erythropterus:74.370539,Sparidae_Stenotomus_chrysops:74.370539):5.900034):5.582788):17.146641,(Siganidae_Siganus_spinus:102.000002,((Scatophagidae_Scatophagus_argus:75.860371,Priacanthidae_Heteropriacanthus_cruentatus:75.860371):25.139631,((Caproidae_Antigonia_rubescens:10.905674,Caproidae_Antigonia_capros:10.905674):89.094328,(((Lophiidae_Lophiodes_reticulatus:22.462185,Lophiidae_Lophius_gastrophysus:22.462185):42.032119,((Antennariidae_Histrio_histrio:12.333102,Antennariidae_Antennatus_coccineus:12.333102):33.466899,(Chaunacidae_Chaunax_suttkusi:38.499563,(Gigantactinidae_Gigantactis_sp:32.911618,((Ceratiidae_Cryptopsaras_couesii:20.548708,Ceratiidae_Ceratias_holboelli:20.548708):10.416157,((Himantolophidae_Himantolophus_albinares_sagamius:14.456802,Melanocetidae_Melanocetus_murrayi:14.456802):9.124159,(Oneirodidae_Oneirodes_macrosteus:10.164357,Oneirodidae_Dolopichthys_sp:10.164357):13.416604):7.383904):1.946753):5.587945):7.300438):18.694303):34.505698,(((Prohollardia_avita:39.7157285,(Protacanthodes_nimesensis:1,Triacanthidae_Triacanthus_biaculeatus:45.56316175):18.92980925):10.123893,(Aracanidae_Aracana_aurita:54.800001,(Ostraciidae_Ostracion_cubicus:51.000002,Ostraciidae_Rhinesomus_triqueter:51.000002):3.799999):19.816863):3.183137,(((Diodontidae_Diodon_holocanthus:12.198387,Diodontidae_Chilomycterus_schoepfii:12.198387):43.701614,(Tetraodontidae_Canthigaster_bennetti:24.22571,Tetraodontidae_Tetraodon_miurus:24.22571):31.674291):17.334859,((Molidae_Ranzania_laevis:22,Molidae_Mola_mola:22):46.61027,((Monacanthidae_Aluterus_scriptus:27.823604,Monacanthidae_Stephanolepis_hispidus:27.823604):15.976396,(Balistidae_Xanthichthys_ringens:20.7,(Balistidae_Balistes_vetula:19.7,Balistidae_Sufflamen_fraenatum:19.7):1):23.1):24.81027):4.62459):4.565141):21.200001):1):1):1):1):1):1):1):1):1):3.894248):4.592438):5.113313):5.2):6):12.2):1.46362):8.33638):6.58283):11.71717):9.7):9.5):19):3.553787):17.946213):17.8):23.2):9.5):1):1):37.2):1):1):1):1):23.6):1):5.678575219):1):11.73558888):17.43422863):1):1):1):1):4.051608272):1):47);

((Guiyu_oneiros:1,(Onychodus_jandemarrai:33.96742205,((Diplocercides:1,(Latimeriidae_Latimeria_chalumnae:325.4575305,Rhabdoderma:1):40.23288693):46.82520952,((Neoceratodontidae_Neoceratodus_forsteri:279.7982796,Lepidosirenidae_Lepidosiren_paradoxa:279.7982796):128.2017204,((Osteolepis_macrolepidotus:1,Gyroptychius_milleri:5.236749934):1,Eusthenopterus_foordi:22.36778145):10.55870526):4.515627):1):12.98390369):16.50047131,((Cheirolepis_trailli:1,Cheirolepis_schultzei:6.355921627):5.590088144,((Polypteridae_Erpetoichthys_calabaricus:29.200001,Polypteridae_Polypterus_senegalus:29.200001):365.800001,(Osorioichthys_marginis:23.56806398,((Tegeolepis_clarki:18.99771044,Howqualepis_rostridens:1):2.732410545,((Gogosardina_coatesi:3.344978217,(Mimipiscis_bartrami:3.102787122,Mimipiscis_toombsi:1):1):5.301741581,((Moythomasia_lineata:1,Moythomasia_durgaringa:11.28622323):1,(Stegotrachelus_finlayi:1,(Limnomis_delaneyi:1,(Wendyichthys_dicksoni:31.46846679,(Kentuckia_deani:1,((Mesopoma_planti:1.715578914,Mesopoma_carricki:1):4.072640584,((Birgeria_stensioei:1,(Chondrosteus_acipenseroides:1,(Saurichthys_dawaziensis:1,((Protopsephurus_liui:1,Polyodontidae_Polyodon_spathula:128.3426121):10.55738989,(Acipenseridae_Acipenser_fulvescens:38.579335,(Acipenseridae_Scaphirhynchus_platorynchus:19.375515,Acipenseridae_Scaphirhynchus_albus:19.375515):19.20382):100.320667):24.72297685):37.55185872):51.8869022):97.03826123,(Boreosomus:74.36339795,(((Fukangichthys_longidorsalis:6.97819833,(Scanilepis_dubia:45.62643877,Evenkia_eunotoptera:1):1):1,Australosomus:2.95787118):70.87485304,(Perleidus_altolepis:82.76022445,(Luganoia_lepidosteoides:94.45134129,(((Macrosemius_fourneti:88.76993585,((Kyphosichthys_grandei:1,Lepisosteidae_Lepisosteus_osseus:243.9516461):1,Semionotus_elegans:45.56924661):1):21.94835488,(Watsonulus_eugnathoides:1,(Amblysemius:1,(Pachyamia_latimaxillaris:4.36357373,(Tomognathus_mordax:1,Amiidae_Amia_calva:101.4371806):1):49.02548562):101.5279531):14.90938172):54.6,(Pachycormus:105.0879528,(Crossognathus_danubiensis:176.1859559,(((Anaethalion_angustus:1,Anaethalion_knorri:3.184472488):46.37920439,((Megalopidae_Megalops_atlanticus:133.565966,Elopidae_Elops_saurus:133.565966):62.634035,(Albulidae_Albula_vulpes:150.800001,((Notacanthidae_Notacanthus_chemnitzii:50.669287,(Halosauridae_Halosauropsis_macrochir:40.366211,Halosauridae_Aldrovandia_affinis:40.366211):10.303076):50.330714,(((Eurypharyngidae_Eurypharynx_pelecanoides:25.866364,Saccopharyngidae_Saccopharynx_ampullaceus:25.866364):44.322494,(Nemichthyidae_Nemichthys_scolopaceus:58.739485,Anguillidae_Anguilla_rostrata:58.739485):11.449373):9.198895,(Serrivomeridae_Serrivomer_beanii:76.053487,(Congridae_Conger_oceanicus:65.416147,(Muraenesocidae_Muraenesox_cinereus:57.193624,Ophichthidae_Myrichthys_maculosus:57.193624):8.222523):10.63734):3.334266):21.612248):49.8):45.4):1):86.1,((Lycoptera_davidi:98.57943517,(Paralycoptera_wui:108.0880749,(Xixiaichthys_tongxinens:99.55519644,(((Eohiodon_woodruffi:1,Eohiodon_rosei:1.157679153):1,(Hiodon_consteniorum:1,(Hiodontidae_Hiodon_tergisus:9.520291,Hiodontidae_Hiodon_alosoides:9.520291):28.60492336):19.06556911):169.9092175,(Chauliopareion_mahengeense:116.3174548,(Pantodontidae_Pantodon_buchholzi:163.100001,((((Brychaetus_muelleri:1,Phareodus_encaustus:11.83272974):23.98206866,Arapaimidae_Arapaima_gigas:79.3):25.549112,Osteoglossidae_Osteoglossum_bicirrhosum:104.849112):43.475578,(Notopteridae_Xenomystus_nigri:120.300002,Gymnarchidae_Gymnarchus_niloticus:120.300002):28.024688):14.775311):1):63):1):1):1):43.7,((((Diplomystus_brevissimus:45.31360314,Sorbinichthys_africanus:1):92.36947887,(Denticipitidae_Denticeps_clupeoides:188.900001,(Chirocentridae_Chirocentrus_dorab:87.096454,((Engraulidae_Coilia_nasus:47.06527,Engraulidae_Engraulis_mordax_eurystole:47.06527):33.347234,((Pristigasteridae_Pellona_flavipinnis:27.450803,Pristigasteridae_Ilisha_elongata:27.450803):46.430359,(Clupeidae_Alosa_pseudoharengus:54.138864,Clupeidae_Dorosoma_cepedianum:54.138864):19.742298):6.531342):6.68395):101.803547):1):40.3,((Alepocephalidae_Talismania_bifurcata:53.266521,((Alepocephalidae_Bathylaco_nigricans:33.802485,Alepocephalidae_Alepocephalus_tenebrosus:33.802485):10.988666,((Alepocephalidae_Rouleina_attrita:22.275085,Alepocephalidae_Xenodermichthys_copei:22.275085):19.924916,Platytroctidae_Sagamichthys_abei:42.200001):2.59115):8.47537):166.430015,((Gonorynchidae_Gonorynchus_abbreviatus:175.900001,(Mahengichthys_singidaensis:99.70806458,Chanidae_Chanos_chanos:147.100001):28.8):22.7,((((Gyrinocheilidae_Gyrinocheilus_sp:70.111694,(Catostomidae_Hypentelium_nigricans:23.594381,(Catostomidae_Carpiodes_carpio:16.355089,Catostomidae_Ictiobus_bubalus:16.355089):7.239292):46.517313):8.688307,Cobitidae_Cobitis_taenia:78.800001):20.5,(Cyprinidae_Danio_rerio:63.300001,(((Cyprinidae_Zacco_sieboldii_platypus:12.727958,Cyprinidae_Opsariichthys_uncirostris_bidens:12.727958):14.610086,((Cyprinidae_Xenocypris_argentea:12.904177,Cyprinidae_Hypophthalmichthys_molitrix:12.904177):2.917503,(Cyprinidae_Luciobrama_macrocephalus:13.439618,(Cyprinidae_Squaliobarbus_curriculus:11.472717,Cyprinidae_Mylopharyngodon_piceus:11.472717):1.966901):2.382062):11.516364):12.401957,(Cyprinidae_Tanakia_lanceolata_himantegus:35.004261,(Cyprinidae_Notemigonus_crysoleucas:22.933404,(Cyprinidae_Semotilus_atromaculatus:21.325215,((Cyprinidae_Campostoma_oligolepis:12.800846,Cyprinidae_Rhinichthys_cataractae:12.800846):4.408707,(Cyprinidae_Phenacobius_uranops:12.822623,(Cyprinidae_Pimephales_promelas_notatus:9.460336,(Cyprinidae_Luxilus_coccogenis:6.738861,Cyprinidae_Notropis_asperifrons:6.738861):2.721475):3.362287):4.38693):4.115662):1.608189):12.070857):4.73574):23.56):36):73.6,((Gymnotidae_Electrophorus_electricus:63.909447,Gymnotidae_Gymnotus_sp:63.909447):83.924067,(((Distichodontidae_Distichodus_maculatus:103.604365,Citharinidae_Citharinus_congicus:103.604365):11.170138,(Alestidae_Alestes_baremoze:106.666979,((Parodontidae_Parodon_nasus:68.800001,Hemiodontidae_Hemiodus_immaculatus:68.800001):22.415197,((Bryconidae_Brycon_pesu:66.003418,(Gasteropelecidae_Thoracocharax_stellatus:27.746828,Gasteropelecidae_Gasteropelecus_sternicla:27.746828):38.25659):1.227196,Characidae_Astyanax_mexicanus:67.230614):23.984584):15.451781):8.107524):22.236489,((Nematogenyidae_Nematogenys_inermis:110.648758,(Trichomycteridae_Trichomycterus_sp:105.886466,((Loricariidae_Loricaria_simillima:70.313425,Astroblepidae_Astroblepus_sp:70.313425):28.122359,(Callichthyidae_Callichthys_callichthys:69.200001,Callichthyidae_Corydoras_trilineatus:69.200001):29.235783):7.450682):4.762292):5.151243,(Diplomystidae_Diplomystes_nahuelbutaensis:106.203801,((Clariidae_Clarias_batrachus:47.60069,Heteropneustidae_Heteropneustes_fossilis:47.60069):38.718856,(((Sisoridae_Bagarius_yarrelli:55.522058,(Amblycipitidae_Liobagrus_aequilabris:48.027606,Akysidae_Akysis_sp:48.027606):7.494452):21.358911,(Schilbeidae_Pseudeutropius_brachypopterus:74.448774,(Bagridae_Mystus_bocourti:36.077943,Bagridae_Bagrus_ubangensis:36.077943):38.370831):2.432195):8.408138,(((Chacidae_Chaca_sp:72.429482,Cetopsidae_Cetopsis_coecutiens:72.429482):10.714797,(Cranoglanididae_Cranoglanis_bouderius:68.700001,Ictaluridae_Ictalurus_punctatus:68.700001):14.444278):1.039619,(Pangasiidae_Pangasianodon_hypophthalmus:82.158061,((Amphiliidae_Amphilius_jacksonii:69.199757,(Malapteruridae_Malapterurus_beninensis:64.740321,Mochokidae_Synodontis_batesii:64.740321):4.459436):10.993535,((Plotosidae_Plotosus_lineatus:73.039645,Siluridae_Hemisilurus_moolenburghi:73.039645):5.503862,(Auchenipteridae_Ageneiosus_atronasus:51.101654,Doradidae_Anduzedoras_oxyrhynchus:51.101654):27.441853):1.649785):1.964769):2.025837):1.105209):1.030439):19.884255):9.5962):21.210991):10.822522):25.066487):25.7):21.096535):10.503465):20.4,(Orthogonikleithrus_francogalliensis:78.63750318,(((Bathylagidae_Bathylagus_euryops:70.542512,(Microstomatidae_Nansenia_longicauda_ardesiaca:51.394,(Opisthoproctidae_Macropinna_microstoma:33.11268,Argentinidae_Argentina_sialis_silus:33.11268):18.28132):19.148512):89.057489,((Galaxiidae_Neochanna_burrowsius:34.505049,Galaxiidae_Galaxias_maculatus:34.505049):111.121934,(((Umbridae_Novumbra_hubbsi:55.762491,Umbridae_Umbra_limi:55.762491):23.63751,(Esocidae_Esox_lucius:31.366195,Esocidae_Esox_americanus:31.366195):48.033806):25.064928,(Salmonidae_Coregonus_clupeaformis:35.300001,(Salmonidae_Thymallus_brevirostris:32.456522,(Salmonidae_Oncorhynchus_nerka_mykiss:19.929533,(Salmonidae_Salvelinus_alpinus:16.737138,Salmonidae_Salmo_salar:16.737138):3.192395):12.526989):2.843479):69.164928):41.162054):13.973018):55.253787,(((Retropinnidae_Retropinna_semoni:73.700001,(((Osmeridae_Osmerus_mordax:10.332899,Osmeridae_Thaleichthys_pacificus:10.332899):5.872101,(Osmeridae_Mallotus_villosus:14.961758,Osmeridae_Hypomesus_pretiosus:14.961758):1.243242):18.133836,(Plecoglossidae_Plecoglossus_altivelis:28.70318,Salangidae_Neosalangichthys_ishikawae:28.70318):5.635656):39.361165):55.7,((Diplophidae_Diplophos_taenia:77.330737,((Gonostomatidae_Bonapartia_pedaliota:54.297445,Gonostomatidae_Margrethia_obtusirostra:54.297445):20.014374,(Gonostomatidae_Gonostoma_elongatum:69.948163,Gonostomatidae_Cyclothone_microdon:69.948163):4.363656):3.018918):6.469264,(Phosichthyidae_Pollichthys_mauli:77.722827,((Sternoptychidae_Argyropelecus_gigas:25.230481,Sternoptychidae_Maurolicus_weitzmani:25.230481):47.329876,(Stomiidae_Chauliodus_macouni_danae:63.126299,(Stomiidae_Stomias_boa:36.381906,(Stomiidae_Chirostomias_pliopterus:31.578039,((Stomiidae_Melanostomias_margaritifer:21.790927,Stomiidae_Leptostomias_longibarba:21.790927):6.240428,((Stomiidae_Photonectes_margarita:19.053467,Stomiidae_Tactostoma_macropus:19.053467):7.977888,(Stomiidae_Malacosteus_niger:25.031355,(Stomiidae_Eustomias_polyaster:24.031355,(Stomiidae_Aristostomias_scintillans:23.031355,Stomiidae_Bathophilus_flemingi_pawneei:23.031355):1):1):2):1):3.546684):4.803867):26.744393):9.434058):5.16247):6.077174):45.6):81.9,((Ateleopodidae_Ateleopus_japonicus:8.089622,Ateleopodidae_Ijimaia_antillarum:8.089622):184.210379,(((Synodontidae_Trachinocephalus_myops:34.928634,Synodontidae_Synodus_foetens:34.928634):78.971367,((Paraulopidae_Paraulopus_oblongus:101.985288,(Synodontidae_Saurida_gracilis:50.257509,Synodontidae_Harpadon_microchir:50.257509):51.727779):8.326796,(((Aulopidae_Aulopus_filamentosus:47.160387,Bathysauridae_Bathysaurus_ferox:47.160387):10.843902,(Ipnopidae_Ipnops_murrayi:13.82,Ipnopidae_Ipnops_agassizi:13.82):44.184289):23.635948,((Ipnopidae_Bathypterois_atricolor:69.328167,(Giganturidae_Gigantura_indica:13.525058,Giganturidae_Gigantura_chuni:13.525058):55.803109):6.462647,(Scopelarchidae_Benthalbella_macropinna:62.490696,(Chlorophthalmidae_Chlorophthalmus_agassizi:61.13635,(Sudidae_Sudis_atrox:55.026295,((Alepisauridae_Anotopterus_pharao:45.661943,(Alepisauridae_Omosudis_lowii:36.284216,Alepisauridae_Alepisaurus_ferox:36.284216):9.377727):8.238175,(Paralepididae_Stemonosudis_intermedia_macrura:50.122687,(Evermannellidae_Evermannella_balbo:46.686813,Paralepididae_Paralepis_coregonoides:46.686813):3.435874):3.777431):1.126177):6.110055):1.354346):13.300118):5.849423):28.671847):3.587917):68.9,(((Neoscopelidae_Neoscopelus_macrolepidotus:42.408365,Neoscopelidae_Scopelengys_tristis:42.408365):31.191636,((Myctophidae_Lampadena_speculigera:39.81673,Myctophidae_Lampanyctus_macdonaldi:39.81673):11.872,(Myctophidae_Benthosema_glaciale:28.30241,Myctophidae_Myctophum_punctatum:28.30241):23.38632):21.911271):99.5,(((Lamprididae_Lampris_guttatus:65.552505,(Regalecidae_Regalecus_russelii:37.682943,Trachipteridae_Trachipterus_trachypterus:37.682943):27.869562):84.747496,(((Mcconichthys_longipinnis:1,(Aphredoderidae_Aphredoderus_sayanus:41.500001,Amblyopsidae_Chologaster_cornuta:41.500001):25.31324515):1,(Lateopisciculus_turrifumosus:1,(Percopsidae_Percopsis_omiscomaycus:14.617149,Percopsidae_Percopsis_transmontana:14.617149):43.3167698):9.879327343):67.17708585,(Zeidae_Zeus_faber:107.100001,(Stylephoridae_Stylephorus_chordatus:78.800001,(Merlucciidae_Merluccius_productus:45.655086,(((Macrouridae_Gadomus_dispar:9.947595,Macrouridae_Bathygadus_favosus:9.947595):28.939269,(Steindachneriidae_Steindachneria_argentea:35.929071,((Macrouridae_Malacocephalus_laevis:17.761718,Macrouridae_Trachonurus_sulcatus:17.761718):10.126413,(Macrouridae_Coelorinchus_caribbaeus:21.883517,Macrouridae_Coryphaenoides_armatus:21.883517):6.004614):8.04094):2.957793):2.613137,((Moridae_Laemonema_goodebeanorum:25.08833,(Moridae_Halargyreus_johnsonii:16.444614,(Moridae_Lepidion_ensiferus:5.35693,Moridae_Antimora_rostrata:5.35693):11.087684):8.643716):13.1716,((Phycidae_Urophycis_tenuis:5.240351,Phycidae_Urophycis_chuss:5.240351):28.607969,(Lotidae_Gaidropsarus_ensis:29.800286,(Lotidae_Lota_lota:23.935071,(Gadidae_Melanogrammus_aeglefinus:5.86839,Gadidae_Gadus_morhua:5.86839):18.066681):5.865215):4.048034):4.41161):3.240071):4.155085):33.144915):28.3):27.890331):15.309669):11.08283,(Polymixiidae_Polymixia_japonica:154.800001,(((Diretmidae_Diretmus_argenteus:53.99855,(Monocentridae_Monocentris_japonica:33.600001,((Trachichthyidae_Hoplostethus_occidentalis_atlanticus:24.361165,Trachichthyidae_Gephyroberyx_darwinii:24.361165):7.919156,(Anoplogastridae_Anoplogaster_cornuta:31.22776,Trachichthyidae_Paratrachichthys_sajademalensis:31.22776):1.052561):1.31968):20.398549):71.275924,((Berycidae_Beryx_decadactylus:70.612394,(Melamphaidae_Poromitra_crassiceps:39.138751,(Melamphaidae_Scopelogadus_beanii:32.603956,Melamphaidae_Melamphaes_suborbitalis:32.603956):6.534795):31.473643):40.295775,(((Rondeletiidae_Rondeletia_bicolor:18.616456,Rondeletiidae_Rondeletia_loricata:18.616456):36.662305,Barbourisiidae_Barbourisia_rufa:55.278761):34.605685,(Cetomimidae_Cetostoma_regani:55.492218,(Cetomimidae_Cetomimus_craneae:10.537863,Cetomimidae_Cetomimus_compuctus:10.537863):44.954355):34.392228):21.023723):14.366305):21.189147,((Holocentridae_Sargocentron_diadema:25.43456,Holocentridae_Holocentrus_rufus:25.43456):119.565441,((Ophidiidae_Brotula_multibarbata:66.227334,(Ophidiidae_Lepophidium_profundorum:23.036156,(Ophidiidae_Genypterus_blacodes:21.590629,Ophidiidae_Ophidion_holbrookii:21.590629):1.445527):43.191178):66.572667,((Batrachoididae_Porichthys_notatus:39.800001,Batrachoididae_Opsanus_tau:39.800001):87,(((Kurtidae_Kurtus_indicus:80.300001,Apogonidae_Apogon_campbelli:80.300001):22,(Eleotridae_Eleotris_acanthopoma_pisonis:64.430477,(Gobiidae_Gobiosoma_bosc:45.630567,Microdesmidae_Microdesmus_longipinnis:45.630567):18.79991):37.869524):19.3,((((Dactylopteridae_Dactylopterus_volitans:68.121568,(Aulostomidae_Aulostomus_maculatus:3.200001,Aulostomidae_Aulostomus_chinensis:3.200001):64.921567):6.178433,((Fistulariidae_Fistularia_petimba:65.463647,Mullidae_Mullus_auratus:65.463647):6.26906,(Callionymidae_Callionymus_sp_bairdi:69.154942,Syngnathidae_Syngnathus_fuscus:69.154942):2.577765):2.567294):20.27,(Chiasmodontidae_Chiasmodon_sp:43.900001,((Centrolophidae_Icichthys_lockingtoni:34.370774,Pomatomidae_Pomatomus_saltatrix:34.370774):6.934458,(((Ariommatidae_Ariomma_bondi:25.644121,Nomeidae_Psenes_cyanophrys:25.644121):5.370208,Scombridae_Auxis_rochei:31.014329):6.588438,((Stromateidae_Peprilus_paru:35.602767,(Gempylidae_Paradiplospinus_gracilis:31.499638,Bramidae_Brama_brama:31.499638):4.103129):1,(Icosteidae_Icosteus_aenigmaticus:33.972252,Trichiuridae_Trichiurus_lepturus:33.972252):2.630515):1):3.702465):2.594769):50.67):21.916687,((((((Synbranchidae_Monopterus_albus:69.146846,Indostomidae_Indostomus_paradoxus:69.146846):4.548479,(Mastacembelidae_Macrognathus_siamensis:18.057555,Mastacembelidae_Mastacembelus_erythrotaenia:18.057555):55.63777):7.004676,((Anabantidae_Ctenopoma_acutirostre_kingsleyae:62.782323,(Helostomatidae_Helostoma_temminkii:59.687826,Osphronemidae_Trichopodus_pectoralis:59.687826):3.094497):7.86905,(Channidae_Channa_striata:67.428514,Nandidae_Nandus_nandus:67.428514):3.222859):10.048628):15.7,((Nematistiidae_Nematistius_pectoralis:60.067614,((Coryphaenidae_Coryphaena_hippurus:19.124214,Rachycentridae_Rachycentron_canadum:19.124214):12.775787,(Echeneidae_Remora_osteochir_australis:10.178122,Echeneidae_Echeneis_naucrates:10.178122):21.721879):28.167613):9.732387,((Sphyraenidae_Sphyraena_sphyraena:65.900885,(Menidae_Mene_maculata:60.339531,Polynemidae_Polydactylus_octonemus:60.339531):5.561354):2.899116,((((Leptobramidae_Leptobrama_muelleri:46.58559,Toxotidae_Toxotes_jaculatrix:46.58559):5.096327,(Xiphiidae_Xiphias_gladius:27.833842,Istiophoridae_Istiophorus_platypterus:27.833842):23.848075):5.298938,Carangidae_Trachinotus_falcatus:56.980855):4.861848,((Eolates_gracilis:1,(Centropomidae_Psammoperca_waigiensis:33.068144,(Centropomidae_Lates_calcarifer:13.595548,Centropomidae_Lates_microlepis:13.595548):19.472596):22.29870867):5.475850331,(Heteronectes_chaneti:6.667624216,(Amphistium_paradoxum:5.23883098,(Psettodidae_Psettodes_erumei:57.842703,(Citharidae_Lepidoblepharon_ophthalmolepis:53.600001,((Cynoglossidae_Cynoglossus_interruptus:42.400002,(Soleidae_Solea_solea:24.944331,Soleidae_Soleichthys_heterorhinos:24.944331):17.455671):9.674607,((Scophthalmidae_Scophthalmus_aquosus:23.305906,Scophthalmidae_Lepidorhombus_boscii:23.305906):26.973285,((Paralichthyidae_Paralichthys_dentatus:40.749719,Bothidae_Bothus_lunatus:40.749719):4.387561,(Paralichthyidae_Pseudorhombus_pentophthalmus:21.404631,(((Pleuronectidae_Glyptocephalus_zachirus:5.096079,Pleuronectidae_Microstomus_pacificus:5.096079):2.538752,(Pleuronectidae_Lyopsetta_exilis:6.085211,Pleuronectidae_Hippoglossus_hippoglossus:6.085211):1.54962):2.215451,(Pleuronectidae_Limanda_limanda:8.798272,((Pleuronectidae_Platichthys_stellatus:3.717184,Pleuronectidae_Pleuronectes_platessa:3.717184):3.081088,(Pleuronectidae_Lepidopsetta_bilineata:4.798272,Pleuronectidae_Psettichthys_melanostictus:4.798272):2):2):1.05201):11.554349):23.732649):5.141911):1.795418):1.525392):4.242702):1):1):1):1):6.957298):1):26.6):12.043384,(((Polycentridae_Polycentrus_schomburgkii:94.214904,(Pholidichthyidae_Pholidichthys_leucotaenia:88.700001,Cichlidae_Cichla_temensis:88.700001):5.514903):1,(((Atherinopsidae_Atherinopsis_californiensis:48.715388,Atherinopsidae_Menidia_menidia:48.715388):22.225577,(Isonidae_Iso_sp:49.317292,(Atherinidae_Atherinomorus_stipes:43.78646,(Melanotaeniidae_Melanotaenia_splendida:39.870052,Pseudomugilidae_Pseudomugil_gertrudae:39.870052):3.916408):5.530832):21.623673):6.46052,(((Poeciliidae_Poecilia_latipinna_reticulata:14.292152,(Poeciliidae_Heterandria_formosa:11.366823,(Poeciliidae_Gambusia_affinis:5.736451,Poeciliidae_Belonesox_belizanus:5.736451):5.630372):2.925329):30.13151,(Cyprinodontidae_Cyprinodon_variegatus:40.469994,Fundulidae_Fundulus_parvipinnis:40.469994):3.953668):31.977823,(Adrianichthyidae_Oryzias_latipes:71.878571,((Zenarchopteridae_Dermogenys_collettei:29.883878,((Belonidae_Tylosurus_crocodilus:6.568056,Belonidae_Ablennes_hians:6.568056):17.429484,(Scomberesocidae_Cololabis_saira:2.646712,Scomberesocidae_Scomberesox_saurus:2.646712):21.350828):5.886338):17.178962,(Hemiramphidae_Hyporhamphus_affinis:36.892311,((Hemiramphidae_Hemiramphus_brasiliensis:13.391766,Hemiramphidae_Oxyporhamphus_micropterus:13.391766):10.259915,((Exocoetidae_Cypselurus_callopterus:6.413654,Exocoetidae_Exocoetus_monocirrhus:6.413654):4.605967,Exocoetidae_Cheilopogon_pinnatibarbatus:11.019621):12.63206):13.24063):10.170529):24.815731):4.522914):1):17.813419):1.999954,((Pseudochromidae_Halidesmus_scapularis:84.850287,Pomacentridae_Pomacentrus_brachialis:84.850287):8.187103,(Embiotocidae_Cymatogaster_aggregata:91.529056,(Mugilidae_Mugil_cephalus:89.757655,(Plesiopidae_Plesiops_coeruleolineatus:86.977996,(Opistognathidae_Opistognathus_maxillosus:79.196597,(((Gobiesocidae_Lepadichthys_lineatus:37.560081,Gobiesocidae_Gobiesox_maeandricus:37.560081):35.985217,Tripterygiidae_Enneanectes_altivelis:73.545298):3.122761,((Clinidae_Clinus_cottoides:50.741024,(Dactyloscopidae_Platygillellus_rubrocinctus:45.641062,Chaenopsidae_Chaenopsis_alepidota:45.641062):5.099962):17.520576,(Blenniidae_Stanulus_seychellensis:31.042358,Blenniidae_Blenniella_cyanostigma:31.042358):37.219242):8.406459):2.528538):7.781399):2.779659):1.771401):1.508334):4.177468):11.228527):3.450865,(Gerreidae_Eucinostomus_argenteus:108.000002,(((Labridae_Lachnolaimus_maximus:59.822938,Odacidae_Haletta_semifasciata:59.822938):16.826114,Scaridae_Scarus_globiceps:76.649052):30.35095,(((Uranoscopidae_Uranoscopus_sulphureus:83.996275,(Ammodytidae_Ammodytes_hexapterus:78.584442,Pinguipedidae_Parapercis_punctulata:78.584442):5.411833):11.903726,(((Acropomatidae_Acropoma_japonicum:90.360051,((Percophidae_Acanthaphritis_unoorum:66.16617,Creediidae_Limnichthys_sp:66.16617):18.796708,(Glaucosomatidae_Glaucosoma_buergeri:66.617288,Pempheridae_Pempheris_schomburgkii:66.617288):18.34559):5.397173):2.814411,(((Oplegnathidae_Oplegnathus_punctatus:61.206964,Kuhliidae_Kuhlia_rupestris:61.206964):7.298332,Kyphosidae_Kyphosus_sectatrix:68.505296):16.157201,(Percichthyidae_Percichthys_trucha:69.999181,((Cirrhitidae_Cirrhitichthys_falco:66.467051,Cheilodactylidae_Cheilodactylus_fasciatus:66.467051):2.53213,(Enoplosidae_Enoplosus_armatus:63.04721,Centrarchidae_Acantharchus_pomotis:63.04721):5.951971):1):14.663316):8.511965):1.725539,(Serranidae_Pseudogramma_polyacantha:82.200001,(Percidae_Perca_fluviatilis:78.577195,((Bovichtidae_Bovichtus_diacanthus:63.021097,(Nototheniidae_Notothenia_coriiceps:9.671082,(Harpagiferidae_Harpagifer_antarcticus:8.390321,(Bathydraconidae_Gymnodraco_acuticeps:7.390321,Channichthyidae_Chionodraco_rastrospinosus:7.390321):1):1.280761):53.350015):12.936917,(((Peristediidae_Peristedion_ecuadorense:54.532565,Triglidae_Prionotus_carolinus:54.532565):11.953546,(Synanceiidae_Synanceia_verrucosa:62.510934,Scorpaenidae_Scorpaenodes_guamensis:62.510934):3.975177):6.621302,(Platycephalidae_Platycephalus_indicus:70.795272,(Anoplopomatidae_Anoplopoma_fimbria:46.504594,((Bathymasteridae_Rathbunella_hypoplecta:22.798552,(Stichaeidae_Cebidichthys_violaceus:15.58542,(Zoarcidae_Zoarces_americanus_viviparus:12.493576,(Anarhichadidae_Anarrhichthys_ocellatus:9.933504,(Zaproridae_Zaprora_silenus:8.933504,Cryptacanthodidae_Cryptacanthodes_maculatus:8.933504):1):2.560072):3.091844):7.213132):19.028758,(((Aulorhynchidae_Aulorhynchus_flavidus:25.280385,Hypoptychidae_Hypoptychus_dybowskii:25.280385):1.019618,((Gasterosteidae_Culaea_inconstans:19.844899,(Gasterosteidae_Apeltes_quadracus:17.136644,Gasterosteidae_Spinachia_spinachia:17.136644):2.708255):1.782129,(Gasterosteidae_Pungitius_pungitius:16.827665,Gasterosteidae_Gasterosteus_aculeatus:16.827665):4.799363):4.672975):13.53479,(Hexagrammidae_Hexagrammos_decagrammus:27.818969,((Cyclopteridae_Cyclopterus_lumpus:20.762394,Liparidae_Liparis_pulchellus:20.762394):4.037609,(Agonidae_Hypsagonus_quadricornis:20.371382,(Cottidae_Cottus_carolinae:18.19359,Psychrolutidae_Psychrolutes_phrictus:18.19359):2.177792):4.428621):3.018966):12.015824):1.992517):4.677284):24.290678):2.312141):2.850601):2.619181):3.622806):12.7):1):10.100001,((Drepaneidae_Drepane_punctata:67.658011,Ephippidae_Chaetodipterus_faber:67.658011):37.341991,(((Lobotidae_Lobotes_pacificus_surinamensis:86.438004,Sciaenidae_Menticirrhus_undulatus_littoralis:86.438004):5.761997,((Monodactylidae_Monodactylus_sebae:70.333247,(Avitoluvarus_eocaenicus:21.89021466,(Zanclidae_Zanclus_cornutus:55.859022,Acanthuridae_Acanthurus_triostegus:55.859022):4.740979):9.733246):15.366754,((Pomacanthidae_Pomacanthus_semicirculatus:54.434118,(Leiognathidae_Leiognathus_equulus:32.700001,Chaetodontidae_Chelmon_rostratus:32.700001):21.734117):17.438962,(Emmelichthyidae_Erythrocles_schlegelii:69.871718,(Malacanthidae_Malacanthus_plumieri:68.303733,(Haemulidae_Haemulon_aurolineatum:64.600001,Lutjanidae_Lutjanus_griseus:64.600001):3.703732):1.567985):2.001362):13.826921):6.5):11.800001,((Sillaginidae_Sillago_sihama:85.853361,(Nemipteridae_Pentapodus_caninus:80.270573,(Lethrinidae_Lethrinus_erythropterus:74.370539,Sparidae_Stenotomus_chrysops:74.370539):5.900034):5.582788):17.146641,(Siganidae_Siganus_spinus:102.000002,((Scatophagidae_Scatophagus_argus:75.860371,Priacanthidae_Heteropriacanthus_cruentatus:75.860371):25.139631,((Caproidae_Antigonia_rubescens:10.905674,Caproidae_Antigonia_capros:10.905674):89.094328,(((Lophiidae_Lophiodes_reticulatus:22.462185,Lophiidae_Lophius_gastrophysus:22.462185):42.032119,((Antennariidae_Histrio_histrio:12.333102,Antennariidae_Antennatus_coccineus:12.333102):33.466899,(Chaunacidae_Chaunax_suttkusi:38.499563,(Gigantactinidae_Gigantactis_sp:32.911618,((Ceratiidae_Cryptopsaras_couesii:20.548708,Ceratiidae_Ceratias_holboelli:20.548708):10.416157,((Himantolophidae_Himantolophus_albinares_sagamius:14.456802,Melanocetidae_Melanocetus_murrayi:14.456802):9.124159,(Oneirodidae_Oneirodes_macrosteus:10.164357,Oneirodidae_Dolopichthys_sp:10.164357):13.416604):7.383904):1.946753):5.587945):7.300438):18.694303):34.505698,(((Prohollardia_avita:36.41558848,(Protacanthodes_nimesensis:1,Triacanthidae_Triacanthus_biaculeatus:44.40402957):20.08894143):10.123893,(Aracanidae_Aracana_aurita:54.800001,(Ostraciidae_Ostracion_cubicus:51.000002,Ostraciidae_Rhinesomus_triqueter:51.000002):3.799999):19.816863):3.183137,(((Diodontidae_Diodon_holocanthus:12.198387,Diodontidae_Chilomycterus_schoepfii:12.198387):43.701614,(Tetraodontidae_Canthigaster_bennetti:24.22571,Tetraodontidae_Tetraodon_miurus:24.22571):31.674291):17.334859,((Molidae_Ranzania_laevis:22,Molidae_Mola_mola:22):46.61027,((Monacanthidae_Stephanolepis_hispidus:28.823604,Monacanthidae_Aluterus_scriptus:28.823604):14.976396,(Balistidae_Xanthichthys_ringens:20.7,(Balistidae_Balistes_vetula:19.7,Balistidae_Sufflamen_fraenatum:19.7):1):23.1):24.81027):4.62459):4.565141):21.200001):1):1):1):1):1):1):1):1):1):3.894248):4.592438):5.113313):5.2):6):12.2):1.46362):8.33638):6.58283):11.71717):9.7):9.5):19):3.553787):17.946213):17.8):23.2):9.5):1):1):37.2):1):1):1):1):23.6):1):6.898403996):1):8.873499982):17.88395099):2.685635542):1):1):1):3.558510491):1):47);

((Guiyu_oneiros:1,(Onychodus_jandemarrai:35.49331638,((Diplocercides:1,(Latimeriidae_Latimeria_chalumnae:331.8853709,Rhabdoderma:1):35.64036563):44.98989043,((Neoceratodontidae_Neoceratodus_forsteri:279.7982796,Lepidosirenidae_Lepidosiren_paradoxa:279.7982796):128.2017204,((Osteolepis_macrolepidotus:1,Gyroptychius_milleri:15.46539011):1,Eusthenopterus_foordi:26.86412093):1.821833899):4.515627):1):10.88749349):18.59688151,((Cheirolepis_trailli:1,Cheirolepis_schultzei:7.053552667):2.248243522,((Polypteridae_Erpetoichthys_calabaricus:29.200001,Polypteridae_Polypterus_senegalus:29.200001):365.800001,(Osorioichthys_marginis:22.59758948,((Tegeolepis_clarki:16.29573175,Howqualepis_rostridens:1):2.231685364,((Gogosardina_coatesi:10.19294754,(Mimipiscis_bartrami:1,Mimipiscis_toombsi:2.323909927):1):4.993448815,((Moythomasia_lineata:1,Moythomasia_durgaringa:8.640482952):1.289162183,(Stegotrachelus_finlayi:1,(Limnomis_delaneyi:1,(Wendyichthys_dicksoni:22.42863978,(Kentuckia_deani:1.301392136,((Mesopoma_planti:11.89443676,Mesopoma_carricki:1):4.306534282,((Birgeria_stensioei:1,(Chondrosteus_acipenseroides:1,(Saurichthys_dawaziensis:1,((Protopsephurus_liui:1,Polyodontidae_Polyodon_spathula:129.0889678):9.811034176,(Acipenseridae_Acipenser_fulvescens:38.579335,(Acipenseridae_Scaphirhynchus_platorynchus:19.375515,Acipenseridae_Scaphirhynchus_albus:19.375515):19.20382):100.320667):23.49184442):39.16613891):50.96198826):97.58002742,(Boreosomus:74.35535115,(((Fukangichthys_longidorsalis:7.05299367,(Scanilepis_dubia:44.99023734,Evenkia_eunotoptera:1):1):1,Australosomus:2.830402015):70.91118986,(Perleidus_altolepis:82.89124338,(Luganoia_lepidosteoides:92.30529127,(((Macrosemius_fourneti:97.11227703,((Semionotus_elegans:47.39870685,Kyphosichthys_grandei:1):1,Lepisosteidae_Lepisosteus_osseus:249.1491318):1):17.75086919,(Watsonulus_eugnathoides:1,(Amblysemius:1,(Pachyamia_latimaxillaris:1,(Tomognathus_mordax:1,Amiidae_Amia_calva:96.51913151):2.205814578):48.72218398):104.9495093):15.50336161):54.6,(Pachycormus:102.8562616,(Crossognathus_danubiensis:174.9230658,(((Anaethalion_angustus:1.042550338,Anaethalion_knorri:1):48.69339514,((Megalopidae_Megalops_atlanticus:133.565966,Elopidae_Elops_saurus:133.565966):62.634035,(Albulidae_Albula_vulpes:150.800001,((Notacanthidae_Notacanthus_chemnitzii:50.669287,(Halosauridae_Halosauropsis_macrochir:40.366211,Halosauridae_Aldrovandia_affinis:40.366211):10.303076):50.330714,(((Eurypharyngidae_Eurypharynx_pelecanoides:25.866364,Saccopharyngidae_Saccopharynx_ampullaceus:25.866364):44.322494,(Nemichthyidae_Nemichthys_scolopaceus:58.739485,Anguillidae_Anguilla_rostrata:58.739485):11.449373):9.198895,(Serrivomeridae_Serrivomer_beanii:76.053487,(Congridae_Conger_oceanicus:65.416147,(Muraenesocidae_Muraenesox_cinereus:57.193624,Ophichthidae_Myrichthys_maculosus:57.193624):8.222523):10.63734):3.334266):21.612248):49.8):45.4):1):86.1,((Lycoptera_davidi:97.72920958,(Paralycoptera_wui:105.845127,(Xixiaichthys_tongxinens:102.4446595,(((Eohiodon_woodruffi:1.302684134,Eohiodon_rosei:1):1,(Hiodon_consteniorum:1,(Hiodontidae_Hiodon_tergisus:9.520291,Hiodontidae_Hiodon_alosoides:9.520291):29.28592198):18.01418683):170.2796012,(Chauliopareion_mahengeense:119.9404779,(Pantodontidae_Pantodon_buchholzi:163.100001,((((Brychaetus_muelleri:1,Phareodus_encaustus:7.875386657):1,Osteoglossidae_Osteoglossum_bicirrhosum:55.68163934):49.16747266,Arapaimidae_Arapaima_gigas:104.849112):43.475578,(Notopteridae_Xenomystus_nigri:120.300002,Gymnarchidae_Gymnarchus_niloticus:120.300002):28.024688):14.775311):1):63):1):1):1):43.7,((((Diplomystus_brevissimus:44.09079661,Sorbinichthys_africanus:1):92.71908505,(Denticipitidae_Denticeps_clupeoides:188.900001,(Chirocentridae_Chirocentrus_dorab:87.096454,((Engraulidae_Coilia_nasus:47.06527,Engraulidae_Engraulis_mordax_eurystole:47.06527):33.347234,((Pristigasteridae_Pellona_flavipinnis:27.450803,Pristigasteridae_Ilisha_elongata:27.450803):46.430359,(Clupeidae_Alosa_pseudoharengus:54.138864,Clupeidae_Dorosoma_cepedianum:54.138864):19.742298):6.531342):6.68395):101.803547):1):40.3,((Alepocephalidae_Talismania_bifurcata:53.266521,((Alepocephalidae_Bathylaco_nigricans:33.802485,Alepocephalidae_Alepocephalus_tenebrosus:33.802485):10.988666,((Alepocephalidae_Rouleina_attrita:22.275085,Alepocephalidae_Xenodermichthys_copei:22.275085):19.924916,Platytroctidae_Sagamichthys_abei:42.200001):2.59115):8.47537):166.430015,((Gonorynchidae_Gonorynchus_abbreviatus:175.900001,(Mahengichthys_singidaensis:101.6477891,Chanidae_Chanos_chanos:147.100001):28.8):22.7,((((Gyrinocheilidae_Gyrinocheilus_sp:70.111694,(Catostomidae_Hypentelium_nigricans:23.594381,(Catostomidae_Carpiodes_carpio:16.355089,Catostomidae_Ictiobus_bubalus:16.355089):7.239292):46.517313):8.688307,Cobitidae_Cobitis_taenia:78.800001):20.5,(Cyprinidae_Danio_rerio:63.300001,(((Cyprinidae_Zacco_sieboldii_platypus:12.727958,Cyprinidae_Opsariichthys_uncirostris_bidens:12.727958):14.610086,((Cyprinidae_Xenocypris_argentea:12.904177,Cyprinidae_Hypophthalmichthys_molitrix:12.904177):2.917503,(Cyprinidae_Luciobrama_macrocephalus:13.439618,(Cyprinidae_Squaliobarbus_curriculus:11.472717,Cyprinidae_Mylopharyngodon_piceus:11.472717):1.966901):2.382062):11.516364):12.401957,(Cyprinidae_Tanakia_lanceolata_himantegus:35.004261,(Cyprinidae_Notemigonus_crysoleucas:22.933404,(Cyprinidae_Semotilus_atromaculatus:21.325215,((Cyprinidae_Campostoma_oligolepis:12.800846,Cyprinidae_Rhinichthys_cataractae:12.800846):4.408707,(Cyprinidae_Phenacobius_uranops:12.822623,(Cyprinidae_Pimephales_promelas_notatus:9.460336,(Cyprinidae_Luxilus_coccogenis:6.738861,Cyprinidae_Notropis_asperifrons:6.738861):2.721475):3.362287):4.38693):4.115662):1.608189):12.070857):4.73574):23.56):36):73.6,((Gymnotidae_Electrophorus_electricus:63.909447,Gymnotidae_Gymnotus_sp:63.909447):83.924067,(((Distichodontidae_Distichodus_maculatus:103.604365,Citharinidae_Citharinus_congicus:103.604365):11.170138,(Alestidae_Alestes_baremoze:106.666979,((Parodontidae_Parodon_nasus:68.800001,Hemiodontidae_Hemiodus_immaculatus:68.800001):22.415197,((Bryconidae_Brycon_pesu:66.003418,(Gasteropelecidae_Thoracocharax_stellatus:27.746828,Gasteropelecidae_Gasteropelecus_sternicla:27.746828):38.25659):1.227196,Characidae_Astyanax_mexicanus:67.230614):23.984584):15.451781):8.107524):22.236489,((Nematogenyidae_Nematogenys_inermis:110.648758,(Trichomycteridae_Trichomycterus_sp:105.886466,((Loricariidae_Loricaria_simillima:70.313425,Astroblepidae_Astroblepus_sp:70.313425):28.122359,(Callichthyidae_Callichthys_callichthys:69.200001,Callichthyidae_Corydoras_trilineatus:69.200001):29.235783):7.450682):4.762292):5.151243,(Diplomystidae_Diplomystes_nahuelbutaensis:106.203801,((Clariidae_Clarias_batrachus:47.60069,Heteropneustidae_Heteropneustes_fossilis:47.60069):38.718856,(((Sisoridae_Bagarius_yarrelli:55.522058,(Amblycipitidae_Liobagrus_aequilabris:48.027606,Akysidae_Akysis_sp:48.027606):7.494452):21.358911,(Schilbeidae_Pseudeutropius_brachypopterus:74.448774,(Bagridae_Mystus_bocourti:36.077943,Bagridae_Bagrus_ubangensis:36.077943):38.370831):2.432195):8.408138,(((Chacidae_Chaca_sp:72.429482,Cetopsidae_Cetopsis_coecutiens:72.429482):10.714797,(Cranoglanididae_Cranoglanis_bouderius:68.700001,Ictaluridae_Ictalurus_punctatus:68.700001):14.444278):1.039619,(Pangasiidae_Pangasianodon_hypophthalmus:82.158061,((Amphiliidae_Amphilius_jacksonii:69.199757,(Malapteruridae_Malapterurus_beninensis:64.740321,Mochokidae_Synodontis_batesii:64.740321):4.459436):10.993535,((Plotosidae_Plotosus_lineatus:73.039645,Siluridae_Hemisilurus_moolenburghi:73.039645):5.503862,(Auchenipteridae_Ageneiosus_atronasus:51.101654,Doradidae_Anduzedoras_oxyrhynchus:51.101654):27.441853):1.649785):1.964769):2.025837):1.105209):1.030439):19.884255):9.5962):21.210991):10.822522):25.066487):25.7):21.096535):10.503465):20.4,(Orthogonikleithrus_francogalliensis:77.88454806,(((Bathylagidae_Bathylagus_euryops:70.542512,(Microstomatidae_Nansenia_longicauda_ardesiaca:51.394,(Opisthoproctidae_Macropinna_microstoma:33.11268,Argentinidae_Argentina_sialis_silus:33.11268):18.28132):19.148512):89.057489,((Galaxiidae_Neochanna_burrowsius:34.505049,Galaxiidae_Galaxias_maculatus:34.505049):111.121934,(((Umbridae_Novumbra_hubbsi:55.762491,Umbridae_Umbra_limi:55.762491):23.63751,(Esocidae_Esox_lucius:31.366195,Esocidae_Esox_americanus:31.366195):48.033806):25.064928,(Salmonidae_Coregonus_clupeaformis:35.300001,(Salmonidae_Thymallus_brevirostris:32.456522,(Salmonidae_Oncorhynchus_nerka_mykiss:19.929533,(Salmonidae_Salvelinus_alpinus:16.737138,Salmonidae_Salmo_salar:16.737138):3.192395):12.526989):2.843479):69.164928):41.162054):13.973018):55.253787,(((Retropinnidae_Retropinna_semoni:73.700001,(((Osmeridae_Osmerus_mordax:10.332899,Osmeridae_Thaleichthys_pacificus:10.332899):5.872101,(Osmeridae_Mallotus_villosus:14.961758,Osmeridae_Hypomesus_pretiosus:14.961758):1.243242):18.133836,(Plecoglossidae_Plecoglossus_altivelis:28.70318,Salangidae_Neosalangichthys_ishikawae:28.70318):5.635656):39.361165):55.7,((Diplophidae_Diplophos_taenia:77.330737,((Gonostomatidae_Bonapartia_pedaliota:54.297445,Gonostomatidae_Margrethia_obtusirostra:54.297445):20.014374,(Gonostomatidae_Gonostoma_elongatum:69.948163,Gonostomatidae_Cyclothone_microdon:69.948163):4.363656):3.018918):6.469264,(Phosichthyidae_Pollichthys_mauli:77.722827,((Sternoptychidae_Argyropelecus_gigas:25.230481,Sternoptychidae_Maurolicus_weitzmani:25.230481):47.329876,(Stomiidae_Chauliodus_macouni_danae:63.126299,(Stomiidae_Stomias_boa:36.381906,(Stomiidae_Chirostomias_pliopterus:31.578039,((Stomiidae_Melanostomias_margaritifer:21.790927,Stomiidae_Leptostomias_longibarba:21.790927):6.240428,((Stomiidae_Photonectes_margarita:19.053467,Stomiidae_Tactostoma_macropus:19.053467):7.977888,(Stomiidae_Malacosteus_niger:25.031355,(Stomiidae_Eustomias_polyaster:24.031355,(Stomiidae_Aristostomias_scintillans:23.031355,Stomiidae_Bathophilus_flemingi_pawneei:23.031355):1):1):2):1):3.546684):4.803867):26.744393):9.434058):5.16247):6.077174):45.6):81.9,((Ateleopodidae_Ateleopus_japonicus:8.089622,Ateleopodidae_Ijimaia_antillarum:8.089622):184.210379,(((Synodontidae_Trachinocephalus_myops:34.928634,Synodontidae_Synodus_foetens:34.928634):78.971367,((Paraulopidae_Paraulopus_oblongus:101.985288,(Synodontidae_Saurida_gracilis:50.257509,Synodontidae_Harpadon_microchir:50.257509):51.727779):8.326796,(((Aulopidae_Aulopus_filamentosus:47.160387,Bathysauridae_Bathysaurus_ferox:47.160387):10.843902,(Ipnopidae_Ipnops_murrayi:13.82,Ipnopidae_Ipnops_agassizi:13.82):44.184289):23.635948,((Ipnopidae_Bathypterois_atricolor:69.328167,(Giganturidae_Gigantura_indica:13.525058,Giganturidae_Gigantura_chuni:13.525058):55.803109):6.462647,(Scopelarchidae_Benthalbella_macropinna:62.490696,(Chlorophthalmidae_Chlorophthalmus_agassizi:61.13635,(Sudidae_Sudis_atrox:55.026295,((Alepisauridae_Anotopterus_pharao:45.661943,(Alepisauridae_Omosudis_lowii:36.284216,Alepisauridae_Alepisaurus_ferox:36.284216):9.377727):8.238175,(Paralepididae_Stemonosudis_intermedia_macrura:50.122687,(Evermannellidae_Evermannella_balbo:46.686813,Paralepididae_Paralepis_coregonoides:46.686813):3.435874):3.777431):1.126177):6.110055):1.354346):13.300118):5.849423):28.671847):3.587917):68.9,(((Neoscopelidae_Neoscopelus_macrolepidotus:42.408365,Neoscopelidae_Scopelengys_tristis:42.408365):31.191636,((Myctophidae_Lampadena_speculigera:39.81673,Myctophidae_Lampanyctus_macdonaldi:39.81673):11.872,(Myctophidae_Benthosema_glaciale:28.30241,Myctophidae_Myctophum_punctatum:28.30241):23.38632):21.911271):99.5,(((Lamprididae_Lampris_guttatus:65.552505,(Regalecidae_Regalecus_russelii:37.682943,Trachipteridae_Trachipterus_trachypterus:37.682943):27.869562):84.747496,(((Mcconichthys_longipinnis:1,(Aphredoderidae_Aphredoderus_sayanus:41.500001,Amblyopsidae_Chologaster_cornuta:41.500001):25.42914746):1,(Lateopisciculus_turrifumosus:1,(Percopsidae_Percopsis_omiscomaycus:14.617149,Percopsidae_Percopsis_transmontana:14.617149):45.13175787):8.180241596):67.06118354,(Zeidae_Zeus_faber:107.100001,(Stylephoridae_Stylephorus_chordatus:78.800001,(Merlucciidae_Merluccius_productus:45.655086,(((Macrouridae_Gadomus_dispar:9.947595,Macrouridae_Bathygadus_favosus:9.947595):28.939269,(Steindachneriidae_Steindachneria_argentea:35.929071,((Macrouridae_Malacocephalus_laevis:17.761718,Macrouridae_Trachonurus_sulcatus:17.761718):10.126413,(Macrouridae_Coelorinchus_caribbaeus:21.883517,Macrouridae_Coryphaenoides_armatus:21.883517):6.004614):8.04094):2.957793):2.613137,((Moridae_Laemonema_goodebeanorum:25.08833,(Moridae_Halargyreus_johnsonii:16.444614,(Moridae_Lepidion_ensiferus:5.35693,Moridae_Antimora_rostrata:5.35693):11.087684):8.643716):13.1716,((Phycidae_Urophycis_tenuis:5.240351,Phycidae_Urophycis_chuss:5.240351):28.607969,(Lotidae_Gaidropsarus_ensis:29.800286,(Lotidae_Lota_lota:23.935071,(Gadidae_Melanogrammus_aeglefinus:5.86839,Gadidae_Gadus_morhua:5.86839):18.066681):5.865215):4.048034):4.41161):3.240071):4.155085):33.144915):28.3):27.890331):15.309669):11.08283,(Polymixiidae_Polymixia_japonica:154.800001,(((Diretmidae_Diretmus_argenteus:53.99855,(Monocentridae_Monocentris_japonica:33.600001,((Trachichthyidae_Hoplostethus_occidentalis_atlanticus:24.361165,Trachichthyidae_Gephyroberyx_darwinii:24.361165):7.919156,(Anoplogastridae_Anoplogaster_cornuta:31.22776,Trachichthyidae_Paratrachichthys_sajademalensis:31.22776):1.052561):1.31968):20.398549):71.275924,((Berycidae_Beryx_decadactylus:70.612394,(Melamphaidae_Poromitra_crassiceps:39.138751,(Melamphaidae_Scopelogadus_beanii:32.603956,Melamphaidae_Melamphaes_suborbitalis:32.603956):6.534795):31.473643):40.295775,(((Rondeletiidae_Rondeletia_bicolor:18.616456,Rondeletiidae_Rondeletia_loricata:18.616456):36.662305,Barbourisiidae_Barbourisia_rufa:55.278761):34.605685,(Cetomimidae_Cetostoma_regani:55.492218,(Cetomimidae_Cetomimus_craneae:10.537863,Cetomimidae_Cetomimus_compuctus:10.537863):44.954355):34.392228):21.023723):14.366305):21.189147,((Holocentridae_Sargocentron_diadema:25.43456,Holocentridae_Holocentrus_rufus:25.43456):119.565441,((Ophidiidae_Brotula_multibarbata:66.227334,(Ophidiidae_Lepophidium_profundorum:23.036156,(Ophidiidae_Genypterus_blacodes:21.590629,Ophidiidae_Ophidion_holbrookii:21.590629):1.445527):43.191178):66.572667,((Batrachoididae_Porichthys_notatus:39.800001,Batrachoididae_Opsanus_tau:39.800001):87,(((Kurtidae_Kurtus_indicus:80.300001,Apogonidae_Apogon_campbelli:80.300001):22,(Eleotridae_Eleotris_acanthopoma_pisonis:64.430477,(Gobiidae_Gobiosoma_bosc:45.630567,Microdesmidae_Microdesmus_longipinnis:45.630567):18.79991):37.869524):19.3,((((Dactylopteridae_Dactylopterus_volitans:68.121568,(Aulostomidae_Aulostomus_maculatus:3.200001,Aulostomidae_Aulostomus_chinensis:3.200001):64.921567):6.178433,((Fistulariidae_Fistularia_petimba:65.463647,Mullidae_Mullus_auratus:65.463647):6.26906,(Callionymidae_Callionymus_sp_bairdi:69.154942,Syngnathidae_Syngnathus_fuscus:69.154942):2.577765):2.567294):20.27,(Chiasmodontidae_Chiasmodon_sp:43.900001,((Centrolophidae_Icichthys_lockingtoni:34.370774,Pomatomidae_Pomatomus_saltatrix:34.370774):6.934458,(((Ariommatidae_Ariomma_bondi:25.644121,Nomeidae_Psenes_cyanophrys:25.644121):5.370208,Scombridae_Auxis_rochei:31.014329):6.588438,((Stromateidae_Peprilus_paru:35.602767,(Gempylidae_Paradiplospinus_gracilis:31.499638,Bramidae_Brama_brama:31.499638):4.103129):1,(Icosteidae_Icosteus_aenigmaticus:33.972252,Trichiuridae_Trichiurus_lepturus:33.972252):2.630515):1):3.702465):2.594769):50.67):21.916687,((((((Synbranchidae_Monopterus_albus:69.146846,Indostomidae_Indostomus_paradoxus:69.146846):4.548479,(Mastacembelidae_Macrognathus_siamensis:18.057555,Mastacembelidae_Mastacembelus_erythrotaenia:18.057555):55.63777):7.004676,((Anabantidae_Ctenopoma_acutirostre_kingsleyae:62.782323,(Helostomatidae_Helostoma_temminkii:59.687826,Osphronemidae_Trichopodus_pectoralis:59.687826):3.094497):7.86905,(Channidae_Channa_striata:67.428514,Nandidae_Nandus_nandus:67.428514):3.222859):10.048628):15.7,((Nematistiidae_Nematistius_pectoralis:60.067614,((Coryphaenidae_Coryphaena_hippurus:19.124214,Rachycentridae_Rachycentron_canadum:19.124214):12.775787,(Echeneidae_Remora_osteochir_australis:10.178122,Echeneidae_Echeneis_naucrates:10.178122):21.721879):28.167613):9.732387,((Sphyraenidae_Sphyraena_sphyraena:65.900885,(Menidae_Mene_maculata:60.339531,Polynemidae_Polydactylus_octonemus:60.339531):5.561354):2.899116,((((Leptobramidae_Leptobrama_muelleri:46.58559,Toxotidae_Toxotes_jaculatrix:46.58559):5.096327,(Xiphiidae_Xiphias_gladius:27.833842,Istiophoridae_Istiophorus_platypterus:27.833842):23.848075):5.298938,Carangidae_Trachinotus_falcatus:56.980855):4.861848,((Eolates_gracilis:1,(Centropomidae_Psammoperca_waigiensis:33.068144,(Centropomidae_Lates_calcarifer:13.595548,Centropomidae_Lates_microlepis:13.595548):19.472596):20.31913058):7.455428422,(Heteronectes_chaneti:3.867916042,(Amphistium_paradoxum:4.437120733,(Psettodidae_Psettodes_erumei:57.842703,(Citharidae_Lepidoblepharon_ophthalmolepis:53.600001,((Cynoglossidae_Cynoglossus_interruptus:42.400002,(Soleidae_Solea_solea:24.944331,Soleidae_Soleichthys_heterorhinos:24.944331):17.455671):9.674607,((Scophthalmidae_Scophthalmus_aquosus:23.305906,Scophthalmidae_Lepidorhombus_boscii:23.305906):26.973285,((Paralichthyidae_Paralichthys_dentatus:40.749719,Bothidae_Bothus_lunatus:40.749719):4.387561,(Paralichthyidae_Pseudorhombus_pentophthalmus:21.404631,(((Pleuronectidae_Glyptocephalus_zachirus:5.096079,Pleuronectidae_Microstomus_pacificus:5.096079):2.538752,(Pleuronectidae_Lyopsetta_exilis:6.085211,Pleuronectidae_Hippoglossus_hippoglossus:6.085211):1.54962):2.215451,(Pleuronectidae_Limanda_limanda:8.798272,((Pleuronectidae_Platichthys_stellatus:3.717184,Pleuronectidae_Pleuronectes_platessa:3.717184):3.081088,(Pleuronectidae_Lepidopsetta_bilineata:4.798272,Pleuronectidae_Psettichthys_melanostictus:4.798272):2):2):1.05201):11.554349):23.732649):5.141911):1.795418):1.525392):4.242702):1):1):1):1):6.957298):1):26.6):12.043384,(((Polycentridae_Polycentrus_schomburgkii:94.214904,(Pholidichthyidae_Pholidichthys_leucotaenia:88.700001,Cichlidae_Cichla_temensis:88.700001):5.514903):1,(((Atherinopsidae_Atherinopsis_californiensis:48.715388,Atherinopsidae_Menidia_menidia:48.715388):22.225577,(Isonidae_Iso_sp:49.317292,(Atherinidae_Atherinomorus_stipes:43.78646,(Melanotaeniidae_Melanotaenia_splendida:39.870052,Pseudomugilidae_Pseudomugil_gertrudae:39.870052):3.916408):5.530832):21.623673):6.46052,(((Poeciliidae_Poecilia_latipinna_reticulata:14.292152,(Poeciliidae_Heterandria_formosa:11.366823,(Poeciliidae_Gambusia_affinis:5.736451,Poeciliidae_Belonesox_belizanus:5.736451):5.630372):2.925329):30.13151,(Cyprinodontidae_Cyprinodon_variegatus:40.469994,Fundulidae_Fundulus_parvipinnis:40.469994):3.953668):31.977823,(Adrianichthyidae_Oryzias_latipes:71.878571,((Zenarchopteridae_Dermogenys_collettei:29.883878,((Belonidae_Tylosurus_crocodilus:6.568056,Belonidae_Ablennes_hians:6.568056):17.429484,(Scomberesocidae_Cololabis_saira:2.646712,Scomberesocidae_Scomberesox_saurus:2.646712):21.350828):5.886338):17.178962,(Hemiramphidae_Hyporhamphus_affinis:36.892311,((Hemiramphidae_Hemiramphus_brasiliensis:13.391766,Hemiramphidae_Oxyporhamphus_micropterus:13.391766):10.259915,((Exocoetidae_Cypselurus_callopterus:6.413654,Exocoetidae_Exocoetus_monocirrhus:6.413654):4.605967,Exocoetidae_Cheilopogon_pinnatibarbatus:11.019621):12.63206):13.24063):10.170529):24.815731):4.522914):1):17.813419):1.999954,((Pseudochromidae_Halidesmus_scapularis:84.850287,Pomacentridae_Pomacentrus_brachialis:84.850287):8.187103,(Embiotocidae_Cymatogaster_aggregata:91.529056,(Mugilidae_Mugil_cephalus:89.757655,(Plesiopidae_Plesiops_coeruleolineatus:86.977996,(Opistognathidae_Opistognathus_maxillosus:79.196597,(((Gobiesocidae_Lepadichthys_lineatus:37.560081,Gobiesocidae_Gobiesox_maeandricus:37.560081):35.985217,Tripterygiidae_Enneanectes_altivelis:73.545298):3.122761,((Clinidae_Clinus_cottoides:50.741024,(Dactyloscopidae_Platygillellus_rubrocinctus:45.641062,Chaenopsidae_Chaenopsis_alepidota:45.641062):5.099962):17.520576,(Blenniidae_Stanulus_seychellensis:31.042358,Blenniidae_Blenniella_cyanostigma:31.042358):37.219242):8.406459):2.528538):7.781399):2.779659):1.771401):1.508334):4.177468):11.228527):3.450865,(Gerreidae_Eucinostomus_argenteus:108.000002,(((Labridae_Lachnolaimus_maximus:59.822938,Odacidae_Haletta_semifasciata:59.822938):16.826114,Scaridae_Scarus_globiceps:76.649052):30.35095,(((Uranoscopidae_Uranoscopus_sulphureus:83.996275,(Ammodytidae_Ammodytes_hexapterus:78.584442,Pinguipedidae_Parapercis_punctulata:78.584442):5.411833):11.903726,(((Acropomatidae_Acropoma_japonicum:90.360051,((Percophidae_Acanthaphritis_unoorum:66.16617,Creediidae_Limnichthys_sp:66.16617):18.796708,(Glaucosomatidae_Glaucosoma_buergeri:66.617288,Pempheridae_Pempheris_schomburgkii:66.617288):18.34559):5.397173):2.814411,(((Oplegnathidae_Oplegnathus_punctatus:61.206964,Kuhliidae_Kuhlia_rupestris:61.206964):7.298332,Kyphosidae_Kyphosus_sectatrix:68.505296):16.157201,(Percichthyidae_Percichthys_trucha:69.999181,((Cirrhitidae_Cirrhitichthys_falco:66.467051,Cheilodactylidae_Cheilodactylus_fasciatus:66.467051):2.53213,(Enoplosidae_Enoplosus_armatus:63.04721,Centrarchidae_Acantharchus_pomotis:63.04721):5.951971):1):14.663316):8.511965):1.725539,(Serranidae_Pseudogramma_polyacantha:82.200001,(Percidae_Perca_fluviatilis:78.577195,((Bovichtidae_Bovichtus_diacanthus:63.021097,(Nototheniidae_Notothenia_coriiceps:9.671082,(Harpagiferidae_Harpagifer_antarcticus:8.390321,(Bathydraconidae_Gymnodraco_acuticeps:7.390321,Channichthyidae_Chionodraco_rastrospinosus:7.390321):1):1.280761):53.350015):12.936917,(((Peristediidae_Peristedion_ecuadorense:54.532565,Triglidae_Prionotus_carolinus:54.532565):11.953546,(Synanceiidae_Synanceia_verrucosa:62.510934,Scorpaenidae_Scorpaenodes_guamensis:62.510934):3.975177):6.621302,(Platycephalidae_Platycephalus_indicus:70.795272,(Anoplopomatidae_Anoplopoma_fimbria:46.504594,((Bathymasteridae_Rathbunella_hypoplecta:22.798552,(Stichaeidae_Cebidichthys_violaceus:15.58542,(Zoarcidae_Zoarces_americanus_viviparus:12.493576,(Anarhichadidae_Anarrhichthys_ocellatus:9.933504,(Zaproridae_Zaprora_silenus:8.933504,Cryptacanthodidae_Cryptacanthodes_maculatus:8.933504):1):2.560072):3.091844):7.213132):19.028758,(((Aulorhynchidae_Aulorhynchus_flavidus:25.280385,Hypoptychidae_Hypoptychus_dybowskii:25.280385):1.019618,((Gasterosteidae_Culaea_inconstans:19.844899,(Gasterosteidae_Apeltes_quadracus:17.136644,Gasterosteidae_Spinachia_spinachia:17.136644):2.708255):1.782129,(Gasterosteidae_Pungitius_pungitius:16.827665,Gasterosteidae_Gasterosteus_aculeatus:16.827665):4.799363):4.672975):13.53479,(Hexagrammidae_Hexagrammos_decagrammus:27.818969,((Cyclopteridae_Cyclopterus_lumpus:20.762394,Liparidae_Liparis_pulchellus:20.762394):4.037609,(Agonidae_Hypsagonus_quadricornis:20.371382,(Cottidae_Cottus_carolinae:18.19359,Psychrolutidae_Psychrolutes_phrictus:18.19359):2.177792):4.428621):3.018966):12.015824):1.992517):4.677284):24.290678):2.312141):2.850601):2.619181):3.622806):12.7):1):10.100001,((Drepaneidae_Drepane_punctata:67.658011,Ephippidae_Chaetodipterus_faber:67.658011):37.341991,(((Lobotidae_Lobotes_pacificus_surinamensis:86.438004,Sciaenidae_Menticirrhus_undulatus_littoralis:86.438004):5.761997,((Monodactylidae_Monodactylus_sebae:70.333247,(Avitoluvarus_eocaenicus:21.59377218,(Zanclidae_Zanclus_cornutus:55.859022,Acanthuridae_Acanthurus_triostegus:55.859022):4.740979):9.733246):15.366754,((Pomacanthidae_Pomacanthus_semicirculatus:54.434118,(Leiognathidae_Leiognathus_equulus:32.700001,Chaetodontidae_Chelmon_rostratus:32.700001):21.734117):17.438962,(Emmelichthyidae_Erythrocles_schlegelii:69.871718,(Malacanthidae_Malacanthus_plumieri:68.303733,(Haemulidae_Haemulon_aurolineatum:64.600001,Lutjanidae_Lutjanus_griseus:64.600001):3.703732):1.567985):2.001362):13.826921):6.5):11.800001,((Sillaginidae_Sillago_sihama:85.853361,(Nemipteridae_Pentapodus_caninus:80.270573,(Lethrinidae_Lethrinus_erythropterus:74.370539,Sparidae_Stenotomus_chrysops:74.370539):5.900034):5.582788):17.146641,(Siganidae_Siganus_spinus:102.000002,((Scatophagidae_Scatophagus_argus:75.860371,Priacanthidae_Heteropriacanthus_cruentatus:75.860371):25.139631,((Caproidae_Antigonia_rubescens:10.905674,Caproidae_Antigonia_capros:10.905674):89.094328,(((Lophiidae_Lophiodes_reticulatus:22.462185,Lophiidae_Lophius_gastrophysus:22.462185):42.032119,((Antennariidae_Histrio_histrio:12.333102,Antennariidae_Antennatus_coccineus:12.333102):33.466899,(Chaunacidae_Chaunax_suttkusi:38.499563,(Gigantactinidae_Gigantactis_sp:32.911618,((Ceratiidae_Cryptopsaras_couesii:20.548708,Ceratiidae_Ceratias_holboelli:20.548708):10.416157,((Himantolophidae_Himantolophus_albinares_sagamius:14.456802,Melanocetidae_Melanocetus_murrayi:14.456802):9.124159,(Oneirodidae_Oneirodes_macrosteus:10.164357,Oneirodidae_Dolopichthys_sp:10.164357):13.416604):7.383904):1.946753):5.587945):7.300438):18.694303):34.505698,(((Prohollardia_avita:38.50745026,(Protacanthodes_nimesensis:1,Triacanthidae_Triacanthus_biaculeatus:48.55616561):15.93680539):10.123893,(Aracanidae_Aracana_aurita:54.800001,(Ostraciidae_Ostracion_cubicus:51.000002,Ostraciidae_Rhinesomus_triqueter:51.000002):3.799999):19.816863):3.183137,(((Diodontidae_Diodon_holocanthus:12.198387,Diodontidae_Chilomycterus_schoepfii:12.198387):43.701614,(Tetraodontidae_Canthigaster_bennetti:24.22571,Tetraodontidae_Tetraodon_miurus:24.22571):31.674291):17.334859,((Molidae_Ranzania_laevis:22,Molidae_Mola_mola:22):46.61027,((Monacanthidae_Aluterus_scriptus:27.823604,Monacanthidae_Stephanolepis_hispidus:27.823604):15.976396,(Balistidae_Xanthichthys_ringens:20.7,(Balistidae_Balistes_vetula:19.7,Balistidae_Sufflamen_fraenatum:19.7):1):23.1):24.81027):4.62459):4.565141):21.200001):1):1):1):1):1):1):1):1):1):3.894248):4.592438):5.113313):5.2):6):12.2):1.46362):8.33638):6.58283):11.71717):9.7):9.5):19):3.553787):17.946213):17.8):23.2):9.5):1):1):37.2):1):1):1):1):23.6):1):1):1):18.23705912):16.07387484):1):1):1):1):3.589067038):1):47);

((Guiyu_oneiros:1,(Onychodus_jandemarrai:34.27126642,((Diplocercides:1,(Latimeriidae_Latimeria_chalumnae:330.6932996,Rhabdoderma:1):41.43361181):40.38871562,((Neoceratodontidae_Neoceratodus_forsteri:279.7982796,Lepidosirenidae_Lepidosiren_paradoxa:279.7982796):130.4132223,((Osteolepis_macrolepidotus:1,Gyroptychius_milleri:15.41547561):1,Eusthenopterus_foordi:29.12554371):1):2.304125103):1):13.1206851):16.3636899,((Cheirolepis_trailli:1,Cheirolepis_schultzei:5.818375651):3.895277444,((Polypteridae_Erpetoichthys_calabaricus:29.200001,Polypteridae_Polypterus_senegalus:29.200001):365.800001,(Osorioichthys_marginis:23.38381077,((Tegeolepis_clarki:19.96057975,Howqualepis_rostridens:1):2.043441119,((Gogosardina_coatesi:5.193800239,(Mimipiscis_bartrami:1,Mimipiscis_toombsi:5.235976478):1):5.240452383,((Moythomasia_lineata:1,Moythomasia_durgaringa:8.064527705):1.945813011,(Stegotrachelus_finlayi:1,(Limnomis_delaneyi:1,(Wendyichthys_dicksoni:28.00418298,(Kentuckia_deani:1,((Mesopoma_planti:4.499881203,Mesopoma_carricki:1):6.397285618,((Birgeria_stensioei:1,(Chondrosteus_acipenseroides:1,(Saurichthys_dawaziensis:1,((Protopsephurus_liui:1,Polyodontidae_Polyodon_spathula:130.2228829):8.677119119,(Acipenseridae_Acipenser_fulvescens:38.579335,(Acipenseridae_Scaphirhynchus_platorynchus:19.375515,Acipenseridae_Scaphirhynchus_albus:19.375515):19.20382):100.320667):25.11334319):37.08781241):51.66615846):97.33268495,(Boreosomus:75.9421163,(((Perleidus_altolepis:85.93978715,(Luganoia_lepidosteoides:89.96596177,(((Macrosemius_fourneti:91.61870457,((Semionotus_elegans:1,Lepisosteidae_Lepisosteus_osseus:201.6062609):44.67622098,Kyphosichthys_grandei:1):1):20.61751916,(Watsonulus_eugnathoides:1,(Amblysemius:1,(Pachyamia_latimaxillaris:4.786275821,(Tomognathus_mordax:1,Amiidae_Amia_calva:100.3423334):1):48.04483551):103.304114):15.20871812):54.6,(Pachycormus:104.4453271,(Crossognathus_danubiensis:173.8844981,(((Anaethalion_angustus:1,Anaethalion_knorri:1.151784781):47.92382695,((Megalopidae_Megalops_atlanticus:133.565966,Elopidae_Elops_saurus:133.565966):62.634035,(Albulidae_Albula_vulpes:150.800001,((Notacanthidae_Notacanthus_chemnitzii:50.669287,(Halosauridae_Halosauropsis_macrochir:40.366211,Halosauridae_Aldrovandia_affinis:40.366211):10.303076):50.330714,(((Eurypharyngidae_Eurypharynx_pelecanoides:25.866364,Saccopharyngidae_Saccopharynx_ampullaceus:25.866364):44.322494,(Nemichthyidae_Nemichthys_scolopaceus:58.739485,Anguillidae_Anguilla_rostrata:58.739485):11.449373):9.198895,(Serrivomeridae_Serrivomer_beanii:76.053487,(Congridae_Conger_oceanicus:65.416147,(Muraenesocidae_Muraenesox_cinereus:57.193624,Ophichthidae_Myrichthys_maculosus:57.193624):8.222523):10.63734):3.334266):21.612248):49.8):45.4):1):86.1,((Lycoptera_davidi:98.35683457,(Paralycoptera_wui:106.6748285,(Xixiaichthys_tongxinens:98.82781059,(((Eohiodon_woodruffi:1,Eohiodon_rosei:2.772418972):1,(Hiodon_consteniorum:1,(Hiodontidae_Hiodon_tergisus:9.520291,Hiodontidae_Hiodon_alosoides:9.520291):26.63899429):21.44503334):169.4956824,(Chauliopareion_mahengeense:121.2267427,(Pantodontidae_Pantodon_buchholzi:163.100001,(((Arapaimidae_Arapaima_gigas:79.3,Osteoglossidae_Osteoglossum_bicirrhosum:79.3):25.549112,(Brychaetus_muelleri:1,Phareodus_encaustus:8.154966587):51.22173442):43.475578,(Notopteridae_Xenomystus_nigri:120.300002,Gymnarchidae_Gymnarchus_niloticus:120.300002):28.024688):14.775311):1):63):1):1):1):43.7,((((Diplomystus_brevissimus:50.11363391,Sorbinichthys_africanus:1):90.16784693,(Denticipitidae_Denticeps_clupeoides:188.900001,(Chirocentridae_Chirocentrus_dorab:87.096454,((Engraulidae_Coilia_nasus:47.06527,Engraulidae_Engraulis_mordax_eurystole:47.06527):33.347234,((Pristigasteridae_Pellona_flavipinnis:27.450803,Pristigasteridae_Ilisha_elongata:27.450803):46.430359,(Clupeidae_Alosa_pseudoharengus:54.138864,Clupeidae_Dorosoma_cepedianum:54.138864):19.742298):6.531342):6.68395):101.803547):1):40.3,((Alepocephalidae_Talismania_bifurcata:53.266521,((Alepocephalidae_Bathylaco_nigricans:33.802485,Alepocephalidae_Alepocephalus_tenebrosus:33.802485):10.988666,((Alepocephalidae_Rouleina_attrita:22.275085,Alepocephalidae_Xenodermichthys_copei:22.275085):19.924916,Platytroctidae_Sagamichthys_abei:42.200001):2.59115):8.47537):166.430015,((Gonorynchidae_Gonorynchus_abbreviatus:175.900001,(Mahengichthys_singidaensis:104.0849897,Chanidae_Chanos_chanos:147.100001):28.8):22.7,((((Gyrinocheilidae_Gyrinocheilus_sp:70.111694,(Catostomidae_Hypentelium_nigricans:23.594381,(Catostomidae_Carpiodes_carpio:16.355089,Catostomidae_Ictiobus_bubalus:16.355089):7.239292):46.517313):8.688307,Cobitidae_Cobitis_taenia:78.800001):20.5,(Cyprinidae_Danio_rerio:63.300001,(((Cyprinidae_Zacco_sieboldii_platypus:12.727958,Cyprinidae_Opsariichthys_uncirostris_bidens:12.727958):14.610086,((Cyprinidae_Xenocypris_argentea:12.904177,Cyprinidae_Hypophthalmichthys_molitrix:12.904177):2.917503,(Cyprinidae_Luciobrama_macrocephalus:13.439618,(Cyprinidae_Squaliobarbus_curriculus:11.472717,Cyprinidae_Mylopharyngodon_piceus:11.472717):1.966901):2.382062):11.516364):12.401957,(Cyprinidae_Tanakia_lanceolata_himantegus:35.004261,(Cyprinidae_Notemigonus_crysoleucas:22.933404,(Cyprinidae_Semotilus_atromaculatus:21.325215,((Cyprinidae_Campostoma_oligolepis:12.800846,Cyprinidae_Rhinichthys_cataractae:12.800846):4.408707,(Cyprinidae_Phenacobius_uranops:12.822623,(Cyprinidae_Pimephales_promelas_notatus:9.460336,(Cyprinidae_Luxilus_coccogenis:6.738861,Cyprinidae_Notropis_asperifrons:6.738861):2.721475):3.362287):4.38693):4.115662):1.608189):12.070857):4.73574):23.56):36):73.6,((Gymnotidae_Electrophorus_electricus:63.909447,Gymnotidae_Gymnotus_sp:63.909447):83.924067,(((Distichodontidae_Distichodus_maculatus:103.604365,Citharinidae_Citharinus_congicus:103.604365):11.170138,(Alestidae_Alestes_baremoze:106.666979,((Parodontidae_Parodon_nasus:68.800001,Hemiodontidae_Hemiodus_immaculatus:68.800001):22.415197,((Bryconidae_Brycon_pesu:66.003418,(Gasteropelecidae_Thoracocharax_stellatus:27.746828,Gasteropelecidae_Gasteropelecus_sternicla:27.746828):38.25659):1.227196,Characidae_Astyanax_mexicanus:67.230614):23.984584):15.451781):8.107524):22.236489,((Nematogenyidae_Nematogenys_inermis:110.648758,(Trichomycteridae_Trichomycterus_sp:105.886466,((Loricariidae_Loricaria_simillima:70.313425,Astroblepidae_Astroblepus_sp:70.313425):28.122359,(Callichthyidae_Callichthys_callichthys:69.200001,Callichthyidae_Corydoras_trilineatus:69.200001):29.235783):7.450682):4.762292):5.151243,(Diplomystidae_Diplomystes_nahuelbutaensis:106.203801,((Clariidae_Clarias_batrachus:47.60069,Heteropneustidae_Heteropneustes_fossilis:47.60069):38.718856,(((Sisoridae_Bagarius_yarrelli:55.522058,(Amblycipitidae_Liobagrus_aequilabris:48.027606,Akysidae_Akysis_sp:48.027606):7.494452):21.358911,(Schilbeidae_Pseudeutropius_brachypopterus:74.448774,(Bagridae_Mystus_bocourti:36.077943,Bagridae_Bagrus_ubangensis:36.077943):38.370831):2.432195):8.408138,(((Chacidae_Chaca_sp:72.429482,Cetopsidae_Cetopsis_coecutiens:72.429482):10.714797,(Cranoglanididae_Cranoglanis_bouderius:68.700001,Ictaluridae_Ictalurus_punctatus:68.700001):14.444278):1.039619,(Pangasiidae_Pangasianodon_hypophthalmus:82.158061,((Amphiliidae_Amphilius_jacksonii:69.199757,(Malapteruridae_Malapterurus_beninensis:64.740321,Mochokidae_Synodontis_batesii:64.740321):4.459436):10.993535,((Plotosidae_Plotosus_lineatus:73.039645,Siluridae_Hemisilurus_moolenburghi:73.039645):5.503862,(Auchenipteridae_Ageneiosus_atronasus:51.101654,Doradidae_Anduzedoras_oxyrhynchus:51.101654):27.441853):1.649785):1.964769):2.025837):1.105209):1.030439):19.884255):9.5962):21.210991):10.822522):25.066487):25.7):21.096535):10.503465):20.4,(Orthogonikleithrus_francogalliensis:77.29134206,(((Bathylagidae_Bathylagus_euryops:70.542512,(Microstomatidae_Nansenia_longicauda_ardesiaca:51.394,(Opisthoproctidae_Macropinna_microstoma:33.11268,Argentinidae_Argentina_sialis_silus:33.11268):18.28132):19.148512):89.057489,((Galaxiidae_Neochanna_burrowsius:34.505049,Galaxiidae_Galaxias_maculatus:34.505049):111.121934,(((Umbridae_Novumbra_hubbsi:55.762491,Umbridae_Umbra_limi:55.762491):23.63751,(Esocidae_Esox_lucius:31.366195,Esocidae_Esox_americanus:31.366195):48.033806):25.064928,(Salmonidae_Coregonus_clupeaformis:35.300001,(Salmonidae_Thymallus_brevirostris:32.456522,(Salmonidae_Oncorhynchus_nerka_mykiss:19.929533,(Salmonidae_Salvelinus_alpinus:16.737138,Salmonidae_Salmo_salar:16.737138):3.192395):12.526989):2.843479):69.164928):41.162054):13.973018):55.253787,(((Retropinnidae_Retropinna_semoni:73.700001,(((Osmeridae_Osmerus_mordax:10.332899,Osmeridae_Thaleichthys_pacificus:10.332899):5.872101,(Osmeridae_Mallotus_villosus:14.961758,Osmeridae_Hypomesus_pretiosus:14.961758):1.243242):18.133836,(Plecoglossidae_Plecoglossus_altivelis:28.70318,Salangidae_Neosalangichthys_ishikawae:28.70318):5.635656):39.361165):55.7,((Diplophidae_Diplophos_taenia:77.330737,((Gonostomatidae_Bonapartia_pedaliota:54.297445,Gonostomatidae_Margrethia_obtusirostra:54.297445):20.014374,(Gonostomatidae_Gonostoma_elongatum:69.948163,Gonostomatidae_Cyclothone_microdon:69.948163):4.363656):3.018918):6.469264,(Phosichthyidae_Pollichthys_mauli:77.722827,((Sternoptychidae_Argyropelecus_gigas:25.230481,Sternoptychidae_Maurolicus_weitzmani:25.230481):47.329876,(Stomiidae_Chauliodus_macouni_danae:63.126299,(Stomiidae_Stomias_boa:36.381906,(Stomiidae_Chirostomias_pliopterus:31.578039,((Stomiidae_Melanostomias_margaritifer:21.790927,Stomiidae_Leptostomias_longibarba:21.790927):6.240428,((Stomiidae_Photonectes_margarita:19.053467,Stomiidae_Tactostoma_macropus:19.053467):7.977888,(Stomiidae_Malacosteus_niger:25.031355,(Stomiidae_Eustomias_polyaster:24.031355,(Stomiidae_Aristostomias_scintillans:23.031355,Stomiidae_Bathophilus_flemingi_pawneei:23.031355):1):1):2):1):3.546684):4.803867):26.744393):9.434058):5.16247):6.077174):45.6):81.9,((Ateleopodidae_Ateleopus_japonicus:8.089622,Ateleopodidae_Ijimaia_antillarum:8.089622):184.210379,(((Synodontidae_Trachinocephalus_myops:34.928634,Synodontidae_Synodus_foetens:34.928634):78.971367,((Paraulopidae_Paraulopus_oblongus:101.985288,(Synodontidae_Saurida_gracilis:50.257509,Synodontidae_Harpadon_microchir:50.257509):51.727779):8.326796,(((Aulopidae_Aulopus_filamentosus:47.160387,Bathysauridae_Bathysaurus_ferox:47.160387):10.843902,(Ipnopidae_Ipnops_murrayi:13.82,Ipnopidae_Ipnops_agassizi:13.82):44.184289):23.635948,((Ipnopidae_Bathypterois_atricolor:69.328167,(Giganturidae_Gigantura_indica:13.525058,Giganturidae_Gigantura_chuni:13.525058):55.803109):6.462647,(Scopelarchidae_Benthalbella_macropinna:62.490696,(Chlorophthalmidae_Chlorophthalmus_agassizi:61.13635,(Sudidae_Sudis_atrox:55.026295,((Alepisauridae_Anotopterus_pharao:45.661943,(Alepisauridae_Omosudis_lowii:36.284216,Alepisauridae_Alepisaurus_ferox:36.284216):9.377727):8.238175,(Paralepididae_Stemonosudis_intermedia_macrura:50.122687,(Evermannellidae_Evermannella_balbo:46.686813,Paralepididae_Paralepis_coregonoides:46.686813):3.435874):3.777431):1.126177):6.110055):1.354346):13.300118):5.849423):28.671847):3.587917):68.9,(((Neoscopelidae_Neoscopelus_macrolepidotus:42.408365,Neoscopelidae_Scopelengys_tristis:42.408365):31.191636,((Myctophidae_Lampadena_speculigera:39.81673,Myctophidae_Lampanyctus_macdonaldi:39.81673):11.872,(Myctophidae_Benthosema_glaciale:28.30241,Myctophidae_Myctophum_punctatum:28.30241):23.38632):21.911271):99.5,(((Lamprididae_Lampris_guttatus:65.552505,(Regalecidae_Regalecus_russelii:37.682943,Trachipteridae_Trachipterus_trachypterus:37.682943):27.869562):84.747496,(((Mcconichthys_longipinnis:1,(Aphredoderidae_Aphredoderus_sayanus:41.500001,Amblyopsidae_Chologaster_cornuta:41.500001):25.38383023):1,(Lateopisciculus_turrifumosus:1,(Percopsidae_Percopsis_omiscomaycus:14.617149,Percopsidae_Percopsis_transmontana:14.617149):44.94163509):8.325047144):67.10650077,(Zeidae_Zeus_faber:107.100001,(Stylephoridae_Stylephorus_chordatus:78.800001,(Merlucciidae_Merluccius_productus:45.655086,(((Macrouridae_Gadomus_dispar:9.947595,Macrouridae_Bathygadus_favosus:9.947595):28.939269,(Steindachneriidae_Steindachneria_argentea:35.929071,((Macrouridae_Malacocephalus_laevis:17.761718,Macrouridae_Trachonurus_sulcatus:17.761718):10.126413,(Macrouridae_Coelorinchus_caribbaeus:21.883517,Macrouridae_Coryphaenoides_armatus:21.883517):6.004614):8.04094):2.957793):2.613137,((Moridae_Laemonema_goodebeanorum:25.08833,(Moridae_Halargyreus_johnsonii:16.444614,(Moridae_Lepidion_ensiferus:5.35693,Moridae_Antimora_rostrata:5.35693):11.087684):8.643716):13.1716,((Phycidae_Urophycis_tenuis:5.240351,Phycidae_Urophycis_chuss:5.240351):28.607969,(Lotidae_Gaidropsarus_ensis:29.800286,(Lotidae_Lota_lota:23.935071,(Gadidae_Melanogrammus_aeglefinus:5.86839,Gadidae_Gadus_morhua:5.86839):18.066681):5.865215):4.048034):4.41161):3.240071):4.155085):33.144915):28.3):27.890331):15.309669):11.08283,(Polymixiidae_Polymixia_japonica:154.800001,(((Diretmidae_Diretmus_argenteus:53.99855,(Monocentridae_Monocentris_japonica:33.600001,((Trachichthyidae_Hoplostethus_occidentalis_atlanticus:24.361165,Trachichthyidae_Gephyroberyx_darwinii:24.361165):7.919156,(Anoplogastridae_Anoplogaster_cornuta:31.22776,Trachichthyidae_Paratrachichthys_sajademalensis:31.22776):1.052561):1.31968):20.398549):71.275924,((Berycidae_Beryx_decadactylus:70.612394,(Melamphaidae_Poromitra_crassiceps:39.138751,(Melamphaidae_Scopelogadus_beanii:32.603956,Melamphaidae_Melamphaes_suborbitalis:32.603956):6.534795):31.473643):40.295775,(((Rondeletiidae_Rondeletia_bicolor:18.616456,Rondeletiidae_Rondeletia_loricata:18.616456):36.662305,Barbourisiidae_Barbourisia_rufa:55.278761):34.605685,(Cetomimidae_Cetostoma_regani:55.492218,(Cetomimidae_Cetomimus_craneae:10.537863,Cetomimidae_Cetomimus_compuctus:10.537863):44.954355):34.392228):21.023723):14.366305):21.189147,((Holocentridae_Sargocentron_diadema:25.43456,Holocentridae_Holocentrus_rufus:25.43456):119.565441,((Ophidiidae_Brotula_multibarbata:66.227334,(Ophidiidae_Lepophidium_profundorum:23.036156,(Ophidiidae_Genypterus_blacodes:21.590629,Ophidiidae_Ophidion_holbrookii:21.590629):1.445527):43.191178):66.572667,((Batrachoididae_Porichthys_notatus:39.800001,Batrachoididae_Opsanus_tau:39.800001):87,(((Kurtidae_Kurtus_indicus:80.300001,Apogonidae_Apogon_campbelli:80.300001):22,(Eleotridae_Eleotris_acanthopoma_pisonis:64.430477,(Gobiidae_Gobiosoma_bosc:45.630567,Microdesmidae_Microdesmus_longipinnis:45.630567):18.79991):37.869524):19.3,((((Dactylopteridae_Dactylopterus_volitans:68.121568,(Aulostomidae_Aulostomus_maculatus:3.200001,Aulostomidae_Aulostomus_chinensis:3.200001):64.921567):6.178433,((Fistulariidae_Fistularia_petimba:65.463647,Mullidae_Mullus_auratus:65.463647):6.26906,(Callionymidae_Callionymus_sp_bairdi:69.154942,Syngnathidae_Syngnathus_fuscus:69.154942):2.577765):2.567294):20.27,(Chiasmodontidae_Chiasmodon_sp:43.900001,((Centrolophidae_Icichthys_lockingtoni:34.370774,Pomatomidae_Pomatomus_saltatrix:34.370774):6.934458,(((Ariommatidae_Ariomma_bondi:25.644121,Nomeidae_Psenes_cyanophrys:25.644121):5.370208,Scombridae_Auxis_rochei:31.014329):6.588438,((Stromateidae_Peprilus_paru:35.602767,(Gempylidae_Paradiplospinus_gracilis:31.499638,Bramidae_Brama_brama:31.499638):4.103129):1,(Icosteidae_Icosteus_aenigmaticus:33.972252,Trichiuridae_Trichiurus_lepturus:33.972252):2.630515):1):3.702465):2.594769):50.67):21.916687,((((((Synbranchidae_Monopterus_albus:69.146846,Indostomidae_Indostomus_paradoxus:69.146846):4.548479,(Mastacembelidae_Macrognathus_siamensis:18.057555,Mastacembelidae_Mastacembelus_erythrotaenia:18.057555):55.63777):7.004676,((Anabantidae_Ctenopoma_acutirostre_kingsleyae:62.782323,(Helostomatidae_Helostoma_temminkii:59.687826,Osphronemidae_Trichopodus_pectoralis:59.687826):3.094497):7.86905,(Channidae_Channa_striata:67.428514,Nandidae_Nandus_nandus:67.428514):3.222859):10.048628):15.7,((Nematistiidae_Nematistius_pectoralis:60.067614,((Coryphaenidae_Coryphaena_hippurus:19.124214,Rachycentridae_Rachycentron_canadum:19.124214):12.775787,(Echeneidae_Remora_osteochir_australis:10.178122,Echeneidae_Echeneis_naucrates:10.178122):21.721879):28.167613):9.732387,((Sphyraenidae_Sphyraena_sphyraena:65.900885,(Menidae_Mene_maculata:60.339531,Polynemidae_Polydactylus_octonemus:60.339531):5.561354):2.899116,((((Leptobramidae_Leptobrama_muelleri:46.58559,Toxotidae_Toxotes_jaculatrix:46.58559):5.096327,(Xiphiidae_Xiphias_gladius:27.833842,Istiophoridae_Istiophorus_platypterus:27.833842):23.848075):5.298938,Carangidae_Trachinotus_falcatus:56.980855):4.861848,((Eolates_gracilis:1,(Centropomidae_Psammoperca_waigiensis:33.068144,(Centropomidae_Lates_calcarifer:13.595548,Centropomidae_Lates_microlepis:13.595548):19.472596):20.54585343):7.228705567,(Heteronectes_chaneti:7.359428185,(Amphistium_paradoxum:5.801031467,(Psettodidae_Psettodes_erumei:57.842703,(Citharidae_Lepidoblepharon_ophthalmolepis:53.600001,((Cynoglossidae_Cynoglossus_interruptus:42.400002,(Soleidae_Solea_solea:24.944331,Soleidae_Soleichthys_heterorhinos:24.944331):17.455671):9.674607,((Scophthalmidae_Scophthalmus_aquosus:23.305906,Scophthalmidae_Lepidorhombus_boscii:23.305906):26.973285,((Paralichthyidae_Paralichthys_dentatus:40.749719,Bothidae_Bothus_lunatus:40.749719):4.387561,(Paralichthyidae_Pseudorhombus_pentophthalmus:21.404631,(((Pleuronectidae_Glyptocephalus_zachirus:5.096079,Pleuronectidae_Microstomus_pacificus:5.096079):2.538752,(Pleuronectidae_Lyopsetta_exilis:6.085211,Pleuronectidae_Hippoglossus_hippoglossus:6.085211):1.54962):2.215451,(Pleuronectidae_Limanda_limanda:8.798272,((Pleuronectidae_Platichthys_stellatus:3.717184,Pleuronectidae_Pleuronectes_platessa:3.717184):3.081088,(Pleuronectidae_Lepidopsetta_bilineata:4.798272,Pleuronectidae_Psettichthys_melanostictus:4.798272):2):2):1.05201):11.554349):23.732649):5.141911):1.795418):1.525392):4.242702):1):1):1):1):6.957298):1):26.6):12.043384,(((Polycentridae_Polycentrus_schomburgkii:94.214904,(Pholidichthyidae_Pholidichthys_leucotaenia:88.700001,Cichlidae_Cichla_temensis:88.700001):5.514903):1,(((Atherinopsidae_Atherinopsis_californiensis:48.715388,Atherinopsidae_Menidia_menidia:48.715388):22.225577,(Isonidae_Iso_sp:49.317292,(Atherinidae_Atherinomorus_stipes:43.78646,(Melanotaeniidae_Melanotaenia_splendida:39.870052,Pseudomugilidae_Pseudomugil_gertrudae:39.870052):3.916408):5.530832):21.623673):6.46052,(((Poeciliidae_Poecilia_latipinna_reticulata:14.292152,(Poeciliidae_Heterandria_formosa:11.366823,(Poeciliidae_Gambusia_affinis:5.736451,Poeciliidae_Belonesox_belizanus:5.736451):5.630372):2.925329):30.13151,(Cyprinodontidae_Cyprinodon_variegatus:40.469994,Fundulidae_Fundulus_parvipinnis:40.469994):3.953668):31.977823,(Adrianichthyidae_Oryzias_latipes:71.878571,((Zenarchopteridae_Dermogenys_collettei:29.883878,((Belonidae_Tylosurus_crocodilus:6.568056,Belonidae_Ablennes_hians:6.568056):17.429484,(Scomberesocidae_Cololabis_saira:2.646712,Scomberesocidae_Scomberesox_saurus:2.646712):21.350828):5.886338):17.178962,(Hemiramphidae_Hyporhamphus_affinis:36.892311,((Hemiramphidae_Hemiramphus_brasiliensis:13.391766,Hemiramphidae_Oxyporhamphus_micropterus:13.391766):10.259915,((Exocoetidae_Cypselurus_callopterus:6.413654,Exocoetidae_Exocoetus_monocirrhus:6.413654):4.605967,Exocoetidae_Cheilopogon_pinnatibarbatus:11.019621):12.63206):13.24063):10.170529):24.815731):4.522914):1):17.813419):1.999954,((Pseudochromidae_Halidesmus_scapularis:84.850287,Pomacentridae_Pomacentrus_brachialis:84.850287):8.187103,(Embiotocidae_Cymatogaster_aggregata:91.529056,(Mugilidae_Mugil_cephalus:89.757655,(Plesiopidae_Plesiops_coeruleolineatus:86.977996,(Opistognathidae_Opistognathus_maxillosus:79.196597,(((Gobiesocidae_Lepadichthys_lineatus:37.560081,Gobiesocidae_Gobiesox_maeandricus:37.560081):35.985217,Tripterygiidae_Enneanectes_altivelis:73.545298):3.122761,((Clinidae_Clinus_cottoides:50.741024,(Dactyloscopidae_Platygillellus_rubrocinctus:45.641062,Chaenopsidae_Chaenopsis_alepidota:45.641062):5.099962):17.520576,(Blenniidae_Stanulus_seychellensis:31.042358,Blenniidae_Blenniella_cyanostigma:31.042358):37.219242):8.406459):2.528538):7.781399):2.779659):1.771401):1.508334):4.177468):11.228527):3.450865,(Gerreidae_Eucinostomus_argenteus:108.000002,(((Labridae_Lachnolaimus_maximus:59.822938,Odacidae_Haletta_semifasciata:59.822938):16.826114,Scaridae_Scarus_globiceps:76.649052):30.35095,(((Uranoscopidae_Uranoscopus_sulphureus:83.996275,(Ammodytidae_Ammodytes_hexapterus:78.584442,Pinguipedidae_Parapercis_punctulata:78.584442):5.411833):11.903726,(((Acropomatidae_Acropoma_japonicum:90.360051,((Percophidae_Acanthaphritis_unoorum:66.16617,Creediidae_Limnichthys_sp:66.16617):18.796708,(Glaucosomatidae_Glaucosoma_buergeri:66.617288,Pempheridae_Pempheris_schomburgkii:66.617288):18.34559):5.397173):2.814411,(((Oplegnathidae_Oplegnathus_punctatus:61.206964,Kuhliidae_Kuhlia_rupestris:61.206964):7.298332,Kyphosidae_Kyphosus_sectatrix:68.505296):16.157201,(Percichthyidae_Percichthys_trucha:69.999181,((Cirrhitidae_Cirrhitichthys_falco:66.467051,Cheilodactylidae_Cheilodactylus_fasciatus:66.467051):2.53213,(Enoplosidae_Enoplosus_armatus:63.04721,Centrarchidae_Acantharchus_pomotis:63.04721):5.951971):1):14.663316):8.511965):1.725539,(Serranidae_Pseudogramma_polyacantha:82.200001,(Percidae_Perca_fluviatilis:78.577195,((Bovichtidae_Bovichtus_diacanthus:63.021097,(Nototheniidae_Notothenia_coriiceps:9.671082,(Harpagiferidae_Harpagifer_antarcticus:8.390321,(Bathydraconidae_Gymnodraco_acuticeps:7.390321,Channichthyidae_Chionodraco_rastrospinosus:7.390321):1):1.280761):53.350015):12.936917,(((Peristediidae_Peristedion_ecuadorense:54.532565,Triglidae_Prionotus_carolinus:54.532565):11.953546,(Synanceiidae_Synanceia_verrucosa:62.510934,Scorpaenidae_Scorpaenodes_guamensis:62.510934):3.975177):6.621302,(Platycephalidae_Platycephalus_indicus:70.795272,(Anoplopomatidae_Anoplopoma_fimbria:46.504594,((Bathymasteridae_Rathbunella_hypoplecta:22.798552,(Stichaeidae_Cebidichthys_violaceus:15.58542,(Zoarcidae_Zoarces_americanus_viviparus:12.493576,(Anarhichadidae_Anarrhichthys_ocellatus:9.933504,(Zaproridae_Zaprora_silenus:8.933504,Cryptacanthodidae_Cryptacanthodes_maculatus:8.933504):1):2.560072):3.091844):7.213132):19.028758,(((Aulorhynchidae_Aulorhynchus_flavidus:25.280385,Hypoptychidae_Hypoptychus_dybowskii:25.280385):1.019618,((Gasterosteidae_Culaea_inconstans:19.844899,(Gasterosteidae_Apeltes_quadracus:17.136644,Gasterosteidae_Spinachia_spinachia:17.136644):2.708255):1.782129,(Gasterosteidae_Pungitius_pungitius:16.827665,Gasterosteidae_Gasterosteus_aculeatus:16.827665):4.799363):4.672975):13.53479,(Hexagrammidae_Hexagrammos_decagrammus:27.818969,((Cyclopteridae_Cyclopterus_lumpus:20.762394,Liparidae_Liparis_pulchellus:20.762394):4.037609,(Agonidae_Hypsagonus_quadricornis:20.371382,(Cottidae_Cottus_carolinae:18.19359,Psychrolutidae_Psychrolutes_phrictus:18.19359):2.177792):4.428621):3.018966):12.015824):1.992517):4.677284):24.290678):2.312141):2.850601):2.619181):3.622806):12.7):1):10.100001,((Drepaneidae_Drepane_punctata:67.658011,Ephippidae_Chaetodipterus_faber:67.658011):37.341991,(((Lobotidae_Lobotes_pacificus_surinamensis:86.438004,Sciaenidae_Menticirrhus_undulatus_littoralis:86.438004):5.761997,((Monodactylidae_Monodactylus_sebae:70.333247,(Avitoluvarus_eocaenicus:19.73560215,(Zanclidae_Zanclus_cornutus:55.859022,Acanthuridae_Acanthurus_triostegus:55.859022):4.740979):9.733246):15.366754,((Pomacanthidae_Pomacanthus_semicirculatus:54.434118,(Leiognathidae_Leiognathus_equulus:32.700001,Chaetodontidae_Chelmon_rostratus:32.700001):21.734117):17.438962,(Emmelichthyidae_Erythrocles_schlegelii:69.871718,(Malacanthidae_Malacanthus_plumieri:68.303733,(Haemulidae_Haemulon_aurolineatum:64.600001,Lutjanidae_Lutjanus_griseus:64.600001):3.703732):1.567985):2.001362):13.826921):6.5):11.800001,((Sillaginidae_Sillago_sihama:85.853361,(Nemipteridae_Pentapodus_caninus:80.270573,(Lethrinidae_Lethrinus_erythropterus:74.370539,Sparidae_Stenotomus_chrysops:74.370539):5.900034):5.582788):17.146641,(Siganidae_Siganus_spinus:102.000002,((Scatophagidae_Scatophagus_argus:75.860371,Priacanthidae_Heteropriacanthus_cruentatus:75.860371):25.139631,((Caproidae_Antigonia_rubescens:10.905674,Caproidae_Antigonia_capros:10.905674):89.094328,(((Lophiidae_Lophiodes_reticulatus:22.462185,Lophiidae_Lophius_gastrophysus:22.462185):42.032119,((Antennariidae_Histrio_histrio:12.333102,Antennariidae_Antennatus_coccineus:12.333102):33.466899,(Chaunacidae_Chaunax_suttkusi:38.499563,(Gigantactinidae_Gigantactis_sp:32.911618,((Ceratiidae_Cryptopsaras_couesii:20.548708,Ceratiidae_Ceratias_holboelli:20.548708):10.416157,((Himantolophidae_Himantolophus_albinares_sagamius:14.456802,Melanocetidae_Melanocetus_murrayi:14.456802):9.124159,(Oneirodidae_Oneirodes_macrosteus:10.164357,Oneirodidae_Dolopichthys_sp:10.164357):13.416604):7.383904):1.946753):5.587945):7.300438):18.694303):34.505698,(((Prohollardia_avita:36.75972878,(Protacanthodes_nimesensis:1,Triacanthidae_Triacanthus_biaculeatus:48.64147799):15.85149301):10.123893,(Aracanidae_Aracana_aurita:54.800001,(Ostraciidae_Ostracion_cubicus:51.000002,Ostraciidae_Rhinesomus_triqueter:51.000002):3.799999):19.816863):3.183137,(((Diodontidae_Diodon_holocanthus:12.198387,Diodontidae_Chilomycterus_schoepfii:12.198387):43.701614,(Tetraodontidae_Canthigaster_bennetti:24.22571,Tetraodontidae_Tetraodon_miurus:24.22571):31.674291):17.334859,((Molidae_Ranzania_laevis:22,Molidae_Mola_mola:22):46.61027,((Monacanthidae_Aluterus_scriptus:27.823604,Monacanthidae_Stephanolepis_hispidus:27.823604):15.976396,(Balistidae_Xanthichthys_ringens:20.7,(Balistidae_Balistes_vetula:19.7,Balistidae_Sufflamen_fraenatum:19.7):1):23.1):24.81027):4.62459):4.565141):21.200001):1):1):1):1):1):1):1):1):1):3.894248):4.592438):5.113313):5.2):6):12.2):1.46362):8.33638):6.58283):11.71717):9.7):9.5):19):3.553787):17.946213):17.8):23.2):9.5):1):1):37.2):1):1):1,(Fukangichthys_longidorsalis:8.810741477,(Scanilepis_dubia:45.6368485,Evenkia_eunotoptera:1):1):71.4111131):1,Australosomus:74.59363579):1):22.6):1):3.083541186):1):17.50564306):14.89814155):1):1):1):1):3.412675198):1):47);

((Guiyu_oneiros:1,(Onychodus_jandemarrai:36.88382805,((Diplocercides:1,(Latimeriidae_Latimeria_chalumnae:329.3295244,Rhabdoderma:1):36.82973194):46.35637064,((Neoceratodontidae_Neoceratodus_forsteri:279.7982796,Lepidosirenidae_Lepidosiren_paradoxa:279.7982796):130.2333383,((Osteolepis_macrolepidotus:1,Gyroptychius_milleri:16.8448442):1,Eusthenopterus_foordi:29.32050441):1):2.484009141):1):12.80965443):16.67472057,((Cheirolepis_trailli:1,Cheirolepis_schultzei:4.545583799):4.045906891,((Polypteridae_Erpetoichthys_calabaricus:29.200001,Polypteridae_Polypterus_senegalus:29.200001):365.800001,(Osorioichthys_marginis:26.83675072,((Tegeolepis_clarki:18.0154422,Howqualepis_rostridens:1):3.719316266,((Gogosardina_coatesi:5.702854904,(Mimipiscis_bartrami:3.058710947,Mimipiscis_toombsi:1):1):10.59705418,((Moythomasia_lineata:1,Moythomasia_durgaringa:7.249561298):2.246366826,(Stegotrachelus_finlayi:1,(Limnomis_delaneyi:1,(Wendyichthys_dicksoni:32.69399423,(Kentuckia_deani:1,((Mesopoma_planti:1,Mesopoma_carricki:9.317143645):7.771396256,((Birgeria_stensioei:1,(Chondrosteus_acipenseroides:1,(Saurichthys_dawaziensis:1,((Protopsephurus_liui:1,Polyodontidae_Polyodon_spathula:130.2429889):8.657013067,(Acipenseridae_Acipenser_fulvescens:38.579335,(Acipenseridae_Scaphirhynchus_platorynchus:19.375515,Acipenseridae_Scaphirhynchus_albus:19.375515):19.20382):100.320667):25.16210565):37.28909628):51.24350865):97.50528843,(Boreosomus:75.68534669,(((Perleidus_altolepis:83.72147898,(Luganoia_lepidosteoides:88.91299165,(((Macrosemius_fourneti:90.89509497,((Lepisosteidae_Lepisosteus_osseus:201.9469192,Semionotus_elegans:1):44.90832558,Kyphosichthys_grandei:1):1):20.04475627,(Watsonulus_eugnathoides:1,(Amblysemius:1,(Pachyamia_latimaxillaris:3.932081123,(Tomognathus_mordax:1,Amiidae_Amia_calva:99.44351275):1):52.3255898):100.399299):14.73159944):54.6,(Pachycormus:107.3772295,(Crossognathus_danubiensis:172.3867014,(((Anaethalion_angustus:3.578272872,Anaethalion_knorri:1):44.24899734,((Megalopidae_Megalops_atlanticus:133.565966,Elopidae_Elops_saurus:133.565966):62.634035,(Albulidae_Albula_vulpes:150.800001,((Notacanthidae_Notacanthus_chemnitzii:50.669287,(Halosauridae_Halosauropsis_macrochir:40.366211,Halosauridae_Aldrovandia_affinis:40.366211):10.303076):50.330714,(((Eurypharyngidae_Eurypharynx_pelecanoides:25.866364,Saccopharyngidae_Saccopharynx_ampullaceus:25.866364):44.322494,(Nemichthyidae_Nemichthys_scolopaceus:58.739485,Anguillidae_Anguilla_rostrata:58.739485):11.449373):9.198895,(Serrivomeridae_Serrivomer_beanii:76.053487,(Congridae_Conger_oceanicus:65.416147,(Muraenesocidae_Muraenesox_cinereus:57.193624,Ophichthidae_Myrichthys_maculosus:57.193624):8.222523):10.63734):3.334266):21.612248):49.8):45.4):1):86.1,((Lycoptera_davidi:97.92096613,(Paralycoptera_wui:104.8886152,(Xixiaichthys_tongxinens:99.75746505,(((Eohiodon_woodruffi:1,Eohiodon_rosei:1.726444551):1,(Hiodon_consteniorum:1,(Hiodontidae_Hiodon_tergisus:9.520291,Hiodontidae_Hiodon_alosoides:9.520291):28.84340518):18.39117894):170.3451259,(Chauliopareion_mahengeense:118.3683547,(Pantodontidae_Pantodon_buchholzi:163.100001,(((Arapaimidae_Arapaima_gigas:79.3,Osteoglossidae_Osteoglossum_bicirrhosum:79.3):25.549112,(Brychaetus_muelleri:1,Phareodus_encaustus:7.216821236):51.61111458):43.475578,(Notopteridae_Xenomystus_nigri:120.300002,Gymnarchidae_Gymnarchus_niloticus:120.300002):28.024688):14.775311):1):63):1):1):1):43.7,((((Diplomystus_brevissimus:47.14577885,Sorbinichthys_africanus:1):89.09699618,(Denticipitidae_Denticeps_clupeoides:188.900001,(Chirocentridae_Chirocentrus_dorab:87.096454,((Engraulidae_Coilia_nasus:47.06527,Engraulidae_Engraulis_mordax_eurystole:47.06527):33.347234,((Pristigasteridae_Pellona_flavipinnis:27.450803,Pristigasteridae_Ilisha_elongata:27.450803):46.430359,(Clupeidae_Alosa_pseudoharengus:54.138864,Clupeidae_Dorosoma_cepedianum:54.138864):19.742298):6.531342):6.68395):101.803547):1):40.3,((Alepocephalidae_Talismania_bifurcata:53.266521,((Alepocephalidae_Bathylaco_nigricans:33.802485,Alepocephalidae_Alepocephalus_tenebrosus:33.802485):10.988666,((Alepocephalidae_Rouleina_attrita:22.275085,Alepocephalidae_Xenodermichthys_copei:22.275085):19.924916,Platytroctidae_Sagamichthys_abei:42.200001):2.59115):8.47537):166.430015,((Gonorynchidae_Gonorynchus_abbreviatus:175.900001,(Mahengichthys_singidaensis:100.0176253,Chanidae_Chanos_chanos:147.100001):28.8):22.7,((((Gyrinocheilidae_Gyrinocheilus_sp:70.111694,(Catostomidae_Hypentelium_nigricans:23.594381,(Catostomidae_Carpiodes_carpio:16.355089,Catostomidae_Ictiobus_bubalus:16.355089):7.239292):46.517313):8.688307,Cobitidae_Cobitis_taenia:78.800001):20.5,(Cyprinidae_Danio_rerio:63.300001,(((Cyprinidae_Zacco_sieboldii_platypus:12.727958,Cyprinidae_Opsariichthys_uncirostris_bidens:12.727958):14.610086,((Cyprinidae_Xenocypris_argentea:12.904177,Cyprinidae_Hypophthalmichthys_molitrix:12.904177):2.917503,(Cyprinidae_Luciobrama_macrocephalus:13.439618,(Cyprinidae_Squaliobarbus_curriculus:11.472717,Cyprinidae_Mylopharyngodon_piceus:11.472717):1.966901):2.382062):11.516364):12.401957,(Cyprinidae_Tanakia_lanceolata_himantegus:35.004261,(Cyprinidae_Notemigonus_crysoleucas:22.933404,(Cyprinidae_Semotilus_atromaculatus:21.325215,((Cyprinidae_Campostoma_oligolepis:12.800846,Cyprinidae_Rhinichthys_cataractae:12.800846):4.408707,(Cyprinidae_Phenacobius_uranops:12.822623,(Cyprinidae_Pimephales_promelas_notatus:9.460336,(Cyprinidae_Luxilus_coccogenis:6.738861,Cyprinidae_Notropis_asperifrons:6.738861):2.721475):3.362287):4.38693):4.115662):1.608189):12.070857):4.73574):23.56):36):73.6,((Gymnotidae_Electrophorus_electricus:63.909447,Gymnotidae_Gymnotus_sp:63.909447):83.924067,(((Distichodontidae_Distichodus_maculatus:103.604365,Citharinidae_Citharinus_congicus:103.604365):11.170138,(Alestidae_Alestes_baremoze:106.666979,((Parodontidae_Parodon_nasus:68.800001,Hemiodontidae_Hemiodus_immaculatus:68.800001):22.415197,((Bryconidae_Brycon_pesu:66.003418,(Gasteropelecidae_Thoracocharax_stellatus:27.746828,Gasteropelecidae_Gasteropelecus_sternicla:27.746828):38.25659):1.227196,Characidae_Astyanax_mexicanus:67.230614):23.984584):15.451781):8.107524):22.236489,((Nematogenyidae_Nematogenys_inermis:110.648758,(Trichomycteridae_Trichomycterus_sp:105.886466,((Loricariidae_Loricaria_simillima:70.313425,Astroblepidae_Astroblepus_sp:70.313425):28.122359,(Callichthyidae_Callichthys_callichthys:69.200001,Callichthyidae_Corydoras_trilineatus:69.200001):29.235783):7.450682):4.762292):5.151243,(Diplomystidae_Diplomystes_nahuelbutaensis:106.203801,((Clariidae_Clarias_batrachus:47.60069,Heteropneustidae_Heteropneustes_fossilis:47.60069):38.718856,(((Sisoridae_Bagarius_yarrelli:55.522058,(Amblycipitidae_Liobagrus_aequilabris:48.027606,Akysidae_Akysis_sp:48.027606):7.494452):21.358911,(Schilbeidae_Pseudeutropius_brachypopterus:74.448774,(Bagridae_Mystus_bocourti:36.077943,Bagridae_Bagrus_ubangensis:36.077943):38.370831):2.432195):8.408138,(((Chacidae_Chaca_sp:72.429482,Cetopsidae_Cetopsis_coecutiens:72.429482):10.714797,(Cranoglanididae_Cranoglanis_bouderius:68.700001,Ictaluridae_Ictalurus_punctatus:68.700001):14.444278):1.039619,(Pangasiidae_Pangasianodon_hypophthalmus:82.158061,((Amphiliidae_Amphilius_jacksonii:69.199757,(Malapteruridae_Malapterurus_beninensis:64.740321,Mochokidae_Synodontis_batesii:64.740321):4.459436):10.993535,((Plotosidae_Plotosus_lineatus:73.039645,Siluridae_Hemisilurus_moolenburghi:73.039645):5.503862,(Auchenipteridae_Ageneiosus_atronasus:51.101654,Doradidae_Anduzedoras_oxyrhynchus:51.101654):27.441853):1.649785):1.964769):2.025837):1.105209):1.030439):19.884255):9.5962):21.210991):10.822522):25.066487):25.7):21.096535):10.503465):20.4,(Orthogonikleithrus_francogalliensis:78.49100122,(((Bathylagidae_Bathylagus_euryops:70.542512,(Microstomatidae_Nansenia_longicauda_ardesiaca:51.394,(Opisthoproctidae_Macropinna_microstoma:33.11268,Argentinidae_Argentina_sialis_silus:33.11268):18.28132):19.148512):89.057489,((Galaxiidae_Neochanna_burrowsius:34.505049,Galaxiidae_Galaxias_maculatus:34.505049):111.121934,(((Umbridae_Novumbra_hubbsi:55.762491,Umbridae_Umbra_limi:55.762491):23.63751,(Esocidae_Esox_lucius:31.366195,Esocidae_Esox_americanus:31.366195):48.033806):25.064928,(Salmonidae_Coregonus_clupeaformis:35.300001,(Salmonidae_Thymallus_brevirostris:32.456522,(Salmonidae_Oncorhynchus_nerka_mykiss:19.929533,(Salmonidae_Salvelinus_alpinus:16.737138,Salmonidae_Salmo_salar:16.737138):3.192395):12.526989):2.843479):69.164928):41.162054):13.973018):55.253787,(((Retropinnidae_Retropinna_semoni:73.700001,(((Osmeridae_Osmerus_mordax:10.332899,Osmeridae_Thaleichthys_pacificus:10.332899):5.872101,(Osmeridae_Mallotus_villosus:14.961758,Osmeridae_Hypomesus_pretiosus:14.961758):1.243242):18.133836,(Plecoglossidae_Plecoglossus_altivelis:28.70318,Salangidae_Neosalangichthys_ishikawae:28.70318):5.635656):39.361165):55.7,((Diplophidae_Diplophos_taenia:77.330737,((Gonostomatidae_Bonapartia_pedaliota:54.297445,Gonostomatidae_Margrethia_obtusirostra:54.297445):20.014374,(Gonostomatidae_Gonostoma_elongatum:69.948163,Gonostomatidae_Cyclothone_microdon:69.948163):4.363656):3.018918):6.469264,(Phosichthyidae_Pollichthys_mauli:77.722827,((Sternoptychidae_Argyropelecus_gigas:25.230481,Sternoptychidae_Maurolicus_weitzmani:25.230481):47.329876,(Stomiidae_Chauliodus_macouni_danae:63.126299,(Stomiidae_Stomias_boa:36.381906,(Stomiidae_Chirostomias_pliopterus:31.578039,((Stomiidae_Melanostomias_margaritifer:21.790927,Stomiidae_Leptostomias_longibarba:21.790927):6.240428,((Stomiidae_Photonectes_margarita:19.053467,Stomiidae_Tactostoma_macropus:19.053467):7.977888,(Stomiidae_Malacosteus_niger:25.031355,(Stomiidae_Eustomias_polyaster:24.031355,(Stomiidae_Aristostomias_scintillans:23.031355,Stomiidae_Bathophilus_flemingi_pawneei:23.031355):1):1):2):1):3.546684):4.803867):26.744393):9.434058):5.16247):6.077174):45.6):81.9,((Ateleopodidae_Ateleopus_japonicus:8.089622,Ateleopodidae_Ijimaia_antillarum:8.089622):184.210379,(((Synodontidae_Trachinocephalus_myops:34.928634,Synodontidae_Synodus_foetens:34.928634):78.971367,((Paraulopidae_Paraulopus_oblongus:101.985288,(Synodontidae_Saurida_gracilis:50.257509,Synodontidae_Harpadon_microchir:50.257509):51.727779):8.326796,(((Aulopidae_Aulopus_filamentosus:47.160387,Bathysauridae_Bathysaurus_ferox:47.160387):10.843902,(Ipnopidae_Ipnops_murrayi:13.82,Ipnopidae_Ipnops_agassizi:13.82):44.184289):23.635948,((Ipnopidae_Bathypterois_atricolor:69.328167,(Giganturidae_Gigantura_indica:13.525058,Giganturidae_Gigantura_chuni:13.525058):55.803109):6.462647,(Scopelarchidae_Benthalbella_macropinna:62.490696,(Chlorophthalmidae_Chlorophthalmus_agassizi:61.13635,(Sudidae_Sudis_atrox:55.026295,((Alepisauridae_Anotopterus_pharao:45.661943,(Alepisauridae_Omosudis_lowii:36.284216,Alepisauridae_Alepisaurus_ferox:36.284216):9.377727):8.238175,(Paralepididae_Stemonosudis_intermedia_macrura:50.122687,(Evermannellidae_Evermannella_balbo:46.686813,Paralepididae_Paralepis_coregonoides:46.686813):3.435874):3.777431):1.126177):6.110055):1.354346):13.300118):5.849423):28.671847):3.587917):68.9,(((Neoscopelidae_Neoscopelus_macrolepidotus:42.408365,Neoscopelidae_Scopelengys_tristis:42.408365):31.191636,((Myctophidae_Lampadena_speculigera:39.81673,Myctophidae_Lampanyctus_macdonaldi:39.81673):11.872,(Myctophidae_Benthosema_glaciale:28.30241,Myctophidae_Myctophum_punctatum:28.30241):23.38632):21.911271):99.5,(((Lamprididae_Lampris_guttatus:65.552505,(Regalecidae_Regalecus_russelii:37.682943,Trachipteridae_Trachipterus_trachypterus:37.682943):27.869562):84.747496,(((Mcconichthys_longipinnis:1,(Aphredoderidae_Aphredoderus_sayanus:41.500001,Amblyopsidae_Chologaster_cornuta:41.500001):25.02347287):1,(Lateopisciculus_turrifumosus:1,(Percopsidae_Percopsis_omiscomaycus:14.617149,Percopsidae_Percopsis_transmontana:14.617149):44.61593733):8.290387536):67.46685813,(Zeidae_Zeus_faber:107.100001,(Stylephoridae_Stylephorus_chordatus:78.800001,(Merlucciidae_Merluccius_productus:45.655086,(((Macrouridae_Gadomus_dispar:9.947595,Macrouridae_Bathygadus_favosus:9.947595):28.939269,(Steindachneriidae_Steindachneria_argentea:35.929071,((Macrouridae_Malacocephalus_laevis:17.761718,Macrouridae_Trachonurus_sulcatus:17.761718):10.126413,(Macrouridae_Coelorinchus_caribbaeus:21.883517,Macrouridae_Coryphaenoides_armatus:21.883517):6.004614):8.04094):2.957793):2.613137,((Moridae_Laemonema_goodebeanorum:25.08833,(Moridae_Halargyreus_johnsonii:16.444614,(Moridae_Lepidion_ensiferus:5.35693,Moridae_Antimora_rostrata:5.35693):11.087684):8.643716):13.1716,((Phycidae_Urophycis_tenuis:5.240351,Phycidae_Urophycis_chuss:5.240351):28.607969,(Lotidae_Gaidropsarus_ensis:29.800286,(Lotidae_Lota_lota:23.935071,(Gadidae_Melanogrammus_aeglefinus:5.86839,Gadidae_Gadus_morhua:5.86839):18.066681):5.865215):4.048034):4.41161):3.240071):4.155085):33.144915):28.3):27.890331):15.309669):11.08283,(Polymixiidae_Polymixia_japonica:154.800001,(((Diretmidae_Diretmus_argenteus:53.99855,(Monocentridae_Monocentris_japonica:33.600001,((Trachichthyidae_Hoplostethus_occidentalis_atlanticus:24.361165,Trachichthyidae_Gephyroberyx_darwinii:24.361165):7.919156,(Anoplogastridae_Anoplogaster_cornuta:31.22776,Trachichthyidae_Paratrachichthys_sajademalensis:31.22776):1.052561):1.31968):20.398549):71.275924,((Berycidae_Beryx_decadactylus:70.612394,(Melamphaidae_Poromitra_crassiceps:39.138751,(Melamphaidae_Scopelogadus_beanii:32.603956,Melamphaidae_Melamphaes_suborbitalis:32.603956):6.534795):31.473643):40.295775,(((Rondeletiidae_Rondeletia_bicolor:18.616456,Rondeletiidae_Rondeletia_loricata:18.616456):36.662305,Barbourisiidae_Barbourisia_rufa:55.278761):34.605685,(Cetomimidae_Cetostoma_regani:55.492218,(Cetomimidae_Cetomimus_craneae:10.537863,Cetomimidae_Cetomimus_compuctus:10.537863):44.954355):34.392228):21.023723):14.366305):21.189147,((Holocentridae_Sargocentron_diadema:25.43456,Holocentridae_Holocentrus_rufus:25.43456):119.565441,((Ophidiidae_Brotula_multibarbata:66.227334,(Ophidiidae_Lepophidium_profundorum:23.036156,(Ophidiidae_Genypterus_blacodes:21.590629,Ophidiidae_Ophidion_holbrookii:21.590629):1.445527):43.191178):66.572667,((Batrachoididae_Porichthys_notatus:39.800001,Batrachoididae_Opsanus_tau:39.800001):87,(((Kurtidae_Kurtus_indicus:80.300001,Apogonidae_Apogon_campbelli:80.300001):22,(Eleotridae_Eleotris_acanthopoma_pisonis:64.430477,(Gobiidae_Gobiosoma_bosc:45.630567,Microdesmidae_Microdesmus_longipinnis:45.630567):18.79991):37.869524):19.3,((((Dactylopteridae_Dactylopterus_volitans:68.121568,(Aulostomidae_Aulostomus_maculatus:3.200001,Aulostomidae_Aulostomus_chinensis:3.200001):64.921567):6.178433,((Fistulariidae_Fistularia_petimba:65.463647,Mullidae_Mullus_auratus:65.463647):6.26906,(Callionymidae_Callionymus_sp_bairdi:69.154942,Syngnathidae_Syngnathus_fuscus:69.154942):2.577765):2.567294):20.27,(Chiasmodontidae_Chiasmodon_sp:43.900001,((Centrolophidae_Icichthys_lockingtoni:34.370774,Pomatomidae_Pomatomus_saltatrix:34.370774):6.934458,(((Ariommatidae_Ariomma_bondi:25.644121,Nomeidae_Psenes_cyanophrys:25.644121):5.370208,Scombridae_Auxis_rochei:31.014329):6.588438,((Stromateidae_Peprilus_paru:35.602767,(Gempylidae_Paradiplospinus_gracilis:31.499638,Bramidae_Brama_brama:31.499638):4.103129):1,(Icosteidae_Icosteus_aenigmaticus:33.972252,Trichiuridae_Trichiurus_lepturus:33.972252):2.630515):1):3.702465):2.594769):50.67):21.916687,((((((Synbranchidae_Monopterus_albus:69.146846,Indostomidae_Indostomus_paradoxus:69.146846):4.548479,(Mastacembelidae_Macrognathus_siamensis:18.057555,Mastacembelidae_Mastacembelus_erythrotaenia:18.057555):55.63777):7.004676,((Anabantidae_Ctenopoma_acutirostre_kingsleyae:62.782323,(Helostomatidae_Helostoma_temminkii:59.687826,Osphronemidae_Trichopodus_pectoralis:59.687826):3.094497):7.86905,(Channidae_Channa_striata:67.428514,Nandidae_Nandus_nandus:67.428514):3.222859):10.048628):15.7,((Nematistiidae_Nematistius_pectoralis:60.067614,((Coryphaenidae_Coryphaena_hippurus:19.124214,Rachycentridae_Rachycentron_canadum:19.124214):12.775787,(Echeneidae_Remora_osteochir_australis:10.178122,Echeneidae_Echeneis_naucrates:10.178122):21.721879):28.167613):9.732387,((Sphyraenidae_Sphyraena_sphyraena:65.900885,(Menidae_Mene_maculata:60.339531,Polynemidae_Polydactylus_octonemus:60.339531):5.561354):2.899116,((((Leptobramidae_Leptobrama_muelleri:46.58559,Toxotidae_Toxotes_jaculatrix:46.58559):5.096327,(Xiphiidae_Xiphias_gladius:27.833842,Istiophoridae_Istiophorus_platypterus:27.833842):23.848075):5.298938,Carangidae_Trachinotus_falcatus:56.980855):4.861848,((Eolates_gracilis:1,(Centropomidae_Psammoperca_waigiensis:33.068144,(Centropomidae_Lates_calcarifer:13.595548,Centropomidae_Lates_microlepis:13.595548):19.472596):20.53012337):7.244435628,(Heteronectes_chaneti:5.520782702,(Amphistium_paradoxum:7.887654036,(Psettodidae_Psettodes_erumei:57.842703,(Citharidae_Lepidoblepharon_ophthalmolepis:53.600001,((Cynoglossidae_Cynoglossus_interruptus:42.400002,(Soleidae_Solea_solea:24.944331,Soleidae_Soleichthys_heterorhinos:24.944331):17.455671):9.674607,((Scophthalmidae_Scophthalmus_aquosus:23.305906,Scophthalmidae_Lepidorhombus_boscii:23.305906):26.973285,((Paralichthyidae_Paralichthys_dentatus:40.749719,Bothidae_Bothus_lunatus:40.749719):4.387561,(Paralichthyidae_Pseudorhombus_pentophthalmus:21.404631,(((Pleuronectidae_Glyptocephalus_zachirus:5.096079,Pleuronectidae_Microstomus_pacificus:5.096079):2.538752,(Pleuronectidae_Lyopsetta_exilis:6.085211,Pleuronectidae_Hippoglossus_hippoglossus:6.085211):1.54962):2.215451,(Pleuronectidae_Limanda_limanda:8.798272,((Pleuronectidae_Platichthys_stellatus:3.717184,Pleuronectidae_Pleuronectes_platessa:3.717184):3.081088,(Pleuronectidae_Lepidopsetta_bilineata:4.798272,Pleuronectidae_Psettichthys_melanostictus:4.798272):2):2):1.05201):11.554349):23.732649):5.141911):1.795418):1.525392):4.242702):1):1):1):1):6.957298):1):26.6):12.043384,(((Polycentridae_Polycentrus_schomburgkii:94.214904,(Pholidichthyidae_Pholidichthys_leucotaenia:88.700001,Cichlidae_Cichla_temensis:88.700001):5.514903):1,(((Atherinopsidae_Atherinopsis_californiensis:48.715388,Atherinopsidae_Menidia_menidia:48.715388):22.225577,(Isonidae_Iso_sp:49.317292,(Atherinidae_Atherinomorus_stipes:43.78646,(Melanotaeniidae_Melanotaenia_splendida:39.870052,Pseudomugilidae_Pseudomugil_gertrudae:39.870052):3.916408):5.530832):21.623673):6.46052,(((Poeciliidae_Poecilia_latipinna_reticulata:14.292152,(Poeciliidae_Heterandria_formosa:11.366823,(Poeciliidae_Gambusia_affinis:5.736451,Poeciliidae_Belonesox_belizanus:5.736451):5.630372):2.925329):30.13151,(Cyprinodontidae_Cyprinodon_variegatus:40.469994,Fundulidae_Fundulus_parvipinnis:40.469994):3.953668):31.977823,(Adrianichthyidae_Oryzias_latipes:71.878571,((Zenarchopteridae_Dermogenys_collettei:29.883878,((Belonidae_Tylosurus_crocodilus:6.568056,Belonidae_Ablennes_hians:6.568056):17.429484,(Scomberesocidae_Cololabis_saira:2.646712,Scomberesocidae_Scomberesox_saurus:2.646712):21.350828):5.886338):17.178962,(Hemiramphidae_Hyporhamphus_affinis:36.892311,((Hemiramphidae_Hemiramphus_brasiliensis:13.391766,Hemiramphidae_Oxyporhamphus_micropterus:13.391766):10.259915,((Exocoetidae_Cypselurus_callopterus:6.413654,Exocoetidae_Exocoetus_monocirrhus:6.413654):4.605967,Exocoetidae_Cheilopogon_pinnatibarbatus:11.019621):12.63206):13.24063):10.170529):24.815731):4.522914):1):17.813419):1.999954,((Pseudochromidae_Halidesmus_scapularis:84.850287,Pomacentridae_Pomacentrus_brachialis:84.850287):8.187103,(Embiotocidae_Cymatogaster_aggregata:91.529056,(Mugilidae_Mugil_cephalus:89.757655,(Plesiopidae_Plesiops_coeruleolineatus:86.977996,(Opistognathidae_Opistognathus_maxillosus:79.196597,(((Gobiesocidae_Lepadichthys_lineatus:37.560081,Gobiesocidae_Gobiesox_maeandricus:37.560081):35.985217,Tripterygiidae_Enneanectes_altivelis:73.545298):3.122761,((Clinidae_Clinus_cottoides:50.741024,(Dactyloscopidae_Platygillellus_rubrocinctus:45.641062,Chaenopsidae_Chaenopsis_alepidota:45.641062):5.099962):17.520576,(Blenniidae_Stanulus_seychellensis:31.042358,Blenniidae_Blenniella_cyanostigma:31.042358):37.219242):8.406459):2.528538):7.781399):2.779659):1.771401):1.508334):4.177468):11.228527):3.450865,(Gerreidae_Eucinostomus_argenteus:108.000002,(((Labridae_Lachnolaimus_maximus:59.822938,Odacidae_Haletta_semifasciata:59.822938):16.826114,Scaridae_Scarus_globiceps:76.649052):30.35095,(((Uranoscopidae_Uranoscopus_sulphureus:83.996275,(Ammodytidae_Ammodytes_hexapterus:78.584442,Pinguipedidae_Parapercis_punctulata:78.584442):5.411833):11.903726,(((Acropomatidae_Acropoma_japonicum:90.360051,((Percophidae_Acanthaphritis_unoorum:66.16617,Creediidae_Limnichthys_sp:66.16617):18.796708,(Glaucosomatidae_Glaucosoma_buergeri:66.617288,Pempheridae_Pempheris_schomburgkii:66.617288):18.34559):5.397173):2.814411,(((Oplegnathidae_Oplegnathus_punctatus:61.206964,Kuhliidae_Kuhlia_rupestris:61.206964):7.298332,Kyphosidae_Kyphosus_sectatrix:68.505296):16.157201,(Percichthyidae_Percichthys_trucha:69.999181,((Cirrhitidae_Cirrhitichthys_falco:66.467051,Cheilodactylidae_Cheilodactylus_fasciatus:66.467051):2.53213,(Enoplosidae_Enoplosus_armatus:63.04721,Centrarchidae_Acantharchus_pomotis:63.04721):5.951971):1):14.663316):8.511965):1.725539,(Serranidae_Pseudogramma_polyacantha:82.200001,(Percidae_Perca_fluviatilis:78.577195,((Bovichtidae_Bovichtus_diacanthus:63.021097,(Nototheniidae_Notothenia_coriiceps:9.671082,(Harpagiferidae_Harpagifer_antarcticus:8.390321,(Bathydraconidae_Gymnodraco_acuticeps:7.390321,Channichthyidae_Chionodraco_rastrospinosus:7.390321):1):1.280761):53.350015):12.936917,(((Peristediidae_Peristedion_ecuadorense:54.532565,Triglidae_Prionotus_carolinus:54.532565):11.953546,(Synanceiidae_Synanceia_verrucosa:62.510934,Scorpaenidae_Scorpaenodes_guamensis:62.510934):3.975177):6.621302,(Platycephalidae_Platycephalus_indicus:70.795272,(Anoplopomatidae_Anoplopoma_fimbria:46.504594,((Bathymasteridae_Rathbunella_hypoplecta:22.798552,(Stichaeidae_Cebidichthys_violaceus:15.58542,(Zoarcidae_Zoarces_americanus_viviparus:12.493576,(Anarhichadidae_Anarrhichthys_ocellatus:9.933504,(Zaproridae_Zaprora_silenus:8.933504,Cryptacanthodidae_Cryptacanthodes_maculatus:8.933504):1):2.560072):3.091844):7.213132):19.028758,(((Aulorhynchidae_Aulorhynchus_flavidus:25.280385,Hypoptychidae_Hypoptychus_dybowskii:25.280385):1.019618,((Gasterosteidae_Culaea_inconstans:19.844899,(Gasterosteidae_Apeltes_quadracus:17.136644,Gasterosteidae_Spinachia_spinachia:17.136644):2.708255):1.782129,(Gasterosteidae_Pungitius_pungitius:16.827665,Gasterosteidae_Gasterosteus_aculeatus:16.827665):4.799363):4.672975):13.53479,(Hexagrammidae_Hexagrammos_decagrammus:27.818969,((Cyclopteridae_Cyclopterus_lumpus:20.762394,Liparidae_Liparis_pulchellus:20.762394):4.037609,(Agonidae_Hypsagonus_quadricornis:20.371382,(Cottidae_Cottus_carolinae:18.19359,Psychrolutidae_Psychrolutes_phrictus:18.19359):2.177792):4.428621):3.018966):12.015824):1.992517):4.677284):24.290678):2.312141):2.850601):2.619181):3.622806):12.7):1):10.100001,((Drepaneidae_Drepane_punctata:67.658011,Ephippidae_Chaetodipterus_faber:67.658011):37.341991,(((Lobotidae_Lobotes_pacificus_surinamensis:86.438004,Sciaenidae_Menticirrhus_undulatus_littoralis:86.438004):5.761997,((Monodactylidae_Monodactylus_sebae:70.333247,(Avitoluvarus_eocaenicus:20.64044215,(Zanclidae_Zanclus_cornutus:55.859022,Acanthuridae_Acanthurus_triostegus:55.859022):4.740979):9.733246):15.366754,((Pomacanthidae_Pomacanthus_semicirculatus:54.434118,(Leiognathidae_Leiognathus_equulus:32.700001,Chaetodontidae_Chelmon_rostratus:32.700001):21.734117):17.438962,(Emmelichthyidae_Erythrocles_schlegelii:69.871718,(Malacanthidae_Malacanthus_plumieri:68.303733,(Haemulidae_Haemulon_aurolineatum:64.600001,Lutjanidae_Lutjanus_griseus:64.600001):3.703732):1.567985):2.001362):13.826921):6.5):11.800001,((Sillaginidae_Sillago_sihama:85.853361,(Nemipteridae_Pentapodus_caninus:80.270573,(Lethrinidae_Lethrinus_erythropterus:74.370539,Sparidae_Stenotomus_chrysops:74.370539):5.900034):5.582788):17.146641,(Siganidae_Siganus_spinus:102.000002,((Scatophagidae_Scatophagus_argus:75.860371,Priacanthidae_Heteropriacanthus_cruentatus:75.860371):25.139631,((Caproidae_Antigonia_rubescens:10.905674,Caproidae_Antigonia_capros:10.905674):89.094328,(((Lophiidae_Lophiodes_reticulatus:22.462185,Lophiidae_Lophius_gastrophysus:22.462185):42.032119,((Antennariidae_Histrio_histrio:12.333102,Antennariidae_Antennatus_coccineus:12.333102):33.466899,(Chaunacidae_Chaunax_suttkusi:38.499563,(Gigantactinidae_Gigantactis_sp:32.911618,((Ceratiidae_Cryptopsaras_couesii:20.548708,Ceratiidae_Ceratias_holboelli:20.548708):10.416157,((Himantolophidae_Himantolophus_albinares_sagamius:14.456802,Melanocetidae_Melanocetus_murrayi:14.456802):9.124159,(Oneirodidae_Oneirodes_macrosteus:10.164357,Oneirodidae_Dolopichthys_sp:10.164357):13.416604):7.383904):1.946753):5.587945):7.300438):18.694303):34.505698,(((Prohollardia_avita:37.59388844,(Protacanthodes_nimesensis:1,Triacanthidae_Triacanthus_biaculeatus:46.56624912):17.92672188):10.123893,(Aracanidae_Aracana_aurita:54.800001,(Ostraciidae_Ostracion_cubicus:51.000002,Ostraciidae_Rhinesomus_triqueter:51.000002):3.799999):19.816863):3.183137,(((Diodontidae_Diodon_holocanthus:12.198387,Diodontidae_Chilomycterus_schoepfii:12.198387):43.701614,(Tetraodontidae_Canthigaster_bennetti:24.22571,Tetraodontidae_Tetraodon_miurus:24.22571):31.674291):17.334859,((Molidae_Ranzania_laevis:22,Molidae_Mola_mola:22):46.61027,((Monacanthidae_Stephanolepis_hispidus:28.823604,Monacanthidae_Aluterus_scriptus:28.823604):14.976396,(Balistidae_Xanthichthys_ringens:20.7,(Balistidae_Balistes_vetula:19.7,Balistidae_Sufflamen_fraenatum:19.7):1):23.1):24.81027):4.62459):4.565141):21.200001):1):1):1):1):1):1):1):1):1):3.894248):4.592438):5.113313):5.2):6):12.2):1.46362):8.33638):6.58283):11.71717):9.7):9.5):19):3.553787):17.946213):17.8):23.2):9.5):1):1):37.2):1):1):1,(Fukangichthys_longidorsalis:8.37070273,(Scanilepis_dubia:44.28039863,Evenkia_eunotoptera:1):1):72.14340316):1,Australosomus:74.62162067):1):22.6):1):8.245000714):1):4.097758869):23.21564321):1):1):1):1):3.341598211):1):47);

((Guiyu_oneiros:1,(Onychodus_jandemarrai:31.86025622,((Diplocercides:1,(Latimeriidae_Latimeria_chalumnae:329.2199514,Rhabdoderma:1):35.36566924):47.93000633,((Neoceratodontidae_Neoceratodus_forsteri:279.7982796,Lepidosirenidae_Lepidosiren_paradoxa:279.7982796):128.2017204,((Osteolepis_macrolepidotus:1,Gyroptychius_milleri:6.385910055):1,Eusthenopterus_foordi:19.61822829):8.68749733):4.515627):1):11.85186665):17.63250835,((Cheirolepis_trailli:1,Cheirolepis_schultzei:7.785347184):2.705766956,((Polypteridae_Erpetoichthys_calabaricus:29.200001,Polypteridae_Polypterus_senegalus:29.200001):365.800001,(Osorioichthys_marginis:27.33176285,((Tegeolepis_clarki:17.91095436,Howqualepis_rostridens:1):2.578596918,((Gogosardina_coatesi:5.215114042,(Mimipiscis_bartrami:3.685608109,Mimipiscis_toombsi:1):1):7.246991112,((Moythomasia_lineata:1,Moythomasia_durgaringa:6.002994305):1.793465447,(Stegotrachelus_finlayi:1,(Limnomis_delaneyi:1,(Wendyichthys_dicksoni:28.50222929,(Kentuckia_deani:1,((Mesopoma_planti:1,Mesopoma_carricki:11.85163562):4.48775447,((Birgeria_stensioei:1,(Chondrosteus_acipenseroides:1,(Saurichthys_dawaziensis:1,((Protopsephurus_liui:1,Polyodontidae_Polyodon_spathula:129.8212165):9.078785514,(Acipenseridae_Acipenser_fulvescens:38.579335,(Acipenseridae_Scaphirhynchus_platorynchus:19.375515,Acipenseridae_Scaphirhynchus_albus:19.375515):19.20382):100.320667):23.93587929):38.96354437):50.97541547):97.32515988,(Boreosomus:75.9125777,(((Perleidus_altolepis:83.00638301,(Luganoia_lepidosteoides:94.26361438,(((Macrosemius_fourneti:92.98940076,((Kyphosichthys_grandei:1,Semionotus_elegans:47.06652391):1,Lepisosteidae_Lepisosteus_osseus:248.6197138):1):18.2802872,(Watsonulus_eugnathoides:1,(Amblysemius:1,(Pachyamia_latimaxillaris:1,(Tomognathus_mordax:1,Amiidae_Amia_calva:99.20107809):2.106770322):51.41538206):100.09593):15.08084052):54.6,(Pachycormus:104.6559592,(Crossognathus_danubiensis:172.5988643,(((Anaethalion_angustus:1,Anaethalion_knorri:1.255669244):44.93701173,((Megalopidae_Megalops_atlanticus:133.565966,Elopidae_Elops_saurus:133.565966):62.634035,(Albulidae_Albula_vulpes:150.800001,((Notacanthidae_Notacanthus_chemnitzii:50.669287,(Halosauridae_Halosauropsis_macrochir:40.366211,Halosauridae_Aldrovandia_affinis:40.366211):10.303076):50.330714,(((Eurypharyngidae_Eurypharynx_pelecanoides:25.866364,Saccopharyngidae_Saccopharynx_ampullaceus:25.866364):44.322494,(Nemichthyidae_Nemichthys_scolopaceus:58.739485,Anguillidae_Anguilla_rostrata:58.739485):11.449373):9.198895,(Serrivomeridae_Serrivomer_beanii:76.053487,(Congridae_Conger_oceanicus:65.416147,(Muraenesocidae_Muraenesox_cinereus:57.193624,Ophichthidae_Myrichthys_maculosus:57.193624):8.222523):10.63734):3.334266):21.612248):49.8):45.4):1):86.1,((Lycoptera_davidi:97.35143278,(Paralycoptera_wui:106.653572,(Xixiaichthys_tongxinens:99.04767148,(((Eohiodon_woodruffi:1,Eohiodon_rosei:1.111021197):1,(Hiodon_consteniorum:1,(Hiodontidae_Hiodon_tergisus:9.520291,Hiodontidae_Hiodon_alosoides:9.520291):28.89763719):19.32104244):169.3610304,(Chauliopareion_mahengeense:117.449082,(Pantodontidae_Pantodon_buchholzi:163.100001,((((Brychaetus_muelleri:1,Phareodus_encaustus:6.732575247):26.12950398,Arapaimidae_Arapaima_gigas:79.3):25.549112,Osteoglossidae_Osteoglossum_bicirrhosum:104.849112):43.475578,(Notopteridae_Xenomystus_nigri:120.300002,Gymnarchidae_Gymnarchus_niloticus:120.300002):28.024688):14.775311):1):63):1):1):1):43.7,((((Diplomystus_brevissimus:43.66050005,Sorbinichthys_africanus:1):91.51554723,(Denticipitidae_Denticeps_clupeoides:188.900001,(Chirocentridae_Chirocentrus_dorab:87.096454,((Engraulidae_Coilia_nasus:47.06527,Engraulidae_Engraulis_mordax_eurystole:47.06527):33.347234,((Pristigasteridae_Pellona_flavipinnis:27.450803,Pristigasteridae_Ilisha_elongata:27.450803):46.430359,(Clupeidae_Alosa_pseudoharengus:54.138864,Clupeidae_Dorosoma_cepedianum:54.138864):19.742298):6.531342):6.68395):101.803547):1):40.3,((Alepocephalidae_Talismania_bifurcata:53.266521,((Alepocephalidae_Bathylaco_nigricans:33.802485,Alepocephalidae_Alepocephalus_tenebrosus:33.802485):10.988666,((Alepocephalidae_Rouleina_attrita:22.275085,Alepocephalidae_Xenodermichthys_copei:22.275085):19.924916,Platytroctidae_Sagamichthys_abei:42.200001):2.59115):8.47537):166.430015,((Gonorynchidae_Gonorynchus_abbreviatus:175.900001,(Mahengichthys_singidaensis:102.3183924,Chanidae_Chanos_chanos:147.100001):28.8):22.7,((((Gyrinocheilidae_Gyrinocheilus_sp:70.111694,(Catostomidae_Hypentelium_nigricans:23.594381,(Catostomidae_Carpiodes_carpio:16.355089,Catostomidae_Ictiobus_bubalus:16.355089):7.239292):46.517313):8.688307,Cobitidae_Cobitis_taenia:78.800001):20.5,(Cyprinidae_Danio_rerio:63.300001,(((Cyprinidae_Zacco_sieboldii_platypus:12.727958,Cyprinidae_Opsariichthys_uncirostris_bidens:12.727958):14.610086,((Cyprinidae_Xenocypris_argentea:12.904177,Cyprinidae_Hypophthalmichthys_molitrix:12.904177):2.917503,(Cyprinidae_Luciobrama_macrocephalus:13.439618,(Cyprinidae_Squaliobarbus_curriculus:11.472717,Cyprinidae_Mylopharyngodon_piceus:11.472717):1.966901):2.382062):11.516364):12.401957,(Cyprinidae_Tanakia_lanceolata_himantegus:35.004261,(Cyprinidae_Notemigonus_crysoleucas:22.933404,(Cyprinidae_Semotilus_atromaculatus:21.325215,((Cyprinidae_Campostoma_oligolepis:12.800846,Cyprinidae_Rhinichthys_cataractae:12.800846):4.408707,(Cyprinidae_Phenacobius_uranops:12.822623,(Cyprinidae_Pimephales_promelas_notatus:9.460336,(Cyprinidae_Luxilus_coccogenis:6.738861,Cyprinidae_Notropis_asperifrons:6.738861):2.721475):3.362287):4.38693):4.115662):1.608189):12.070857):4.73574):23.56):36):73.6,((Gymnotidae_Electrophorus_electricus:63.909447,Gymnotidae_Gymnotus_sp:63.909447):83.924067,(((Distichodontidae_Distichodus_maculatus:103.604365,Citharinidae_Citharinus_congicus:103.604365):11.170138,(Alestidae_Alestes_baremoze:106.666979,((Parodontidae_Parodon_nasus:68.800001,Hemiodontidae_Hemiodus_immaculatus:68.800001):22.415197,((Bryconidae_Brycon_pesu:66.003418,(Gasteropelecidae_Thoracocharax_stellatus:27.746828,Gasteropelecidae_Gasteropelecus_sternicla:27.746828):38.25659):1.227196,Characidae_Astyanax_mexicanus:67.230614):23.984584):15.451781):8.107524):22.236489,((Nematogenyidae_Nematogenys_inermis:110.648758,(Trichomycteridae_Trichomycterus_sp:105.886466,((Loricariidae_Loricaria_simillima:70.313425,Astroblepidae_Astroblepus_sp:70.313425):28.122359,(Callichthyidae_Callichthys_callichthys:69.200001,Callichthyidae_Corydoras_trilineatus:69.200001):29.235783):7.450682):4.762292):5.151243,(Diplomystidae_Diplomystes_nahuelbutaensis:106.203801,((Clariidae_Clarias_batrachus:47.60069,Heteropneustidae_Heteropneustes_fossilis:47.60069):38.718856,(((Sisoridae_Bagarius_yarrelli:55.522058,(Amblycipitidae_Liobagrus_aequilabris:48.027606,Akysidae_Akysis_sp:48.027606):7.494452):21.358911,(Schilbeidae_Pseudeutropius_brachypopterus:74.448774,(Bagridae_Mystus_bocourti:36.077943,Bagridae_Bagrus_ubangensis:36.077943):38.370831):2.432195):8.408138,(((Chacidae_Chaca_sp:72.429482,Cetopsidae_Cetopsis_coecutiens:72.429482):10.714797,(Cranoglanididae_Cranoglanis_bouderius:68.700001,Ictaluridae_Ictalurus_punctatus:68.700001):14.444278):1.039619,(Pangasiidae_Pangasianodon_hypophthalmus:82.158061,((Amphiliidae_Amphilius_jacksonii:69.199757,(Malapteruridae_Malapterurus_beninensis:64.740321,Mochokidae_Synodontis_batesii:64.740321):4.459436):10.993535,((Plotosidae_Plotosus_lineatus:73.039645,Siluridae_Hemisilurus_moolenburghi:73.039645):5.503862,(Auchenipteridae_Ageneiosus_atronasus:51.101654,Doradidae_Anduzedoras_oxyrhynchus:51.101654):27.441853):1.649785):1.964769):2.025837):1.105209):1.030439):19.884255):9.5962):21.210991):10.822522):25.066487):25.7):21.096535):10.503465):20.4,(Orthogonikleithrus_francogalliensis:75.78708006,(((Bathylagidae_Bathylagus_euryops:70.542512,(Microstomatidae_Nansenia_longicauda_ardesiaca:51.394,(Opisthoproctidae_Macropinna_microstoma:33.11268,Argentinidae_Argentina_sialis_silus:33.11268):18.28132):19.148512):89.057489,((Galaxiidae_Neochanna_burrowsius:34.505049,Galaxiidae_Galaxias_maculatus:34.505049):111.121934,(((Umbridae_Novumbra_hubbsi:55.762491,Umbridae_Umbra_limi:55.762491):23.63751,(Esocidae_Esox_lucius:31.366195,Esocidae_Esox_americanus:31.366195):48.033806):25.064928,(Salmonidae_Coregonus_clupeaformis:35.300001,(Salmonidae_Thymallus_brevirostris:32.456522,(Salmonidae_Oncorhynchus_nerka_mykiss:19.929533,(Salmonidae_Salvelinus_alpinus:16.737138,Salmonidae_Salmo_salar:16.737138):3.192395):12.526989):2.843479):69.164928):41.162054):13.973018):55.253787,(((Retropinnidae_Retropinna_semoni:73.700001,(((Osmeridae_Osmerus_mordax:10.332899,Osmeridae_Thaleichthys_pacificus:10.332899):5.872101,(Osmeridae_Mallotus_villosus:14.961758,Osmeridae_Hypomesus_pretiosus:14.961758):1.243242):18.133836,(Plecoglossidae_Plecoglossus_altivelis:28.70318,Salangidae_Neosalangichthys_ishikawae:28.70318):5.635656):39.361165):55.7,((Diplophidae_Diplophos_taenia:77.330737,((Gonostomatidae_Bonapartia_pedaliota:54.297445,Gonostomatidae_Margrethia_obtusirostra:54.297445):20.014374,(Gonostomatidae_Gonostoma_elongatum:69.948163,Gonostomatidae_Cyclothone_microdon:69.948163):4.363656):3.018918):6.469264,(Phosichthyidae_Pollichthys_mauli:77.722827,((Sternoptychidae_Argyropelecus_gigas:25.230481,Sternoptychidae_Maurolicus_weitzmani:25.230481):47.329876,(Stomiidae_Chauliodus_macouni_danae:63.126299,(Stomiidae_Stomias_boa:36.381906,(Stomiidae_Chirostomias_pliopterus:31.578039,((Stomiidae_Melanostomias_margaritifer:21.790927,Stomiidae_Leptostomias_longibarba:21.790927):6.240428,((Stomiidae_Photonectes_margarita:19.053467,Stomiidae_Tactostoma_macropus:19.053467):7.977888,(Stomiidae_Malacosteus_niger:25.031355,(Stomiidae_Eustomias_polyaster:24.031355,(Stomiidae_Aristostomias_scintillans:23.031355,Stomiidae_Bathophilus_flemingi_pawneei:23.031355):1):1):2):1):3.546684):4.803867):26.744393):9.434058):5.16247):6.077174):45.6):81.9,((Ateleopodidae_Ateleopus_japonicus:8.089622,Ateleopodidae_Ijimaia_antillarum:8.089622):184.210379,(((Synodontidae_Trachinocephalus_myops:34.928634,Synodontidae_Synodus_foetens:34.928634):78.971367,((Paraulopidae_Paraulopus_oblongus:101.985288,(Synodontidae_Saurida_gracilis:50.257509,Synodontidae_Harpadon_microchir:50.257509):51.727779):8.326796,(((Aulopidae_Aulopus_filamentosus:47.160387,Bathysauridae_Bathysaurus_ferox:47.160387):10.843902,(Ipnopidae_Ipnops_murrayi:13.82,Ipnopidae_Ipnops_agassizi:13.82):44.184289):23.635948,((Ipnopidae_Bathypterois_atricolor:69.328167,(Giganturidae_Gigantura_indica:13.525058,Giganturidae_Gigantura_chuni:13.525058):55.803109):6.462647,(Scopelarchidae_Benthalbella_macropinna:62.490696,(Chlorophthalmidae_Chlorophthalmus_agassizi:61.13635,(Sudidae_Sudis_atrox:55.026295,((Alepisauridae_Anotopterus_pharao:45.661943,(Alepisauridae_Omosudis_lowii:36.284216,Alepisauridae_Alepisaurus_ferox:36.284216):9.377727):8.238175,(Paralepididae_Stemonosudis_intermedia_macrura:50.122687,(Evermannellidae_Evermannella_balbo:46.686813,Paralepididae_Paralepis_coregonoides:46.686813):3.435874):3.777431):1.126177):6.110055):1.354346):13.300118):5.849423):28.671847):3.587917):68.9,(((Neoscopelidae_Neoscopelus_macrolepidotus:42.408365,Neoscopelidae_Scopelengys_tristis:42.408365):31.191636,((Myctophidae_Lampadena_speculigera:39.81673,Myctophidae_Lampanyctus_macdonaldi:39.81673):11.872,(Myctophidae_Benthosema_glaciale:28.30241,Myctophidae_Myctophum_punctatum:28.30241):23.38632):21.911271):99.5,(((Lamprididae_Lampris_guttatus:65.552505,(Regalecidae_Regalecus_russelii:37.682943,Trachipteridae_Trachipterus_trachypterus:37.682943):27.869562):84.747496,(((Mcconichthys_longipinnis:1,(Aphredoderidae_Aphredoderus_sayanus:41.500001,Amblyopsidae_Chologaster_cornuta:41.500001):22.55867407):1,(Lateopisciculus_turrifumosus:1,(Percopsidae_Percopsis_omiscomaycus:14.617149,Percopsidae_Percopsis_transmontana:14.617149):44.9609131):5.480612972):69.93165693,(Zeidae_Zeus_faber:107.100001,(Stylephoridae_Stylephorus_chordatus:78.800001,(Merlucciidae_Merluccius_productus:45.655086,(((Macrouridae_Gadomus_dispar:9.947595,Macrouridae_Bathygadus_favosus:9.947595):28.939269,(Steindachneriidae_Steindachneria_argentea:35.929071,((Macrouridae_Malacocephalus_laevis:17.761718,Macrouridae_Trachonurus_sulcatus:17.761718):10.126413,(Macrouridae_Coelorinchus_caribbaeus:21.883517,Macrouridae_Coryphaenoides_armatus:21.883517):6.004614):8.04094):2.957793):2.613137,((Moridae_Laemonema_goodebeanorum:25.08833,(Moridae_Halargyreus_johnsonii:16.444614,(Moridae_Lepidion_ensiferus:5.35693,Moridae_Antimora_rostrata:5.35693):11.087684):8.643716):13.1716,((Phycidae_Urophycis_tenuis:5.240351,Phycidae_Urophycis_chuss:5.240351):28.607969,(Lotidae_Gaidropsarus_ensis:29.800286,(Lotidae_Lota_lota:23.935071,(Gadidae_Melanogrammus_aeglefinus:5.86839,Gadidae_Gadus_morhua:5.86839):18.066681):5.865215):4.048034):4.41161):3.240071):4.155085):33.144915):28.3):27.890331):15.309669):11.08283,(Polymixiidae_Polymixia_japonica:154.800001,(((Diretmidae_Diretmus_argenteus:53.99855,(Monocentridae_Monocentris_japonica:33.600001,((Trachichthyidae_Hoplostethus_occidentalis_atlanticus:24.361165,Trachichthyidae_Gephyroberyx_darwinii:24.361165):7.919156,(Anoplogastridae_Anoplogaster_cornuta:31.22776,Trachichthyidae_Paratrachichthys_sajademalensis:31.22776):1.052561):1.31968):20.398549):71.275924,((Berycidae_Beryx_decadactylus:70.612394,(Melamphaidae_Poromitra_crassiceps:39.138751,(Melamphaidae_Scopelogadus_beanii:32.603956,Melamphaidae_Melamphaes_suborbitalis:32.603956):6.534795):31.473643):40.295775,(((Rondeletiidae_Rondeletia_bicolor:18.616456,Rondeletiidae_Rondeletia_loricata:18.616456):36.662305,Barbourisiidae_Barbourisia_rufa:55.278761):34.605685,(Cetomimidae_Cetostoma_regani:55.492218,(Cetomimidae_Cetomimus_craneae:10.537863,Cetomimidae_Cetomimus_compuctus:10.537863):44.954355):34.392228):21.023723):14.366305):21.189147,((Holocentridae_Sargocentron_diadema:25.43456,Holocentridae_Holocentrus_rufus:25.43456):119.565441,((Ophidiidae_Brotula_multibarbata:66.227334,(Ophidiidae_Lepophidium_profundorum:23.036156,(Ophidiidae_Genypterus_blacodes:21.590629,Ophidiidae_Ophidion_holbrookii:21.590629):1.445527):43.191178):66.572667,((Batrachoididae_Porichthys_notatus:39.800001,Batrachoididae_Opsanus_tau:39.800001):87,(((Kurtidae_Kurtus_indicus:80.300001,Apogonidae_Apogon_campbelli:80.300001):22,(Eleotridae_Eleotris_acanthopoma_pisonis:64.430477,(Gobiidae_Gobiosoma_bosc:45.630567,Microdesmidae_Microdesmus_longipinnis:45.630567):18.79991):37.869524):19.3,((((Dactylopteridae_Dactylopterus_volitans:68.121568,(Aulostomidae_Aulostomus_maculatus:3.200001,Aulostomidae_Aulostomus_chinensis:3.200001):64.921567):6.178433,((Fistulariidae_Fistularia_petimba:65.463647,Mullidae_Mullus_auratus:65.463647):6.26906,(Callionymidae_Callionymus_sp_bairdi:69.154942,Syngnathidae_Syngnathus_fuscus:69.154942):2.577765):2.567294):20.27,(Chiasmodontidae_Chiasmodon_sp:43.900001,((Centrolophidae_Icichthys_lockingtoni:34.370774,Pomatomidae_Pomatomus_saltatrix:34.370774):6.934458,(((Ariommatidae_Ariomma_bondi:25.644121,Nomeidae_Psenes_cyanophrys:25.644121):5.370208,Scombridae_Auxis_rochei:31.014329):6.588438,((Stromateidae_Peprilus_paru:35.602767,(Gempylidae_Paradiplospinus_gracilis:31.499638,Bramidae_Brama_brama:31.499638):4.103129):1,(Icosteidae_Icosteus_aenigmaticus:33.972252,Trichiuridae_Trichiurus_lepturus:33.972252):2.630515):1):3.702465):2.594769):50.67):21.916687,((((((Synbranchidae_Monopterus_albus:69.146846,Indostomidae_Indostomus_paradoxus:69.146846):4.548479,(Mastacembelidae_Macrognathus_siamensis:18.057555,Mastacembelidae_Mastacembelus_erythrotaenia:18.057555):55.63777):7.004676,((Anabantidae_Ctenopoma_acutirostre_kingsleyae:62.782323,(Helostomatidae_Helostoma_temminkii:59.687826,Osphronemidae_Trichopodus_pectoralis:59.687826):3.094497):7.86905,(Channidae_Channa_striata:67.428514,Nandidae_Nandus_nandus:67.428514):3.222859):10.048628):15.7,((Nematistiidae_Nematistius_pectoralis:60.067614,((Coryphaenidae_Coryphaena_hippurus:19.124214,Rachycentridae_Rachycentron_canadum:19.124214):12.775787,(Echeneidae_Remora_osteochir_australis:10.178122,Echeneidae_Echeneis_naucrates:10.178122):21.721879):28.167613):9.732387,((Sphyraenidae_Sphyraena_sphyraena:65.900885,(Menidae_Mene_maculata:60.339531,Polynemidae_Polydactylus_octonemus:60.339531):5.561354):2.899116,((((Leptobramidae_Leptobrama_muelleri:46.58559,Toxotidae_Toxotes_jaculatrix:46.58559):5.096327,(Xiphiidae_Xiphias_gladius:27.833842,Istiophoridae_Istiophorus_platypterus:27.833842):23.848075):5.298938,Carangidae_Trachinotus_falcatus:56.980855):4.861848,((Eolates_gracilis:1,(Centropomidae_Psammoperca_waigiensis:33.068144,(Centropomidae_Lates_calcarifer:13.595548,Centropomidae_Lates_microlepis:13.595548):19.472596):21.99625597):5.778303033,(Heteronectes_chaneti:6.904531386,(Amphistium_paradoxum:4.939850541,(Psettodidae_Psettodes_erumei:57.842703,(Citharidae_Lepidoblepharon_ophthalmolepis:53.600001,((Cynoglossidae_Cynoglossus_interruptus:42.400002,(Soleidae_Solea_solea:24.944331,Soleidae_Soleichthys_heterorhinos:24.944331):17.455671):9.674607,((Scophthalmidae_Scophthalmus_aquosus:23.305906,Scophthalmidae_Lepidorhombus_boscii:23.305906):26.973285,((Paralichthyidae_Paralichthys_dentatus:40.749719,Bothidae_Bothus_lunatus:40.749719):4.387561,(Paralichthyidae_Pseudorhombus_pentophthalmus:21.404631,(((Pleuronectidae_Glyptocephalus_zachirus:5.096079,Pleuronectidae_Microstomus_pacificus:5.096079):2.538752,(Pleuronectidae_Lyopsetta_exilis:6.085211,Pleuronectidae_Hippoglossus_hippoglossus:6.085211):1.54962):2.215451,(Pleuronectidae_Limanda_limanda:8.798272,((Pleuronectidae_Platichthys_stellatus:3.717184,Pleuronectidae_Pleuronectes_platessa:3.717184):3.081088,(Pleuronectidae_Lepidopsetta_bilineata:4.798272,Pleuronectidae_Psettichthys_melanostictus:4.798272):2):2):1.05201):11.554349):23.732649):5.141911):1.795418):1.525392):4.242702):1):1):1):1):6.957298):1):26.6):12.043384,(((Polycentridae_Polycentrus_schomburgkii:94.214904,(Pholidichthyidae_Pholidichthys_leucotaenia:88.700001,Cichlidae_Cichla_temensis:88.700001):5.514903):1,(((Atherinopsidae_Atherinopsis_californiensis:48.715388,Atherinopsidae_Menidia_menidia:48.715388):22.225577,(Isonidae_Iso_sp:49.317292,(Atherinidae_Atherinomorus_stipes:43.78646,(Melanotaeniidae_Melanotaenia_splendida:39.870052,Pseudomugilidae_Pseudomugil_gertrudae:39.870052):3.916408):5.530832):21.623673):6.46052,(((Poeciliidae_Poecilia_latipinna_reticulata:14.292152,(Poeciliidae_Heterandria_formosa:11.366823,(Poeciliidae_Gambusia_affinis:5.736451,Poeciliidae_Belonesox_belizanus:5.736451):5.630372):2.925329):30.13151,(Cyprinodontidae_Cyprinodon_variegatus:40.469994,Fundulidae_Fundulus_parvipinnis:40.469994):3.953668):31.977823,(Adrianichthyidae_Oryzias_latipes:71.878571,((Zenarchopteridae_Dermogenys_collettei:29.883878,((Belonidae_Tylosurus_crocodilus:6.568056,Belonidae_Ablennes_hians:6.568056):17.429484,(Scomberesocidae_Cololabis_saira:2.646712,Scomberesocidae_Scomberesox_saurus:2.646712):21.350828):5.886338):17.178962,(Hemiramphidae_Hyporhamphus_affinis:36.892311,((Hemiramphidae_Hemiramphus_brasiliensis:13.391766,Hemiramphidae_Oxyporhamphus_micropterus:13.391766):10.259915,((Exocoetidae_Cypselurus_callopterus:6.413654,Exocoetidae_Exocoetus_monocirrhus:6.413654):4.605967,Exocoetidae_Cheilopogon_pinnatibarbatus:11.019621):12.63206):13.24063):10.170529):24.815731):4.522914):1):17.813419):1.999954,((Pseudochromidae_Halidesmus_scapularis:84.850287,Pomacentridae_Pomacentrus_brachialis:84.850287):8.187103,(Embiotocidae_Cymatogaster_aggregata:91.529056,(Mugilidae_Mugil_cephalus:89.757655,(Plesiopidae_Plesiops_coeruleolineatus:86.977996,(Opistognathidae_Opistognathus_maxillosus:79.196597,(((Gobiesocidae_Lepadichthys_lineatus:37.560081,Gobiesocidae_Gobiesox_maeandricus:37.560081):35.985217,Tripterygiidae_Enneanectes_altivelis:73.545298):3.122761,((Clinidae_Clinus_cottoides:50.741024,(Dactyloscopidae_Platygillellus_rubrocinctus:45.641062,Chaenopsidae_Chaenopsis_alepidota:45.641062):5.099962):17.520576,(Blenniidae_Stanulus_seychellensis:31.042358,Blenniidae_Blenniella_cyanostigma:31.042358):37.219242):8.406459):2.528538):7.781399):2.779659):1.771401):1.508334):4.177468):11.228527):3.450865,(Gerreidae_Eucinostomus_argenteus:108.000002,(((Labridae_Lachnolaimus_maximus:59.822938,Odacidae_Haletta_semifasciata:59.822938):16.826114,Scaridae_Scarus_globiceps:76.649052):30.35095,(((Uranoscopidae_Uranoscopus_sulphureus:83.996275,(Ammodytidae_Ammodytes_hexapterus:78.584442,Pinguipedidae_Parapercis_punctulata:78.584442):5.411833):11.903726,(((Acropomatidae_Acropoma_japonicum:90.360051,((Percophidae_Acanthaphritis_unoorum:66.16617,Creediidae_Limnichthys_sp:66.16617):18.796708,(Glaucosomatidae_Glaucosoma_buergeri:66.617288,Pempheridae_Pempheris_schomburgkii:66.617288):18.34559):5.397173):2.814411,(((Oplegnathidae_Oplegnathus_punctatus:61.206964,Kuhliidae_Kuhlia_rupestris:61.206964):7.298332,Kyphosidae_Kyphosus_sectatrix:68.505296):16.157201,(Percichthyidae_Percichthys_trucha:69.999181,((Cirrhitidae_Cirrhitichthys_falco:66.467051,Cheilodactylidae_Cheilodactylus_fasciatus:66.467051):2.53213,(Enoplosidae_Enoplosus_armatus:63.04721,Centrarchidae_Acantharchus_pomotis:63.04721):5.951971):1):14.663316):8.511965):1.725539,(Serranidae_Pseudogramma_polyacantha:82.200001,(Percidae_Perca_fluviatilis:78.577195,((Bovichtidae_Bovichtus_diacanthus:63.021097,(Nototheniidae_Notothenia_coriiceps:9.671082,(Harpagiferidae_Harpagifer_antarcticus:8.390321,(Bathydraconidae_Gymnodraco_acuticeps:7.390321,Channichthyidae_Chionodraco_rastrospinosus:7.390321):1):1.280761):53.350015):12.936917,(((Peristediidae_Peristedion_ecuadorense:54.532565,Triglidae_Prionotus_carolinus:54.532565):11.953546,(Synanceiidae_Synanceia_verrucosa:62.510934,Scorpaenidae_Scorpaenodes_guamensis:62.510934):3.975177):6.621302,(Platycephalidae_Platycephalus_indicus:70.795272,(Anoplopomatidae_Anoplopoma_fimbria:46.504594,((Bathymasteridae_Rathbunella_hypoplecta:22.798552,(Stichaeidae_Cebidichthys_violaceus:15.58542,(Zoarcidae_Zoarces_americanus_viviparus:12.493576,(Anarhichadidae_Anarrhichthys_ocellatus:9.933504,(Zaproridae_Zaprora_silenus:8.933504,Cryptacanthodidae_Cryptacanthodes_maculatus:8.933504):1):2.560072):3.091844):7.213132):19.028758,(((Aulorhynchidae_Aulorhynchus_flavidus:25.280385,Hypoptychidae_Hypoptychus_dybowskii:25.280385):1.019618,((Gasterosteidae_Culaea_inconstans:19.844899,(Gasterosteidae_Apeltes_quadracus:17.136644,Gasterosteidae_Spinachia_spinachia:17.136644):2.708255):1.782129,(Gasterosteidae_Pungitius_pungitius:16.827665,Gasterosteidae_Gasterosteus_aculeatus:16.827665):4.799363):4.672975):13.53479,(Hexagrammidae_Hexagrammos_decagrammus:27.818969,((Cyclopteridae_Cyclopterus_lumpus:20.762394,Liparidae_Liparis_pulchellus:20.762394):4.037609,(Agonidae_Hypsagonus_quadricornis:20.371382,(Cottidae_Cottus_carolinae:18.19359,Psychrolutidae_Psychrolutes_phrictus:18.19359):2.177792):4.428621):3.018966):12.015824):1.992517):4.677284):24.290678):2.312141):2.850601):2.619181):3.622806):12.7):1):10.100001,((Drepaneidae_Drepane_punctata:67.658011,Ephippidae_Chaetodipterus_faber:67.658011):37.341991,(((Lobotidae_Lobotes_pacificus_surinamensis:86.438004,Sciaenidae_Menticirrhus_undulatus_littoralis:86.438004):5.761997,((Monodactylidae_Monodactylus_sebae:70.333247,(Avitoluvarus_eocaenicus:20.05589174,(Zanclidae_Zanclus_cornutus:55.859022,Acanthuridae_Acanthurus_triostegus:55.859022):4.740979):9.733246):15.366754,((Pomacanthidae_Pomacanthus_semicirculatus:54.434118,(Leiognathidae_Leiognathus_equulus:32.700001,Chaetodontidae_Chelmon_rostratus:32.700001):21.734117):17.438962,(Emmelichthyidae_Erythrocles_schlegelii:69.871718,(Malacanthidae_Malacanthus_plumieri:68.303733,(Haemulidae_Haemulon_aurolineatum:64.600001,Lutjanidae_Lutjanus_griseus:64.600001):3.703732):1.567985):2.001362):13.826921):6.5):11.800001,((Sillaginidae_Sillago_sihama:85.853361,(Nemipteridae_Pentapodus_caninus:80.270573,(Lethrinidae_Lethrinus_erythropterus:74.370539,Sparidae_Stenotomus_chrysops:74.370539):5.900034):5.582788):17.146641,(Siganidae_Siganus_spinus:102.000002,((Scatophagidae_Scatophagus_argus:75.860371,Priacanthidae_Heteropriacanthus_cruentatus:75.860371):25.139631,((Caproidae_Antigonia_rubescens:10.905674,Caproidae_Antigonia_capros:10.905674):89.094328,(((Lophiidae_Lophiodes_reticulatus:22.462185,Lophiidae_Lophius_gastrophysus:22.462185):42.032119,((Antennariidae_Histrio_histrio:12.333102,Antennariidae_Antennatus_coccineus:12.333102):33.466899,(Chaunacidae_Chaunax_suttkusi:38.499563,(Gigantactinidae_Gigantactis_sp:32.911618,((Ceratiidae_Cryptopsaras_couesii:20.548708,Ceratiidae_Ceratias_holboelli:20.548708):10.416157,((Himantolophidae_Himantolophus_albinares_sagamius:14.456802,Melanocetidae_Melanocetus_murrayi:14.456802):9.124159,(Oneirodidae_Oneirodes_macrosteus:10.164357,Oneirodidae_Dolopichthys_sp:10.164357):13.416604):7.383904):1.946753):5.587945):7.300438):18.694303):34.505698,(((Prohollardia_avita:37.64118985,(Protacanthodes_nimesensis:1,Triacanthidae_Triacanthus_biaculeatus:46.00753933):18.48543167):10.123893,(Aracanidae_Aracana_aurita:54.800001,(Ostraciidae_Ostracion_cubicus:51.000002,Ostraciidae_Rhinesomus_triqueter:51.000002):3.799999):19.816863):3.183137,(((Diodontidae_Diodon_holocanthus:12.198387,Diodontidae_Chilomycterus_schoepfii:12.198387):43.701614,(Tetraodontidae_Canthigaster_bennetti:24.22571,Tetraodontidae_Tetraodon_miurus:24.22571):31.674291):17.334859,((Molidae_Ranzania_laevis:22,Molidae_Mola_mola:22):46.61027,((Monacanthidae_Stephanolepis_hispidus:27.823604,Monacanthidae_Aluterus_scriptus:27.823604):15.976396,(Balistidae_Xanthichthys_ringens:20.7,(Balistidae_Balistes_vetula:19.7,Balistidae_Sufflamen_fraenatum:19.7):1):23.1):24.81027):4.62459):4.565141):21.200001):1):1):1):1):1):1):1):1):1):3.894248):4.592438):5.113313):5.2):6):12.2):1.46362):8.33638):6.58283):11.71717):9.7):9.5):19):3.553787):17.946213):17.8):23.2):9.5):1):1):37.2):1):1):1,(Fukangichthys_longidorsalis:8.939032108,(Scanilepis_dubia:48.02043347,Evenkia_eunotoptera:1):1):71.71126857):1,Australosomus:74.33250379):1):22.6):1):5.672579031):1):10.33282178):19.24550607):1):1):1):1):3.649094115):1):47);

((Guiyu_oneiros:1,(Onychodus_jandemarrai:37.0316059,((Diplocercides:1,(Latimeriidae_Latimeria_chalumnae:330.4467046,Rhabdoderma:1):37.8702185):44.19870388,((Neoceratodontidae_Neoceratodus_forsteri:279.7982796,Lepidosirenidae_Lepidosiren_paradoxa:279.7982796):129.7450105,((Osteolepis_macrolepidotus:1,Gyroptychius_milleri:15.14122341):1,Eusthenopterus_foordi:34.64742796):1):2.972336906):1):13.28580935):16.19856565,((Cheirolepis_trailli:1,Cheirolepis_schultzei:8.024031491):2.986769703,((Polypteridae_Erpetoichthys_calabaricus:29.200001,Polypteridae_Polypterus_senegalus:29.200001):365.800001,(Osorioichthys_marginis:29.95474769,((Tegeolepis_clarki:22.13791124,Howqualepis_rostridens:1):3.619646991,((Gogosardina_coatesi:3.868069721,(Mimipiscis_bartrami:3.587229005,Mimipiscis_toombsi:1):1):9.017160918,((Moythomasia_lineata:1,Moythomasia_durgaringa:7.915160409):2.008763266,(Stegotrachelus_finlayi:1,(Limnomis_delaneyi:1,(Wendyichthys_dicksoni:29.11236805,(Kentuckia_deani:1,((Mesopoma_planti:1,Mesopoma_carricki:4.653529335):9.763225721,((Birgeria_stensioei:1,(Chondrosteus_acipenseroides:1,(Saurichthys_dawaziensis:1,((Protopsephurus_liui:1,Polyodontidae_Polyodon_spathula:129.5754765):9.324525468,(Acipenseridae_Acipenser_fulvescens:38.579335,(Acipenseridae_Scaphirhynchus_platorynchus:19.375515,Acipenseridae_Scaphirhynchus_albus:19.375515):19.20382):100.320667):23.60156613):38.81502415):51.119701):97.66370772,(Boreosomus:75.14088771,((Australosomus:3.003527544,(Fukangichthys_longidorsalis:10.32803339,(Scanilepis_dubia:48.5737121,Evenkia_eunotoptera:1):1):1):70.88920194,(Perleidus_altolepis:83.47042568,(Luganoia_lepidosteoides:90.94095002,(((Macrosemius_fourneti:92.68373864,((Lepisosteidae_Lepisosteus_osseus:201.2821662,Semionotus_elegans:1):44.55053186,Kyphosichthys_grandei:1):1):21.06730293,(Watsonulus_eugnathoides:1,(Amblysemius:1,(Pachyamia_latimaxillaris:1.194339815,(Tomognathus_mordax:1,Amiidae_Amia_calva:97.4991491):1):54.05925484):100.0952244):15.24637266):54.6,(Pachycormus:106.1286104,(Crossognathus_danubiensis:179.8081027,(((Anaethalion_angustus:1.031057944,Anaethalion_knorri:1):44.86850945,((Megalopidae_Megalops_atlanticus:133.565966,Elopidae_Elops_saurus:133.565966):62.634035,(Albulidae_Albula_vulpes:150.800001,((Notacanthidae_Notacanthus_chemnitzii:50.669287,(Halosauridae_Halosauropsis_macrochir:40.366211,Halosauridae_Aldrovandia_affinis:40.366211):10.303076):50.330714,(((Eurypharyngidae_Eurypharynx_pelecanoides:25.866364,Saccopharyngidae_Saccopharynx_ampullaceus:25.866364):44.322494,(Nemichthyidae_Nemichthys_scolopaceus:58.739485,Anguillidae_Anguilla_rostrata:58.739485):11.449373):9.198895,(Serrivomeridae_Serrivomer_beanii:76.053487,(Congridae_Conger_oceanicus:65.416147,(Muraenesocidae_Muraenesox_cinereus:57.193624,Ophichthidae_Myrichthys_maculosus:57.193624):8.222523):10.63734):3.334266):21.612248):49.8):45.4):1):86.1,((Lycoptera_davidi:98.85027409,(Paralycoptera_wui:107.6475596,(Xixiaichthys_tongxinens:100.5760139,(((Eohiodon_woodruffi:6.298455197,Eohiodon_rosei:1):1,(Hiodon_consteniorum:1,(Hiodontidae_Hiodon_tergisus:9.520291,Hiodontidae_Hiodon_alosoides:9.520291):27.78550754):20.17633321):169.6178693,(Chauliopareion_mahengeense:116.5428642,(Pantodontidae_Pantodon_buchholzi:163.100001,(((Osteoglossidae_Osteoglossum_bicirrhosum:79.3,Arapaimidae_Arapaima_gigas:79.3):25.549112,(Brychaetus_muelleri:1,Phareodus_encaustus:9.737488492):48.58580305):43.475578,(Notopteridae_Xenomystus_nigri:120.300002,Gymnarchidae_Gymnarchus_niloticus:120.300002):28.024688):14.775311):1):63):1):1):1):43.7,((((Diplomystus_brevissimus:43.46426384,Sorbinichthys_africanus:1):91.83534251,(Denticipitidae_Denticeps_clupeoides:188.900001,(Chirocentridae_Chirocentrus_dorab:87.096454,((Engraulidae_Coilia_nasus:47.06527,Engraulidae_Engraulis_mordax_eurystole:47.06527):33.347234,((Pristigasteridae_Pellona_flavipinnis:27.450803,Pristigasteridae_Ilisha_elongata:27.450803):46.430359,(Clupeidae_Alosa_pseudoharengus:54.138864,Clupeidae_Dorosoma_cepedianum:54.138864):19.742298):6.531342):6.68395):101.803547):1):40.3,((Alepocephalidae_Talismania_bifurcata:53.266521,((Alepocephalidae_Bathylaco_nigricans:33.802485,Alepocephalidae_Alepocephalus_tenebrosus:33.802485):10.988666,((Alepocephalidae_Rouleina_attrita:22.275085,Alepocephalidae_Xenodermichthys_copei:22.275085):19.924916,Platytroctidae_Sagamichthys_abei:42.200001):2.59115):8.47537):166.430015,((Gonorynchidae_Gonorynchus_abbreviatus:175.900001,(Mahengichthys_singidaensis:100.3131525,Chanidae_Chanos_chanos:147.100001):28.8):22.7,((((Gyrinocheilidae_Gyrinocheilus_sp:70.111694,(Catostomidae_Hypentelium_nigricans:23.594381,(Catostomidae_Carpiodes_carpio:16.355089,Catostomidae_Ictiobus_bubalus:16.355089):7.239292):46.517313):8.688307,Cobitidae_Cobitis_taenia:78.800001):20.5,(Cyprinidae_Danio_rerio:63.300001,(((Cyprinidae_Zacco_sieboldii_platypus:12.727958,Cyprinidae_Opsariichthys_uncirostris_bidens:12.727958):14.610086,((Cyprinidae_Xenocypris_argentea:12.904177,Cyprinidae_Hypophthalmichthys_molitrix:12.904177):2.917503,(Cyprinidae_Luciobrama_macrocephalus:13.439618,(Cyprinidae_Squaliobarbus_curriculus:11.472717,Cyprinidae_Mylopharyngodon_piceus:11.472717):1.966901):2.382062):11.516364):12.401957,(Cyprinidae_Tanakia_lanceolata_himantegus:35.004261,(Cyprinidae_Notemigonus_crysoleucas:22.933404,(Cyprinidae_Semotilus_atromaculatus:21.325215,((Cyprinidae_Campostoma_oligolepis:12.800846,Cyprinidae_Rhinichthys_cataractae:12.800846):4.408707,(Cyprinidae_Phenacobius_uranops:12.822623,(Cyprinidae_Pimephales_promelas_notatus:9.460336,(Cyprinidae_Luxilus_coccogenis:6.738861,Cyprinidae_Notropis_asperifrons:6.738861):2.721475):3.362287):4.38693):4.115662):1.608189):12.070857):4.73574):23.56):36):73.6,((Gymnotidae_Electrophorus_electricus:63.909447,Gymnotidae_Gymnotus_sp:63.909447):83.924067,(((Distichodontidae_Distichodus_maculatus:103.604365,Citharinidae_Citharinus_congicus:103.604365):11.170138,(Alestidae_Alestes_baremoze:106.666979,((Parodontidae_Parodon_nasus:68.800001,Hemiodontidae_Hemiodus_immaculatus:68.800001):22.415197,((Bryconidae_Brycon_pesu:66.003418,(Gasteropelecidae_Thoracocharax_stellatus:27.746828,Gasteropelecidae_Gasteropelecus_sternicla:27.746828):38.25659):1.227196,Characidae_Astyanax_mexicanus:67.230614):23.984584):15.451781):8.107524):22.236489,((Nematogenyidae_Nematogenys_inermis:110.648758,(Trichomycteridae_Trichomycterus_sp:105.886466,((Loricariidae_Loricaria_simillima:70.313425,Astroblepidae_Astroblepus_sp:70.313425):28.122359,(Callichthyidae_Callichthys_callichthys:69.200001,Callichthyidae_Corydoras_trilineatus:69.200001):29.235783):7.450682):4.762292):5.151243,(Diplomystidae_Diplomystes_nahuelbutaensis:106.203801,((Clariidae_Clarias_batrachus:47.60069,Heteropneustidae_Heteropneustes_fossilis:47.60069):38.718856,(((Sisoridae_Bagarius_yarrelli:55.522058,(Amblycipitidae_Liobagrus_aequilabris:48.027606,Akysidae_Akysis_sp:48.027606):7.494452):21.358911,(Schilbeidae_Pseudeutropius_brachypopterus:74.448774,(Bagridae_Mystus_bocourti:36.077943,Bagridae_Bagrus_ubangensis:36.077943):38.370831):2.432195):8.408138,(((Chacidae_Chaca_sp:72.429482,Cetopsidae_Cetopsis_coecutiens:72.429482):10.714797,(Cranoglanididae_Cranoglanis_bouderius:68.700001,Ictaluridae_Ictalurus_punctatus:68.700001):14.444278):1.039619,(Pangasiidae_Pangasianodon_hypophthalmus:82.158061,((Amphiliidae_Amphilius_jacksonii:69.199757,(Malapteruridae_Malapterurus_beninensis:64.740321,Mochokidae_Synodontis_batesii:64.740321):4.459436):10.993535,((Plotosidae_Plotosus_lineatus:73.039645,Siluridae_Hemisilurus_moolenburghi:73.039645):5.503862,(Auchenipteridae_Ageneiosus_atronasus:51.101654,Doradidae_Anduzedoras_oxyrhynchus:51.101654):27.441853):1.649785):1.964769):2.025837):1.105209):1.030439):19.884255):9.5962):21.210991):10.822522):25.066487):25.7):21.096535):10.503465):20.4,(Orthogonikleithrus_francogalliensis:77.72537639,(((Bathylagidae_Bathylagus_euryops:70.542512,(Microstomatidae_Nansenia_longicauda_ardesiaca:51.394,(Opisthoproctidae_Macropinna_microstoma:33.11268,Argentinidae_Argentina_sialis_silus:33.11268):18.28132):19.148512):89.057489,((Galaxiidae_Neochanna_burrowsius:34.505049,Galaxiidae_Galaxias_maculatus:34.505049):111.121934,(((Umbridae_Novumbra_hubbsi:55.762491,Umbridae_Umbra_limi:55.762491):23.63751,(Esocidae_Esox_lucius:31.366195,Esocidae_Esox_americanus:31.366195):48.033806):25.064928,(Salmonidae_Coregonus_clupeaformis:35.300001,(Salmonidae_Thymallus_brevirostris:32.456522,(Salmonidae_Oncorhynchus_nerka_mykiss:19.929533,(Salmonidae_Salvelinus_alpinus:16.737138,Salmonidae_Salmo_salar:16.737138):3.192395):12.526989):2.843479):69.164928):41.162054):13.973018):55.253787,(((Retropinnidae_Retropinna_semoni:73.700001,(((Osmeridae_Osmerus_mordax:10.332899,Osmeridae_Thaleichthys_pacificus:10.332899):5.872101,(Osmeridae_Mallotus_villosus:14.961758,Osmeridae_Hypomesus_pretiosus:14.961758):1.243242):18.133836,(Plecoglossidae_Plecoglossus_altivelis:28.70318,Salangidae_Neosalangichthys_ishikawae:28.70318):5.635656):39.361165):55.7,((Diplophidae_Diplophos_taenia:77.330737,((Gonostomatidae_Bonapartia_pedaliota:54.297445,Gonostomatidae_Margrethia_obtusirostra:54.297445):20.014374,(Gonostomatidae_Gonostoma_elongatum:69.948163,Gonostomatidae_Cyclothone_microdon:69.948163):4.363656):3.018918):6.469264,(Phosichthyidae_Pollichthys_mauli:77.722827,((Sternoptychidae_Argyropelecus_gigas:25.230481,Sternoptychidae_Maurolicus_weitzmani:25.230481):47.329876,(Stomiidae_Chauliodus_macouni_danae:63.126299,(Stomiidae_Stomias_boa:36.381906,(Stomiidae_Chirostomias_pliopterus:31.578039,((Stomiidae_Melanostomias_margaritifer:21.790927,Stomiidae_Leptostomias_longibarba:21.790927):6.240428,((Stomiidae_Photonectes_margarita:19.053467,Stomiidae_Tactostoma_macropus:19.053467):7.977888,(Stomiidae_Malacosteus_niger:25.031355,(Stomiidae_Eustomias_polyaster:24.031355,(Stomiidae_Aristostomias_scintillans:23.031355,Stomiidae_Bathophilus_flemingi_pawneei:23.031355):1):1):2):1):3.546684):4.803867):26.744393):9.434058):5.16247):6.077174):45.6):81.9,((Ateleopodidae_Ateleopus_japonicus:8.089622,Ateleopodidae_Ijimaia_antillarum:8.089622):184.210379,(((Synodontidae_Trachinocephalus_myops:34.928634,Synodontidae_Synodus_foetens:34.928634):78.971367,((Paraulopidae_Paraulopus_oblongus:101.985288,(Synodontidae_Saurida_gracilis:50.257509,Synodontidae_Harpadon_microchir:50.257509):51.727779):8.326796,(((Aulopidae_Aulopus_filamentosus:47.160387,Bathysauridae_Bathysaurus_ferox:47.160387):10.843902,(Ipnopidae_Ipnops_murrayi:13.82,Ipnopidae_Ipnops_agassizi:13.82):44.184289):23.635948,((Ipnopidae_Bathypterois_atricolor:69.328167,(Giganturidae_Gigantura_indica:13.525058,Giganturidae_Gigantura_chuni:13.525058):55.803109):6.462647,(Scopelarchidae_Benthalbella_macropinna:62.490696,(Chlorophthalmidae_Chlorophthalmus_agassizi:61.13635,(Sudidae_Sudis_atrox:55.026295,((Alepisauridae_Anotopterus_pharao:45.661943,(Alepisauridae_Omosudis_lowii:36.284216,Alepisauridae_Alepisaurus_ferox:36.284216):9.377727):8.238175,(Paralepididae_Stemonosudis_intermedia_macrura:50.122687,(Evermannellidae_Evermannella_balbo:46.686813,Paralepididae_Paralepis_coregonoides:46.686813):3.435874):3.777431):1.126177):6.110055):1.354346):13.300118):5.849423):28.671847):3.587917):68.9,(((Neoscopelidae_Neoscopelus_macrolepidotus:42.408365,Neoscopelidae_Scopelengys_tristis:42.408365):31.191636,((Myctophidae_Lampadena_speculigera:39.81673,Myctophidae_Lampanyctus_macdonaldi:39.81673):11.872,(Myctophidae_Benthosema_glaciale:28.30241,Myctophidae_Myctophum_punctatum:28.30241):23.38632):21.911271):99.5,(((Lamprididae_Lampris_guttatus:65.552505,(Regalecidae_Regalecus_russelii:37.682943,Trachipteridae_Trachipterus_trachypterus:37.682943):27.869562):84.747496,(((Mcconichthys_longipinnis:1,(Aphredoderidae_Aphredoderus_sayanus:41.500001,Amblyopsidae_Chologaster_cornuta:41.500001):22.56615902):1,(Lateopisciculus_turrifumosus:1,(Percopsidae_Percopsis_omiscomaycus:14.617149,Percopsidae_Percopsis_transmontana:14.617149):43.18696924):7.262041783):69.92417198,(Zeidae_Zeus_faber:107.100001,(Stylephoridae_Stylephorus_chordatus:78.800001,(Merlucciidae_Merluccius_productus:45.655086,(((Macrouridae_Gadomus_dispar:9.947595,Macrouridae_Bathygadus_favosus:9.947595):28.939269,(Steindachneriidae_Steindachneria_argentea:35.929071,((Macrouridae_Malacocephalus_laevis:17.761718,Macrouridae_Trachonurus_sulcatus:17.761718):10.126413,(Macrouridae_Coelorinchus_caribbaeus:21.883517,Macrouridae_Coryphaenoides_armatus:21.883517):6.004614):8.04094):2.957793):2.613137,((Moridae_Laemonema_goodebeanorum:25.08833,(Moridae_Halargyreus_johnsonii:16.444614,(Moridae_Lepidion_ensiferus:5.35693,Moridae_Antimora_rostrata:5.35693):11.087684):8.643716):13.1716,((Phycidae_Urophycis_tenuis:5.240351,Phycidae_Urophycis_chuss:5.240351):28.607969,(Lotidae_Gaidropsarus_ensis:29.800286,(Lotidae_Lota_lota:23.935071,(Gadidae_Melanogrammus_aeglefinus:5.86839,Gadidae_Gadus_morhua:5.86839):18.066681):5.865215):4.048034):4.41161):3.240071):4.155085):33.144915):28.3):27.890331):15.309669):11.08283,(Polymixiidae_Polymixia_japonica:154.800001,(((Diretmidae_Diretmus_argenteus:53.99855,(Monocentridae_Monocentris_japonica:33.600001,((Trachichthyidae_Hoplostethus_occidentalis_atlanticus:24.361165,Trachichthyidae_Gephyroberyx_darwinii:24.361165):7.919156,(Anoplogastridae_Anoplogaster_cornuta:31.22776,Trachichthyidae_Paratrachichthys_sajademalensis:31.22776):1.052561):1.31968):20.398549):71.275924,((Berycidae_Beryx_decadactylus:70.612394,(Melamphaidae_Poromitra_crassiceps:39.138751,(Melamphaidae_Scopelogadus_beanii:32.603956,Melamphaidae_Melamphaes_suborbitalis:32.603956):6.534795):31.473643):40.295775,(((Rondeletiidae_Rondeletia_bicolor:18.616456,Rondeletiidae_Rondeletia_loricata:18.616456):36.662305,Barbourisiidae_Barbourisia_rufa:55.278761):34.605685,(Cetomimidae_Cetostoma_regani:55.492218,(Cetomimidae_Cetomimus_craneae:10.537863,Cetomimidae_Cetomimus_compuctus:10.537863):44.954355):34.392228):21.023723):14.366305):21.189147,((Holocentridae_Sargocentron_diadema:25.43456,Holocentridae_Holocentrus_rufus:25.43456):119.565441,((Ophidiidae_Brotula_multibarbata:66.227334,(Ophidiidae_Lepophidium_profundorum:23.036156,(Ophidiidae_Genypterus_blacodes:21.590629,Ophidiidae_Ophidion_holbrookii:21.590629):1.445527):43.191178):66.572667,((Batrachoididae_Porichthys_notatus:39.800001,Batrachoididae_Opsanus_tau:39.800001):87,(((Kurtidae_Kurtus_indicus:80.300001,Apogonidae_Apogon_campbelli:80.300001):22,(Eleotridae_Eleotris_acanthopoma_pisonis:64.430477,(Gobiidae_Gobiosoma_bosc:45.630567,Microdesmidae_Microdesmus_longipinnis:45.630567):18.79991):37.869524):19.3,((((Dactylopteridae_Dactylopterus_volitans:68.121568,(Aulostomidae_Aulostomus_maculatus:3.200001,Aulostomidae_Aulostomus_chinensis:3.200001):64.921567):6.178433,((Fistulariidae_Fistularia_petimba:65.463647,Mullidae_Mullus_auratus:65.463647):6.26906,(Callionymidae_Callionymus_sp_bairdi:69.154942,Syngnathidae_Syngnathus_fuscus:69.154942):2.577765):2.567294):20.27,(Chiasmodontidae_Chiasmodon_sp:43.900001,((Centrolophidae_Icichthys_lockingtoni:34.370774,Pomatomidae_Pomatomus_saltatrix:34.370774):6.934458,(((Ariommatidae_Ariomma_bondi:25.644121,Nomeidae_Psenes_cyanophrys:25.644121):5.370208,Scombridae_Auxis_rochei:31.014329):6.588438,((Stromateidae_Peprilus_paru:35.602767,(Gempylidae_Paradiplospinus_gracilis:31.499638,Bramidae_Brama_brama:31.499638):4.103129):1,(Icosteidae_Icosteus_aenigmaticus:33.972252,Trichiuridae_Trichiurus_lepturus:33.972252):2.630515):1):3.702465):2.594769):50.67):21.916687,((((((Synbranchidae_Monopterus_albus:69.146846,Indostomidae_Indostomus_paradoxus:69.146846):4.548479,(Mastacembelidae_Macrognathus_siamensis:18.057555,Mastacembelidae_Mastacembelus_erythrotaenia:18.057555):55.63777):7.004676,((Anabantidae_Ctenopoma_acutirostre_kingsleyae:62.782323,(Helostomatidae_Helostoma_temminkii:59.687826,Osphronemidae_Trichopodus_pectoralis:59.687826):3.094497):7.86905,(Channidae_Channa_striata:67.428514,Nandidae_Nandus_nandus:67.428514):3.222859):10.048628):15.7,((Nematistiidae_Nematistius_pectoralis:60.067614,((Coryphaenidae_Coryphaena_hippurus:19.124214,Rachycentridae_Rachycentron_canadum:19.124214):12.775787,(Echeneidae_Remora_osteochir_australis:10.178122,Echeneidae_Echeneis_naucrates:10.178122):21.721879):28.167613):9.732387,((Sphyraenidae_Sphyraena_sphyraena:65.900885,(Menidae_Mene_maculata:60.339531,Polynemidae_Polydactylus_octonemus:60.339531):5.561354):2.899116,((((Leptobramidae_Leptobrama_muelleri:46.58559,Toxotidae_Toxotes_jaculatrix:46.58559):5.096327,(Xiphiidae_Xiphias_gladius:27.833842,Istiophoridae_Istiophorus_platypterus:27.833842):23.848075):5.298938,Carangidae_Trachinotus_falcatus:56.980855):4.861848,((Eolates_gracilis:1,(Centropomidae_Psammoperca_waigiensis:33.068144,(Centropomidae_Lates_calcarifer:13.595548,Centropomidae_Lates_microlepis:13.595548):19.472596):19.1366804):8.637878596,(Heteronectes_chaneti:8.14623573,(Amphistium_paradoxum:3.008806731,(Psettodidae_Psettodes_erumei:57.842703,(Citharidae_Lepidoblepharon_ophthalmolepis:53.600001,((Cynoglossidae_Cynoglossus_interruptus:42.400002,(Soleidae_Solea_solea:24.944331,Soleidae_Soleichthys_heterorhinos:24.944331):17.455671):9.674607,((Scophthalmidae_Scophthalmus_aquosus:23.305906,Scophthalmidae_Lepidorhombus_boscii:23.305906):26.973285,((Paralichthyidae_Paralichthys_dentatus:40.749719,Bothidae_Bothus_lunatus:40.749719):4.387561,(Paralichthyidae_Pseudorhombus_pentophthalmus:21.404631,(((Pleuronectidae_Glyptocephalus_zachirus:5.096079,Pleuronectidae_Microstomus_pacificus:5.096079):2.538752,(Pleuronectidae_Lyopsetta_exilis:6.085211,Pleuronectidae_Hippoglossus_hippoglossus:6.085211):1.54962):2.215451,(Pleuronectidae_Limanda_limanda:8.798272,((Pleuronectidae_Platichthys_stellatus:3.717184,Pleuronectidae_Pleuronectes_platessa:3.717184):3.081088,(Pleuronectidae_Lepidopsetta_bilineata:4.798272,Pleuronectidae_Psettichthys_melanostictus:4.798272):2):2):1.05201):11.554349):23.732649):5.141911):1.795418):1.525392):4.242702):1):1):1):1):6.957298):1):26.6):12.043384,(((Polycentridae_Polycentrus_schomburgkii:94.214904,(Pholidichthyidae_Pholidichthys_leucotaenia:88.700001,Cichlidae_Cichla_temensis:88.700001):5.514903):1,(((Atherinopsidae_Atherinopsis_californiensis:48.715388,Atherinopsidae_Menidia_menidia:48.715388):22.225577,(Isonidae_Iso_sp:49.317292,(Atherinidae_Atherinomorus_stipes:43.78646,(Melanotaeniidae_Melanotaenia_splendida:39.870052,Pseudomugilidae_Pseudomugil_gertrudae:39.870052):3.916408):5.530832):21.623673):6.46052,(((Poeciliidae_Poecilia_latipinna_reticulata:14.292152,(Poeciliidae_Heterandria_formosa:11.366823,(Poeciliidae_Gambusia_affinis:5.736451,Poeciliidae_Belonesox_belizanus:5.736451):5.630372):2.925329):30.13151,(Cyprinodontidae_Cyprinodon_variegatus:40.469994,Fundulidae_Fundulus_parvipinnis:40.469994):3.953668):31.977823,(Adrianichthyidae_Oryzias_latipes:71.878571,((Zenarchopteridae_Dermogenys_collettei:29.883878,((Belonidae_Tylosurus_crocodilus:6.568056,Belonidae_Ablennes_hians:6.568056):17.429484,(Scomberesocidae_Cololabis_saira:2.646712,Scomberesocidae_Scomberesox_saurus:2.646712):21.350828):5.886338):17.178962,(Hemiramphidae_Hyporhamphus_affinis:36.892311,((Hemiramphidae_Hemiramphus_brasiliensis:13.391766,Hemiramphidae_Oxyporhamphus_micropterus:13.391766):10.259915,((Exocoetidae_Cypselurus_callopterus:6.413654,Exocoetidae_Exocoetus_monocirrhus:6.413654):4.605967,Exocoetidae_Cheilopogon_pinnatibarbatus:11.019621):12.63206):13.24063):10.170529):24.815731):4.522914):1):17.813419):1.999954,((Pseudochromidae_Halidesmus_scapularis:84.850287,Pomacentridae_Pomacentrus_brachialis:84.850287):8.187103,(Embiotocidae_Cymatogaster_aggregata:91.529056,(Mugilidae_Mugil_cephalus:89.757655,(Plesiopidae_Plesiops_coeruleolineatus:86.977996,(Opistognathidae_Opistognathus_maxillosus:79.196597,(((Gobiesocidae_Lepadichthys_lineatus:37.560081,Gobiesocidae_Gobiesox_maeandricus:37.560081):35.985217,Tripterygiidae_Enneanectes_altivelis:73.545298):3.122761,((Clinidae_Clinus_cottoides:50.741024,(Dactyloscopidae_Platygillellus_rubrocinctus:45.641062,Chaenopsidae_Chaenopsis_alepidota:45.641062):5.099962):17.520576,(Blenniidae_Stanulus_seychellensis:31.042358,Blenniidae_Blenniella_cyanostigma:31.042358):37.219242):8.406459):2.528538):7.781399):2.779659):1.771401):1.508334):4.177468):11.228527):3.450865,(Gerreidae_Eucinostomus_argenteus:108.000002,(((Labridae_Lachnolaimus_maximus:59.822938,Odacidae_Haletta_semifasciata:59.822938):16.826114,Scaridae_Scarus_globiceps:76.649052):30.35095,(((Uranoscopidae_Uranoscopus_sulphureus:83.996275,(Ammodytidae_Ammodytes_hexapterus:78.584442,Pinguipedidae_Parapercis_punctulata:78.584442):5.411833):11.903726,(((Acropomatidae_Acropoma_japonicum:90.360051,((Percophidae_Acanthaphritis_unoorum:66.16617,Creediidae_Limnichthys_sp:66.16617):18.796708,(Glaucosomatidae_Glaucosoma_buergeri:66.617288,Pempheridae_Pempheris_schomburgkii:66.617288):18.34559):5.397173):2.814411,(((Oplegnathidae_Oplegnathus_punctatus:61.206964,Kuhliidae_Kuhlia_rupestris:61.206964):7.298332,Kyphosidae_Kyphosus_sectatrix:68.505296):16.157201,(Percichthyidae_Percichthys_trucha:69.999181,((Cirrhitidae_Cirrhitichthys_falco:66.467051,Cheilodactylidae_Cheilodactylus_fasciatus:66.467051):2.53213,(Enoplosidae_Enoplosus_armatus:63.04721,Centrarchidae_Acantharchus_pomotis:63.04721):5.951971):1):14.663316):8.511965):1.725539,(Serranidae_Pseudogramma_polyacantha:82.200001,(Percidae_Perca_fluviatilis:78.577195,((Bovichtidae_Bovichtus_diacanthus:63.021097,(Nototheniidae_Notothenia_coriiceps:9.671082,(Harpagiferidae_Harpagifer_antarcticus:8.390321,(Bathydraconidae_Gymnodraco_acuticeps:7.390321,Channichthyidae_Chionodraco_rastrospinosus:7.390321):1):1.280761):53.350015):12.936917,(((Peristediidae_Peristedion_ecuadorense:54.532565,Triglidae_Prionotus_carolinus:54.532565):11.953546,(Synanceiidae_Synanceia_verrucosa:62.510934,Scorpaenidae_Scorpaenodes_guamensis:62.510934):3.975177):6.621302,(Platycephalidae_Platycephalus_indicus:70.795272,(Anoplopomatidae_Anoplopoma_fimbria:46.504594,((Bathymasteridae_Rathbunella_hypoplecta:22.798552,(Stichaeidae_Cebidichthys_violaceus:15.58542,(Zoarcidae_Zoarces_americanus_viviparus:12.493576,(Anarhichadidae_Anarrhichthys_ocellatus:9.933504,(Zaproridae_Zaprora_silenus:8.933504,Cryptacanthodidae_Cryptacanthodes_maculatus:8.933504):1):2.560072):3.091844):7.213132):19.028758,(((Aulorhynchidae_Aulorhynchus_flavidus:25.280385,Hypoptychidae_Hypoptychus_dybowskii:25.280385):1.019618,((Gasterosteidae_Culaea_inconstans:19.844899,(Gasterosteidae_Apeltes_quadracus:17.136644,Gasterosteidae_Spinachia_spinachia:17.136644):2.708255):1.782129,(Gasterosteidae_Pungitius_pungitius:16.827665,Gasterosteidae_Gasterosteus_aculeatus:16.827665):4.799363):4.672975):13.53479,(Hexagrammidae_Hexagrammos_decagrammus:27.818969,((Cyclopteridae_Cyclopterus_lumpus:20.762394,Liparidae_Liparis_pulchellus:20.762394):4.037609,(Agonidae_Hypsagonus_quadricornis:20.371382,(Cottidae_Cottus_carolinae:18.19359,Psychrolutidae_Psychrolutes_phrictus:18.19359):2.177792):4.428621):3.018966):12.015824):1.992517):4.677284):24.290678):2.312141):2.850601):2.619181):3.622806):12.7):1):10.100001,((Drepaneidae_Drepane_punctata:67.658011,Ephippidae_Chaetodipterus_faber:67.658011):37.341991,(((Lobotidae_Lobotes_pacificus_surinamensis:86.438004,Sciaenidae_Menticirrhus_undulatus_littoralis:86.438004):5.761997,((Monodactylidae_Monodactylus_sebae:70.333247,(Avitoluvarus_eocaenicus:19.83632885,(Zanclidae_Zanclus_cornutus:55.859022,Acanthuridae_Acanthurus_triostegus:55.859022):4.740979):9.733246):15.366754,((Pomacanthidae_Pomacanthus_semicirculatus:54.434118,(Leiognathidae_Leiognathus_equulus:32.700001,Chaetodontidae_Chelmon_rostratus:32.700001):21.734117):17.438962,(Emmelichthyidae_Erythrocles_schlegelii:69.871718,(Malacanthidae_Malacanthus_plumieri:68.303733,(Haemulidae_Haemulon_aurolineatum:64.600001,Lutjanidae_Lutjanus_griseus:64.600001):3.703732):1.567985):2.001362):13.826921):6.5):11.800001,((Sillaginidae_Sillago_sihama:85.853361,(Nemipteridae_Pentapodus_caninus:80.270573,(Lethrinidae_Lethrinus_erythropterus:74.370539,Sparidae_Stenotomus_chrysops:74.370539):5.900034):5.582788):17.146641,(Siganidae_Siganus_spinus:102.000002,((Scatophagidae_Scatophagus_argus:75.860371,Priacanthidae_Heteropriacanthus_cruentatus:75.860371):25.139631,((Caproidae_Antigonia_rubescens:10.905674,Caproidae_Antigonia_capros:10.905674):89.094328,(((Lophiidae_Lophiodes_reticulatus:22.462185,Lophiidae_Lophius_gastrophysus:22.462185):42.032119,((Antennariidae_Histrio_histrio:12.333102,Antennariidae_Antennatus_coccineus:12.333102):33.466899,(Chaunacidae_Chaunax_suttkusi:38.499563,(Gigantactinidae_Gigantactis_sp:32.911618,((Ceratiidae_Cryptopsaras_couesii:20.548708,Ceratiidae_Ceratias_holboelli:20.548708):10.416157,((Himantolophidae_Himantolophus_albinares_sagamius:14.456802,Melanocetidae_Melanocetus_murrayi:14.456802):9.124159,(Oneirodidae_Oneirodes_macrosteus:10.164357,Oneirodidae_Dolopichthys_sp:10.164357):13.416604):7.383904):1.946753):5.587945):7.300438):18.694303):34.505698,(((Prohollardia_avita:38.44369173,(Protacanthodes_nimesensis:1,Triacanthidae_Triacanthus_biaculeatus:47.36420652):17.12876448):10.123893,(Aracanidae_Aracana_aurita:54.800001,(Ostraciidae_Ostracion_cubicus:51.000002,Ostraciidae_Rhinesomus_triqueter:51.000002):3.799999):19.816863):3.183137,(((Diodontidae_Diodon_holocanthus:12.198387,Diodontidae_Chilomycterus_schoepfii:12.198387):43.701614,(Tetraodontidae_Canthigaster_bennetti:24.22571,Tetraodontidae_Tetraodon_miurus:24.22571):31.674291):17.334859,((Molidae_Ranzania_laevis:22,Molidae_Mola_mola:22):46.61027,((Monacanthidae_Aluterus_scriptus:27.823604,Monacanthidae_Stephanolepis_hispidus:27.823604):15.976396,(Balistidae_Xanthichthys_ringens:20.7,(Balistidae_Balistes_vetula:19.7,Balistidae_Sufflamen_fraenatum:19.7):1):23.1):24.81027):4.62459):4.565141):21.200001):1):1):1):1):1):1):1):1):1):3.894248):4.592438):5.113313):5.2):6):12.2):1.46362):8.33638):6.58283):11.71717):9.7):9.5):19):3.553787):17.946213):17.8):23.2):9.5):1):1):37.2):1):1):1):1):23.6):1):7.291640481):1):10.96415915):17.81113563):1):1):1):1):2.833065743):1):47);

((Guiyu_oneiros:1,(Onychodus_jandemarrai:32.12777601,((Diplocercides:1,(Latimeriidae_Latimeria_chalumnae:330.9783958,Rhabdoderma:1):39.73214545):41.80508573,((Neoceratodontidae_Neoceratodus_forsteri:279.7982796,Lepidosirenidae_Lepidosiren_paradoxa:279.7982796):130.5484995,((Osteolepis_macrolepidotus:1,Gyroptychius_milleri:18.24625079):1,Eusthenopterus_foordi:27.17404918):1):2.168847902):1):11.36772028):18.11665472,((Cheirolepis_trailli:1,Cheirolepis_schultzei:7.061188288):2.817695256,((Polypteridae_Erpetoichthys_calabaricus:29.200001,Polypteridae_Polypterus_senegalus:29.200001):365.800001,(Osorioichthys_marginis:29.30484674,((Tegeolepis_clarki:18.10390462,Howqualepis_rostridens:1):2.18837487,((Gogosardina_coatesi:2.846839232,(Mimipiscis_bartrami:1,Mimipiscis_toombsi:4.732662817):1):6.368656177,((Moythomasia_lineata:1,Moythomasia_durgaringa:7.452910172):1.110595518,(Stegotrachelus_finlayi:1,(Limnomis_delaneyi:1,(Wendyichthys_dicksoni:29.31448624,(Kentuckia_deani:1,((Mesopoma_planti:1,Mesopoma_carricki:7.235036038):8.479881273,((Birgeria_stensioei:1,(Chondrosteus_acipenseroides:1,(Saurichthys_dawaziensis:1,((Protopsephurus_liui:1,Polyodontidae_Polyodon_spathula:130.1779361):8.722065895,(Acipenseridae_Acipenser_fulvescens:38.579335,(Acipenseridae_Scaphirhynchus_platorynchus:19.375515,Acipenseridae_Scaphirhynchus_albus:19.375515):19.20382):100.320667):24.04375475):38.91256375):50.60772545):97.63595505,(Boreosomus:75.76789444,(((Perleidus_altolepis:83.40412969,(Luganoia_lepidosteoides:89.44468069,(((Macrosemius_fourneti:92.17247315,((Kyphosichthys_grandei:1,Lepisosteidae_Lepisosteus_osseus:247.2572221):1,Semionotus_elegans:48.31311066):1):18.64277892,(Watsonulus_eugnathoides:1,(Amblysemius:1,(Pachyamia_latimaxillaris:1,(Tomognathus_mordax:1,Amiidae_Amia_calva:96.9289864):4.502720698):47.94547577):103.5919676):14.93085052):54.6,(Pachycormus:104.0701424,(Crossognathus_danubiensis:180.2173912,(((Anaethalion_angustus:1.254606829,Anaethalion_knorri:1):46.91360063,((Megalopidae_Megalops_atlanticus:133.565966,Elopidae_Elops_saurus:133.565966):62.634035,(Albulidae_Albula_vulpes:150.800001,((Notacanthidae_Notacanthus_chemnitzii:50.669287,(Halosauridae_Halosauropsis_macrochir:40.366211,Halosauridae_Aldrovandia_affinis:40.366211):10.303076):50.330714,(((Eurypharyngidae_Eurypharynx_pelecanoides:25.866364,Saccopharyngidae_Saccopharynx_ampullaceus:25.866364):44.322494,(Nemichthyidae_Nemichthys_scolopaceus:58.739485,Anguillidae_Anguilla_rostrata:58.739485):11.449373):9.198895,(Serrivomeridae_Serrivomer_beanii:76.053487,(Congridae_Conger_oceanicus:65.416147,(Muraenesocidae_Muraenesox_cinereus:57.193624,Ophichthidae_Myrichthys_maculosus:57.193624):8.222523):10.63734):3.334266):21.612248):49.8):45.4):1):86.1,((Lycoptera_davidi:97.77617578,(Paralycoptera_wui:104.672724,(Xixiaichthys_tongxinens:100.6248329,(((Eohiodon_woodruffi:1,Eohiodon_rosei:5.74450056):1,(Hiodon_consteniorum:1,(Hiodontidae_Hiodon_tergisus:9.520291,Hiodontidae_Hiodon_alosoides:9.520291):28.25330269):17.40740147):171.9190058,(Chauliopareion_mahengeense:119.0429813,(Pantodontidae_Pantodon_buchholzi:163.100001,((((Brychaetus_muelleri:1,Phareodus_encaustus:10.43513101):25.82967256,Arapaimidae_Arapaima_gigas:79.3):25.549112,Osteoglossidae_Osteoglossum_bicirrhosum:104.849112):43.475578,(Notopteridae_Xenomystus_nigri:120.300002,Gymnarchidae_Gymnarchus_niloticus:120.300002):28.024688):14.775311):1):63):1):1):1):43.7,((((Diplomystus_brevissimus:47.23952984,Sorbinichthys_africanus:1):88.46089116,(Denticipitidae_Denticeps_clupeoides:188.900001,(Chirocentridae_Chirocentrus_dorab:87.096454,((Engraulidae_Coilia_nasus:47.06527,Engraulidae_Engraulis_mordax_eurystole:47.06527):33.347234,((Pristigasteridae_Pellona_flavipinnis:27.450803,Pristigasteridae_Ilisha_elongata:27.450803):46.430359,(Clupeidae_Alosa_pseudoharengus:54.138864,Clupeidae_Dorosoma_cepedianum:54.138864):19.742298):6.531342):6.68395):101.803547):1):40.3,((Alepocephalidae_Talismania_bifurcata:53.266521,((Alepocephalidae_Bathylaco_nigricans:33.802485,Alepocephalidae_Alepocephalus_tenebrosus:33.802485):10.988666,((Alepocephalidae_Rouleina_attrita:22.275085,Alepocephalidae_Xenodermichthys_copei:22.275085):19.924916,Platytroctidae_Sagamichthys_abei:42.200001):2.59115):8.47537):166.430015,((Gonorynchidae_Gonorynchus_abbreviatus:175.900001,(Mahengichthys_singidaensis:100.1045117,Chanidae_Chanos_chanos:147.100001):28.8):22.7,((((Gyrinocheilidae_Gyrinocheilus_sp:70.111694,(Catostomidae_Hypentelium_nigricans:23.594381,(Catostomidae_Carpiodes_carpio:16.355089,Catostomidae_Ictiobus_bubalus:16.355089):7.239292):46.517313):8.688307,Cobitidae_Cobitis_taenia:78.800001):20.5,(Cyprinidae_Danio_rerio:63.300001,(((Cyprinidae_Zacco_sieboldii_platypus:12.727958,Cyprinidae_Opsariichthys_uncirostris_bidens:12.727958):14.610086,((Cyprinidae_Xenocypris_argentea:12.904177,Cyprinidae_Hypophthalmichthys_molitrix:12.904177):2.917503,(Cyprinidae_Luciobrama_macrocephalus:13.439618,(Cyprinidae_Squaliobarbus_curriculus:11.472717,Cyprinidae_Mylopharyngodon_piceus:11.472717):1.966901):2.382062):11.516364):12.401957,(Cyprinidae_Tanakia_lanceolata_himantegus:35.004261,(Cyprinidae_Notemigonus_crysoleucas:22.933404,(Cyprinidae_Semotilus_atromaculatus:21.325215,((Cyprinidae_Campostoma_oligolepis:12.800846,Cyprinidae_Rhinichthys_cataractae:12.800846):4.408707,(Cyprinidae_Phenacobius_uranops:12.822623,(Cyprinidae_Pimephales_promelas_notatus:9.460336,(Cyprinidae_Luxilus_coccogenis:6.738861,Cyprinidae_Notropis_asperifrons:6.738861):2.721475):3.362287):4.38693):4.115662):1.608189):12.070857):4.73574):23.56):36):73.6,((Gymnotidae_Electrophorus_electricus:63.909447,Gymnotidae_Gymnotus_sp:63.909447):83.924067,(((Distichodontidae_Distichodus_maculatus:103.604365,Citharinidae_Citharinus_congicus:103.604365):11.170138,(Alestidae_Alestes_baremoze:106.666979,((Parodontidae_Parodon_nasus:68.800001,Hemiodontidae_Hemiodus_immaculatus:68.800001):22.415197,((Bryconidae_Brycon_pesu:66.003418,(Gasteropelecidae_Thoracocharax_stellatus:27.746828,Gasteropelecidae_Gasteropelecus_sternicla:27.746828):38.25659):1.227196,Characidae_Astyanax_mexicanus:67.230614):23.984584):15.451781):8.107524):22.236489,((Nematogenyidae_Nematogenys_inermis:110.648758,(Trichomycteridae_Trichomycterus_sp:105.886466,((Loricariidae_Loricaria_simillima:70.313425,Astroblepidae_Astroblepus_sp:70.313425):28.122359,(Callichthyidae_Callichthys_callichthys:69.200001,Callichthyidae_Corydoras_trilineatus:69.200001):29.235783):7.450682):4.762292):5.151243,(Diplomystidae_Diplomystes_nahuelbutaensis:106.203801,((Clariidae_Clarias_batrachus:47.60069,Heteropneustidae_Heteropneustes_fossilis:47.60069):38.718856,(((Sisoridae_Bagarius_yarrelli:55.522058,(Amblycipitidae_Liobagrus_aequilabris:48.027606,Akysidae_Akysis_sp:48.027606):7.494452):21.358911,(Schilbeidae_Pseudeutropius_brachypopterus:74.448774,(Bagridae_Mystus_bocourti:36.077943,Bagridae_Bagrus_ubangensis:36.077943):38.370831):2.432195):8.408138,(((Chacidae_Chaca_sp:72.429482,Cetopsidae_Cetopsis_coecutiens:72.429482):10.714797,(Cranoglanididae_Cranoglanis_bouderius:68.700001,Ictaluridae_Ictalurus_punctatus:68.700001):14.444278):1.039619,(Pangasiidae_Pangasianodon_hypophthalmus:82.158061,((Amphiliidae_Amphilius_jacksonii:69.199757,(Malapteruridae_Malapterurus_beninensis:64.740321,Mochokidae_Synodontis_batesii:64.740321):4.459436):10.993535,((Plotosidae_Plotosus_lineatus:73.039645,Siluridae_Hemisilurus_moolenburghi:73.039645):5.503862,(Auchenipteridae_Ageneiosus_atronasus:51.101654,Doradidae_Anduzedoras_oxyrhynchus:51.101654):27.441853):1.649785):1.964769):2.025837):1.105209):1.030439):19.884255):9.5962):21.210991):10.822522):25.066487):25.7):21.096535):10.503465):20.4,(Orthogonikleithrus_francogalliensis:75.94906163,(((Bathylagidae_Bathylagus_euryops:70.542512,(Microstomatidae_Nansenia_longicauda_ardesiaca:51.394,(Opisthoproctidae_Macropinna_microstoma:33.11268,Argentinidae_Argentina_sialis_silus:33.11268):18.28132):19.148512):89.057489,((Galaxiidae_Neochanna_burrowsius:34.505049,Galaxiidae_Galaxias_maculatus:34.505049):111.121934,(((Umbridae_Novumbra_hubbsi:55.762491,Umbridae_Umbra_limi:55.762491):23.63751,(Esocidae_Esox_lucius:31.366195,Esocidae_Esox_americanus:31.366195):48.033806):25.064928,(Salmonidae_Coregonus_clupeaformis:35.300001,(Salmonidae_Thymallus_brevirostris:32.456522,(Salmonidae_Oncorhynchus_nerka_mykiss:19.929533,(Salmonidae_Salvelinus_alpinus:16.737138,Salmonidae_Salmo_salar:16.737138):3.192395):12.526989):2.843479):69.164928):41.162054):13.973018):55.253787,(((Retropinnidae_Retropinna_semoni:73.700001,(((Osmeridae_Osmerus_mordax:10.332899,Osmeridae_Thaleichthys_pacificus:10.332899):5.872101,(Osmeridae_Mallotus_villosus:14.961758,Osmeridae_Hypomesus_pretiosus:14.961758):1.243242):18.133836,(Plecoglossidae_Plecoglossus_altivelis:28.70318,Salangidae_Neosalangichthys_ishikawae:28.70318):5.635656):39.361165):55.7,((Diplophidae_Diplophos_taenia:77.330737,((Gonostomatidae_Bonapartia_pedaliota:54.297445,Gonostomatidae_Margrethia_obtusirostra:54.297445):20.014374,(Gonostomatidae_Gonostoma_elongatum:69.948163,Gonostomatidae_Cyclothone_microdon:69.948163):4.363656):3.018918):6.469264,(Phosichthyidae_Pollichthys_mauli:77.722827,((Sternoptychidae_Argyropelecus_gigas:25.230481,Sternoptychidae_Maurolicus_weitzmani:25.230481):47.329876,(Stomiidae_Chauliodus_macouni_danae:63.126299,(Stomiidae_Stomias_boa:36.381906,(Stomiidae_Chirostomias_pliopterus:31.578039,((Stomiidae_Melanostomias_margaritifer:21.790927,Stomiidae_Leptostomias_longibarba:21.790927):6.240428,((Stomiidae_Photonectes_margarita:19.053467,Stomiidae_Tactostoma_macropus:19.053467):7.977888,(Stomiidae_Malacosteus_niger:25.031355,(Stomiidae_Eustomias_polyaster:24.031355,(Stomiidae_Aristostomias_scintillans:23.031355,Stomiidae_Bathophilus_flemingi_pawneei:23.031355):1):1):2):1):3.546684):4.803867):26.744393):9.434058):5.16247):6.077174):45.6):81.9,((Ateleopodidae_Ateleopus_japonicus:8.089622,Ateleopodidae_Ijimaia_antillarum:8.089622):184.210379,(((Synodontidae_Trachinocephalus_myops:34.928634,Synodontidae_Synodus_foetens:34.928634):78.971367,((Paraulopidae_Paraulopus_oblongus:101.985288,(Synodontidae_Saurida_gracilis:50.257509,Synodontidae_Harpadon_microchir:50.257509):51.727779):8.326796,(((Aulopidae_Aulopus_filamentosus:47.160387,Bathysauridae_Bathysaurus_ferox:47.160387):10.843902,(Ipnopidae_Ipnops_murrayi:13.82,Ipnopidae_Ipnops_agassizi:13.82):44.184289):23.635948,((Ipnopidae_Bathypterois_atricolor:69.328167,(Giganturidae_Gigantura_indica:13.525058,Giganturidae_Gigantura_chuni:13.525058):55.803109):6.462647,(Scopelarchidae_Benthalbella_macropinna:62.490696,(Chlorophthalmidae_Chlorophthalmus_agassizi:61.13635,(Sudidae_Sudis_atrox:55.026295,((Alepisauridae_Anotopterus_pharao:45.661943,(Alepisauridae_Omosudis_lowii:36.284216,Alepisauridae_Alepisaurus_ferox:36.284216):9.377727):8.238175,(Paralepididae_Stemonosudis_intermedia_macrura:50.122687,(Evermannellidae_Evermannella_balbo:46.686813,Paralepididae_Paralepis_coregonoides:46.686813):3.435874):3.777431):1.126177):6.110055):1.354346):13.300118):5.849423):28.671847):3.587917):68.9,(((Neoscopelidae_Neoscopelus_macrolepidotus:42.408365,Neoscopelidae_Scopelengys_tristis:42.408365):31.191636,((Myctophidae_Lampadena_speculigera:39.81673,Myctophidae_Lampanyctus_macdonaldi:39.81673):11.872,(Myctophidae_Benthosema_glaciale:28.30241,Myctophidae_Myctophum_punctatum:28.30241):23.38632):21.911271):99.5,(((Lamprididae_Lampris_guttatus:65.552505,(Regalecidae_Regalecus_russelii:37.682943,Trachipteridae_Trachipterus_trachypterus:37.682943):27.869562):84.747496,(((Mcconichthys_longipinnis:1,(Aphredoderidae_Aphredoderus_sayanus:41.500001,Amblyopsidae_Chologaster_cornuta:41.500001):22.27388374):1,(Lateopisciculus_turrifumosus:1,(Percopsidae_Percopsis_omiscomaycus:14.617149,Percopsidae_Percopsis_transmontana:14.617149):44.53988011):5.616855636):70.21644726,(Zeidae_Zeus_faber:107.100001,(Stylephoridae_Stylephorus_chordatus:78.800001,(Merlucciidae_Merluccius_productus:45.655086,(((Macrouridae_Gadomus_dispar:9.947595,Macrouridae_Bathygadus_favosus:9.947595):28.939269,(Steindachneriidae_Steindachneria_argentea:35.929071,((Macrouridae_Malacocephalus_laevis:17.761718,Macrouridae_Trachonurus_sulcatus:17.761718):10.126413,(Macrouridae_Coelorinchus_caribbaeus:21.883517,Macrouridae_Coryphaenoides_armatus:21.883517):6.004614):8.04094):2.957793):2.613137,((Moridae_Laemonema_goodebeanorum:25.08833,(Moridae_Halargyreus_johnsonii:16.444614,(Moridae_Lepidion_ensiferus:5.35693,Moridae_Antimora_rostrata:5.35693):11.087684):8.643716):13.1716,((Phycidae_Urophycis_tenuis:5.240351,Phycidae_Urophycis_chuss:5.240351):28.607969,(Lotidae_Gaidropsarus_ensis:29.800286,(Lotidae_Lota_lota:23.935071,(Gadidae_Melanogrammus_aeglefinus:5.86839,Gadidae_Gadus_morhua:5.86839):18.066681):5.865215):4.048034):4.41161):3.240071):4.155085):33.144915):28.3):27.890331):15.309669):11.08283,(Polymixiidae_Polymixia_japonica:154.800001,(((Diretmidae_Diretmus_argenteus:53.99855,(Monocentridae_Monocentris_japonica:33.600001,((Trachichthyidae_Hoplostethus_occidentalis_atlanticus:24.361165,Trachichthyidae_Gephyroberyx_darwinii:24.361165):7.919156,(Anoplogastridae_Anoplogaster_cornuta:31.22776,Trachichthyidae_Paratrachichthys_sajademalensis:31.22776):1.052561):1.31968):20.398549):71.275924,((Berycidae_Beryx_decadactylus:70.612394,(Melamphaidae_Poromitra_crassiceps:39.138751,(Melamphaidae_Scopelogadus_beanii:32.603956,Melamphaidae_Melamphaes_suborbitalis:32.603956):6.534795):31.473643):40.295775,(((Rondeletiidae_Rondeletia_bicolor:18.616456,Rondeletiidae_Rondeletia_loricata:18.616456):36.662305,Barbourisiidae_Barbourisia_rufa:55.278761):34.605685,(Cetomimidae_Cetostoma_regani:55.492218,(Cetomimidae_Cetomimus_craneae:10.537863,Cetomimidae_Cetomimus_compuctus:10.537863):44.954355):34.392228):21.023723):14.366305):21.189147,((Holocentridae_Sargocentron_diadema:25.43456,Holocentridae_Holocentrus_rufus:25.43456):119.565441,((Ophidiidae_Brotula_multibarbata:66.227334,(Ophidiidae_Lepophidium_profundorum:23.036156,(Ophidiidae_Genypterus_blacodes:21.590629,Ophidiidae_Ophidion_holbrookii:21.590629):1.445527):43.191178):66.572667,((Batrachoididae_Porichthys_notatus:39.800001,Batrachoididae_Opsanus_tau:39.800001):87,(((Kurtidae_Kurtus_indicus:80.300001,Apogonidae_Apogon_campbelli:80.300001):22,(Eleotridae_Eleotris_acanthopoma_pisonis:64.430477,(Gobiidae_Gobiosoma_bosc:45.630567,Microdesmidae_Microdesmus_longipinnis:45.630567):18.79991):37.869524):19.3,((((Dactylopteridae_Dactylopterus_volitans:68.121568,(Aulostomidae_Aulostomus_maculatus:3.200001,Aulostomidae_Aulostomus_chinensis:3.200001):64.921567):6.178433,((Fistulariidae_Fistularia_petimba:65.463647,Mullidae_Mullus_auratus:65.463647):6.26906,(Callionymidae_Callionymus_sp_bairdi:69.154942,Syngnathidae_Syngnathus_fuscus:69.154942):2.577765):2.567294):20.27,(Chiasmodontidae_Chiasmodon_sp:43.900001,((Centrolophidae_Icichthys_lockingtoni:34.370774,Pomatomidae_Pomatomus_saltatrix:34.370774):6.934458,(((Ariommatidae_Ariomma_bondi:25.644121,Nomeidae_Psenes_cyanophrys:25.644121):5.370208,Scombridae_Auxis_rochei:31.014329):6.588438,((Stromateidae_Peprilus_paru:35.602767,(Gempylidae_Paradiplospinus_gracilis:31.499638,Bramidae_Brama_brama:31.499638):4.103129):1,(Icosteidae_Icosteus_aenigmaticus:33.972252,Trichiuridae_Trichiurus_lepturus:33.972252):2.630515):1):3.702465):2.594769):50.67):21.916687,((((((Synbranchidae_Monopterus_albus:69.146846,Indostomidae_Indostomus_paradoxus:69.146846):4.548479,(Mastacembelidae_Macrognathus_siamensis:18.057555,Mastacembelidae_Mastacembelus_erythrotaenia:18.057555):55.63777):7.004676,((Anabantidae_Ctenopoma_acutirostre_kingsleyae:62.782323,(Helostomatidae_Helostoma_temminkii:59.687826,Osphronemidae_Trichopodus_pectoralis:59.687826):3.094497):7.86905,(Channidae_Channa_striata:67.428514,Nandidae_Nandus_nandus:67.428514):3.222859):10.048628):15.7,((Nematistiidae_Nematistius_pectoralis:60.067614,((Coryphaenidae_Coryphaena_hippurus:19.124214,Rachycentridae_Rachycentron_canadum:19.124214):12.775787,(Echeneidae_Remora_osteochir_australis:10.178122,Echeneidae_Echeneis_naucrates:10.178122):21.721879):28.167613):9.732387,((Sphyraenidae_Sphyraena_sphyraena:65.900885,(Menidae_Mene_maculata:60.339531,Polynemidae_Polydactylus_octonemus:60.339531):5.561354):2.899116,((((Leptobramidae_Leptobrama_muelleri:46.58559,Toxotidae_Toxotes_jaculatrix:46.58559):5.096327,(Xiphiidae_Xiphias_gladius:27.833842,Istiophoridae_Istiophorus_platypterus:27.833842):23.848075):5.298938,Carangidae_Trachinotus_falcatus:56.980855):4.861848,((Eolates_gracilis:1,(Centropomidae_Psammoperca_waigiensis:33.068144,(Centropomidae_Lates_calcarifer:13.595548,Centropomidae_Lates_microlepis:13.595548):19.472596):18.86730096):8.907258043,(Heteronectes_chaneti:8.220956377,(Amphistium_paradoxum:5.859527994,(Psettodidae_Psettodes_erumei:57.842703,(Citharidae_Lepidoblepharon_ophthalmolepis:53.600001,((Cynoglossidae_Cynoglossus_interruptus:42.400002,(Soleidae_Solea_solea:24.944331,Soleidae_Soleichthys_heterorhinos:24.944331):17.455671):9.674607,((Scophthalmidae_Scophthalmus_aquosus:23.305906,Scophthalmidae_Lepidorhombus_boscii:23.305906):26.973285,((Paralichthyidae_Paralichthys_dentatus:40.749719,Bothidae_Bothus_lunatus:40.749719):4.387561,(Paralichthyidae_Pseudorhombus_pentophthalmus:21.404631,(((Pleuronectidae_Glyptocephalus_zachirus:5.096079,Pleuronectidae_Microstomus_pacificus:5.096079):2.538752,(Pleuronectidae_Lyopsetta_exilis:6.085211,Pleuronectidae_Hippoglossus_hippoglossus:6.085211):1.54962):2.215451,(Pleuronectidae_Limanda_limanda:8.798272,((Pleuronectidae_Platichthys_stellatus:3.717184,Pleuronectidae_Pleuronectes_platessa:3.717184):3.081088,(Pleuronectidae_Lepidopsetta_bilineata:4.798272,Pleuronectidae_Psettichthys_melanostictus:4.798272):2):2):1.05201):11.554349):23.732649):5.141911):1.795418):1.525392):4.242702):1):1):1):1):6.957298):1):26.6):12.043384,(((Polycentridae_Polycentrus_schomburgkii:94.214904,(Pholidichthyidae_Pholidichthys_leucotaenia:88.700001,Cichlidae_Cichla_temensis:88.700001):5.514903):1,(((Atherinopsidae_Atherinopsis_californiensis:48.715388,Atherinopsidae_Menidia_menidia:48.715388):22.225577,(Isonidae_Iso_sp:49.317292,(Atherinidae_Atherinomorus_stipes:43.78646,(Melanotaeniidae_Melanotaenia_splendida:39.870052,Pseudomugilidae_Pseudomugil_gertrudae:39.870052):3.916408):5.530832):21.623673):6.46052,(((Poeciliidae_Poecilia_latipinna_reticulata:14.292152,(Poeciliidae_Heterandria_formosa:11.366823,(Poeciliidae_Gambusia_affinis:5.736451,Poeciliidae_Belonesox_belizanus:5.736451):5.630372):2.925329):30.13151,(Cyprinodontidae_Cyprinodon_variegatus:40.469994,Fundulidae_Fundulus_parvipinnis:40.469994):3.953668):31.977823,(Adrianichthyidae_Oryzias_latipes:71.878571,((Zenarchopteridae_Dermogenys_collettei:29.883878,((Belonidae_Tylosurus_crocodilus:6.568056,Belonidae_Ablennes_hians:6.568056):17.429484,(Scomberesocidae_Cololabis_saira:2.646712,Scomberesocidae_Scomberesox_saurus:2.646712):21.350828):5.886338):17.178962,(Hemiramphidae_Hyporhamphus_affinis:36.892311,((Hemiramphidae_Hemiramphus_brasiliensis:13.391766,Hemiramphidae_Oxyporhamphus_micropterus:13.391766):10.259915,((Exocoetidae_Cypselurus_callopterus:6.413654,Exocoetidae_Exocoetus_monocirrhus:6.413654):4.605967,Exocoetidae_Cheilopogon_pinnatibarbatus:11.019621):12.63206):13.24063):10.170529):24.815731):4.522914):1):17.813419):1.999954,((Pseudochromidae_Halidesmus_scapularis:84.850287,Pomacentridae_Pomacentrus_brachialis:84.850287):8.187103,(Embiotocidae_Cymatogaster_aggregata:91.529056,(Mugilidae_Mugil_cephalus:89.757655,(Plesiopidae_Plesiops_coeruleolineatus:86.977996,(Opistognathidae_Opistognathus_maxillosus:79.196597,(((Gobiesocidae_Lepadichthys_lineatus:37.560081,Gobiesocidae_Gobiesox_maeandricus:37.560081):35.985217,Tripterygiidae_Enneanectes_altivelis:73.545298):3.122761,((Clinidae_Clinus_cottoides:50.741024,(Dactyloscopidae_Platygillellus_rubrocinctus:45.641062,Chaenopsidae_Chaenopsis_alepidota:45.641062):5.099962):17.520576,(Blenniidae_Stanulus_seychellensis:31.042358,Blenniidae_Blenniella_cyanostigma:31.042358):37.219242):8.406459):2.528538):7.781399):2.779659):1.771401):1.508334):4.177468):11.228527):3.450865,(Gerreidae_Eucinostomus_argenteus:108.000002,(((Labridae_Lachnolaimus_maximus:59.822938,Odacidae_Haletta_semifasciata:59.822938):16.826114,Scaridae_Scarus_globiceps:76.649052):30.35095,(((Uranoscopidae_Uranoscopus_sulphureus:83.996275,(Ammodytidae_Ammodytes_hexapterus:78.584442,Pinguipedidae_Parapercis_punctulata:78.584442):5.411833):11.903726,(((Acropomatidae_Acropoma_japonicum:90.360051,((Percophidae_Acanthaphritis_unoorum:66.16617,Creediidae_Limnichthys_sp:66.16617):18.796708,(Glaucosomatidae_Glaucosoma_buergeri:66.617288,Pempheridae_Pempheris_schomburgkii:66.617288):18.34559):5.397173):2.814411,(((Oplegnathidae_Oplegnathus_punctatus:61.206964,Kuhliidae_Kuhlia_rupestris:61.206964):7.298332,Kyphosidae_Kyphosus_sectatrix:68.505296):16.157201,(Percichthyidae_Percichthys_trucha:69.999181,((Cirrhitidae_Cirrhitichthys_falco:66.467051,Cheilodactylidae_Cheilodactylus_fasciatus:66.467051):2.53213,(Enoplosidae_Enoplosus_armatus:63.04721,Centrarchidae_Acantharchus_pomotis:63.04721):5.951971):1):14.663316):8.511965):1.725539,(Serranidae_Pseudogramma_polyacantha:82.200001,(Percidae_Perca_fluviatilis:78.577195,((Bovichtidae_Bovichtus_diacanthus:63.021097,(Nototheniidae_Notothenia_coriiceps:9.671082,(Harpagiferidae_Harpagifer_antarcticus:8.390321,(Bathydraconidae_Gymnodraco_acuticeps:7.390321,Channichthyidae_Chionodraco_rastrospinosus:7.390321):1):1.280761):53.350015):12.936917,(((Peristediidae_Peristedion_ecuadorense:54.532565,Triglidae_Prionotus_carolinus:54.532565):11.953546,(Synanceiidae_Synanceia_verrucosa:62.510934,Scorpaenidae_Scorpaenodes_guamensis:62.510934):3.975177):6.621302,(Platycephalidae_Platycephalus_indicus:70.795272,(Anoplopomatidae_Anoplopoma_fimbria:46.504594,((Bathymasteridae_Rathbunella_hypoplecta:22.798552,(Stichaeidae_Cebidichthys_violaceus:15.58542,(Zoarcidae_Zoarces_americanus_viviparus:12.493576,(Anarhichadidae_Anarrhichthys_ocellatus:9.933504,(Zaproridae_Zaprora_silenus:8.933504,Cryptacanthodidae_Cryptacanthodes_maculatus:8.933504):1):2.560072):3.091844):7.213132):19.028758,(((Aulorhynchidae_Aulorhynchus_flavidus:25.280385,Hypoptychidae_Hypoptychus_dybowskii:25.280385):1.019618,((Gasterosteidae_Culaea_inconstans:19.844899,(Gasterosteidae_Apeltes_quadracus:17.136644,Gasterosteidae_Spinachia_spinachia:17.136644):2.708255):1.782129,(Gasterosteidae_Pungitius_pungitius:16.827665,Gasterosteidae_Gasterosteus_aculeatus:16.827665):4.799363):4.672975):13.53479,(Hexagrammidae_Hexagrammos_decagrammus:27.818969,((Cyclopteridae_Cyclopterus_lumpus:20.762394,Liparidae_Liparis_pulchellus:20.762394):4.037609,(Agonidae_Hypsagonus_quadricornis:20.371382,(Cottidae_Cottus_carolinae:18.19359,Psychrolutidae_Psychrolutes_phrictus:18.19359):2.177792):4.428621):3.018966):12.015824):1.992517):4.677284):24.290678):2.312141):2.850601):2.619181):3.622806):12.7):1):10.100001,((Drepaneidae_Drepane_punctata:67.658011,Ephippidae_Chaetodipterus_faber:67.658011):37.341991,(((Lobotidae_Lobotes_pacificus_surinamensis:86.438004,Sciaenidae_Menticirrhus_undulatus_littoralis:86.438004):5.761997,((Monodactylidae_Monodactylus_sebae:70.333247,(Avitoluvarus_eocaenicus:19.88655809,(Zanclidae_Zanclus_cornutus:55.859022,Acanthuridae_Acanthurus_triostegus:55.859022):4.740979):9.733246):15.366754,((Pomacanthidae_Pomacanthus_semicirculatus:54.434118,(Leiognathidae_Leiognathus_equulus:32.700001,Chaetodontidae_Chelmon_rostratus:32.700001):21.734117):17.438962,(Emmelichthyidae_Erythrocles_schlegelii:69.871718,(Malacanthidae_Malacanthus_plumieri:68.303733,(Haemulidae_Haemulon_aurolineatum:64.600001,Lutjanidae_Lutjanus_griseus:64.600001):3.703732):1.567985):2.001362):13.826921):6.5):11.800001,((Sillaginidae_Sillago_sihama:85.853361,(Nemipteridae_Pentapodus_caninus:80.270573,(Lethrinidae_Lethrinus_erythropterus:74.370539,Sparidae_Stenotomus_chrysops:74.370539):5.900034):5.582788):17.146641,(Siganidae_Siganus_spinus:102.000002,((Scatophagidae_Scatophagus_argus:75.860371,Priacanthidae_Heteropriacanthus_cruentatus:75.860371):25.139631,((Caproidae_Antigonia_rubescens:10.905674,Caproidae_Antigonia_capros:10.905674):89.094328,(((Lophiidae_Lophiodes_reticulatus:22.462185,Lophiidae_Lophius_gastrophysus:22.462185):42.032119,((Antennariidae_Histrio_histrio:12.333102,Antennariidae_Antennatus_coccineus:12.333102):33.466899,(Chaunacidae_Chaunax_suttkusi:38.499563,(Gigantactinidae_Gigantactis_sp:32.911618,((Ceratiidae_Cryptopsaras_couesii:20.548708,Ceratiidae_Ceratias_holboelli:20.548708):10.416157,((Himantolophidae_Himantolophus_albinares_sagamius:14.456802,Melanocetidae_Melanocetus_murrayi:14.456802):9.124159,(Oneirodidae_Oneirodes_macrosteus:10.164357,Oneirodidae_Dolopichthys_sp:10.164357):13.416604):7.383904):1.946753):5.587945):7.300438):18.694303):34.505698,(((Prohollardia_avita:40.71652462,(Protacanthodes_nimesensis:1,Triacanthidae_Triacanthus_biaculeatus:45.74753692):18.74543408):10.123893,(Aracanidae_Aracana_aurita:54.800001,(Ostraciidae_Ostracion_cubicus:51.000002,Ostraciidae_Rhinesomus_triqueter:51.000002):3.799999):19.816863):3.183137,(((Diodontidae_Diodon_holocanthus:12.198387,Diodontidae_Chilomycterus_schoepfii:12.198387):43.701614,(Tetraodontidae_Canthigaster_bennetti:24.22571,Tetraodontidae_Tetraodon_miurus:24.22571):31.674291):17.334859,((Molidae_Ranzania_laevis:22,Molidae_Mola_mola:22):46.61027,((Monacanthidae_Stephanolepis_hispidus:28.823604,Monacanthidae_Aluterus_scriptus:28.823604):14.976396,(Balistidae_Xanthichthys_ringens:20.7,(Balistidae_Balistes_vetula:19.7,Balistidae_Sufflamen_fraenatum:19.7):1):23.1):24.81027):4.62459):4.565141):21.200001):1):1):1):1):1):1):1):1):1):3.894248):4.592438):5.113313):5.2):6):12.2):1.46362):8.33638):6.58283):11.71717):9.7):9.5):19):3.553787):17.946213):17.8):23.2):9.5):1):1):37.2):1):1):1,Australosomus:73.41476664):1,(Fukangichthys_longidorsalis:9.203881176,(Scanilepis_dubia:48.86223138,Evenkia_eunotoptera:1):1):72.90017144):1):22.6):1):4.005527899):1):12.86081515):18.75581953):1):1):1):1):3.277838418):1):47);

((Guiyu_oneiros:1,(Onychodus_jandemarrai:31.86830367,((Diplocercides:1,(Latimeriidae_Latimeria_chalumnae:328.8731166,Rhabdoderma:1):43.18131748):40.46119292,((Neoceratodontidae_Neoceratodus_forsteri:279.7982796,Lepidosirenidae_Lepidosiren_paradoxa:279.7982796):128.3405626,((Osteolepis_macrolepidotus:1,Gyroptychius_milleri:16.17552522):1,Eusthenopterus_foordi:29.92577509):1):4.376784807):1):12.87890778):16.60546722,((Cheirolepis_trailli:1,Cheirolepis_schultzei:4.262178398):5.506622912,((Polypteridae_Erpetoichthys_calabaricus:29.200001,Polypteridae_Polypterus_senegalus:29.200001):365.800001,(Osorioichthys_marginis:18.81855902,((Tegeolepis_clarki:22.90711753,Howqualepis_rostridens:1):1.431015053,((Gogosardina_coatesi:1.399229479,(Mimipiscis_bartrami:1,Mimipiscis_toombsi:4.412484559):1):6.617901345,((Moythomasia_lineata:1,Moythomasia_durgaringa:8.426816265):1,(Stegotrachelus_finlayi:1,(Limnomis_delaneyi:1,(Wendyichthys_dicksoni:29.36886847,(Kentuckia_deani:3.116092281,((Mesopoma_planti:5.684353618,Mesopoma_carricki:1):6.599718941,((Birgeria_stensioei:1,(Chondrosteus_acipenseroides:1,(Saurichthys_dawaziensis:1,((Protopsephurus_liui:1,Polyodontidae_Polyodon_spathula:129.2881152):9.611886825,(Acipenseridae_Acipenser_fulvescens:38.579335,(Acipenseridae_Scaphirhynchus_platorynchus:19.375515,Acipenseridae_Scaphirhynchus_albus:19.375515):19.20382):100.320667):24.61820926):37.20109266):52.37381091):97.00688617,(Boreosomus:75.63127954,(((Perleidus_altolepis:82.69433379,(Luganoia_lepidosteoides:89.53203827,(((Macrosemius_fourneti:93.33861612,((Lepisosteidae_Lepisosteus_osseus:247.5484112,Kyphosichthys_grandei:1):1,Semionotus_elegans:48.60724966):1):18.35158976,(Watsonulus_eugnathoides:1,(Amblysemius:1,(Pachyamia_latimaxillaris:2.424460425,(Tomognathus_mordax:1,Amiidae_Amia_calva:99.12212162):1):52.50347325):99.74928739):15.52511874):54.6,(Pachycormus:103.5475847,(Crossognathus_danubiensis:176.9523809,(((Anaethalion_angustus:1,Anaethalion_knorri:1.142867223):46.14581891,((Megalopidae_Megalops_atlanticus:133.565966,Elopidae_Elops_saurus:133.565966):62.634035,(Albulidae_Albula_vulpes:150.800001,((Notacanthidae_Notacanthus_chemnitzii:50.669287,(Halosauridae_Halosauropsis_macrochir:40.366211,Halosauridae_Aldrovandia_affinis:40.366211):10.303076):50.330714,(((Eurypharyngidae_Eurypharynx_pelecanoides:25.866364,Saccopharyngidae_Saccopharynx_ampullaceus:25.866364):44.322494,(Nemichthyidae_Nemichthys_scolopaceus:58.739485,Anguillidae_Anguilla_rostrata:58.739485):11.449373):9.198895,(Serrivomeridae_Serrivomer_beanii:76.053487,(Congridae_Conger_oceanicus:65.416147,(Muraenesocidae_Muraenesox_cinereus:57.193624,Ophichthidae_Myrichthys_maculosus:57.193624):8.222523):10.63734):3.334266):21.612248):49.8):45.4):1):86.1,((Lycoptera_davidi:100.1519096,(Paralycoptera_wui:105.8965781,(Xixiaichthys_tongxinens:98.80275244,(((Eohiodon_woodruffi:2.101720076,Eohiodon_rosei:1):1,(Hiodon_consteniorum:1,(Hiodontidae_Hiodon_tergisus:9.520291,Hiodontidae_Hiodon_alosoides:9.520291):26.64739616):17.58858803):173.3437258,(Chauliopareion_mahengeense:120.4663502,(Pantodontidae_Pantodon_buchholzi:163.100001,(((Osteoglossidae_Osteoglossum_bicirrhosum:55.03139758,(Brychaetus_muelleri:1,Phareodus_encaustus:9.133804388):1):49.81771442,Arapaimidae_Arapaima_gigas:104.849112):43.475578,(Notopteridae_Xenomystus_nigri:120.300002,Gymnarchidae_Gymnarchus_niloticus:120.300002):28.024688):14.775311):1):63):1):1):1):43.7,((((Diplomystus_brevissimus:44.42870242,Sorbinichthys_africanus:1):90.48713579,(Denticipitidae_Denticeps_clupeoides:188.900001,(Chirocentridae_Chirocentrus_dorab:87.096454,((Engraulidae_Coilia_nasus:47.06527,Engraulidae_Engraulis_mordax_eurystole:47.06527):33.347234,((Pristigasteridae_Pellona_flavipinnis:27.450803,Pristigasteridae_Ilisha_elongata:27.450803):46.430359,(Clupeidae_Alosa_pseudoharengus:54.138864,Clupeidae_Dorosoma_cepedianum:54.138864):19.742298):6.531342):6.68395):101.803547):1):40.3,((Alepocephalidae_Talismania_bifurcata:53.266521,((Alepocephalidae_Bathylaco_nigricans:33.802485,Alepocephalidae_Alepocephalus_tenebrosus:33.802485):10.988666,((Alepocephalidae_Rouleina_attrita:22.275085,Alepocephalidae_Xenodermichthys_copei:22.275085):19.924916,Platytroctidae_Sagamichthys_abei:42.200001):2.59115):8.47537):166.430015,((Gonorynchidae_Gonorynchus_abbreviatus:175.900001,(Mahengichthys_singidaensis:103.3083385,Chanidae_Chanos_chanos:147.100001):28.8):22.7,((((Gyrinocheilidae_Gyrinocheilus_sp:70.111694,(Catostomidae_Hypentelium_nigricans:23.594381,(Catostomidae_Carpiodes_carpio:16.355089,Catostomidae_Ictiobus_bubalus:16.355089):7.239292):46.517313):8.688307,Cobitidae_Cobitis_taenia:78.800001):20.5,(Cyprinidae_Danio_rerio:63.300001,(((Cyprinidae_Zacco_sieboldii_platypus:12.727958,Cyprinidae_Opsariichthys_uncirostris_bidens:12.727958):14.610086,((Cyprinidae_Xenocypris_argentea:12.904177,Cyprinidae_Hypophthalmichthys_molitrix:12.904177):2.917503,(Cyprinidae_Luciobrama_macrocephalus:13.439618,(Cyprinidae_Squaliobarbus_curriculus:11.472717,Cyprinidae_Mylopharyngodon_piceus:11.472717):1.966901):2.382062):11.516364):12.401957,(Cyprinidae_Tanakia_lanceolata_himantegus:35.004261,(Cyprinidae_Notemigonus_crysoleucas:22.933404,(Cyprinidae_Semotilus_atromaculatus:21.325215,((Cyprinidae_Campostoma_oligolepis:12.800846,Cyprinidae_Rhinichthys_cataractae:12.800846):4.408707,(Cyprinidae_Phenacobius_uranops:12.822623,(Cyprinidae_Pimephales_promelas_notatus:9.460336,(Cyprinidae_Luxilus_coccogenis:6.738861,Cyprinidae_Notropis_asperifrons:6.738861):2.721475):3.362287):4.38693):4.115662):1.608189):12.070857):4.73574):23.56):36):73.6,((Gymnotidae_Electrophorus_electricus:63.909447,Gymnotidae_Gymnotus_sp:63.909447):83.924067,(((Distichodontidae_Distichodus_maculatus:103.604365,Citharinidae_Citharinus_congicus:103.604365):11.170138,(Alestidae_Alestes_baremoze:106.666979,((Parodontidae_Parodon_nasus:68.800001,Hemiodontidae_Hemiodus_immaculatus:68.800001):22.415197,((Bryconidae_Brycon_pesu:66.003418,(Gasteropelecidae_Thoracocharax_stellatus:27.746828,Gasteropelecidae_Gasteropelecus_sternicla:27.746828):38.25659):1.227196,Characidae_Astyanax_mexicanus:67.230614):23.984584):15.451781):8.107524):22.236489,((Nematogenyidae_Nematogenys_inermis:110.648758,(Trichomycteridae_Trichomycterus_sp:105.886466,((Loricariidae_Loricaria_simillima:70.313425,Astroblepidae_Astroblepus_sp:70.313425):28.122359,(Callichthyidae_Callichthys_callichthys:69.200001,Callichthyidae_Corydoras_trilineatus:69.200001):29.235783):7.450682):4.762292):5.151243,(Diplomystidae_Diplomystes_nahuelbutaensis:106.203801,((Clariidae_Clarias_batrachus:47.60069,Heteropneustidae_Heteropneustes_fossilis:47.60069):38.718856,(((Sisoridae_Bagarius_yarrelli:55.522058,(Amblycipitidae_Liobagrus_aequilabris:48.027606,Akysidae_Akysis_sp:48.027606):7.494452):21.358911,(Schilbeidae_Pseudeutropius_brachypopterus:74.448774,(Bagridae_Mystus_bocourti:36.077943,Bagridae_Bagrus_ubangensis:36.077943):38.370831):2.432195):8.408138,(((Chacidae_Chaca_sp:72.429482,Cetopsidae_Cetopsis_coecutiens:72.429482):10.714797,(Cranoglanididae_Cranoglanis_bouderius:68.700001,Ictaluridae_Ictalurus_punctatus:68.700001):14.444278):1.039619,(Pangasiidae_Pangasianodon_hypophthalmus:82.158061,((Amphiliidae_Amphilius_jacksonii:69.199757,(Malapteruridae_Malapterurus_beninensis:64.740321,Mochokidae_Synodontis_batesii:64.740321):4.459436):10.993535,((Plotosidae_Plotosus_lineatus:73.039645,Siluridae_Hemisilurus_moolenburghi:73.039645):5.503862,(Auchenipteridae_Ageneiosus_atronasus:51.101654,Doradidae_Anduzedoras_oxyrhynchus:51.101654):27.441853):1.649785):1.964769):2.025837):1.105209):1.030439):19.884255):9.5962):21.210991):10.822522):25.066487):25.7):21.096535):10.503465):20.4,(Orthogonikleithrus_francogalliensis:77.12350258,(((Bathylagidae_Bathylagus_euryops:70.542512,(Microstomatidae_Nansenia_longicauda_ardesiaca:51.394,(Opisthoproctidae_Macropinna_microstoma:33.11268,Argentinidae_Argentina_sialis_silus:33.11268):18.28132):19.148512):89.057489,((Galaxiidae_Neochanna_burrowsius:34.505049,Galaxiidae_Galaxias_maculatus:34.505049):111.121934,(((Umbridae_Novumbra_hubbsi:55.762491,Umbridae_Umbra_limi:55.762491):23.63751,(Esocidae_Esox_lucius:31.366195,Esocidae_Esox_americanus:31.366195):48.033806):25.064928,(Salmonidae_Coregonus_clupeaformis:35.300001,(Salmonidae_Thymallus_brevirostris:32.456522,(Salmonidae_Oncorhynchus_nerka_mykiss:19.929533,(Salmonidae_Salvelinus_alpinus:16.737138,Salmonidae_Salmo_salar:16.737138):3.192395):12.526989):2.843479):69.164928):41.162054):13.973018):55.253787,(((Retropinnidae_Retropinna_semoni:73.700001,(((Osmeridae_Osmerus_mordax:10.332899,Osmeridae_Thaleichthys_pacificus:10.332899):5.872101,(Osmeridae_Mallotus_villosus:14.961758,Osmeridae_Hypomesus_pretiosus:14.961758):1.243242):18.133836,(Plecoglossidae_Plecoglossus_altivelis:28.70318,Salangidae_Neosalangichthys_ishikawae:28.70318):5.635656):39.361165):55.7,((Diplophidae_Diplophos_taenia:77.330737,((Gonostomatidae_Bonapartia_pedaliota:54.297445,Gonostomatidae_Margrethia_obtusirostra:54.297445):20.014374,(Gonostomatidae_Gonostoma_elongatum:69.948163,Gonostomatidae_Cyclothone_microdon:69.948163):4.363656):3.018918):6.469264,(Phosichthyidae_Pollichthys_mauli:77.722827,((Sternoptychidae_Argyropelecus_gigas:25.230481,Sternoptychidae_Maurolicus_weitzmani:25.230481):47.329876,(Stomiidae_Chauliodus_macouni_danae:63.126299,(Stomiidae_Stomias_boa:36.381906,(Stomiidae_Chirostomias_pliopterus:31.578039,((Stomiidae_Melanostomias_margaritifer:21.790927,Stomiidae_Leptostomias_longibarba:21.790927):6.240428,((Stomiidae_Photonectes_margarita:19.053467,Stomiidae_Tactostoma_macropus:19.053467):7.977888,(Stomiidae_Malacosteus_niger:25.031355,(Stomiidae_Eustomias_polyaster:24.031355,(Stomiidae_Aristostomias_scintillans:23.031355,Stomiidae_Bathophilus_flemingi_pawneei:23.031355):1):1):2):1):3.546684):4.803867):26.744393):9.434058):5.16247):6.077174):45.6):81.9,((Ateleopodidae_Ateleopus_japonicus:8.089622,Ateleopodidae_Ijimaia_antillarum:8.089622):184.210379,(((Synodontidae_Trachinocephalus_myops:34.928634,Synodontidae_Synodus_foetens:34.928634):78.971367,((Paraulopidae_Paraulopus_oblongus:101.985288,(Synodontidae_Saurida_gracilis:50.257509,Synodontidae_Harpadon_microchir:50.257509):51.727779):8.326796,(((Aulopidae_Aulopus_filamentosus:47.160387,Bathysauridae_Bathysaurus_ferox:47.160387):10.843902,(Ipnopidae_Ipnops_murrayi:13.82,Ipnopidae_Ipnops_agassizi:13.82):44.184289):23.635948,((Ipnopidae_Bathypterois_atricolor:69.328167,(Giganturidae_Gigantura_indica:13.525058,Giganturidae_Gigantura_chuni:13.525058):55.803109):6.462647,(Scopelarchidae_Benthalbella_macropinna:62.490696,(Chlorophthalmidae_Chlorophthalmus_agassizi:61.13635,(Sudidae_Sudis_atrox:55.026295,((Alepisauridae_Anotopterus_pharao:45.661943,(Alepisauridae_Omosudis_lowii:36.284216,Alepisauridae_Alepisaurus_ferox:36.284216):9.377727):8.238175,(Paralepididae_Stemonosudis_intermedia_macrura:50.122687,(Evermannellidae_Evermannella_balbo:46.686813,Paralepididae_Paralepis_coregonoides:46.686813):3.435874):3.777431):1.126177):6.110055):1.354346):13.300118):5.849423):28.671847):3.587917):68.9,(((Neoscopelidae_Neoscopelus_macrolepidotus:42.408365,Neoscopelidae_Scopelengys_tristis:42.408365):31.191636,((Myctophidae_Lampadena_speculigera:39.81673,Myctophidae_Lampanyctus_macdonaldi:39.81673):11.872,(Myctophidae_Benthosema_glaciale:28.30241,Myctophidae_Myctophum_punctatum:28.30241):23.38632):21.911271):99.5,(((Lamprididae_Lampris_guttatus:65.552505,(Regalecidae_Regalecus_russelii:37.682943,Trachipteridae_Trachipterus_trachypterus:37.682943):27.869562):84.747496,(((Mcconichthys_longipinnis:1,(Aphredoderidae_Aphredoderus_sayanus:41.500001,Amblyopsidae_Chologaster_cornuta:41.500001):24.38403355):1,(Lateopisciculus_turrifumosus:1,(Percopsidae_Percopsis_omiscomaycus:14.617149,Percopsidae_Percopsis_transmontana:14.617149):42.83544085):9.431444704):68.10629745,(Zeidae_Zeus_faber:107.100001,(Stylephoridae_Stylephorus_chordatus:78.800001,(Merlucciidae_Merluccius_productus:45.655086,(((Macrouridae_Gadomus_dispar:9.947595,Macrouridae_Bathygadus_favosus:9.947595):28.939269,(Steindachneriidae_Steindachneria_argentea:35.929071,((Macrouridae_Malacocephalus_laevis:17.761718,Macrouridae_Trachonurus_sulcatus:17.761718):10.126413,(Macrouridae_Coelorinchus_caribbaeus:21.883517,Macrouridae_Coryphaenoides_armatus:21.883517):6.004614):8.04094):2.957793):2.613137,((Moridae_Laemonema_goodebeanorum:25.08833,(Moridae_Halargyreus_johnsonii:16.444614,(Moridae_Lepidion_ensiferus:5.35693,Moridae_Antimora_rostrata:5.35693):11.087684):8.643716):13.1716,((Phycidae_Urophycis_tenuis:5.240351,Phycidae_Urophycis_chuss:5.240351):28.607969,(Lotidae_Gaidropsarus_ensis:29.800286,(Lotidae_Lota_lota:23.935071,(Gadidae_Melanogrammus_aeglefinus:5.86839,Gadidae_Gadus_morhua:5.86839):18.066681):5.865215):4.048034):4.41161):3.240071):4.155085):33.144915):28.3):27.890331):15.309669):11.08283,(Polymixiidae_Polymixia_japonica:154.800001,(((Diretmidae_Diretmus_argenteus:53.99855,(Monocentridae_Monocentris_japonica:33.600001,((Trachichthyidae_Hoplostethus_occidentalis_atlanticus:24.361165,Trachichthyidae_Gephyroberyx_darwinii:24.361165):7.919156,(Anoplogastridae_Anoplogaster_cornuta:31.22776,Trachichthyidae_Paratrachichthys_sajademalensis:31.22776):1.052561):1.31968):20.398549):71.275924,((Berycidae_Beryx_decadactylus:70.612394,(Melamphaidae_Poromitra_crassiceps:39.138751,(Melamphaidae_Scopelogadus_beanii:32.603956,Melamphaidae_Melamphaes_suborbitalis:32.603956):6.534795):31.473643):40.295775,(((Rondeletiidae_Rondeletia_bicolor:18.616456,Rondeletiidae_Rondeletia_loricata:18.616456):36.662305,Barbourisiidae_Barbourisia_rufa:55.278761):34.605685,(Cetomimidae_Cetostoma_regani:55.492218,(Cetomimidae_Cetomimus_craneae:10.537863,Cetomimidae_Cetomimus_compuctus:10.537863):44.954355):34.392228):21.023723):14.366305):21.189147,((Holocentridae_Sargocentron_diadema:25.43456,Holocentridae_Holocentrus_rufus:25.43456):119.565441,((Ophidiidae_Brotula_multibarbata:66.227334,(Ophidiidae_Lepophidium_profundorum:23.036156,(Ophidiidae_Genypterus_blacodes:21.590629,Ophidiidae_Ophidion_holbrookii:21.590629):1.445527):43.191178):66.572667,((Batrachoididae_Porichthys_notatus:39.800001,Batrachoididae_Opsanus_tau:39.800001):87,(((Kurtidae_Kurtus_indicus:80.300001,Apogonidae_Apogon_campbelli:80.300001):22,(Eleotridae_Eleotris_acanthopoma_pisonis:64.430477,(Gobiidae_Gobiosoma_bosc:45.630567,Microdesmidae_Microdesmus_longipinnis:45.630567):18.79991):37.869524):19.3,((((Dactylopteridae_Dactylopterus_volitans:68.121568,(Aulostomidae_Aulostomus_maculatus:3.200001,Aulostomidae_Aulostomus_chinensis:3.200001):64.921567):6.178433,((Fistulariidae_Fistularia_petimba:65.463647,Mullidae_Mullus_auratus:65.463647):6.26906,(Callionymidae_Callionymus_sp_bairdi:69.154942,Syngnathidae_Syngnathus_fuscus:69.154942):2.577765):2.567294):20.27,(Chiasmodontidae_Chiasmodon_sp:43.900001,((Centrolophidae_Icichthys_lockingtoni:34.370774,Pomatomidae_Pomatomus_saltatrix:34.370774):6.934458,(((Ariommatidae_Ariomma_bondi:25.644121,Nomeidae_Psenes_cyanophrys:25.644121):5.370208,Scombridae_Auxis_rochei:31.014329):6.588438,((Stromateidae_Peprilus_paru:35.602767,(Gempylidae_Paradiplospinus_gracilis:31.499638,Bramidae_Brama_brama:31.499638):4.103129):1,(Icosteidae_Icosteus_aenigmaticus:33.972252,Trichiuridae_Trichiurus_lepturus:33.972252):2.630515):1):3.702465):2.594769):50.67):21.916687,((((((Synbranchidae_Monopterus_albus:69.146846,Indostomidae_Indostomus_paradoxus:69.146846):4.548479,(Mastacembelidae_Macrognathus_siamensis:18.057555,Mastacembelidae_Mastacembelus_erythrotaenia:18.057555):55.63777):7.004676,((Anabantidae_Ctenopoma_acutirostre_kingsleyae:62.782323,(Helostomatidae_Helostoma_temminkii:59.687826,Osphronemidae_Trichopodus_pectoralis:59.687826):3.094497):7.86905,(Channidae_Channa_striata:67.428514,Nandidae_Nandus_nandus:67.428514):3.222859):10.048628):15.7,((Nematistiidae_Nematistius_pectoralis:60.067614,((Coryphaenidae_Coryphaena_hippurus:19.124214,Rachycentridae_Rachycentron_canadum:19.124214):12.775787,(Echeneidae_Remora_osteochir_australis:10.178122,Echeneidae_Echeneis_naucrates:10.178122):21.721879):28.167613):9.732387,((Sphyraenidae_Sphyraena_sphyraena:65.900885,(Menidae_Mene_maculata:60.339531,Polynemidae_Polydactylus_octonemus:60.339531):5.561354):2.899116,((((Leptobramidae_Leptobrama_muelleri:46.58559,Toxotidae_Toxotes_jaculatrix:46.58559):5.096327,(Xiphiidae_Xiphias_gladius:27.833842,Istiophoridae_Istiophorus_platypterus:27.833842):23.848075):5.298938,Carangidae_Trachinotus_falcatus:56.980855):4.861848,((Eolates_gracilis:1,(Centropomidae_Psammoperca_waigiensis:33.068144,(Centropomidae_Lates_calcarifer:13.595548,Centropomidae_Lates_microlepis:13.595548):19.472596):22.32678256):5.44777644,(Heteronectes_chaneti:5.486088885,(Amphistium_paradoxum:2.931440045,(Psettodidae_Psettodes_erumei:57.842703,(Citharidae_Lepidoblepharon_ophthalmolepis:53.600001,((Cynoglossidae_Cynoglossus_interruptus:42.400002,(Soleidae_Solea_solea:24.944331,Soleidae_Soleichthys_heterorhinos:24.944331):17.455671):9.674607,((Scophthalmidae_Scophthalmus_aquosus:23.305906,Scophthalmidae_Lepidorhombus_boscii:23.305906):26.973285,((Paralichthyidae_Paralichthys_dentatus:40.749719,Bothidae_Bothus_lunatus:40.749719):4.387561,(Paralichthyidae_Pseudorhombus_pentophthalmus:21.404631,(((Pleuronectidae_Glyptocephalus_zachirus:5.096079,Pleuronectidae_Microstomus_pacificus:5.096079):2.538752,(Pleuronectidae_Lyopsetta_exilis:6.085211,Pleuronectidae_Hippoglossus_hippoglossus:6.085211):1.54962):2.215451,(Pleuronectidae_Limanda_limanda:8.798272,((Pleuronectidae_Platichthys_stellatus:3.717184,Pleuronectidae_Pleuronectes_platessa:3.717184):3.081088,(Pleuronectidae_Lepidopsetta_bilineata:4.798272,Pleuronectidae_Psettichthys_melanostictus:4.798272):2):2):1.05201):11.554349):23.732649):5.141911):1.795418):1.525392):4.242702):1):1):1):1):6.957298):1):26.6):12.043384,(((Polycentridae_Polycentrus_schomburgkii:94.214904,(Pholidichthyidae_Pholidichthys_leucotaenia:88.700001,Cichlidae_Cichla_temensis:88.700001):5.514903):1,(((Atherinopsidae_Atherinopsis_californiensis:48.715388,Atherinopsidae_Menidia_menidia:48.715388):22.225577,(Isonidae_Iso_sp:49.317292,(Atherinidae_Atherinomorus_stipes:43.78646,(Melanotaeniidae_Melanotaenia_splendida:39.870052,Pseudomugilidae_Pseudomugil_gertrudae:39.870052):3.916408):5.530832):21.623673):6.46052,(((Poeciliidae_Poecilia_latipinna_reticulata:14.292152,(Poeciliidae_Heterandria_formosa:11.366823,(Poeciliidae_Gambusia_affinis:5.736451,Poeciliidae_Belonesox_belizanus:5.736451):5.630372):2.925329):30.13151,(Cyprinodontidae_Cyprinodon_variegatus:40.469994,Fundulidae_Fundulus_parvipinnis:40.469994):3.953668):31.977823,(Adrianichthyidae_Oryzias_latipes:71.878571,((Zenarchopteridae_Dermogenys_collettei:29.883878,((Belonidae_Tylosurus_crocodilus:6.568056,Belonidae_Ablennes_hians:6.568056):17.429484,(Scomberesocidae_Cololabis_saira:2.646712,Scomberesocidae_Scomberesox_saurus:2.646712):21.350828):5.886338):17.178962,(Hemiramphidae_Hyporhamphus_affinis:36.892311,((Hemiramphidae_Hemiramphus_brasiliensis:13.391766,Hemiramphidae_Oxyporhamphus_micropterus:13.391766):10.259915,((Exocoetidae_Cypselurus_callopterus:6.413654,Exocoetidae_Exocoetus_monocirrhus:6.413654):4.605967,Exocoetidae_Cheilopogon_pinnatibarbatus:11.019621):12.63206):13.24063):10.170529):24.815731):4.522914):1):17.813419):1.999954,((Pseudochromidae_Halidesmus_scapularis:84.850287,Pomacentridae_Pomacentrus_brachialis:84.850287):8.187103,(Embiotocidae_Cymatogaster_aggregata:91.529056,(Mugilidae_Mugil_cephalus:89.757655,(Plesiopidae_Plesiops_coeruleolineatus:86.977996,(Opistognathidae_Opistognathus_maxillosus:79.196597,(((Gobiesocidae_Lepadichthys_lineatus:37.560081,Gobiesocidae_Gobiesox_maeandricus:37.560081):35.985217,Tripterygiidae_Enneanectes_altivelis:73.545298):3.122761,((Clinidae_Clinus_cottoides:50.741024,(Dactyloscopidae_Platygillellus_rubrocinctus:45.641062,Chaenopsidae_Chaenopsis_alepidota:45.641062):5.099962):17.520576,(Blenniidae_Stanulus_seychellensis:31.042358,Blenniidae_Blenniella_cyanostigma:31.042358):37.219242):8.406459):2.528538):7.781399):2.779659):1.771401):1.508334):4.177468):11.228527):3.450865,(Gerreidae_Eucinostomus_argenteus:108.000002,(((Labridae_Lachnolaimus_maximus:59.822938,Odacidae_Haletta_semifasciata:59.822938):16.826114,Scaridae_Scarus_globiceps:76.649052):30.35095,(((Uranoscopidae_Uranoscopus_sulphureus:83.996275,(Ammodytidae_Ammodytes_hexapterus:78.584442,Pinguipedidae_Parapercis_punctulata:78.584442):5.411833):11.903726,(((Acropomatidae_Acropoma_japonicum:90.360051,((Percophidae_Acanthaphritis_unoorum:66.16617,Creediidae_Limnichthys_sp:66.16617):18.796708,(Glaucosomatidae_Glaucosoma_buergeri:66.617288,Pempheridae_Pempheris_schomburgkii:66.617288):18.34559):5.397173):2.814411,(((Oplegnathidae_Oplegnathus_punctatus:61.206964,Kuhliidae_Kuhlia_rupestris:61.206964):7.298332,Kyphosidae_Kyphosus_sectatrix:68.505296):16.157201,(Percichthyidae_Percichthys_trucha:69.999181,((Cirrhitidae_Cirrhitichthys_falco:66.467051,Cheilodactylidae_Cheilodactylus_fasciatus:66.467051):2.53213,(Enoplosidae_Enoplosus_armatus:63.04721,Centrarchidae_Acantharchus_pomotis:63.04721):5.951971):1):14.663316):8.511965):1.725539,(Serranidae_Pseudogramma_polyacantha:82.200001,(Percidae_Perca_fluviatilis:78.577195,((Bovichtidae_Bovichtus_diacanthus:63.021097,(Nototheniidae_Notothenia_coriiceps:9.671082,(Harpagiferidae_Harpagifer_antarcticus:8.390321,(Bathydraconidae_Gymnodraco_acuticeps:7.390321,Channichthyidae_Chionodraco_rastrospinosus:7.390321):1):1.280761):53.350015):12.936917,(((Peristediidae_Peristedion_ecuadorense:54.532565,Triglidae_Prionotus_carolinus:54.532565):11.953546,(Synanceiidae_Synanceia_verrucosa:62.510934,Scorpaenidae_Scorpaenodes_guamensis:62.510934):3.975177):6.621302,(Platycephalidae_Platycephalus_indicus:70.795272,(Anoplopomatidae_Anoplopoma_fimbria:46.504594,((Bathymasteridae_Rathbunella_hypoplecta:22.798552,(Stichaeidae_Cebidichthys_violaceus:15.58542,(Zoarcidae_Zoarces_americanus_viviparus:12.493576,(Anarhichadidae_Anarrhichthys_ocellatus:9.933504,(Zaproridae_Zaprora_silenus:8.933504,Cryptacanthodidae_Cryptacanthodes_maculatus:8.933504):1):2.560072):3.091844):7.213132):19.028758,(((Aulorhynchidae_Aulorhynchus_flavidus:25.280385,Hypoptychidae_Hypoptychus_dybowskii:25.280385):1.019618,((Gasterosteidae_Culaea_inconstans:19.844899,(Gasterosteidae_Apeltes_quadracus:17.136644,Gasterosteidae_Spinachia_spinachia:17.136644):2.708255):1.782129,(Gasterosteidae_Pungitius_pungitius:16.827665,Gasterosteidae_Gasterosteus_aculeatus:16.827665):4.799363):4.672975):13.53479,(Hexagrammidae_Hexagrammos_decagrammus:27.818969,((Cyclopteridae_Cyclopterus_lumpus:20.762394,Liparidae_Liparis_pulchellus:20.762394):4.037609,(Agonidae_Hypsagonus_quadricornis:20.371382,(Cottidae_Cottus_carolinae:18.19359,Psychrolutidae_Psychrolutes_phrictus:18.19359):2.177792):4.428621):3.018966):12.015824):1.992517):4.677284):24.290678):2.312141):2.850601):2.619181):3.622806):12.7):1):10.100001,((Drepaneidae_Drepane_punctata:67.658011,Ephippidae_Chaetodipterus_faber:67.658011):37.341991,(((Lobotidae_Lobotes_pacificus_surinamensis:86.438004,Sciaenidae_Menticirrhus_undulatus_littoralis:86.438004):5.761997,((Monodactylidae_Monodactylus_sebae:70.333247,(Avitoluvarus_eocaenicus:21.19054805,(Zanclidae_Zanclus_cornutus:55.859022,Acanthuridae_Acanthurus_triostegus:55.859022):4.740979):9.733246):15.366754,((Pomacanthidae_Pomacanthus_semicirculatus:54.434118,(Leiognathidae_Leiognathus_equulus:32.700001,Chaetodontidae_Chelmon_rostratus:32.700001):21.734117):17.438962,(Emmelichthyidae_Erythrocles_schlegelii:69.871718,(Malacanthidae_Malacanthus_plumieri:68.303733,(Haemulidae_Haemulon_aurolineatum:64.600001,Lutjanidae_Lutjanus_griseus:64.600001):3.703732):1.567985):2.001362):13.826921):6.5):11.800001,((Sillaginidae_Sillago_sihama:85.853361,(Nemipteridae_Pentapodus_caninus:80.270573,(Lethrinidae_Lethrinus_erythropterus:74.370539,Sparidae_Stenotomus_chrysops:74.370539):5.900034):5.582788):17.146641,(Siganidae_Siganus_spinus:102.000002,((Scatophagidae_Scatophagus_argus:75.860371,Priacanthidae_Heteropriacanthus_cruentatus:75.860371):25.139631,((Caproidae_Antigonia_rubescens:10.905674,Caproidae_Antigonia_capros:10.905674):89.094328,(((Lophiidae_Lophiodes_reticulatus:22.462185,Lophiidae_Lophius_gastrophysus:22.462185):42.032119,((Antennariidae_Histrio_histrio:12.333102,Antennariidae_Antennatus_coccineus:12.333102):33.466899,(Chaunacidae_Chaunax_suttkusi:38.499563,(Gigantactinidae_Gigantactis_sp:32.911618,((Ceratiidae_Cryptopsaras_couesii:20.548708,Ceratiidae_Ceratias_holboelli:20.548708):10.416157,((Himantolophidae_Himantolophus_albinares_sagamius:14.456802,Melanocetidae_Melanocetus_murrayi:14.456802):9.124159,(Oneirodidae_Oneirodes_macrosteus:10.164357,Oneirodidae_Dolopichthys_sp:10.164357):13.416604):7.383904):1.946753):5.587945):7.300438):18.694303):34.505698,(((Prohollardia_avita:38.32056439,(Protacanthodes_nimesensis:1,Triacanthidae_Triacanthus_biaculeatus:43.33362033):21.15935067):10.123893,(Aracanidae_Aracana_aurita:54.800001,(Ostraciidae_Ostracion_cubicus:51.000002,Ostraciidae_Rhinesomus_triqueter:51.000002):3.799999):19.816863):3.183137,(((Diodontidae_Diodon_holocanthus:12.198387,Diodontidae_Chilomycterus_schoepfii:12.198387):43.701614,(Tetraodontidae_Canthigaster_bennetti:24.22571,Tetraodontidae_Tetraodon_miurus:24.22571):31.674291):17.334859,((Molidae_Ranzania_laevis:22,Molidae_Mola_mola:22):46.61027,((Monacanthidae_Aluterus_scriptus:27.823604,Monacanthidae_Stephanolepis_hispidus:27.823604):15.976396,(Balistidae_Xanthichthys_ringens:20.7,(Balistidae_Balistes_vetula:19.7,Balistidae_Sufflamen_fraenatum:19.7):1):23.1):24.81027):4.62459):4.565141):21.200001):1):1):1):1):1):1):1):1):1):3.894248):4.592438):5.113313):5.2):6):12.2):1.46362):8.33638):6.58283):11.71717):9.7):9.5):19):3.553787):17.946213):17.8):23.2):9.5):1):1):37.2):1):1):1,(Fukangichthys_longidorsalis:10.19632545,(Scanilepis_dubia:45.92728495,Evenkia_eunotoptera:1):1):71.35894525):1,Australosomus:74.63967155):1):22.6):1):1):1):12.89208789):20.43649194):1.399082944):1):1):1):4.172338218):1):47);
[truncated: 909,564 more chars]
