# Supplementary material for: On trends and patterns in macroevolution: Williston’s law and the branchiostegal series of extant and extinct osteichthyans
Source: BMC Evol Biol. 2019 Jun 10;19:117. doi: 10.1186/s12862-019-1436-x (PMC6558815; doi:10.1186/s12862-019-1436-x)
Supplement: Supplementary file 2 — Data and scripts used in the subsampling analyses. This consists of 4 files (a,b,c,d). Data files include the branchiostegal count and stratigraphic information collected for extant and extinct species (bst_analysis.csv, tabular data), and the time-scaled trees (trees.tre, Newick format) used in the analyses. The 2 R script files also included were used for running the jackniffing analyses that subsample extinct (fossil_subsampling_1.R) or extant species (extant_subsampling_1.R). The “species” column in the CSV file contains the species in the tree to which the data were mapped. The “old_label” column contains the names of the taxa as they appeared in our original sources. An anonymous referee kindly noted that our data had an erroneous value of 1 branchiostegal ray for Lepidosiren paradoxa, which does not have any branchiostegals in reality. We did not rerun our analyses because they are very time-consuming, and this small correction is unlikely to have any significant effect. However, we amended the CVS file that we provide here, so that the error does not propagate to any possible future uses of the data by other researchers. (ZIP 162 kb) [file 12862_2019_1436_MOESM2_ESM.zip › Additional file 2/Additional file 2c fossil_subsampling_1.R.docx]

print(paste("Analysis started at", Sys.time()))

library(ape)

library(geiger)

library(phytools)

library(mvMORPH)

library(phangorn)

summarise_fit <- function (fit) {

out <- matrix(c(sapply(fit, function(x) x$theta[1]),

sapply(fit, function(x) ifelse(is.na(x$theta[2]), NA, x$theta[2])),

sapply(fit, function(x) x$sigma),

sapply(fit, function(x) ifelse(is.null(x$beta), NA, x$beta)),

sapply(fit, function(x) ifelse(is.null(x$alpha), NA, x$alpha)),

sapply(fit, function(x) ifelse(is.null(x$trend), NA, x$trend)),

sapply(fit, function(x) x$LogLik),

sapply(fit, function(x) x$AICc)

),

nrow = length(fit), ncol = 8)

colnames(out) <- c("theta_0", "theta_1", "sigma", "beta", "alpha", "trend", "lnL", "AICc")

rownames(out) <- names(fit)

aic <- unlist(out[, 8])

rel_lik <- exp(-0.5 * (aic - max(aic)))

a_weight <- rel_lik / sum(rel_lik)

out <- cbind(out, AICc_weight = a_weight)

return(out)

}

fit_models <- function(model, tree, data) {

conv <- 1

while (conv < 11) {

switch(model,

BM = {

foo <- mvBM(tree = tree, data = data, model = "BM1", param = list(trend = FALSE), scale.height = TRUE)

if(foo$convergence) conv <- conv + 1

else break

},

drift = {

foo <- mvBM(tree = tree, data = data, model = "BM1", param = list(trend = TRUE), scale.height = TRUE)

if(foo$convergence) conv <- conv + 1

else break

},

OU1 = {

foo <- mvOU(tree = tree, data = data, model = "OU1", scale.height = TRUE, method = "univarpf", param = list(root = TRUE))

if(foo$convergence) conv <- conv + 1

else break

},

OU2 = {

foo <- mvOU(tree = tree, data = data, model = "OU1", scale.height = TRUE, method = "univarpf", param = list(root = FALSE))

if(foo$convergence) conv <- conv + 1

else break

},

EB = {

foo <- mvEB(tree = tree, data = data, scale.height = TRUE, param = list(up = 10, low = -10))

if(foo$convergence) conv <- conv + 1

else break

}

)

}

if (conv == 10) {

foo$theta <- foo$sigma <- foo$AICc <- NA

foo$LogLik <- NA

}

return(foo)

}

set.seed(29537726)

tr <- read.tree("trees.tre")[1:25]

tr <- multi2di(tr)

bst <- read.csv("bst_analysis_mat.csv", as.is = TRUE, row.names = 1)

x <- bst$mean

names(x) <- rownames(bst)

x <- x[! is.na(x)]

spp <- tr[[1]]$tip.label

fossils <- spp[bst[spp, "geol_unit"] != "Recent"]

ntrees <- length(tr)

ssize <- ceiling(66*((0:9)/10))

nsamples <- 10

models <- c("BM", "EB", "OU1", "drift")

table1 <- array(dim = c(length(models), 9, ntrees, length(ssize), nsamples), dimnames = list(models, c("theta_0", "theta_1", "sigma", "beta", "alpha", "trend", "lnL", "AICc", "AICc_weight"), NULL))

write.table(table1[, , 1, 1, 1], "res/fossil_subsampling.txt", append = TRUE, sep = "\t", quote = FALSE, col.names = TRUE)

fit1 <- vector("list", length= ntrees*nsamples)

for (i in 1:ntrees) {

for (j in 1:length(ssize)) {

fo <- file("res/fossil_subsampling_1.txt", open ="a")

writeLines(paste("With", ssize[j], "fossils:"), con = fo)

close(fo)

fit1 <- NULL

tmp <- array(dim = c(length(models), 9, nsamples), dimnames = list(models, c("theta_0", "theta_1", "sigma", "beta", "alpha", "trend", "lnL", "AICc", "AICc_weight"), NULL))

for (k in 1:nsamples) {

fs <- sample(fossils, ssize[j], replace = FALSE)

spp_drop <- fossils[! fossils %in% fs]

trfs <- drop.tip(tr[[i]], spp_drop)

foo <- treedata(trfs, data = x, sort = TRUE, warnings = FALSE)

y <- foo$data

trx <- foo$phy

fit1 <- lapply(models, fit_models, trx, y)

names(fit1) <- models

tmp[, , k] <- summarise_fit(fit1)

write.table(tmp[, , k], "res/fossil_subsampling_1.txt", append = TRUE, sep = "\t", quote = FALSE, col.names = FALSE)

}

table1[, , i, j, ] <- tmp

cat(paste0("\rProgress: iteration ", nsamples * (i -1) + j, " (", round(100*(nsamples * (i -1) + j)/(nsamples*ntrees), 3), "%) "))

}

}

print(Sys.time())

war <- warnings()

save("fit_results", "fit1", "war", file = "res/fossil_subsampling_1.Rdata")

print(paste("Analysis finished at", Sys.time()))
