# Supplementary figures and images for: On trends and patterns in macroevolution: Williston’s law and the branchiostegal series of extant and extinct osteichthyans
Source: BMC Evol Biol. 2019 Jun 10;19:117. doi: 10.1186/s12862-019-1436-x (PMC6558815; doi:10.1186/s12862-019-1436-x)

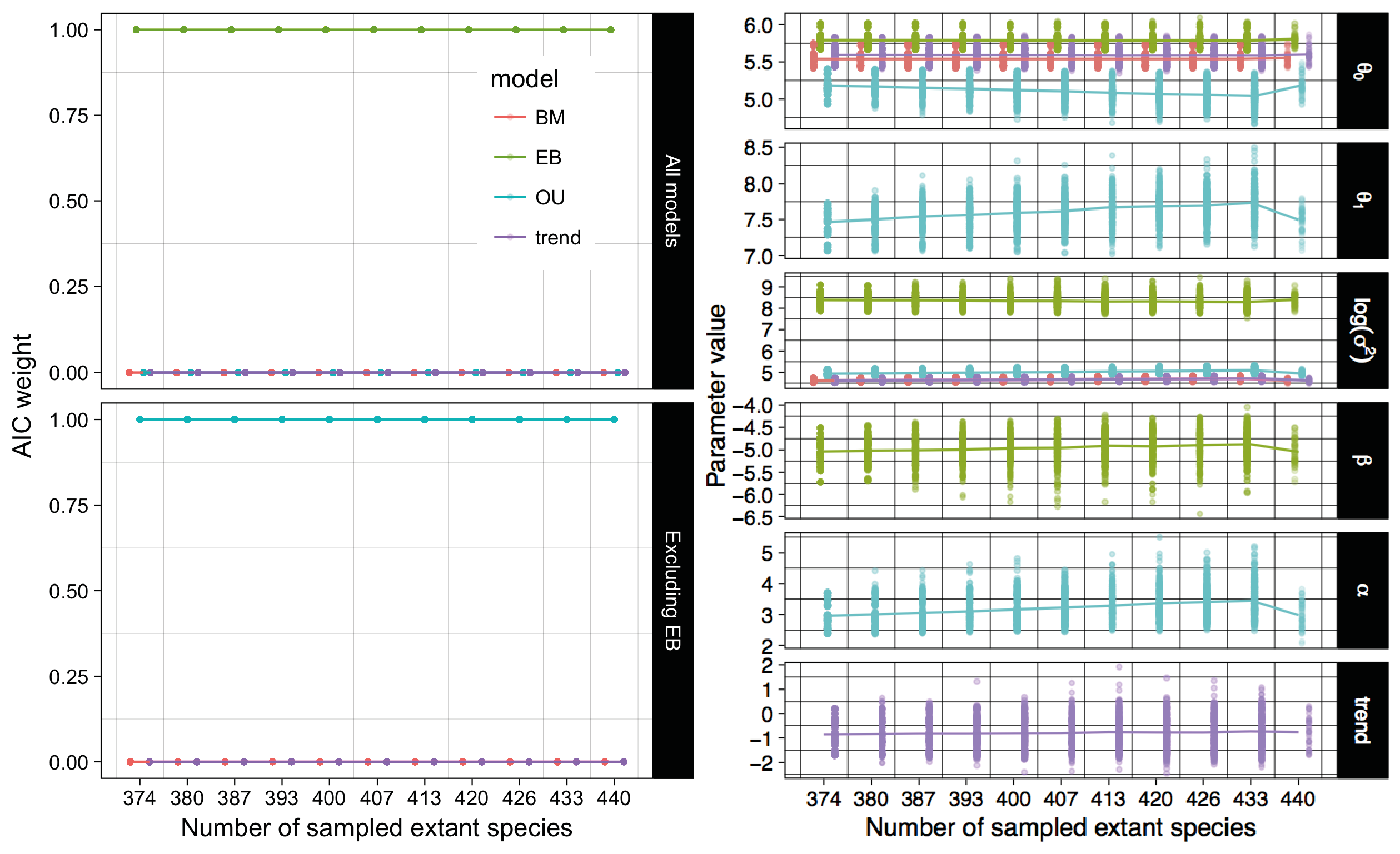

Supplement: Supplementary file 4 — Figure S2. Results of jackkinifing of extant species. Impact of the number of extant species sampled on Akaike weight model support (Akaike weights including all models in top left, Akaike weights excluding EB in bottom left) and the parameter estimates of the macroevolutionary models (right). Refer to Fig. 4 for explanations of the model parameters. (PNG 268 kb) [file 12862_2019_1436_MOESM4_ESM.png]
