# Supplementary material for: On trends and patterns in macroevolution: Williston’s law and the branchiostegal series of extant and extinct osteichthyans
Source: BMC Evol Biol. 2019 Jun 10;19:117. doi: 10.1186/s12862-019-1436-x (PMC6558815; doi:10.1186/s12862-019-1436-x)
Supplement: Supplementary file 5 — Table. Model fitting of Markov models using the mean number ofbranchiostegals, the minimum (observed and “soft”) number of branchiostegals, and the maximum (observed and “soft”) number of branchiostegals. Valuesreported are the median of the model log-likelihoods and rates fitted to 50recalibrated trees. Rate 1 is the rate of branchiostegal gain in the asymmetricmodel, or gain and loss in the symmetric model. Rate 2 is the rate of branchiostegalloss in the asymmetric model.. (DOCX 268 kb) [file 12862_2019_1436_MOESM5_ESM.docx]

| **Model** | **Log-likelihood** | **Rate 1** | **Rate 2** |
| --- | --- | --- | --- |
| Mean number of branchiostegals | | | |
| symmetric | -1273.90 | 26.08378 | – |
| asymmetric | -1273.50 | 25.56799 | 26.7939 |
| Minimum number of branchiostegals | | | |
| symmetric | -1295.34 | 30.4184 | – |
| asymmetric | -1295.14 | 29.5307 | 31.4416 |
| Maximum number of branchiostegals | | | |
| symmetric | -1270.76 | 24.94465 | – |
| asymmetric | -1270.69 | 24.84188 | 25.11154 |
